# Supplementary material for: CRISPRidentify: identification of CRISPR arrays using machine learning approach
Source: Nucleic Acids Res. 2020 Dec 8;49(4):e20. doi: 10.1093/nar/gkaa1158 (PMC7913763; doi:10.1093/nar/gkaa1158)
Supplement: gkaa1158_Supplemental_Files [file gkaa1158_supplemental_files.zip › CRISPRidentify_Suppl-1.pdf]

CRISPRidentify: Identification of CRISPR arrays using machine learning approach – Supplementary Material 1

Alexander Mitrofanov, Omer S Alkhnbashi, Sergey Shmakov, Kira M. Makarova, Eugene V. Koonin and Rolf Backofen

1 Enhancement of Vmatch results

Here we demonstrate the effect of the enhancement step on the synthetic CRISPR array. The combination of the mismatches between repeats and similarities at the beginning or end positions of the spacer sequences challenge the repeat candidate search with Vmatch and lead in absence of the correct sequence in the result domain. In order to overcome such potential loss, we apply enhancement step shown here. First, we demonstrate an example of a repeat-spacer representation of a synthetic CRISPR array. We then highlight the irregularities in the array such as similarities between the leading and trailing parts of spacers and mismatches at the end of the repeat sequences. After that we demonstrate the corresponding Vmatch result. Note that because of the highlighted challenges the Vmatch result does not include the correct repeat sequence. Therefore, we enhance the repeat candidate set. Firstly, we align the Vmatch candidate set. We then utilize the alignment in order to build the maximum and the minimum elements. Based on the maximum and the minimum elements we then complement the candidate set by all the sequences which are substring of the maximum element and contain minimum element as their substring. The correct candidate is shown in green among the all candidates which were obtained during the enhancement.

CRISPR Array

```
Repeats                                 Spacers
GTCTCAATAACCCTAATCCCTTTCAGGGATTGAAAC    AGAATATCACTGTACGGTTcttGCGTACCTCTTG
GTCTCAATAACCCTAATCCCTTTCAGGGATTGAAAC    AGATAAGACAAACAACACCGTTAGAAACATCATCAC
GTCTCAATAACCCTAATCCCTTTCAGGGATTGAAAC    ACCATAAAAGACTCAACAGGTGTGGCGATCGCAATC
GTCTCAATAACCCTAATCCCTTTCAGGGATTGAAGG    GGTTAGATGCTGCGATAGCTGACGCTTCAAGTGCC
GTCTCAATAACCCTAATCCCTTTCAGGGATTGAAGG    GCTACTCCGGTTTGATGGACCACTAAAGCACCACTAG
GTCTCAATAACCCTAATCCCTTTCAGGGATTGAAAC    GTCCGGTGAGCCTCATCTAACACAACAACCTTATAG
GTCTCAATAACCCTAATCCCTTTCAGGGATTGAAAC    CACGCAGCGGGCGAAAACATCATGAATCATGAAC
GTCTCAATAACCCTAATCCCTTTCAGGGATTGAAAC
```

CRISPR Array Dot Representation with highlighted challenges for the correct repeat sequence identification

```
Repeats                                 Spacers
....................................    AGAATATCACTGTACGGTTcttGCGTACCTCTTG
....................................    AGATAAGACAAACAACACCGTTAGAAACATCATCAC
....................................    ACCATAAAAGACTCAACAGGTGTGGCGATCGCAATC
..................................GG    GGTTAGATGCTGCGATAGCTGACGCTTCAAGTGCC
..................................GG    GCTACTCCGGTTTGATGGACCACTAAAGCACCACTAG
....................................    GTCCGGTGAGCCTCATCTAACACAACAACCTTATAG
....................................    CACGCAGCGGGCGAAAACATCATGAATCATGAAC
....................................    CGG...        (flank)
```

Corresponding Vmatch results:

```
GTCTCAATAACCCTAATCCCTTTCAGGGATTGAAACAGAATA
GTCTCAATAACCCTAATCCCTTTCAGGGATTGAAACA
CGTCTCAATAACCCTAATCCCTTTCAGGGATTGAA
CGTCTCAATAACCCTAATCCCTTTCAGGGATTGAAGGG
GTCTCAATAACCCTAATCCCTTTCAGGGATTGAA
TAGGTCTCAATAACCCTAATCCCTTTCAGGGATTGAAAC
GTCTCAATAACCCTAATCCCTTTCAGGGATTGAAACC
```

Repeat candidate list enhancement:

Alignment:

```
   GTCTCAATAACCCTAATCCCTTTCAGGGATTGAAACAGAATA
   GTCTCAATAACCCTAATCCCTTTCAGGGATTGAAACA
  CGTCTCAATAACCCTAATCCCTTTCAGGGATTGAA
  CGTCTCAATAACCCTAATCCCTTTCAGGGATTGAAGGG
   GTCTCAATAACCCTAATCCCTTTCAGGGATTGAA
TAGGTCTCAATAACCCTAATCCCTTTCAGGGATTGAAAC
   GTCTCAATAACCCTAATCCCTTTCAGGGATTGAAACC
```

```
Maximum element:
TACGTCTCAATAACCCTAATCCCTTTCAGGGATTGAAACAGAATA
Minimum element:
GTCTCAATAACCCTAATCCCTTTCAGGGATTGAA

Enhanced candidate set:
GTCTCAATAACCCTAATCCCTTTCAGGGATTGAAA
GTCTCAATAACCCTAATCCCTTTCAGGGATTGAAAC
GTCTCAATAACCCTAATCCCTTTCAGGGATTGAAACA
GTCTCAATAACCCTAATCCCTTTCAGGGATTGAAACAG
GTCTCAATAACCCTAATCCCTTTCAGGGATTGAAACAGA
GTCTCAATAACCCTAATCCCTTTCAGGGATTGAAACAGAA
GTCTCAATAACCCTAATCCCTTTCAGGGATTGAAACAGAAT
GTCTCAATAACCCTAATCCCTTTCAGGGATTGAAACAGAATA

GGTCTCAATAACCCTAATCCCTTTCAGGGATTGAAA
GGTCTCAATAACCCTAATCCCTTTCAGGGATTGAAAC
GGTCTCAATAACCCTAATCCCTTTCAGGGATTGAAACA
GGTCTCAATAACCCTAATCCCTTTCAGGGATTGAAACAG
GGTCTCAATAACCCTAATCCCTTTCAGGGATTGAAACAGA
GGTCTCAATAACCCTAATCCCTTTCAGGGATTGAAACAGAA
GGTCTCAATAACCCTAATCCCTTTCAGGGATTGAAACAGAAT
GGTCTCAATAACCCTAATCCCTTTCAGGGATTGAAACAGAATA

AGGTCTCAATAACCCTAATCCCTTTCAGGGATTGAAA
AGGTCTCAATAACCCTAATCCCTTTCAGGGATTGAAAC
AGGTCTCAATAACCCTAATCCCTTTCAGGGATTGAAACA
AGGTCTCAATAACCCTAATCCCTTTCAGGGATTGAAACAG
AGGTCTCAATAACCCTAATCCCTTTCAGGGATTGAAACAGA
AGGTCTCAATAACCCTAATCCCTTTCAGGGATTGAAACAGAA
AGGTCTCAATAACCCTAATCCCTTTCAGGGATTGAAACAGAAT
AGGTCTCAATAACCCTAATCCCTTTCAGGGATTGAAACAGAATA

TACGTCTCAATAACCCTAATCCCTTTCAGGGATTGAAA
TACGTCTCAATAACCCTAATCCCTTTCAGGGATTGAAAC
TACGTCTCAATAACCCTAATCCCTTTCAGGGATTGAAACA
TACGTCTCAATAACCCTAATCCCTTTCAGGGATTGAAACAG
TACGTCTCAATAACCCTAATCCCTTTCAGGGATTGAAACAGA
TACGTCTCAATAACCCTAATCCCTTTCAGGGATTGAAACAGAA
TACGTCTCAATAACCCTAATCCCTTTCAGGGATTGAAACAGAAT
TACGTCTCAATAACCCTAATCCCTTTCAGGGATTGAAACAGAATA
```

2 Feature Selection

Here we demonstrate in Table S1 the feature domains for three Extra Tree classifiers which achieved the best performance after feature selection with a wrapper approach.

Table S1: Models generated by Feature Selection

**Model 8 features**	**Model 9 features**	**Model 10 features**
Repeat similarity	Number of repeats	Repeat length
Average spacer length	Repeat similarity	Repeat similarity
Spacer similarity	Average spacer length	AT richness
Number of mismatches	Spacer similarity	Average spacer length
Spacer evenness	Spacer similarity	Spacer similarity
MFE score	MFE score	Number of mismatches
ORF score	ORF score	Spacer evenness
Blast score known repeats	Tandem protein score	Tandem protein score
	Blast score similar to known repeats	Blast score similar to known repeats
		Blast score similar to known repeats

3 Distribution for Spacer-Related Features

In this section, we show the distribution of spacer-related features, again for arrays labeled *Bona fide* as well as arrays labeled *low score*. Figure S1 shows the length distribution for spacers. Figure S2 shows the similarity of spacers.

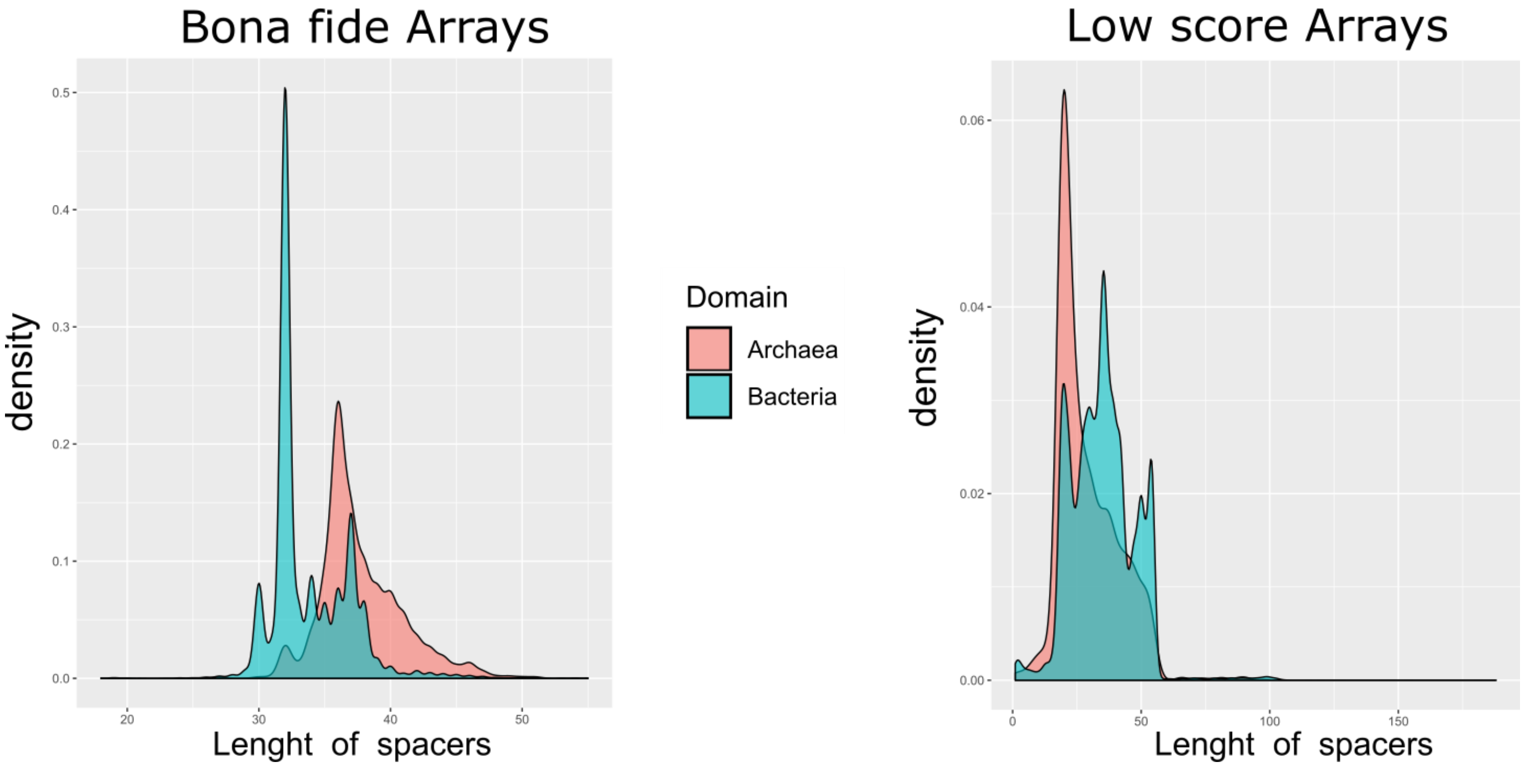

Figure S1: The distribution of spacer lengths in archaea and bacteria for positive and negative arrays. CRISPR arrays were obtained by CRISPRidentify approach on the annotated arrays dataset (see paper Table 1. Bona fide arrays were defined as candidates which obtained certainty score (probability of being an actual CRISPR array) higher or equal to 0.75, Low score arrays obtained certainty scores lower than 0.4

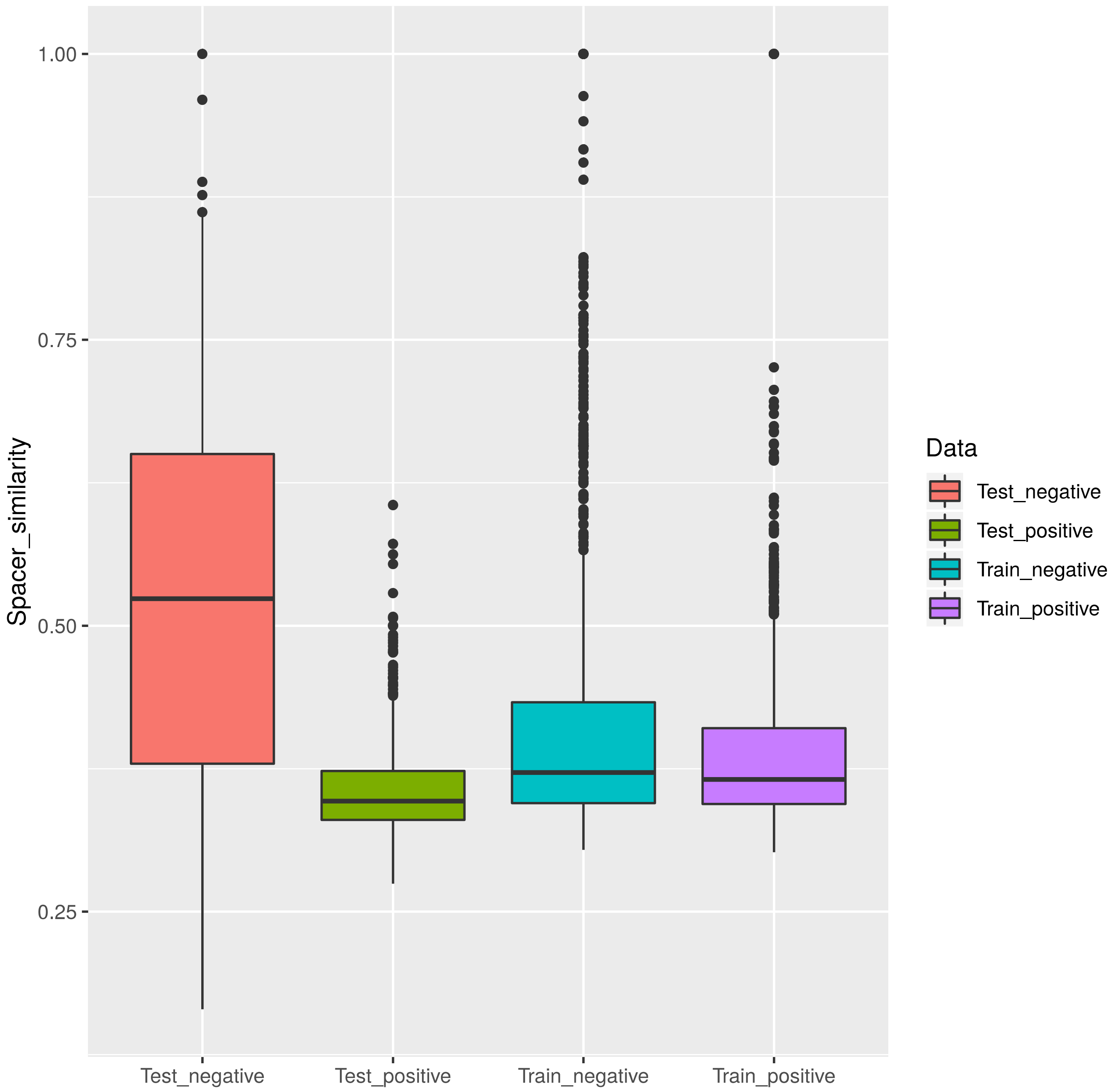

Figure S2: The distribution of spacer similarity for positive and negative arrays. High level of spacer similarity might be providing evidence that considered candidate does not work as a CRISPR array. Although despite the abundance of such cases among both train and test negative cases we still had some cases which demonstrated a high level of spacer similarity even among positive data. Which makes us claim that although such feature definitely has discriminative power it is not sufficient enough by itself for the correct labeling

4 Combined classification for archaeal and bacterial data

We conducted a series of four experiments with the Extra Trees classifier, subjecting different datasets as the training set while keeping the same test set, to assess whether archaeal and bacterial arrays have fundamentally different properties. In this case, two separate classifiers for archaea and bacteria would be beneficial. Otherwise, one classifier containing both types of arrays should show better performance as it is based on more data.

The training sets in these experiments were: **Archaeal dataset** and **Shuffled archaeal dataset**, **Bacterial dataset** and **Shuffled bacterial dataset**, all previous sets combined, and all four combined plus a subset of the **False dataset**. Across all of the experiments the accuracy increased. This development yields three noticeable conclusions. First, the classification accuracy based on training on archaeal data was lower than that achieved by training on bacterial data (91% on archaeal data versus 93% on bacterial data). This discrepancy might originate from the approximately two-fold smaller size of the archaeal dataset. Secondly, however, combining the archaeal and bacterial datasets yielded higher accuracy results than training on bacterial data alone (95.8% accuracy from the combination versus 93% from bacterial data alone). Lastly, the highest observed classification accuracy of 99.8% was achieved by including the part of **False dataset** into the training set. This indicates that combining archaeal and bacterial arrays boosts the classification accuracy.

5 CRISPRidentify output

Here we demonstrate the standard text output of a CRISPR array candidate. The user is provided with the dot representation of the repeat sequences to maximize readability of the matching nucleotides. We provide the information about start and end of the array, as well as consensus repeat and average length of repeats and spacers, for each repeat the starting position is shown in the most left column and the number of editing operations is shown in the right-most column.

```
CRISPR: 1, 1949318-1946847, number of Repeats: 38, avg. length of Repeat: 32, avg length of Spacer: 35

1949318   CA..............................  CAGGCATACTGTCTAAAACAGTATTGCTAATGTTAT      s:2 i:0 d:0
1949250   ................................  GACCTCCGATGATGGTCATGGCAGCTGAAATGGCCT      s:0 i:0 d:0
1949182   ................................  CAAAGCAGCTGGGCAGGTGCAAAATTGGTCATAAT       s:0 i:0 d:0
1949115   ................................  CCGATAGCTTTCCAGTCCTTCTATAAACATCTTCAT      s:0 i:0 d:0
1949047   ................................  GGTTCGATGGCTTTTCTTTTCGGTTGCGATGACA        s:0 i:0 d:0
1948981   ................................  TCTGCCGGGTCCCGTCGGCGGTTATCCGGGAAGTA       s:0 i:0 d:0
1948914   ................................  CATATCAACGGTGACCCACGAGACAATAGGCGAGA       s:0 i:0 d:0
1948847   ................................  ATATGGACTGATTAAGTTAGACAAAAAATATGCTTGA     s:0 i:0 d:0
1948778   ................................  CTCCGAAAATTTCGGAAATATAGAAGTATTGGTTCG      s:0 i:0 d:0
1948710   ................................  TAGAGCCCAGATACTGGCTGATTTCGAGGCCGGGAAAT    s:0 i:0 d:0
1948640   ................................  GCCTGTTACTTCTGATTATAACCTCCCGTTATAATCA     s:0 i:0 d:0
1948571   ................................  AAAGATATAAACTGTTATGTTATATCCTTTGAGCAGG     s:0 i:0 d:0
1948502   ................................  AGCAGAAAATCTGCTTAATTTGTATGACAAAACA        s:0 i:0 d:0
1948436   ................................  ATTTTCCACTCTTACAAATATGAACCAGATGGAGG       s:0 i:0 d:0
1948369   ................................  TTGGGGGTGTAACTTATGCCTGAAAAAGTATATCTT      s:0 i:0 d:0
1948301   ................................  GGATACGATTTCTGGATTTACCAATCCCATCAAA        s:0 i:0 d:0
1948235   ................................  GTGAGTTGGATTATAACGTCTATAATCTTATGCTTGA     s:0 i:0 d:0
1948166   ................................  TGTTCAACACTGATATGGTTCGGGCAATACTGGACG      s:0 i:0 d:0
1948098   ................................  CAGCGGACTGGACGGCAGCCAACAAGGATTATATT       s:0 i:0 d:0
1948031   ................................  GGCTATAGTGGCACACTTCCGGCTAACTACTGCGA       s:0 i:0 d:0
1947964   ................................  ATGAGTATCAGGGAGACAACGACTAAAAGGAGGTATCG    s:0 i:0 d:0
1947894   ................................  ATTGTCGGCACGACAACAGGTTCGCCTTCATATTG       s:0 i:0 d:0
1947827   ................................  AGAATTAGCAATGCAGTACACCAAGGACACGTTGG       s:0 i:0 d:0
1947760   ................................  GAGACCTAGAAAACATGATCCTGCAGAAATAGCAC       s:0 i:0 d:0
1947693   ................................  ACGAAAAAACCTGCGAAGTCGCGTGGCAGTAAGCGG      s:0 i:0 d:0
1947625   ................................  AAAGTATATGGAACATGATGAGATGATTATGCCA        s:0 i:0 d:0
1947559   ................................  ATAAATACCGTCCATAGTGTAGCCGTGCTCATACCG      s:0 i:0 d:0
1947491   ................................  TATGACAATATAACAGATGAACTACGAGCCATATAT      s:0 i:0 d:0
1947423   ................................  GAGTTGTCATAAGCAAAGGATGTAATCAGCTTCCG       s:0 i:0 d:0
1947356   ................................  GAGGACAGAATCAAAGTCGCTCTGTTAAGTTATAAG      s:0 i:0 d:0
1947288   ................................  GGAATACGCCAGGCAAAACAGCGATGTCATTCTTATA     s:0 i:0 d:0
1947219   ................................  ATAAGGGCGAATGCGGAGAAATTATAATTCCCTGCG      s:0 i:0 d:0
1947151   ................................  GACTGACGATAAAGGAAATAAGAAGAAGGACATAGAT     s:0 i:0 d:0
1947082   ................................  TTGCAGTGAAGCTTGGTTAGACCGTTATGTATCA        s:0 i:0 d:0
1947016   ................................  CCTACTCTATTTTAGTTTAATGCAGCCTTTAATA        s:0 i:0 d:0
1946950   ................................  CGGCGTACGAAAACCCATCTCCGGCAAGTGAAGTGA      s:0 i:0 d:0
1946882   ................................  TGGCCCGCGGTACGCCGCTGGGTGGACGAGCATG        s:0 i:0 d:0
1946816   ................................                                            s:0 i:0 d:0
______________________________________________________________________________________________
          GTCGCACCCCATGCGGGTGCGTGGATTGAAAC                                            s:2 i:0 d:0

Leader region
GCCAAGTCCCGATTCGATTAATTGGCGGAAATATCCTTCAATACCGACATATATTTTGGTCAAAATCCCAAGTGTTGCCGACATATAATTTGGGCTTTAA

Strand: Reverse

#   Array features:
#   repeat_similarity: 0.996220579071952
#   avg_spacer_len: 35.62162162162162
#   spacer_similarity: 0.3166345521335409
#   number_mismatches: 2.0
#   spacer_evenness: 0.291377
#   mfe_score: -14.5
#   orf_score: 79.74
#   blast_score_1: 30.7
________________________________
#   Certainty Score: 0.9893240652773534
```

6 Importance of insertions and deletions as possible operations

Here we demonstrate two alternative results of the same CRISPR array in the organism AEAA01000028. As can be seen from the example of the CRISPRCasFinder output the limitation of the editing operation does not allow building the correct CRISPR array repeat&spacer representation. Such limitation not only corrupts the corresponding repeat sequence by introducing five mismatches in its tail but also affect the following spacer sequence adding a nucleotide to its end ruining the homogeneity of the spacer distribution length in the entire array.

CRISPRIdentifier

```
CRISPR: 1, 49776-50963, number of Repeats: 20, avg. length of Repeat: 28, avg length of Spacer: 33

49776   ...................... ......  CTCCAGTTGTTCGTCTGGTATTTTTACTTTGAA     s:0 i:0 d:0
49837   ...................... ......  TCCAGTATTGAATATTTAATTAAGTTAGGTTTA     s:0 i:0 d:0
49898   ...................... ......  TACTTATGAAGATGCTAAAAAGCTATTTGGAGA     s:0 i:0 d:0
49959   ...................... ......  TAAGGTTTATGCTGCAACAGTCAATACTTATGA     s:0 i:0 d:0
50020   ...................... ......  TAAAGTTGTAGAACAAAAAGCAGAGCTTAAAAA     s:0 i:0 d:0
50081   ...................... ......  TTATCCCTTAGGTTATGGAGAGGGAGTTAATCA     s:0 i:0 d:0
50142   ...................... ......  TCAGATAATCAACAATAAAAGAAGTATGTACTT     s:0 i:0 d:0
50203   ...................... ......  CAGCAATAGCAGTAGCAATTAAATTAGTAATGG     s:0 i:0 d:0
50264   ...................... ......  CTGGTTTGAGTGGAGGAGAGATAACCGGAAAGA     s:0 i:0 d:0
50325   ...................... ......  TATTCGATTGATGCTCTAAGCATTTGTAAATCA     s:0 i:0 d:0
50386   ......................G......  TATAATTTGTTGCTTCTTTTTCCGCTTCTTCTA     s:0 i:1 d:0
50448   ...................... ......  CAATGATAGGCTTGTTTATGATATTGATAGGCA     s:0 i:0 d:0
50509   ...................... ......  CAAATATCATTTCCACCAGTTTAGGCATTATTT     s:0 i:0 d:0
50570   ...................... ......  CTACTAGGCCCGAAAGCTTTAATTACTCGTTTT     s:0 i:0 d:0
50631   ...................... ......  TACAGCTCCTCCAAGGAAGAAGAAAAACTACCA     s:0 i:0 d:0
50692   ...................... ......  TGCCAGCTTTTAACTACAAGATAGGAACTAATT     s:0 i:0 d:0
50753   ...................... ......  TATCCCTTGCTTCGATATACCTGACTTTGGTCT     s:0 i:0 d:0
50814   ...................... ......  CTTTGTACGCTCTACTTTAGGGTAAAAGATTTT     s:0 i:0 d:0
50875   ..................T... ......  TGCAGAGTATTAAAAAATAAAGTTGCTAGTATG     s:1 i:0 d:0
50936   ...................... ......                                        s:0 i:0 d:0
___________________________________________________________________________________________
        GTACTACCCGCACGAGCGGGGG TGATCC                                        s:1 i:1 d:0
```

CRISPRfinder

```
        Repeats                             Spacers
49775   TGTACTACCCGCACGAGCGGGGGTGATCCC      TCCAGTTGTTCGTCTGGTATTTTTACTTTGA
49836   AGTACTACCCGCACGAGCGGGGGTGATCCT      CCAGTATTGAATATTTAATTAAGTTAGGTTT
49897   AGTACTACCCGCACGAGCGGGGGTGATCCT      ACTTATGAAGATGCTAAAAAGCTATTTGGAG
49958   AGTACTACCCGCACGAGCGGGGGTGATCCT      AAGGTTTATGCTGCAACAGTCAATACTTATG
50019   AGTACTACCCGCACGAGCGGGGGTGATCCT      AAAGTTGTAGAACAAAAAGCAGAGCTTAAAA
50080   AGTACTACCCGCACGAGCGGGGGTGATCCT      TATCCCTTAGGTTATGGAGAGGGAGTTAATC
50141   AGTACTACCCGCACGAGCGGGGGTGATCCT      CAGATAATCAACAATAAAAGAAGTATGTACT
50202   TGTACTACCCGCACGAGCGGGGGTGATCCC      AGCAATAGCAGTAGCAATTAAATTAGTAATG
50263   GGTACTACCCGCACGAGCGGGGGTGATCCC      TGGTTTGAGTGGAGGAGAGATAACCGGAAAG
50324   AGTACTACCCGCACGAGCGGGGGTGATCCT      ATTCGATTGATGCTCTAAGCATTTGTAAATC
50385   AGTACTACCCGCACGAGCGGGGGGTGATCC      TATAATTTGTTGCTTCTTTTTCCGCTTCTTCT
50447   AGTACTACCCGCACGAGCGGGGGTGATCCC      AATGATAGGCTTGTTTATGATATTGATAGGC
50508   AGTACTACCCGCACGAGCGGGGGTGATCCC      AAATATCATTTCCACCAGTTTAGGCATTATT
50569   TGTACTACCCGCACGAGCGGGGGTGATCCC      TACTAGGCCCGAAAGCTTTAATTACTCGTTT
50630   TGTACTACCCGCACGAGCGGGGGTGATCCT      ACAGCTCCTCCAAGGAAGAAGAAAAACTACC
50691   AGTACTACCCGCACGAGCGGGGGTGATCCT      GCCAGCTTTTAACTACAAGATAGGAACTAAT
50752   TGTACTACCCGCACGAGCGGGGGTGATCCT      ATCCCTTGCTTCGATATACCTGACTTTGGTC
50813   TGTACTACCCGCACGAGCGGGGGTGATCCC      TTTGTACGCTCTACTTTAGGGTAAAAGATTT
50874   TGTACTACCCGCACGAGCGTGGGTGATCCT      GCAGAGTATTAAAAAATAAAGTTGCTAGTAT
50935   GGTACTACCCGCACGAGCGGGGGTGATCCT
```

7 Spacer deletion

Here we demonstrate the ability of the tool to predict arrays with complete spacer deletion even if it occurs at the end of the array.

```
CRISPR: 1 , 157500-157803, number of Repeats: 6, avg. length of Repeat: 37, avg length of Spacer: 35

157500   ..T...........................   GGACAGCAACCCGTGTCGGATATCAGACAGAT   s:0 i:1 d:0
157562   .. ...........................   AGATACGCCTTTACGTCGCCCTCTTTGGCGCG   s:0 i:0 d:0
157623   .. ...........................   ATTAAAAAAGATTAATGTTGGTTATAGTTTTA   s:0 i:0 d:0
157684   .. ...............T...........   TAAAACACCGGTTGCGCAACCTCCGCGGGGAT   s:1 i:0 d:0
157745   .. .......................T.GG                                      s:3 i:0 d:0
157774   -- -..........................                                      s:0 i:0 d:3
_____________________________________________________________________________________________
         CG GTTTATCCCCGCTGGCGCGGGGAACAC
```

8 Degenerated repeat and IS element search

Here we demonstrate the ability of the tool to find degenerated repeats. The shown example could only be found with the introduction of the insertion as a possible editing operation which currently cannot be performed by the other tools what aim at the CRISPR array search.

Search for degenerated repeats

CRISPR array might contain highly damaged degenerated repeats which could be usually found at the end of the array. This sequence can be missed during the CRISPR array identification due to a high number of mismatches exceeding the searching criteria. In our pipeline we apply the search for the degenerated repeat sequences utilizing the approximate search method from the CRISPR identification part. In contrast to the identification search here we have only one sequence for the query namely the CRISPR array consensus repeat, while the searched intervals were limited to CRISPR array flanking regions. In addition to that the editing operations threshold is increased in comparison to the original search for the repeats to incorporate highly damaged cases.

IS-Element

Insertion sequence (IS) element is a type of Transposable elements, which plays an essential role in archaeal and bacterial genomes organization and evolution [1]. IS-element may destroy the structure of the CRISPR array. Because of the size of the IS-element the existing CRISPR detection tools would then report the observed effect as two or more independent CRISPR array despite the relatively close position and identity of the repeat sequences.

In order to be able to incorporate the search for IS-Elements in our pipeline we have taken the following steps. We started by downloading all known IS-Element proteins from various databases [2, 3]. Then we clustered the obtained results using the Markov Cluster Algorithm (MCL) [4]. Finally, we used the computed clusters for building the HMM model of IS-Elements using hmmbuild [5].

The described above setup allowed to complete the existing pipeline with the IS-Element identification. In order to find IS-Elements we first search for CRISPR arrays with identical repeat sequences. If such arrays are found we check if they are located close to each other (in our approach we used the threshold of 2000 nucleotides). Then we utilize Prodigal tool to extract protein sequences from the gap region. Finally, the found proteins are classified with our IS-Elements HMM model. If classification reports the IS-elements our algorithm merges the separated parts together.

```
CRISPR: 11, 5116445-5117145, number of Repeats: 12, avg. length of Repeat: 29, avg length of Spacer: 32

5116445   . ..CC.......GT..-G-G...........  AACCGGGAGGCGGTCAGATCGATGTAGGCCTC     s:4 i:2 d:2
5116506   . ...........  .................  ACGATCGACACCCGTACCGGCTGGCTGCTGAT     s:0 i:0 d:0
5116567   . ...........  .................  AACAGGAAGGAGTCCTGATCGACCGACCCCGA     s:0 i:0 d:0
5116628   . ...........  .................  CCGGGCGACGCCGAGGCGGTGTGGAACATCCG     s:0 i:0 d:0
5116689   . ...........  .................  CTTGCCTTCGAGGGCTTCCTCGATCTCCCGGT     s:0 i:0 d:0
5116750   . ...........  .................  GGAGGCGCTGCTGGAGGCCCCGGCAGTCTGG      s:0 i:0 d:0
5116810   .A...........  .................  AACCGTGGCCAGCGCCAGACGGTACACCCAAA     s:0 i:1 d:0
5116872   . ...........  .................  GAGCTCGTGCAGATAGATAGCCCCGCCGGCCT     s:0 i:0 d:0
5116933   . ...........  .................  CTTCGAGGGCTCGGGGGAAGGGTCCTGGGGCGG    s:0 i:0 d:0
5116995   . ...........  .................  GGTGGCGCAGGCGGCCACTCCGGGCCAGTGGC     s:0 i:0 d:0
5117056   . ...........  .................  TTTTTCGCCGGCTTGATCTCATCCTCGAAGTA     s:0 i:0 d:0
5117117   . ...........  .................                                       s:0 i:0 d:0
_____________________________________________________________________________________________
          G GGGTCATCCCC  GCGTGCGCGGGGAGCAG                                       s:4 i:3 d:2
```

Here we demonstrate the ability of our approach to detect IS elements. We list three CRISPR arrays found in NC_006513 which share the same consensus repeat.

```
>gi|56475432|ref|NC_006513.1| Aromatoleum aromaticum EbN1 chromosome, complete genome

CRISPR: 1, 1946881-1941501, number of Repeats: 76, avg. length of Repeat: 37, avg length of Spacer: 35

1946881   .....................................  TGAGGTTCAGACTGCTGACCGAGTCCTGTGCGTG            s:0 i:0 d:0
1946810   .....................................  GATGCTGCCCAGTGAAGAGACAAGCGCGACCGAG            s:0 i:0 d:0
1946739   .....................................  GGCGCCGAATGGTCGCCTACACCGCCGACTGGATC           s:0 i:0 d:0
1946667   .....................................  GGCTCTGCATGTGCGAGGACTGCGCTCCGTCGTTCGA         s:0 i:0 d:0
1946593   .....................................  GCATCTTTGTTACCACTAAGAGCGAGGACAGAAA            s:0 i:0 d:0
1946522   .....................................  TCGATAAGTACGACAAGGCGACGAAGCAGCGCAA            s:0 i:0 d:0
1946451   .....................................  CGCATCAAAACGGCGCTGCTGTCGCGGCTGTCGGAAATC       s:0 i:0 d:0
1946375   .....................................  TCTCGTCCGTGGTGTCGAGCCGGTACAGCAAGAG            s:0 i:0 d:0
1946304   .....................................  GGTGAGGTCCAGGATGTGCCGCACGTTGCGCGGTGC          s:0 i:0 d:0
1946231   .....................................  GGCTCGTCCATGTCCATGCACAGGACGATGTCGGA           s:0 i:0 d:0
1946159   .....................................  GGGCATGAGCGACGCCGAGTGGGATTACCTGTACC           s:0 i:0 d:0
1946087   .....................................  CCCGGACGTACTGGCTCAGCGGCGCACGGTCGAC            s:0 i:0 d:0
1946016   .....................................  CGATTCTCGGCTGCCTCAAGTTGCGCGGGGCTAAG           s:0 i:0 d:0
1945944   .....................................  GCGCACCGATTCCGCGGTCGTCTTGCTGATCGACGC          s:0 i:0 d:0
1945871   .....................................  CAGGCGACGATTGATGCCGCAGACGCAGCCCAACAG          s:0 i:0 d:0
1945798   .....................................  GGTCATCAAGGCGGTGGTGCACCGGCAGATCGAG            s:0 i:0 d:0
1945727   .....................................  TGGACCAAGATTGGCGGGGGCGATTCGGTTCGGCG           s:0 i:0 d:0
1945655   .....................................  CGGAATTGGAGCGATCAGCACCGTAGCGCCCGACA           s:0 i:0 d:0
1945583   .....................................  GATCCAGGAGGCAGTTGAGGTCTGGATAGAGGGC            s:0 i:0 d:0
1945512   .....................................  ACAGTGATCGCCTGCACGTCGACGAAGGTTTTGTT           s:0 i:0 d:0
1945440   .....................................  GTGGCTGGGGCGGATTTTTTGCGGCTGTTGAGAGC           s:0 i:0 d:0
1945368   .....................................  GCTGGCGACGGGTCGGTGGGAAGGGGGATCACAT            s:0 i:0 d:0
1945297   .....................................  TTCAGCTTCTCGGCCTGCTCATAGAGATAGGTGTC           s:0 i:0 d:0
1945225   .....................................  GCGGCGTGGTCGTGGGCGGGAGGGTTCGGTCAGGC           s:0 i:0 d:0
1945153   .....................................  GAAGCCTTTTTGCGGGTCGTGGTTGTACTTGCGCGC          s:0 i:0 d:0
1945080   .....................................  ACGATCGTCGGCCACGACAACCACGGCAACTACAC           s:0 i:0 d:0
1945008   .....................................  GATGGCCCGACGCGTCGGGTGCGGCACGCTGTCGAT          s:0 i:0 d:0
1944935   .....................................  CGCTCGGCGCTCACGCCTCACCCTCGATCGGCGC            s:0 i:0 d:0
1944864   .....................................  GCCGCAATCCGCAACGGTGCGGAGAACGTCACCGA           s:0 i:0 d:0
1944792   .....................................  CGGCGCTGCGCGATGCGTGCTGCGAACTCGCGCT            s:0 i:0 d:0
1944721   .....................................  GCCCCACACTTGCCCGGCGAGCGCGACGGCCGTATC          s:0 i:0 d:0
1944648   .....................................  CGTCACCACGGTCACGCTCGACCCGGTGCTGCGC            s:0 i:0 d:0
1944577   .....................................  CGTTCGACTTGATGACCGACGCCGCTTATCACGGC           s:0 i:0 d:0
1944505   .....................................  CGCACCTGTTCGTTCCAACCCCTCATGACCCACCT           s:0 i:0 d:0
1944433   .....................................  GGGTGAGCGCGTCGGCGATGAAAATCGACTCGGG            s:0 i:0 d:0
1944362   .....................................  CATGGCGAGGCGCGCCGCAGCGGGGTCGATGTCGGG          s:0 i:0 d:0
1944289   .....................................  CTTCGCCGGTGCCTACCAGCGCGACGACACAGGC            s:0 i:0 d:0
1944218   .....................................  CTCGCCAGGGCGCGACCGGCCGCCGTGCTCATGTC           s:0 i:0 d:0
1944146   .....................................  GGCTGCCCCGTGAGCGTGAAAACCTCGATCCATGCC          s:0 i:0 d:0
1944073   .....................................  CCCGAGATCCTCGCCGCGGACCTGATCGTCAGCCA           s:0 i:0 d:0
1944001   .....................................  TCGACTGCCGAGCACCAGGCGCTGAAGACCGCCCACGACAAGCT  s:0 i:0 d:0
1943920   .....................................  ATGCGAGCGCCAGCGCCTTCACGGCCGAAGACGCC           s:0 i:0 d:0
1943848   .....................................  TTCGAGCAGTCCGGACGACACGCACGACCAGACCG           s:0 i:0 d:0
1943776   .....................................  ACGGCGACCCGCTGCGGCCGGACCTCGATTACCT            s:0 i:0 d:0
1943705   .....................................  ATTAGTTGCACCGACGCGAGCGCCGAGTCGACGTA           s:0 i:0 d:0
1943633   .....................................  CCGGCAGATCGCCGAGCACCGCGCCAAGCTCGAA            s:0 i:0 d:0
1943562   .....................................  CACGTGAGTTGTGCGTCGAGTTCGCTTGGCAGGTT           s:0 i:0 d:0
1943490   .....................................  GCGTCGGTCCATCCGGCGTCGGCGAGCACGAGCTT           s:0 i:0 d:0
1943418   .....................................  GTGGGATCGGGCGCTCGACGACCTGCAACTGATG            s:0 i:0 d:0
1943347   .....................................  CTTGGCGCTTACGCCGTGATCGTGATTGCCGTCAT           s:0 i:0 d:0
1943275   .....................................  CGCGAGGCTAAGGCGTATCTCGGCGTGCAGGACGT           s:0 i:0 d:0
1943203   .....................................  CACGGTCACCAATGCCGGCGTTGCCGGGTAACCACT          s:0 i:0 d:0
1943130   .....................................  GCGCCCGGCGAGCTTCGCCGACGCGGCGAACTTC            s:0 i:0 d:0
1943059   .....................................  CGCGGATCAGCTTGATGTAGGCGTCGGTCCATCCC           s:0 i:0 d:0
1942987   .....................................  CCGGACTGCGACGGAAAGGGGCGGACTGAGAAGAT           s:0 i:0 d:0
1942915   .....................................  GGCTGCCTCGCCGCGGTGAAGCAGGCGATCAGGGA           s:0 i:0 d:0
1942843   .....................................  CGGCGGCAGCAGTAAAAAGTCGAGCACGAATACGAC          s:0 i:0 d:0
1942770   .....................................  GTCCTGAACCCGGCTGACTGGGCGACGATGGAAACG          s:0 i:0 d:0
1942697   .....................................  GCGGCAGGCCGGGCGGTGCGCATCGTCGAGCGG             s:0 i:0 d:0
1942627   .....................................  CATGCACACGGTATTCACGGCATCCTCCCTGTCTC           s:0 i:0 d:0
1942555   .....................................  CTGAGTGATGGCTGACAAAGACAAGAACGAATACCTT         s:0 i:0 d:0
1942481   .....................................  CAAGCTGGCGCCCGCCTCTTTGCGCCTCAAGTCGCAAAT       s:0 i:0 d:0
1942405   .....................................  ATCGTTCGAGTTCGTTCTGCGGCGTAGTCATATT            s:0 i:0 d:0
1942334   .....................................  GCTGGAACGCCTACCAGCAGATCAAGGACAACCT            s:0 i:0 d:0
1942263   .....................................  TCAATGCCGGCTGGGACAACCGCGGCGAACAGCTT           s:0 i:0 d:0
1942191   .....................................  CTCACGGATGTCGCGGTAGTCGAACACCGGCAGGGG          s:0 i:0 d:0
1942118   .....................................  ATGTTCCCCGAGGGGCAGGTGGTCAAGTTCGACCTC          s:0 i:0 d:0
1942045   .....................................  TGTTGACGCAGTCCCACTGCACGCCATGCAGCAC            s:0 i:0 d:0
1941974   .....................................  TTCCTGAGCTTCCCTCCCACACGCTTACGCACGTA           s:0 i:0 d:0
1941902   .....................................  CGATCAGTTCGGCGGGGGGTTCATCCGCGAACTAGAGC        s:0 i:0 d:0
1941827   .....................................  GGTTCGGGTGGTTAGGCATCTCAGCGGTTGGCCGAA          s:0 i:0 d:0
1941754   .....................................  CAAGCGCGAGTTTTACCGGAGGTACGGCAGCGAAGCG         s:0 i:0 d:0
1941680   .....................................  CGTCGCAAAAGAGGACGACAACGGGCTGTACGTC            s:0 i:0 d:0
1941609   .....................................  ACCAAGATCAAGACGATCGGCAAGCTGCTGGACGA           s:0 i:0 d:0
1941537   .....................................  GGCTGCGCCTCGTCGGCGAGCAGGGTCCGGAACTG           s:0 i:0 d:0
1941465   ........................T............                                                s:1 i:0 d:0
_______________________________________________________________________________________________
          GCATCGCCCCTCGGTGACGGGGGGCGTGGATTGAAAC                                                s:1 i:0 d:0

Leader region
GCGGAAGCGGCTCATGCTCTCGGGTTCCAGGGGCGACGCGTCATTTTACAGGGGATAGTCCGACACGCTGCTAGGATCAGGCCATCAAGGTTCGACGGGA

Strand: Reverse

#   Array features:
#   repeat_similarity: 0.9967125788897947
#   avg_spacer_len: 35.21333333333333
#   spacer_similarity: 0.3358659016417535
#   number_mismatches: 1.0
#   spacer_evenness: 0.173116
#   mfe_score: -14.4
#   orf_score: 99.53
#   blast_score_1: 28.8
_____________________________
#   Certainty Score: 0.9893240652773534
```

IS Element: IS-clust-6 [1947003-1948361]

```
CRISPR: 2, 1950432-1948537, number of Repeats: 28, avg. length of Repeat: 37, avg length of Spacer: 34

1950432   .....................................  GGACTGCAAGGCGATGCTGCTGCGCGCCGGCATC        s:0 i:0 d:0
1950361   .....................................  TCAGCACGCCGATCGGCATGTCCTCCGTCGGATA        s:0 i:0 d:0
1950290   .....................................  TGGTTTCCCGCCTTCACGTCTGCCAGGAATTTAGA       s:0 i:0 d:0
1950218   .....................................  GTTTTGTGGATGCGCAGCATTGCTTGCTGCCCAT        s:0 i:0 d:0
1950147   .....................................  GTTGAGTTGGGCGCGAGCATTAGGGATTTGTGAT        s:0 i:0 d:0
1950076   .....................................  CAGAGCGCGACGGATGCGACTTCGGGCCGATTACT       s:0 i:0 d:0
1950004   .....................................  CGCCAGCCCGTCGTCGAATACATCGAGGTCGAATGA      s:0 i:0 d:0
1949931   .....................................  AAAGGCCGGCACCTCAACCACCGCGCCCCAGGCA        s:0 i:0 d:0
1949860   .....................................  CAGCGATGCCGCACTTCAGCATGCTGTCGAGCAC        s:0 i:0 d:0
1949789   .....................................  ATAGCAGTCTCTATGCCGACGTACTGGTCAATCT        s:0 i:0 d:0
1949718   .....................................  GGTCGACGAGTTCGGTGTCAGCCATCTCCTGGCT        s:0 i:0 d:0
1949647   .....................................  GCAGCGTGATCAGCCGACACGCTGGGTCGTCAAT        s:0 i:0 d:0
1949576   .....................................  GTCGATTTCTGCCACCTCAACGGGCTCACCGGT         s:0 i:0 d:0
1949506   .....................................  CCGACTACGCCGGAGCGTGGCAAGCGCTCGGAAC        s:0 i:0 d:0
1949435   .....................................  TAGGTCGGGTTGCCGGCGTGACCGATGCGGAACCA       s:0 i:0 d:0
1949363   .....................................  CGCACGATCAATGGCGTGAGCTTCAACGGCACGGC       s:0 i:0 d:0
1949291   .....................................  ACTCTCCGGCACGGCGATCGATTGGGCCGCCAACGC      s:0 i:0 d:0
1949218   .....................................  GGCGAACGTTTCCGCCTGCAGGGTCATATCGGAG        s:0 i:0 d:0
1949147   .....................................  GATCGAGTGCGTGTTCCGCTACCGCACGCGGACT        s:0 i:0 d:0
1949076   .....................................  GAACCCGGTCACGGGGACGATCCGCTGGTCATCGTC      s:0 i:0 d:0
1949003   .....................................  GGCGTCATCGCCTTGCTCGGTAGCGAACAAAAGC        s:0 i:0 d:0
1948932   .....................................  CCGCCCCCACTGCCGCACCGCCTCGACGAATTCGCC      s:0 i:0 d:0
1948859   .....................................  TCCCCACGATCTTGCCCATGCACTCGTTCTGGCT        s:0 i:0 d:0
1948788   .....................................  GAGGAGAGATCGCCGGCTGCATCCCCATCAACGA        s:0 i:0 d:0
1948717   .....................................  CCTCCTGGTGCGGGAGCAGGTCAGTTGTCGTCGC        s:0 i:0 d:0
1948646   .....................................  ATGATGTTGTCCTTGTTCATGGTCGGCGACTTATT       s:0 i:0 d:0
1948574   .....................................  GGATATGACCTTCGGCTCGATGGTCGGATTCATGGT      s:0 i:0 d:0
1948501   .....................................                                            s:0 i:0 d:0
______________________________________________________________________________________________
          GCATCGCCCCTCGGTGACGGGGGGCGTGGATTGAAAC                                            s:0 i:0 d:0

Leader region
ATCTTCTCAACTCGGTGTATCAACTTTCAATCGGCAGCCCGTATCAACTTACATCCGGCAGTGACAATCCAGAAGTGCGGCCTCACGGCGCCAGTTCCTG

Strand: Reverse

#   Array features:
#   repeat_similarity: 1.0
#   avg_spacer_len: 34.51851851851852
#   spacer_similarity: 0.34150264423613624
#   number_mismatches: 0.0
#   spacer_evenness: -0.0
#   mfe_score: -14.4
#   orf_score: 99.12
#   blast_score_1: 28.8
______________________________
#   Certainty Score: 0.9893240652773534
```

IS Element: IS-clust-85 [1950588-1951676]

```
CRISPR: 3, 1953539-1952640, number of Repeats: 14, avg. length of Repeat: 37, avg length of Spacer: 34

1953539   .....................................  GCGGCGTTGTTGCCGATCGTCTTCGTTACGTTCTG        s:0 i:0 d:0
1953467   .....................................  TTCGGCGTGGCATGGCAACTAAAGCGCGTACTGG         s:0 i:0 d:0
1953396   .....................................  GGATGGGATCGATATTGGTGCAGGGTGGCCTGCGT        s:0 i:0 d:0
1953324   .....................................  GATTGAGTCGGCGGGGTGGATAAGCCACACTAACTA       s:0 i:0 d:0
1953251   .....................................  TCTTGCAGATGTGCACGGTCGAGGTCGAACGTGTC        s:0 i:0 d:0
1953179   .....................................  CTCGGCGCGGCCGGCGGCTACTACAAGCACCTGAT        s:0 i:0 d:0
1953107   .....................................  GCAATCCGATCGTCGATCGGGCCACGGGAGAGGT         s:0 i:0 d:0
1953036   .....................................  CAACCACTGGCACCGACGGAGGGCCGCACAAGAT         s:0 i:0 d:0
1952965   .....................................  AACGACTTGTTCGCGGCGATCCGCTGCGTCTCGAT        s:0 i:0 d:0
1952893   .....................................  AACGCGACCTTGAACAGCGCGAAGCCCTTGCGATG        s:0 i:0 d:0
1952821   .....................................  GTCGGCGACTGCCTGGCCATCACCATGCAGGCGAT        s:0 i:0 d:0
1952749   .....................................  CGCATCTCGTGCCCGCAGAGCGTGCAGCGGAGCA         s:0 i:0 d:0
1952678   .....................................  CAGCGAGGGCGGGCTGGTGGTGTTTGCGGAAACCGAG      s:0 i:0 d:0
1952604   .....................................                                             s:0 i:0 d:0
______________________________________________________________________________________________
          GCATCGCCCCTCGGTGACGGGGGGCGTGGATTGAAAC                                             s:0 i:0 d:0

Leader region
CGGATTGTCCAAATGAAATCACCCCGACGAACTTCTTCGCACAGAGGTTCTCGTAACACGTTCTTAATTCGTCGGCGGATCAATACATTACGTGCGAAGA

Strand: Reverse

#   Array features:
#   repeat_similarity: 1.0
#   avg_spacer_len: 34.92307692307692
#   spacer_similarity: 0.33727097886428414
#   number_mismatches: 0.0
#   spacer_evenness: 0.151354
#   mfe_score: -14.4
#   orf_score: 0.0
#   blast_score_1: 28.8
______________________________
#   Certainty Score: 0.9799339653212158
```

We show that corresponding results can be merged into one CRISPR array

```
1941465    ............A.......................    GGCTGCGCCTCGTCGGCGAGCAGGGTCCGGAACTG
1941537    ....................................    ACCAAGATCAAGACGATCGGCAAGCTGCTGGACGA
1941609    ....................................    CGTCGCAAAAGAGGACGACAACGGGCTGTACGTC
1941680    ....................................    CAAGCGCGAGTTTTACCGGAGGTACGGCAGCGAAGCG
1941754    ....................................    GGTTCGGGTGGTTAGGCATCTCAGCGGTTGGCCGAA
1941827    ....................................    CGATCAGTTCGGCGGGGGGTTCATCCGCGAACTAGAGC
1941902    ....................................    TTCCTGAGCTTCCCTCCCACACGCTTACGCACGTA
1941974    ....................................    TGTTGACGCAGTCCCACTGCACGCCATGCAGCAC
1942045    ....................................    ATGTTCCCCGAGGGGCAGGTGGTCAAGTTCGACCTC
1942118    ....................................    CTCACGGATGTCGCGGTAGTCGAACACCGGCAGGGG
1942191    ....................................    TCAATGCCGGCTGGGACAACCGCGGCGAACAGCTT
1942263    ....................................    GCTGGAACGCCTACCAGCAGATCAAGGACAACCT
1942334    ....................................    ATCGTTCGAGTTCGTTCTGCGGCGTAGTCATATT
1942405    ....................................    CAAGCTGGCGCCCGCCTCTTTGCGCCTCAAGTCGCAAAT
1942481    ....................................    CTGAGTGATGGCTGACAAAGACAAGAACGAATACCTT
1942555    ....................................    CATGCACACGGTATTCACGGCATCCTCCCTGTCTC
1942627    ....................................    GCGGCAGGCCGGGCGGTGCGCATCGTCGAGCGG
1942697    ....................................    GTCCTGAACCCGGCTGACTGGGCGACGATGGAAACG
1942770    ....................................    CGGCGGCAGCAGTAAAAAGTCGAGCACGAATACGAC
1942843    ....................................    GGCTGCCTCGCCGCGGTGAAGCAGGCGATCAGGGA
1942915    ....................................    CCGGACTGCGACGGAAAGGGGCGGACTGAGAAGAT
1942987    ....................................    CGCGGATCAGCTTGATGTAGGCGTCGGTCCATCCC
1943059    ....................................    GCGCCCGGCGAGCTTCGCCGACGCGGCGAACTTC
1943130    ....................................    CACGGTCACCAATGCCGGCGTTGCCGGGTAACCACT
1943203    ....................................    CGCGAGGCTAAGGCGTATCTCGGCGTGCAGGACGT
1943275    ....................................    CTTGGCGCTTACGCCGTGATCGTGATTGCCGTCAT
1943347    ....................................    GTGGGATCGGGCGCTCGACGACCTGCAACTGATG
1943418    ....................................    GCGTCGGTCCATCCGGCGTCGGCGAGCACGAGCTT
1943490    ....................................    CACGTGAGTTGTGCGTCGAGTTCGCTTGGCAGGTT
1943562    ....................................    CCGGCAGATCGCCGAGCACCGCGCCAAGCTCGAA
1943633    ....................................    ATTAGTTGCACCGACGCGAGCGCCGAGTCGACGTA
1943705    ....................................    ACGGCGACCCGCTGCGGCCGGACCTCGATTACCT
1943776    ....................................    TTCGAGCAGTCCGGACGACACGCACGACCAGACCG
1943848    ....................................    ATGCGAGCGCCAGCGCCTTCACGGCCGAAGACGCC
1943920    ....................................    TCGACTGCCGAGCACCAGGCGCTGAAGACCGCCCACGACAAGCT
1944001    ....................................    CCCGAGATCCTCGCCGCGGACCTGATCGTCAGCCA
1944073    ....................................    GGCTGCCCCGTGAGCGTGAAAACCTCGATCCATGCC
1944146    ....................................    CTCGCCAGGGCGCGACCGGCCGCCGTGCTCATGTC
1944218    ....................................    CTTCGCCGGTGCCTACCAGCGCGACGACACAGGC
1944289    ....................................    CATGGCGAGGCGCGCCGCAGCGGGGTCGATGTCGGG
1944362    ....................................    GGGTGAGCGCGTCGGCGATGAAAATCGACTCGGG
1944433    ....................................    CGCACCTGTTCGTTCCAACCCCTCATGACCCACCT
1944505    ....................................    CGTTCGACTTGATGACCGACGCCGCTTATCACGGC
1944577    ....................................    CGTCACCACGGTCACGCTCGACCCGGTGCTGCGC
1944648    ....................................    GCCCCACACTTGCCCGGCGAGCGCGACGGCCGTATC
1944721    ....................................    CGGCGCTGCGCGATGCGTGCTGCGAACTCGCGCT
1944792    ....................................    GCCGCAATCCGCAACGGTGCGGAGAACGTCACCGA
1944864    ....................................    CGCTCGGCGCTCACGCCTCACCCTCGATCGGCGC
1944935    ....................................    GATGGCCCGACGCGTCGGGTGCGGCACGCTGTCGAT
1945008    ....................................    ACGATCGTCGGCCACGACAACCACGGCAACTACAC
1945080    ....................................    GAAGCCTTTTTGCGGGTCGTGGTTGTACTTGCGCGC
1945153    ....................................    GCGGCGTGGTCGTGGGCGGGAGGGTTCGGTCAGGC
1945225    ....................................    TTCAGCTTCTCGGCCTGCTCATAGAGATAGGTGTC
1945297    ....................................    GCTGGCGACGGGTCGGTGGGAAGGGGGATCACAT
1945368    ....................................    GTGGCTGGGGCGGATTTTTTGCGGCTGTTGAGAGC
1945440    ....................................    ACAGTGATCGCCTGCACGTCGACGAAGGTTTTGTT
1945512    ....................................    GATCCAGGAGGCAGTTGAGGTCTGGATAGAGGGC
1945583    ....................................    CGGAATTGGAGCGATCAGCACCGTAGCGCCCGACA
1945655    ....................................    TGGACCAAGATTGGCGGGGGCGATTCGGTTCGGCG
1945727    ....................................    GGTCATCAAGGCGGTGGTGCACCGGCAGATCGAG
1945798    ....................................    CAGGCGACGATTGATGCCGCAGACGCAGCCCAACAG
1945871    ....................................    GCGCACCGATTCCGCGGTCGTCTTGCTGATCGACGC
1945944    ....................................    CGATTCTCGGCTGCCTCAAGTTGCGCGGGGCTAAG
1946016    ....................................    CCCGGACGTACTGGCTCAGCGGCGCACGGTCGAC
1946087    ....................................    GGGCATGAGCGACGCCGAGTGGGATTACCTGTACC
1946159    ....................................    GGCTCGTCCATGTCCATGCACAGGACGATGTCGGA
1946231    ....................................    GGTGAGGTCCAGGATGTGCCGCACGTTGCGCGGTGC
1946304    ....................................    TCTCGTCCGTGGTGTCGAGCCGGTACAGCAAGAG
1946375    ....................................    CGCATCAAAACGGCGCTGCTGTCGCGGCTGTCGGAAATC
1946451    ....................................    TCGATAAGTACGACAAGGCGACGAAGCAGCGCAA
1946522    ....................................    GCATCTTTGTTACCACTAAGAGCGAGGACAGAAA
1946593    ....................................    GGCTCTGCATGTGCGAGGACTGCGCTCCGTCGTTCGA
1946667    ....................................    GGCGCCGAATGGTCGCCTACACCGCCGACTGGATC
1946739    ....................................    GATGCTGCCCAGTGAAGAGACAAGCGCGACCGAG
1946810    ....................................    TGAGGTTCAGACTGCTGACCGAGTCCTGTGCGTG
1946881    ....................................    TCCCGTCGAACCTTGATGGCCTGATCCTAGCAGCGTGTCGGACTATCCCCTGTAAAATGACGCGTCGCCCCTGGAACCCGAGAGCATGAGCCGCTTCCGCCC ...
1948501    ....................................    GGATATGACCTTCGGCTCGATGGTCGGATTCATGGT
1948574    ....................................    ATGATGTTGTCCTTGTTCATGGTCGGCGACTTATT
1948646    ....................................    CCTCCTGGTGCGGGAGCAGGTCAGTTGTCGTCGC
1948717    ....................................    GAGGAGAGATCGCCGGCTGCATCCCCATCAACGA
1948788    ....................................    TCCCCACGATCTTGCCCATGCACTCGTTCTGGCT
1948859    ....................................    CCGCCCCCACTGCCGCACCGCCTCGACGAATTCGCC
1948932    ....................................    GGCGTCATCGCCTTGCTCGGTAGCGAACAAAAGC
1949003    ....................................    GAACCCGGTCACGGGGACGATCCGCTGGTCATCGTC
1949076    ....................................    GATCGAGTGCGTGTTCCGCTACCGCACGCGGACT
1949147    ....................................    GGCGAACGTTTCCGCCTGCAGGGTCATATCGGAG
1949218    ....................................    ACTCTCCGGCACGGCGATCGATTGGGCCGCCAACGC
1949291    ....................................    CGCACGATCAATGGCGTGAGCTTCAACGGCACGGC
1949363    ....................................    TAGGTCGGGTTGCCGGCGTGACCGATGCGGAACCA
1949435    ....................................    CCGACTACGCCGGAGCGTGGCAAGCGCTCGGAAC
1949506    ....................................    GTCGATTTCTGCCACCTCAACGGGCTCACCGGT
1949576    ....................................    GCAGCGTGATCAGCCGACACGCTGGGTCGTCAAT
1949647    ....................................    GGTCGACGAGTTCGGTGTCAGCCATCTCCTGGCT
1949718    ....................................    ATAGCAGTCTCTATGCCGACGTACTGGTCAATCT
1949789    ....................................    CAGCGATGCCGCACTTCAGCATGCTGTCGAGCAC
1949860    ....................................    AAAGGCCGGCACCTCAACCACCGCGCCCCAGGCA
1949931    ....................................    CGCCAGCCCGTCGTCGAATACATCGAGGTCGAATGA
1950004    ....................................    CAGAGCGCGACGGATGCGACTTCGGGCCGATTACT
1950076    ....................................    GTTGAGTTGGGCGCGAGCATTAGGGATTTGTGAT
1950147    ....................................    GTTTTGTGGATGCGCAGCATTGCTTGCTGCCCAT
1950218    ....................................    TGGTTTCCCGCCTTCACGTCTGCCAGGAATTTAGA
1950290    ....................................    TCAGCACGCCGATCGGCATGTCCTCCGTCGGATA
1950361    ....................................    GGACTGCAAGGCGATGCTGCTGCGCGCCGGCATC
1950432    ....................................    CAGGAACTGGCGCCGTGAGGCCGCACTTCTGGATTGTCACTGCCGGATGTAAGTTGATACGGGCTGCCGATTGAAAGTTGATACACCGAGTTGAGAAGAT ...
1952604    ....................................    CAGCGAGGGCGGGCTGGTGGTGTTTGCGGAAACCGAG
1952678    ....................................    CGCATCTCGTGCCCGCAGAGCGTGCAGCGGAGCA
1952749    ....................................    GTCGGCGACTGCCTGGCCATCACCATGCAGGCGAT
1952821    ....................................    AACGCGACCTTGAACAGCGCGAAGCCCTTGCGATG
1952893    ....................................    AACGACTTGTTCGCGGCGATCCGCTGCGTCTCGAT
1952965    ....................................    CAACCACTGGCACCGACGGAGGGCCGCACAAGAT
1953036    ....................................    GCAATCCGATCGTCGATCGGGCCACGGGAGAGGT
1953107    ....................................    CTCGGCGCGGCCGGCGGCTACTACAAGCACCTGAT
1953179    ....................................    TCTTGCAGATGTGCACGGTCGAGGTCGAACGTGTC
1953251    ....................................    GATTGAGTCGGCGGGGTGGATAAGCCACACTAACTA
1953324    ....................................    GGATGGGATCGATATTGGTGCAGGGTGGCCTGCGT
1953396    ....................................    TTCGGCGTGGCATGGCAACTAAAGCGCGTACTGG
1953467    ....................................    GCGGCGTTGTTGCCGATCGTCTTCGTTACGTTCTG
1953539    ....................................
__________________________________________________________________________________
           GTTTCAATCCACGCCCCCGTCACCGAGGGGCGATGC
```

9 Spurious Arrays in Existing Tools

The following figures show examples of spurious arrays found by the different tools in the CRISPRCasFinder data set.

Mismatches are marked in red. Orange and yellow marks show different patches on identical subsequences.

```
NC_010805-2079941-2081421

CRISPR 2   Range: 991 - 1490
POSITION      REPEAT                       SPACER
--------      --------------------         ---------------------------------
991           TCGACGGGCCTCAGCTCGAC         CGACAGCTCGGTCGCTTCGCTGTCGACCTCGACT [ 20, 34 ]
1045          TCGACCGGCCTCTCGTCGGC         GAATAGCTCGATCACGTCCTTGTCGACTTCGACC [ 20, 34 ]
1099          TCGACCGGCCTCTCTTCGGC         TAACAGCTCGATCGGTTCTCTC       [ 20, 22 ]
1141          TCGACCGGGCTCTCGTCGGC         AGACAGCTCGATCACGTCGCTCTCGACCTCGACT [ 20, 34 ]
1195          TCGACCGGCCTCTCCTCGGC         CACTAGCTCGATCGGCTCCCTG       [ 20, 22 ]
1237          TCGACCGGCCTCTCCTCGGC         TAACAGCTCGATCGGCTCCCTG       [ 20, 22 ]
1279          TCGACCGGCCTCAGCTCGAC         CAACAGCTCGGTCGCTTCGCTG       [ 20, 22 ]
1321          TCGACCGGCCTCAGCTCGAC         CGATAGCTCGCTCGCTTCGCTGTCGACCTCGACCTCGTCCGGCCTC   [ 20, 46 ]
1387          TCGTCCGCCACCAGCTCGAT         CGGCTCGCTGTCCACCGCCCTGAGCTCGACCGACAGCACGCTCACGTCGCTGTCCACGTCGACC     [ 20, 64 ]
1471          TCGTCGGGCATCGGCTCGCT
--------      -------------------- ---------------------------------
Repeats: 10   Average Length: 20         Average Length: 33

NZ_CP007739-2469188-2469435

CRISPR 1   Range: 9 - 122
POSITION      REPEAT                       SPACER
--------      ------------------------     ---------------------
9             AAATTCTTGTATCGGTGAAACGGG     CATTTGCTTTTCCACTTTAGG      [ 24, 21 ]
54            AAATTCTTGTATCGGTGAAACGGG     CATTTGCTTTTCTACTTTAGG      [ 24, 21 ]
99            AAACTCCTGTATCGGTGAAACGGG
--------      ------------------------     ---------------------
Repeats: 3    Average Length: 24           Average Length: 21


NZ_CP012727-1182797-1183497


CRISPR 1   Range: 38 - 717
POSITION      REPEAT                       SPACER
--------      -----------------------      ------------------------------------
38            CCAGTCGGTCCAGTCGGTCCAGT      CAGTCCGGTAGCACCAGTTGGCCCAGTTGGTCCAGTTGGCCCAGTTGGT      [ 23, 49 ]
110           CCAGTTGGTCCAGTTGGCCCAGT      TGGTCCAGTAGTACCAGTGTCTCCAGTTGGCCCAGTTGGT [ 23, 40 ]
173           CCAGTTGGTCCAGTTGGCCCAGT      TGGTCCAGTAGTACCAGTGTCTCCAGTTGGC    [ 23, 31 ]
227           CCAGTTGGTCCAGTTGGCCCAGT      CGGCCCAGTTGGTCCAGTTGGCCCAGTCGGCCCAGTCGGTCCAGTCGGCCCAGTTGGT    [ 23, 58 ]
308           CCAGTTGGTCCAGTTGGCCCAGT      TGGTCCAGTAGTACCAGTGTCTCCAGTTGGCCCAGTTGGT [ 23, 40 ]
371           CCAGTTGGTCCAGTTGGCCCAGT      TGGTCCAGTAGTACCAGTGTCTCCAGTTGGC    [ 23, 31 ]
425           CCAGTTGGTCCAGTTGGCCCAGT      CGGCCCAGTTGGTCCAGTTGGCCCAGTCGGC    [ 23, 31 ]
479           CCAGTTGGTCCAGTTGGCCCAGT      TGGTCCAGTAGTACCAGTGTCTCCAGTTGGC    [ 23, 31 ]
533           CCAGTTGGTCCAGTTGGCCCAGT      CGGCCCAGTTGGTCCAGTAGTACCAGTGTCTCCAGTTGGC [ 23, 40 ]
596           CCAGTTGGTCCAGTTGGCCCAGT      CGGCCCAGTTGGTCCAGTTGGCCCAGTCGGC    [ 23, 31 ]
650           CCAGTTGGTCCAGTTGGCCCAGT      CGGCCCAGTCGGTCCAGTCGGC     [ 23, 22 ]
695           CCAGTTGGTCCGGTAGGAAGGGT
--------      -----------------------      ------------------------------------
Repeats: 12   Average Length: 23           Average Length: 36

NZ_CP017107-1181346-1185268

CRISPR 1   Range: 39 - 3934
POSITION      REPEAT                       SPACER
--------      --------------------         ------------------------------------------
39            CCAGGCAAAGAGCCGGAAAC         TCCAGTGGGCCCAAGTGAA [ 20, 19 ]
78            CCAGGCAAAGAGCCAGAAAC         TCCAGTGGATCCAAGTGAA [ 20, 19 ]
117           CCAGGCAAAGAGCCAGAAAC         ACCAGTGGATCCAAGTGAA [ 20, 19 ]
156           CCAGGCAAAGAGCCGGAAAC         ACCAGTGGATCCAAGTGAA [ 20, 19 ]
195           CCAGGCAAAGAGCCAGAAAC         TCCAGTGGATCCAAGTGAA [ 20, 19 ]
234           CCAGGCAAAGAACCGGAAAC         ACCAGTGGCCCCAAGTGAG [ 20, 19 ]
273           CCAGGCAAAGAGCCGGAAAC         ACCAGTGGACCCAAGTGAA [ 20, 19 ]
312           CCGGGCAAAGAACCGGAAAC         TCCAGTGAACCCAAGTGAG [ 20, 19 ]
351           CCAGGCAAAGAGCCGGAAAC         ACCAGTGGATCCAAGTGAG [ 20, 19 ]
390           CCAGGCAAAGAACCGGAAAC         CCCAGTGGGCCCAAGTGAG [ 20, 19 ]
429           CCAGGCAAAGAGCCGGAAAC         ACCAGTGGATCCAAGTGAA [ 20, 19 ]
468           CCAGGTAAAGAGCCAGAAAC         TCCAGTAGATCCAAGTGAA [ 20, 19 ]
507           CCAGGCAAAGAACCGGAAAC         TCCAGTGGATCCAAGTGAG [ 20, 19 ]
546           CCAGGCAAAGAGCCAGAAAC         TCCAGTGGATCCAAGTGAA [ 20, 19 ]
585           TCGGGCAAAGAACCAGAAAC         ACCAGTGGATCCAAGTGAG [ 20, 19 ]
624           CCAGGAAAAGAACCGGAAAA         CCCAGTGGATCCAAGTGAG [ 20, 19 ]
663           CCAGGAAAAGAACCGGAAAC         TCCAGTGGGCCCAAGTGAA [ 20, 19 ]
702           CCGGGCAAAGAACCGGAAAC         TCCAGTGAACCCAAGTGAG [ 20, 19 ]
741           CCAGGAAAAGAGCCGGAAAC         ACCAGTGGATCCAAGTGAA [ 20, 19 ]
780           CCAGGAAAAGAACCGGAAAC         TCCAGTGGACCCAAGTGAA [ 20, 19 ]
819           CCGGGCAAAGAGCCGGAAAC         ACCAGTGGATCCAAGTGAA [ 20, 19 ]
858           CCAGGAAAAGAACCGGAAAC         GCCAGTAGATCCAAGTGAA [ 20, 19 ]
897           CCGGGCAAAGAGCCGGAAAC         ACCAGTGGATCCAAGTGAA [ 20, 19 ]
936           TCAGGTAAAGAGCCCGAAAC         CCCAGTAGATCCAAGTGAA [ 20, 19 ]
975           CCAGGCAAAGAGCCAGAAAC         CCCAGTGGACCCAAGTGAACCGGAAACCCCAGTAGACCCAAGTGAG  [ 20, 46 ]
1041          CCAGGCAAAGAGCCGGAAAT         TCCAGTGGGCCCAAGTAAACCGGAAACCCCAGTAGACCCAAGTGAG  [ 20, 46 ]
1107          CCAGGCAAAGAGCCAGAAAC         CCCAGTGGATCCAAGTGAACCGGGAAAAGAACCGGAAACACCAGTAGATCCAAGTGAA   [ 20, 58 ]
1185          CCAGGAAAAGAACCGGAAAC         ACCAGTGGATCCAAGCAAACCGGGTAAAGAGCCGGAAACTCCAGTGGATCCAAGTGAA   [ 20, 58 ]
1263          CCGGGCAAAGAACCAGAAAC         CCCAGTGGATCCAAGCGAACCGGGTAAAGAGCCGGAAATTCCAGTGGATCCAAGTGAA   [ 20, 58 ]
1341          CCGGGCAAAGAACCGGAAAC         ACCAGTAGATCCAAGTGAATCAGGTAAAGAGCCAGAAACCCTAGTGGATCCAAGTGAA   [ 20, 58 ]
1419          CCAGGAAAAGAACCGGAAAC         TCCAGTGGACCCAAGTGAACCGGGCAAAGAGCCGGAAACACCAGTGGATCCAAGCGAA   [ 20, 58 ]
1497          CCGGGTAAAGAGCTGGAAAT         TACAGTGGATCCAAGTGAACCAGGAAAAGAACCGGAAACTCCAGTGGGCCCAAGTGAA   [ 20, 58 ]
1575          CCGGGAAAAGAACCGGAAAC         ACCAGTGAACCCAAGTGAGCCAGGCAAAGAGCCAGAAACCCCAGTGGATCCAAGCAAA   [ 20, 58 ]
1653          CCGGGAAAAGAACCAGAAAC         ACCAGTAGATCCAAGTGAACCAGGAAAAGAACCAGAAACCCCAGTGGATCCAAGCGAA   [ 20, 58 ]
1731          CCGGGTAAAGAGCTGGAAAT         TACAGTGGATCCAAGTGAACCGGGCAAAGAACCGGAAACACCAGTAGATCCAAGTGAA   [ 20, 58 ]
1809          TCAGGTAAAGAGCCCGAAAC         CCCAGTGGATCCAAGTGAACCAGGCAAAGAACCGGAAACTCCAGTGGGCCCAAGTGAG   [ 20, 58 ]
1887          CCAGGAAAAGAGCCCGAAAC         ACCAGTAGATCCAAGTGAACCAGGAAAAGAACCGGAAACTCCAGTGGGCCCAAGTGAG   [ 20, 58 ]
1965          CCAGGAAAAGAGCCCGAAAC         ACCAGTAGATCCAAGTGAACCGGGAAAAGAACCAGAAACTCCAGTGGATCCAAGTGAA   [ 20, 58 ]
2043          TCAGGCAAAGAACCAGAAAC         CCCAGTGGATCCAAGCGAACCGGGTAAAGAGCCGGAAACTCCAGTGGATCCAAGTGAG   [ 20, 58 ]
2121          CCAGGAAAAGAACCGGAAAC         TCCAGTGGCCCCAAGTGAACCAGTTAAAGAGCCCGAAACCCCAGTGGATCCAAGTGAG   [ 20, 58 ]
2199          CCAGGCAAAGAGCCGGAAAC         TCCAGTGGATCCAAGTGAACCAGTTAAAGAGCCCGAAACCCCAGTGAATCCAAGTGAA   [ 20, 58 ]
2277          CCAGGCAAAGAACCGGAAAC         TCCAGTGGACCCAAGTGAGCCAGGCAAAGAGCCCGAAACACCAGTGGATCCAAGTGAA   [ 20, 58 ]
2355          CCAGGCAAAGAACCGGAAAC         TCCAGTGAATCCAAGTGAACCAGGTAAAGAGCCCGAAACACCAATAGATCCAAGTGAA   [ 20, 58 ]
2433          CCAGGTAAAGAACCGGAAAC         ACCAGTAGATCCAAGTGAGCCAGGAAAAGAACCGGAAACACCAGTGGATCCAAGCGAA   [ 20, 58 ]
2511          CCGGGAAAAGAACCGGAAAC         ACCAGTAGATCCAAGTGAGCCAGGAAAAGAACCGGAAACTCCAGTGGATCCAAGCGAA   [ 20, 58 ]
2589          CCGGGCAAAGAACCAGAAAC         CCCAGTAGATCCAAGTGAACCAGGCAAAGAACCGGAAACCCCAGTGGATCCAAGTGAA   [ 20, 58 ]
2667          CCAGGTAAAGAGCCCGAAAC         CCCAGTGGATCCAAGTGAACCAGGAAAAGAACCGGAAACTCCAGTGAATCCAAGTGAG   [ 20, 58 ]
2745          CCAGGAAAAGAACCAGAAAC         CCCAGTGGATCCAAGTGAGCCAGGAAAAGAACCAGAAACCCCAGTGGATTCAAGTGAG   [ 20, 58 ]
2823          CCAGGAAAAGAACCGGAAAC         TCCAGTGGGCCCAAGTGAACCGGGCAAAGAACCGGAAACACCAGTGAACCCAAGTGAG   [ 20, 58 ]
2901          CCAGGCAAAGAGCCCGAAAC         CCCAGTGGATCCAAGTGAACCGGGAAAAGAACCGGAAACACCAGTAGATCCAAGTGAA   [ 20, 58 ]
2979          CCAGGAAAAGAACCGGAAAC         ACCAGTGGATCCAAGCGAACCGGGTAAAGGGCCGGAAACTCCAGTGGATCCAAGTGAA   [ 20, 58 ]
3057          CCGGGAAAAGAACCAGAAAC         CCCAGTGAATCCAAGTGAGCCAGGAAAAGAACCAGAAACCCCAGTGGATTCAAGTGAG   [ 20, 58 ]
3135          CCAGGAAAAGAACCGGAAAC         TCCAGTGGGCCCAAGTGAACCGGGCAAAGAACCGGAAACACCAGTGAACCCAAGTGAG   [ 20, 58 ]
3213          CCAGGCAAAGAGCCCGAAAC         CCCAGTGGATCCAAGTGAACCGGGAAAAGAACCGGAAACACCAGTAGATCCAAGTGAA   [ 20, 58 ]
3291          CCAGGAAAAGAACCGGAAAC         CCCAGTGGATCCAAGCGAACCGGGAAAAGGGCCGGAAACTCCAGTGGATCCAAGTGAA   [ 20, 58 ]
3369          CCGGGAAAAGAACCAGAAAC         CCCAGTGAATCCAAGTGAGCCAGGAAAAGAACCAGAAACCCCAGTGGATCCAAGTGAG   [ 20, 58 ]
3447          CCAGGAAAAGAACCAGAAAC         CCCAGTGGATCCAAGTGAGCCAGGAAAAGAACCGGAAACTCCAGTGGGCCCAAGTGAA   [ 20, 58 ]
3525          CCAGGAAAAGAACCAGAAAC         ACCAGTAGATCCAAGTGAACCGGGTAAAGAGCCAGAAACCCCAGTGGATTCAAGTGAG   [ 20, 58 ]
3603          CCAGGAAAAGAACCGGAAAC         TCCAGTGAGCCCAAGTGAACCGGGAAAAGAACCGGAAACTCCAGTGAACCCAAGTGAG   [ 20, 58 ]
3681          CCAGGAAAAGAACCAGAAAC         CCCAGTGGATCCAAGCGAACCGGGAAAAGAACCGGAAACTCCAGTGGATCCAAGTGAA   [ 20, 58 ]
3759          TCAGGCAAAGAGCCAGAAAC         CCCAGTGGATCCAAGCGAACCGGGTAAAGAGCCAGAAACCCCAGTGGATTCAAGTGAG   [ 20, 58 ]
3837          CCAGGAAAAGAGCCGGAAAC         CCCAGTGGATTCAAGTGAGCTAGGCAAAGAGCCGGAAACACCAGTAGATCCAAGTGAG   [ 20, 58 ]
3915          CCAGGAAAAGAACCAGAAAC
--------      -------------------- ----------------------------------------
Repeats: 63   Average Length: 20           Average Length: 42
```

Figure S3: Suspicious results of CRT: The first array demonstrates a very low repeat sequences conservation level. On top of that spacers show high level of similarity and have relatively big identical chunks. In the second example presented spacer sequences are almost identical and with only one nucleotide difference. In the third example the spacer sequences not only demonstrate high similarity level but also share a big

chunk with almost all repeat sequences of the array. In the last example the distribution of the length of the spacer sequences showed abnormal change from 19 nucleotides in the first 24 spacers to 58 among the last 36 spacers in the array. Such visual inspection allows us to claim that the presented cases highly unlikely work as CRISPR arrays

NC_019382-4340905-4341224

```
Position      Repeat          %id       Spacer        Repeat_Sequence              Spacer_Sequence                       Insertion/Deletion
==========    ======       ======       ======        ==========================   ==================================    ==================
        10        26        100.0           19        ..........................   GGCTTTCTTGGCTACAGCC
        55        26         92.3           34        ........A..T..............   GGCTTTCTTCGCGACGGCTTTCTTGGCAGGCGCC
       115        26        100.0            4        ..........................   CGCT                                  Deletion [145]
       145        26         96.2           19        ........C.................   GGCTTTCTTGGCGACAGCT
       190        26         92.3           31        .....C..C.................   AGCCTTCTTGGCGACTTTCTTGGCCGCGGGT
       247        26        100.0            0        ..........................   |
==========    ======       ======       ======        ==========================   ==================================    ==================
         6        26         96.8           22        TTCTTGGCGACGGCCTTCTTGGCTAC
```

NZ_CP009605-2741998-2742959

```
  Position    Repeat          %id       Spacer        Repeat_Sequence                     Spacer_Sequence
              Insertion/Deletion
==========    ======       ======       ======        ================================    =====================================================================================
              ==================
       301        32         96.9           67        ..G.............................    TGCTCAAGGTGCTCAAGGACCAGCGGGTGCTACTGGACCTCAAGGACCTCAAGGTGTTCAAGGGCCA
       400        32         90.6           45        .......................AC.T.....    TGTTCAAGGACCAGCAGGTGCTACTGGCGCTACTGGTGCGCAAGG
       443        32        100.0           45        ................................    GGTGCTCAAGGACCAGCGGGTGCTACTGGCGCTACTGGTGCGCAA
       488        32         96.9           49        ..G.............................    GGTGCTCAAGGGCCAGCGGGTGCTACTGGACCTCAAGGTATTCAAGGAC
       571        32        100.0           85        ................................    ACCGGCGGGTGCTACTGGACCTCAAGGGCCTCAAGGTGCTCAAGGGCCAGCGGGTGCTACTGGACCTCAAGGTATTCAAGGACCG
       688        32        100.0            0        ................................    |
==========    ======       ======       ======        ================================    =====================================================================================
              ==================
         6        32         97.4           58        GCAGGTGCTACTGGCGCTACTGGTGCGCAAGG
```

NC_021485-2005949-2006205

```
 Position     Repeat          %id       Spacer        Repeat_Sequence            Spacer_Sequence                                       Insertion/Deletion
==========    ======       ======       ======        =======================    ==================================================    ==================
        67        23        100.0            1        .......................    C                                                     Deletion [91]
        91        23        100.0           49        .......................    CTCTGGTTTGGCTTCTGGTTTGGCCTCTGGTTTGGCTTCTGGTTTGGCC
       163        23         91.3           37        ........GG.............    TTCTGGCTTAACGTCTGGCTTAACATCTGGTTTGGCT
       223        23        100.0            0        .......................    |
==========    ======       ======       ======        =======================    ==================================================    ==================
         4        23         97.8           29        TCTGGTTTAACTTCTGGCTTAGC
```

Figure S4: Suspicious results of CRISPRDetect: The first example demonstrates a very high level of variance in the distribution of the spacer length. The second example shows height level of spacer similarity, with long (up to 24 nucleotides) pieces being shared between multiple instances. The third case demonstrates a highly repetitive structure of the spacer sequence as well. Such visual inspection allows us to claim that the presented cases highly unlikely work as CRISPR arrays

```
NC_017531-4475368-4477745

Repeats                                Spacers
GCGGGCTACGGCAGTACCCAAAC CGCAGGTGAAGAGAGCAGCCAGATG
GCGGGTTACGGCAGCACGCAGAC CGGCATGAAAGGCAGCGATCTCACC
GCCGGTTACGGCAGTACCGGCAC GGCGGGCGCTGACAGCTCTCTGATC
GCCGGTTACGGCAGTACCCAGAC GGCAGGCGAAGACAGCTCGCTGACA
GCCGGTTACGGCAGCACGCAGAC CGCGCAGAAGGGCAGCGATCTTACC
GCGGGTTATGGCAGTACCGGCAC GGCGGGTGCCGACAGTTCATTAATC
GCGGGCTACGGCAGCACCCAGAC GGCCGGGGAAGAAAGCACCCAGACA
GCCGGTTATGGCAGCACCCAGAC CGCGCAGAAGGGCAGCGACCTTACG
GCCGGTTACGGCAGTACCGGTAC GGCGGGTGACGACAGCTCCCTGATC
GCCGGTTACGGCAGTACCCAGAC GGCGGGCGAAGACAGCTCGCTGACA
GCCGGTTACGGCAGTACCCAGAC CGCGCAGAAGGGCAGCGATCTTACT
GCAGGTTATGGCAGTACCGGCAC GGCGGGTGCCGACAGTTCATTAATT
GCGGGCTACGGCAGCACCCAGAC GGCCGGGGAAGAGAGCACCCAGACA
GCCGGTTATGGCAGCACCCAGAC CGCGCAGAAGGGCAGCGACCTTACG
GCCGGTTACGGCAGCACCGGCAC GGCAGGCTCGGACAGCTCAATCATT
GCAGGTTACGGCAGCACCCAGAC CGCCAGTTATCACAGTAGCCTGACG
GCGGGCTACGGCAGTACGCAGAC GGCGCGCGAACAGAGTGTGCTGACG
ACCGGTTACGGCAGCACCTCAAC CGCCGGCGCCGACAGTTCCCTGATT
GCAGGCTATGGCAGCACGCAGAC CGCCGGTTACAACAGTATTCTGACG
GCCGGTTACGGCAGCACCCAGAC GGCGCAGGAGGGCAGCGATCTCACC
GCAGGTTATGGCAGTACCTCAAC

NC_016816-3919853-3920889
Repeats                                Spacers
GTCTGCGTGCTGCCATAGCCTGC GATCAGTGAACTATTTGCCCCCGGT
GTAGACGTACTGCCATAGCCGGT GATGAGATCGCTTTCGTTCTGCGCG
GTCTGCGTACTGCCGTAACCCGC TGTCAGCGTGCTACCGTAACCCGCT
GTCTGGGTACTGCCATAGCCCGC GATCAATGAACTTTCATAGCCTGCC
GTAGAGGTACTACCATACCCGGC CGTAAGATCGCTGCCCTTCTGCGCG
GTCTGGGTACTGCCGTAACCGGC TGTCAGTGAGCTGTCTTCGCCCGCT
GTCTGGGTACTGCCGTAACCGGC GATCAGAGAGCTGTCGTCACCCGCC
GTACCGGTACTGCCGTAACCGGC CGTAAGGTCGCTGCCCTTCTGCGCG
GTCTGGGTGCTGCCATAACCGGC TGTCTGGGTGCTCTCTTCCCCGGCC
GTCTGGGTGCTACCGTAGCCCGC GATTAATGAACTGTCGGCACCCGCC
GTGCCGGTACTGCCATAACCTGC AGTAAGATCGCTGCCCTTCTGCGCG
GTCTGGGTACTGCCGTAACCGGC TGTCAGCGAGCTGTCTTCGCCCGCC
GTCTGGGTACTGCCGTAACCGGC GATCAGGGAGCTGTCGTCACCCGCC
GTACCGGTACTGCCGTAACCGGC CGTAAGGTCGCTGCCCTTCTGCGCG
GTCTGGGTGCTGCCATAACCGGC TGTCTGGGTGCTCTCTTCCCCGGCC
GTCTGGGTGCTCCCGTAGCCCGC GATTAATGAACTGTCGGCACCCGCC
GTGCCGGTACTGCCATAACCCGC GGTAAGAGCGCTGCCCTTCTGCGCG
GTCTGCGTGCTGCCGTAACCGGC TGTCAGCGAGCTGTCTTCGCCCGCC
GTCTGGGTACTGCCGTAACCGGC GATCAGGGAGCTGTCGTCACCCGCC
GTGCCGGTACTGCCGTAACCGGC GGTGAGATCGCTGCCTTTCATGCCG
GTCTGCGTGCTGCCGTAACCCGC
```

Figure S5: Suspicious results of CRISPRCasFinder: The shown examples not only demonstrate a height level of spacer similarity but also consist of repetitive pattern which makes them unlikely perform as CRISPR arrays

10 Feature Encoding for False Arrays used in train&validate (100) and test (200) datasets

Here we provide in Table S3 the full feature table for our set of false CRISPR arrays used in the train&validation. In Table S4 we show the full feature table for our set of false CRISPR arrays used for test. Table S2 provides the description of the feature indexes listed in the Table S3 and Table S4.

Table S2: Mapping of Feature Names to Feature Indices

Feature index	Feature Name
1	Repeat Length
2	Number Repeats
3	Repeat Similarity
4	AT Richness
5	Avergae Spacer Length
6	Spacer Similarity
7	Repeat Number Mismatches
8	Spacer Evenness
9	MFE Score
10	ORF Score
11	Tandem Protein Score
12	BLAST Score Known Repeats
13	BLAST Score Similar Known Repeat

Table S3: Feature Encoding for 100 False Arrays used in train&validation

**Acc number**	**Start**	**End**	**1**	**2**	**3**	**4**	**5**	**6**	**7**	**8**	**9**	**10**	**11**	**12**	**13**
NZ CP012515	3998117	3998854	20.0	8.0	0.82	0.61	26.57	0.64	6.0	-0.0	-2.8	0.0	0.0	12.2	14.0
NC 019779	2824223	2825617	32.0	13.0	0.92	0.48	19.0	0.77	21.0	-0.0	-4.6	0.0	0.0	12.2	17.7
NZ CP010436	129935	130689	32.0	5.0	0.87	0.28	50.5	0.8	7.0	0.58	0.0	0.0	89.2	15.9	15.9
NC 018877	3816460	3817563	38.0	9.0	0.89	0.6	19.0	0.96	8.0	-0.0	-12.4	0.0	0.0	15.9	15.9
NZ CP011248	533459	536116	20.0	20.0	0.69	0.75	46.79	0.77	37.0	0.73	-1.4	0.0	0.0	12.2	0.0
NZ CP009544	3691640	3692152	20.0	5.0	0.77	0.68	38.5	0.76	4.0	0.31	-5.9	0.0	0.0	12.2	0.0
NZ CP008788	2192665	2195406	20.0	23.0	0.65	0.41	40.0	0.7	37.0	0.63	-6.2	0.0	-17.1	0.0	0.0
NZ CP010295	2388924	2389316	23.0	4.0	0.85	0.64	34.0	0.66	9.0	-0.0	-4.9	0.0	0.0	15.9	15.9
NZ LN847356	1579098	1579729	23.0	6.0	0.91	0.54	34.6	0.8	3.0	0.28	0.0	0.0	0.0	17.7	17.7
NZ CP011995	1984375	1987287	30.0	22.0	0.74	0.45	36.0	0.57	31.0	-0.0	-2.1	0.0	0.0	14.0	15.9
NC 011753	2944470	2944994	20.0	6.0	0.7	0.43	20.8	0.78	7.0	0.28	-2.8	0.0	-7.6	12.2	14.0
NZ HG917972	1633330	1634469	20.0	8.0	0.64	0.93	57.14	0.63	16.0	0.64	-4.4	0.0	0.0	14.0	14.0
NC 020532	801109	801703	29.0	5.0	0.84	0.57	29.75	0.77	9.0	-0.0	-2.7	0.0	0.0	12.2	0.0
NZ CP010028	305295	305819	19.0	5.0	0.79	0.37	35.0	0.56	9.0	0.31	-2.7	0.0	0.0	0.0	0.0
NZ CP015130	5136686	5145199	26.0	63.0	0.59	0.45	41.26	0.66	335.0	0.56	-1.6	0.0	-94.6	12.2	14.0
NZ CP013652	2797971	2799930	35.0	13.0	0.69	0.43	40.0	0.61	46.0	-0.0	-7.2	98.78	0.0	17.7	17.7
NZ CP012015	2483087	2483479	23.0	4.0	0.85	0.64	34.0	0.66	9.0	-0.0	-6.7	0.0	0.0	15.9	15.9
NZ CP013957	1227576	1228072	21.0	4.0	0.84	0.33	35.33	0.66	5.0	-0.0	-4.6	0.0	0.0	15.9	15.9
NC 018018	4227514	4228855	29.0	9.0	0.71	0.31	46.0	0.53	22.0	-0.0	0.0	0.0	0.0	17.7	17.7
NZ CP012721	4333122	4333778	30.0	5.0	0.81	0.54	33.0	0.68	7.0	0.58	-3.5	0.0	0.0	14.0	17.7
NC 008578	2208610	2211623	35.0	24.0	0.99	0.59	29.0	0.35	6.0	-0.0	-6.3	0.0	0.0	15.9	17.7
NZ CP003182	1608286	1609376	29.0	8.0	0.63	0.37	28.0	0.54	21.0	-0.0	-5.3	63.53	0.0	12.2	0.0
NZ CP013430	2055341	2055757	29.0	4.0	0.88	0.82	19.0	0.8	3.0	-0.0	-9.6	0.0	0.0	12.2	14.0
NC 020529	801154	801748	29.0	5.0	0.84	0.57	29.75	0.77	9.0	-0.0	-3.5	0.0	0.0	12.2	0.0
NZ CP008746	4316911	4319081	19.0	15.0	0.66	0.43	53.0	0.89	22.0	0.42	-2.6	91.73	0.0	14.0	14.0
NZ CP007672	608912	609668	27.0	4.0	0.8	0.53	65.0	0.92	7.0	0.61	-7.1	0.0	6.7	14.0	14.0
NC 011658	483938	485244	25.0	11.0	0.79	0.55	34.1	0.71	16.0	0.71	-0.8	0.0	-25.3	14.0	14.0
NZ CP007213	3028243	3028986	35.0	5.0	0.73	0.73	34.75	0.67	13.0	0.77	-12.1	0.0	10.4	14.0	0.0
NZ CP010526	2371806	2372196	23.0	4.0	0.85	0.64	33.67	0.71	10.0	-0.0	-3.3	0.0	0.0	15.9	15.9
NZ LN854573	2546753	2547124	20.0	4.0	0.7	0.64	27.0	0.65	8.0	0.36	-5.3	0.0	0.0	15.9	15.9
NZ CP014933	1480333	1485257	20.0	33.0	0.71	0.4	54.44	0.72	59.0	0.32	0.0	0.0	0.0	12.2	0.0
NZ CP007512	4383469	4384621	24.0	8.0	0.72	0.41	51.0	0.82	13.0	0.23	0.0	0.0	-12.2	14.0	14.0
NZ CP007674	2354519	2354911	23.0	4.0	0.85	0.64	34.0	0.66	9.0	-0.0	-4.4	0.0	0.0	15.9	15.9

Continued on next page

Table S3: Feature Encoding for 100 False Arrays used in train&validation (Cont.)

Acc_number	Start	End	1	2	3	4	5	6	7	8	9	10	11	12	13
NZ_CP013431	2204047	2205579	23.0	12.0	0.58	0.67	40.82	0.65	36.0	0.65	-4.1	0.0	14.6	15.9	15.9
NZ_CP010797	1566380	1566859	19.0	5.0	0.72	0.6	35.75	0.58	9.0	0.31	-6.7	0.0	-8.2	12.2	0.0
NZ_CP009623	1965609	1966412	32.0	6.0	0.76	0.61	40.0	0.8	12.0	0.28	-1.3	0.0	0.0	14.0	14.0
NZ_CP009600	3827955	3828555	21.0	4.0	0.85	0.56	57.0	0.91	4.0	0.36	-2.5	0.0	0.0	12.2	17.7
NC_015564	3258771	3259412	20.0	5.0	0.89	0.62	41.5	0.76	3.0	0.31	-1.0	0.0	-10.0	14.0	15.9
NC_021150	3726696	3727786	19.0	11.0	0.74	0.57	30.8	0.63	18.0	-0.0	0.0	0.0	-0.5	14.0	14.0
NC_002953	596648	597604	35.0	6.0	0.81	0.52	38.2	0.77	17.0	0.53	-5.7	0.0	6.3	14.0	0.0
NC_015383	402908	404076	19.0	9.0	0.89	0.6	43.88	0.51	5.0	-0.0	-1.4	0.0	0.0	14.0	14.0
NZ_CP015126	1116861	1117373	23.0	5.0	0.78	0.52	28.0	0.68	8.0	-0.0	0.0	0.0	0.0	14.0	14.0
NZ_CP009281	5810329	5811276	32.0	9.0	0.88	0.58	20.0	0.69	13.0	-0.0	-5.2	0.0	0.0	14.0	19.6
NZ_CP010322	2049748	2050368	30.0	5.0	0.81	0.54	33.0	0.68	7.0	0.58	-2.3	0.0	0.0	14.0	17.7
NC_010002	2281385	2282319	20.0	7.0	0.69	0.78	44.0	0.72	13.0	-0.0	-1.1	0.0	0.2	12.2	0.0
NZ_LN606601	4164	4690	21.0	5.0	0.81	0.41	28.5	0.5	5.0	0.58	0.0	0.0	0.0	14.0	14.0
NC_015567	4175167	4175535	23.0	4.0	0.87	0.41	24.0	0.63	2.0	0.36	-0.2	0.0	0.0	14.0	17.7
NZ_CP006861	1954433	1956763	23.0	17.0	0.8	0.64	45.25	0.71	14.0	0.79	-2.1	0.0	0.0	12.2	14.0
NZ_CP009300	3698986	3699650	19.0	6.0	0.67	0.46	41.0	0.73	14.0	0.38	0.0	0.0	-0.3	15.9	17.7
NC_014550	2979564	2982817	22.0	25.0	0.63	0.51	43.0	0.73	48.0	0.21	-5.2	0.0	-35.9	12.2	0.0
NC_019679	21557	22632	35.0	8.0	0.98	0.6	36.86	0.45	1.0	0.38	-4.2	0.0	0.0	17.7	27.0
NZ_CP010890	778762	779238	29.0	4.0	0.87	0.58	29.67	0.73	8.0	-0.0	-3.7	0.0	0.0	12.2	0.0
NC_021823	1365335	1370059	29.0	29.0	0.73	0.66	52.43	0.75	113.0	0.82	-1.8	0.0	0.0	12.2	0.0
NZ_CP004004	79216	82126	35.0	17.0	0.79	0.65	49.0	0.79	31.0	-0.0	-7.4	94.89	0.0	14.0	0.0
NC_018515	322925	325467	26.0	19.0	0.72	0.34	40.56	0.72	65.0	0.61	-7.3	0.0	0.0	14.0	14.0
NC_016109	2679913	2680307	19.0	4.0	0.82	0.24	26.0	0.68	3.0	0.36	-2.6	0.0	0.0	12.2	0.0
NC_003210	1870245	1872869	23.0	21.0	0.71	0.66	39.4	0.7	40.0	0.75	-1.4	0.0	0.0	14.0	14.0
NZ_CP013274	1195529	1196106	23.0	5.0	0.73	0.36	33.25	0.67	6.0	0.58	-1.8	0.0	0.0	12.2	0.0
NZ_CP012330	2023326	2026936	23.0	28.0	0.65	0.53	41.67	0.6	61.0	0.72	-0.1	98.28	0.0	12.2	0.0
NZ_CP013527	2883295	2884599	29.0	12.0	0.74	0.34	25.55	0.69	22.0	0.33	-11.9	0.0	0.0	0.0	15.9
NZ_CP013727	3620963	3624099	20.0	21.0	0.51	0.38	54.25	0.58	58.0	0.89	-0.3	0.0	0.0	0.0	0.0
NC_022793	777016	777390	20.0	4.0	0.73	0.6	26.0	0.6	7.0	0.36	-1.8	0.0	0.9	17.7	17.7
NZ_CP007606	894386	894784	25.0	4.0	0.8	0.3	32.0	0.6	4.0	-0.0	-1.4	0.0	0.0	15.9	15.9
NZ_CP012117	487748	489279	28.0	10.0	0.76	0.41	53.67	0.61	18.0	0.54	-0.3	0.0	0.0	14.0	17.7
NC_019567	3031767	3032521	21.0	6.0	0.68	0.39	27.6	0.53	11.0	0.38	0.0	0.0	0.0	14.0	14.0
NZ_CP007690	2389073	2389465	23.0	4.0	0.85	0.64	34.0	0.66	9.0	-0.0	0.0	0.0	0.0	15.9	15.9
NZ_CP013231	2523770	2524526	27.0	4.0	0.8	0.53	65.0	0.92	7.0	0.61	-0.3	0.0	6.7	14.0	14.0
NZ_CP007492	1849978	1851135	20.0	10.0	0.67	0.67	39.33	0.73	22.0	0.73	-0.1	0.0	0.0	12.2	0.0
NZ_CP008782	2527739	2531061	27.0	20.0	0.82	0.7	57.0	0.82	27.0	-0.0	-7.2	0.0	0.0	12.2	0.0
NZ_CP004002	2949741	2950541	20.0	7.0	0.75	0.71	37.0	0.69	7.0	0.74	-3.5	0.0	12.7	0.0	0.0
NZ_CP009156	1086525	1089520	36.0	18.0	0.85	0.72	48.0	0.82	27.0	-0.0	-6.3	61.12	-31.7	14.0	0.0
NZ_CP009506	2277781	2279021	25.0	9.0	0.73	0.68	44.0	0.58	15.0	-0.0	0.0	0.0	0.0	14.0	14.0
NZ_CP013730	1166286	1167659	20.0	11.0	0.83	0.84	42.1	0.81	13.0	0.79	-2.1	0.0	-103.4	0.0	0.0
NZ_CP012159	1853186	1853776	23.0	6.0	0.79	0.25	19.0	0.94	8.0	-0.0	-4.1	0.0	0.0	12.2	15.9
NZ_CP007684	1869183	1871495	35.0	16.0	0.78	0.66	36.6	0.74	63.0	0.66	-6.2	0.0	0.0	12.2	0.0
NC_010334	2117421	2118092	20.0	8.0	0.84	0.38	22.43	0.63	8.0	0.33	0.0	0.0	0.0	15.9	15.9
NC_010572	7548444	7548946	35.0	4.0	0.83	0.31	19.0	0.75	12.0	-0.0	-9.7	0.0	-8.0	12.2	0.0
NZ_CP007499	1952918	1953334	36.0	4.0	0.86	0.49	21.67	0.66	18.0	-0.0	-4.4	0.0	0.0	15.9	15.9
NZ_CP009235	912075	912627	20.0	6.0	0.78	0.57	26.6	0.66	5.0	-0.0	-0.9	0.0	0.0	12.2	14.0
NC_009380	1620246	1621965	20.0	16.0	0.67	0.75	34.0	0.66	37.0	0.45	-0.3	0.0	0.0	12.2	0.0
NZ_CP008732	1644121	1644798	19.0	7.0	0.67	0.4	30.5	0.59	11.0	0.25	-1.8	0.0	0.0	12.2	14.0
NC_009089	3781410	3782147	20.0	7.0	0.78	0.48	28.0	0.7	5.0	0.36	0.0	0.0	0.0	14.0	19.6
NZ_CP016926	801030	806642	20.0	47.0	0.58	0.56	39.61	0.67	106.0	0.61	-1.8	0.0	-56.3	12.2	14.0
NZ_CP006858	1988851	1991181	23.0	17.0	0.8	0.64	45.25	0.71	14.0	0.79	-0.2	0.0	0.0	12.2	14.0
NC_020291	2169570	2172757	33.0	24.0	0.67	0.42	32.35	0.61	109.0	0.71	-6.3	99.99	0.0	12.2	0.0
NZ_CP009913	1534055	1536259	26.0	13.0	0.71	0.48	55.0	0.65	22.0	0.63	-6.3	0.0	-19.7	12.2	0.0
NZ_CP010905	798925	800006	21.0	8.0	0.82	0.83	46.57	0.72	6.0	-0.0	0.0	0.0	0.0	14.0	17.7
NZ_CP010978	3733705	3735559	21.0	16.0	0.8	0.59	38.4	0.77	15.0	0.48	-3.6	0.0	0.0	14.0	14.0
NZ_CP006934	851886	852992	28.0	7.0	0.93	0.49	47.0	0.81	6.0	0.61	-6.8	0.0	5.5	0.0	0.0
NC_015978	571071	571911	19.0	7.0	0.7	0.65	44.5	0.69	13.0	0.56	-3.2	0.0	0.0	12.2	0.0
NZ_CP010904	1849761	1850078	19.0	4.0	0.74	0.58	27.33	0.68	7.0	0.36	-2.2	0.0	0.0	0.0	0.0
NZ_CP013145	2675699	2676334	21.0	5.0	0.79	0.83	41.25	0.82	13.0	0.31	0.0	0.0	0.0	15.9	15.9

Continued on next page

Table S3: Feature Encoding for 100 False Arrays used in train&validation (Cont.)

Acc number	Start	End	1	2	3	4	5	6	7	8	9	10	11	12	13
NC_016021	970	1471	20.0	6.0	0.81	0.59	26.2	0.46	5.0	0.28	-1.8	0.0	0.0	14.0	14.0
NC_017477	1571276	1574275	20.0	19.0	0.64	0.41	60.0	0.75	26.0	0.54	0.0	0.0	0.0	14.0	14.0
NZ_CP011774	2637335	2637877	20.0	4.0	0.7	0.35	53.0	0.81	9.0	0.36	0.0	0.0	-12.3	12.2	14.0
NZ_CP015125	1404261	1404725	26.0	5.0	0.8	0.6	19.0	0.67	11.0	-0.0	-0.8	0.0	0.0	12.2	0.0
NZ_CP012041	2251278	2252075	38.0	6.0	0.83	0.62	30.4	0.62	26.0	0.59	-4.8	0.0	-16.8	14.0	0.0
NC_019780	963158	963739	24.0	6.0	0.86	0.62	20.4	0.74	6.0	0.28	0.0	0.0	0.0	14.0	14.0
NC_019693	5228838	5229335	38.0	4.0	0.89	0.6	21.67	0.77	7.0	0.36	-2.7	0.0	0.0	14.0	0.0
NZ_CP007676	2358395	2358787	23.0	4.0	0.85	0.64	34.0	0.66	9.0	-0.0	-3.3	0.0	0.0	15.9	15.9

Table S4: Feature Encoding for the False Arrays used in Test Dataset (200)

Acc number	Start	End	1	2	3	4	5	6	7	8	9	10	11	12	13	Score
NC_009782	849204	849535	29	5	0.79	0.43	29.75	0.69	9	0.00	-12	0	0	12.2	0	0.26
NC_010296	80874	81761	36	11	0.69	0.63	40.80	0.65	32	0.28	-4.7	0	-27	12.2	0	0.19
NC_009641	1178378	1178566	19	4	0.74	0.32	37.00	0.49	4	0.00	-2.1	0	0	12.2	0	0.26
NC_009523	5627400	5627809	20	7	0.78	0.20	29.00	0.47	11	0.36	-6	0	0	14	14	0.32
JNJK01000014	63561	63890	24	7	0.51	0.67	27.00	0.37	23	0.00	0	0	-15	14	15.9	0.16
NC_015275	3710171	3710454	21	4	0.84	0.67	54.00	0.60	2	0.36	0	0	0	15.9	17.7	0.27
NZ_CP010874	1925688	1926112	22	3	0.79	0.52	44.00	0.73	2	0.00	-0.6	0	15	14	14	0.22
FAOZ01000020	23972	24327	26	7	0.48	0.39	29.00	0.38	28	0.36	-3.1	0	0	12.2	0	0.11
NC_009697	1713974	1714890	23	19	0.73	0.77	23.00	0.60	27	0.24	0	0	0	15.9	17.7	0.23
AOLS01000060	45695	45852	23	4	0.52	0.34	22.00	0.31	10	0.00	-1.9	0	0	12.2	14	0.14
NC_007795	549935	550533	23	9	0.74	0.52	43.00	0.76	12	0.58	-0.5	0	4.6	12.2	14	0.16
LGDF01000002	91883	92079	23	5	0.42	0.27	20.50	0.35	21	0.31	-2.5	0	0	12.2	0	0.09
CACA01000349	20263	20468	23	5	0.49	0.27	22.75	0.33	18	0.00	0	0	0	14	17.7	0.12
NC_009641	603852	604306	23	6	0.78	0.50	46.60	0.80	5	0.53	-0.5	0	7	12.2	14	0.18
NZ_CP007739	2469188	2469435	24	3	0.81	0.56	21.00	0.77	2	0.00	-1.4	0	0	14	15.9	0.23
NC_008312	2941354	2941964	37	3	0.81	0.72	32.00	0.83	8	0.39	-1.6	0	0	15.9	15.9	0.20
NZ_CP004858	679851	680046	28	3	0.69	0.74	26.00	0.48	4	0.00	-1.2	0	0	14	15.9	0.32
NC_006368	3077960	3078399	20	9	0.70	0.44	26.13	0.68	11	0.21	-2.5	0	0	14	14	0.18
NC_007355	1460785	1462223	19	33	0.66	0.52	24.13	0.70	59	0.36	-5.3	81	0	14	14	0.16
KB235903	2637262	2637467	23	5	0.49	0.27	22.75	0.33	18	0.00	0	0	0	14	17.7	0.12
NZ_CP015150	653095	653290	28	3	0.69	0.74	26.00	0.48	4	0.00	-1.2	0	0	14	15.9	0.32
NC_014539	3447067	3449363	29	20	0.55	0.44	53.42	0.52	89	0.48	-7.9	100	0	14	15.9	0.19
NZ_CP009605	2741998	2742959	20	16	0.66	0.35	40.00	0.62	37	0.70	-2.6	0	0	14	14	0.15
NC_007350	2157482	2157623	31	3	0.81	0.60	23.50	0.29	2	0.00	-4.3	0	0	12.2	14	0.42
NC_005957	810175	810539	22	6	0.62	0.50	39.20	0.65	15	0.38	-3.6	0	-1.9	19.6	19.6	0.26
NC_007168	328624	336938	28	154	0.68	0.59	26.12	0.62	323	0.02	-1.3	0	0	12.2	0	0.18
NZ_CP010089	769186	769381	28	3	0.69	0.74	26.00	0.48	4	0.00	-1.2	0	0	14	15.9	0.32
NC_004842	810291	810823	20	3	0.91	0.52	35.50	0.49	1	0.39	-0.3	0	0	12.2	14	0.44
NC_007348	235268	235762	19	6	0.68	0.32	35.60	0.52	10	0.38	-1.8	0	0	15.9	15.9	0.24
LN875035	444907	445068	25	4	0.50	0.69	20.67	0.32	12	0.36	0	0	0	12.2	14	0.11
NZ_CP015529	897815	898131	28	3	0.68	0.38	27.50	0.29	5	0.00	-9.9	0	0	12.2	0	0.36
NC_009675	2435709	2436031	29	4	0.91	0.27	41.00	0.94	3	0.00	-6.3	0	0	14	14	0.35
NC_008095	2276709	2277028	37	5	0.77	0.31	32.00	0.72	7	0.39	-9.6	0	0	0	0	0.22
JXJP01000032	20757	21007	23	5	0.53	0.53	34.00	0.33	17	0.31	0	0	0	0	0	0.15
NC_009481	2216523	2219380	23	3	0.74	0.51	40.00	0.40	3	0.00	0	0	0	12.2	0	0.36
NC_006274	4573795	4575591	35	14	0.59	0.43	34.92	0.57	97	0.67	-1.2	0	0	14	0	0.18
NC_017030	2080275	2081002	38	4	0.66	0.32	31.33	0.39	13	0.00	-13	0	0	14	0	0.38
CP003422	3770218	3770406	25	4	0.42	0.56	29.67	0.34	16	0.36	-5.8	0	0	12.2	0	0.12
NC_009705	121426	121681	26	4	0.79	0.58	43.67	0.57	6	0.00	-5.1	0	0	14	14	0.31
CM000721	2352168	2352477	25	7	0.49	0.41	22.50	0.37	33	0.25	-6.3	0	0	12.2	0	0.13
NC_008600	832661	833007	21	5	0.65	0.46	54.00	0.71	9	0.31	-2.5	0	0.3	12.2	17.7	0.19
BAXE01000020	72584	72777	23	5	0.46	0.55	19.75	0.31	24	0.31	0	0	-13	12.2	0	0.09
NZ_CP004870	693915	694110	28	3	0.69	0.74	26.00	0.48	4	0.00	-1.2	0	0	14	15.9	0.32
KB912877	4515179	4515384	23	4	0.48	0.43	38.00	0.34	14	0.61	0	0	0	12.2	15.9	0.11
JOJB01000005	276150	276307	23	4	0.51	0.28	22.00	0.34	10	0.00	-0.5	0	0	14	14	0.15
NZ_AP012327	282753	282990	26	3	0.69	0.40	27.00	0.23	5	0.00	-4.4	0	0	14	14	0.39

Continued on next page

Table S4: Feature Encoding for the False Arrays used in Test Dataset 200, Cont.)

Acc number	Start	End	1	2	3	4	5	6	7	8	9	10	11	12	13	Score
NC 009675	3812215	3812378	37	3	0.85	0.25	26.00	0.55	2	0.39	-14	0	0	15.9	15.9	0.40
NC 009074	3395922	3396370	29	6	0.76	0.37	42.40	0.64	15	0.28	-13	0	-17	14	14	0.29
AUJY01000030	11961	12206	24	5	0.43	0.59	31.50	0.31	20	0.39	-2	0	0	12.2	15.9	0.11
ANFZ01000006	117570	117740	24	4	0.51	0.26	25.00	0.31	15	0.36	-6.5	0	0	0	0	0.16
NZ CP014113	118037	118178	31	3	0.81	0.60	23.50	0.29	2	0.00	-4.3	0	0	12.2	14	0.42
NC 010175	5026857	5027959	38	15	0.84	0.36	31.00	0.76	22	0.49	-7.7	0	-54	12.2	0	0.20
NC 005956	1609332	1609973	19	14	0.68	0.44	25.92	0.63	18	0.44	0	0	0	0	14	0.14
NZ CP009554	2040188	2040503	26	3	0.75	0.54	32.00	0.45	4	0.00	-2	0	0	15.9	15.9	0.35
FR883783	25535	25794	26	6	0.49	0.27	20.80	0.36	27	0.38	-6.9	0	0	12.2	0	0.14
NZ CP009499	2403497	2403599	28	2	0.90	0.25	55.00	1.00	4	0.00	-1.8	0	0	17.7	17.7	0.40
NZ CP009686	3285603	3285735	20	3	0.70	0.47	34.00	0.41	2	0.00	-0.9	0	0	0	0	0.31
NC 017362	122581	122842	20	4	0.72	0.71	36.00	0.49	7	0.36	0	0	67.7	15.9	15.9	0.26
KI929931	337	581	29	5	0.52	0.57	25.00	0.32	21	0.00	-1.6	0	0	12.2	14	0.15
NC 008702	677308	677864	26	7	0.57	0.31	22.00	0.44	22	0.00	-5.2	0	0	15.9	15.9	0.25
NZ CP012282	9331	9485	35	2	1.00	0.54	19.00	1.00	0	0.00	-6.2	0	0	15.9	15.9	0.39
NC 009654	864939	865395	33	5	0.86	0.41	20.75	0.88	4	0.00	-9.5	0	0	12.2	0	0.28
NC 002758	847759	848090	29	5	0.79	0.43	29.75	0.69	9	0.00	-12	0	0	12.2	0	0.26
HG426535	24050	24236	23	5	0.47	0.95	18.00	0.32	26	0.39	-0.2	0	0	0	0	0.09
NC 021485	2005949	2006205	29	5	0.67	0.53	22.00	0.51	16	0.31	-1.3	0	17.6	12.2	0	0.20
NC 009656	6197309	6197841	25	7	0.72	0.25	42.00	0.77	13	0.25	-8.3	0	-20	12.2	0	0.23
NC 014371	1121404	1121858	42	5	0.80	0.67	27.00	0.40	8	0.00	-12	0	0	15.9	15.9	0.42
NC 003923	290102	290304	23	4	0.77	0.38	32.33	0.52	10	0.00	-4.7	0	0	15.9	15.9	0.32
LIPU01000008	4639	4856	23	5	0.51	0.32	25.75	0.38	16	0.39	-5.1	0	0	12.2	0	0.16
NC 005945	4333033	4333408	30	6	0.80	0.46	31.20	0.60	7	0.59	0	0	0	14	17.7	0.25
LOWC01000021	31667	31833	26	4	0.53	0.21	21.00	0.27	17	0.36	-6.3	0	3.1	12.2	0	0.16
NC 007912	4501013	4501281	26	4	0.68	0.38	54.00	0.62	6	0.36	-0.8	0	0	0	0	0.18
NC 008229	1410864	1411108	24	4	0.83	0.57	42.00	0.76	6	0.00	0	0	0	14	14	0.28
NC 002952	2068152	2068467	26	3	0.75	0.54	32.00	0.45	4	0.00	-2	0	0	15.9	15.9	0.35
NC 004193	3168886	3169178	33	5	0.70	0.61	19.00	0.51	9	0.00	-6.5	0	0	12.2	15.9	0.28
NC 016816	3919853	3920889	23	8	0.53	0.35	25.00	0.35	20	0.00	-5.4	0	0	17.7	17.7	0.24
NC 009921	1813547	1815030	21	26	0.76	0.24	36.96	0.67	21	0.33	-8.7	0	0	12.2	0	0.21
NC 022234	791590	792043	44	4	0.60	0.29	41.00	0.55	24	0.00	-16	0	0	14	0	0.33
NC 010184	1192724	1193073	30	5	0.68	0.31	46.50	0.64	17	0.00	-3.7	0	0	12.2	0	0.22
NC 009767	3980358	3980745	30	6	0.75	0.49	28.00	0.57	9	0.38	-8.7	0	0	12.2	14	0.26
NC 008785	3317106	3317469	19	7	0.68	0.40	27.50	0.53	9	0.00	-6.5	0	0	12.2	14	0.23
NC 012472	800906	801252	20	5	0.69	0.52	41.50	0.58	6	0.39	-3.2	0	-0.5	12.2	15.9	0.20
NZ AP014710	1250965	1251226	20	4	0.61	0.70	35.67	0.54	14	0.36	0	0	72.7	12.2	14	0.19
NC 009617	3028582	3029704	26	20	0.54	0.49	29.89	0.57	84	0.61	-4.6	0	0	14	14	0.15
NZ CP015377	1476683	1477047	23	2	0.83	0.22	37.00	1.00	1	0.00	-10	0	0	14	14	0.29
NZ CP012727	1182797	1183497	23	12	0.84	0.39	36.73	0.71	9	0.65	-4.8	0	0	12.2	0	0.23
NC 007530	2006790	2007166	25	6	0.86	0.63	20.00	0.79	4	0.00	-2.7	0	8.6	14	15.9	0.28
NC 008817	16297	16521	35	3	0.94	0.29	32.50	0.87	2	0.39	-11	0	-3.7	12.2	0	0.29
NZ CP014773	2630865	2631015	25	3	0.87	0.39	37.00	0.54	1	0.39	0	0	0	14	14	0.40
NZ CP014485	3980346	3980479	30	3	0.77	0.27	21.00	0.46	3	0.00	-5.6	0	0	12.2	15.9	0.36
NC 008268	746326	746876	25	7	0.92	0.29	39.00	0.90	3	0.61	-2.7	0	-40	14	14	0.28
JNXY01000001	781266	781494	23	5	0.47	0.13	28.50	0.34	20	0.31	-7.2	0	0	12.2	0	0.12
NC 008312	2705071	2705869	33	11	0.88	0.80	43.30	0.84	42	0.45	0	0	0	0	0	0.22
NC 007795	1776485	1776694	37	4	0.79	0.49	20.67	0.46	19	0.00	-14	0	0	15.9	15.9	0.43
NZ CP009361	1976285	1976656	19	7	0.71	0.33	38.50	0.49	12	0.00	-1.3	0	0	12.2	0	0.30
NC 008531	1275766	1276100	32	6	0.67	0.53	19.00	0.62	17	0.00	-4.6	0	0	15.9	15.9	0.24
NZ CP015235	4986204	4986341	29	3	0.83	0.31	28.00	0.36	3	0.00	-12	0	0	12.2	14	0.45
NC 009641	2393433	2393632	23	4	0.79	0.36	34.00	0.47	9	0.00	-5.9	0	0	15.9	15.9	0.40
NC 010397	4126774	4126909	29	2	0.70	0.33	34.00	1.00	9	0.00	-8.9	0	0	12.2	14	0.23
NC 008278	5918314	5918630	29	5	0.88	0.22	28.00	0.60	4	0.58	-12	0	-19	12.2	15.9	0.35
NC 016935	6483669	6483808	23	3	0.63	0.62	25.50	0.35	6	0.39	-9.5	0	0	15.9	15.9	0.31
NC 006274	5100784	5101124	29	4	0.81	0.60	63.00	0.75	3	0.00	0	0	-1.5	17.7	17.7	0.28
NC 008686	2414379	2419621	29	65	0.65	0.34	52.28	0.69	264	0.84	-3.3	0	0	12.2	14	0.16
AULQ01000006	297646	298017	24	7	0.50	0.61	34.00	0.36	28	0.25	0	0	0	12.2	0	0.11

Continued on next page

Table S4: Feature Encoding for the False Arrays used in Test Dataset 200, Cont.)

Acc number	Start	End	1	2	3	4	5	6	7	8	9	10	11	12	13	Score
NZ_CP006824	177466	177727	20	4	0.72	0.71	36.00	0.49	7	0.36	0	0	67.7	15.9	15.9	0.26
NZ_CP010089	545652	545847	28	3	0.69	0.74	26.00	0.48	4	0.00	-1.2	0	0	14	15.9	0.32
NC_012466	1593167	1599960	20	99	0.53	0.39	48.88	0.56	221	0.66	-0.7	96	-185	12.2	0	0.12
NC_008542	958596	959047	23	6	0.67	0.24	46.00	0.73	12	0.00	-5.7	0	0	12.2	0	0.21
NC_008836	1628022	1628385	19	7	0.68	0.40	27.50	0.53	9	0.00	-6.5	0	0	12.2	14	0.23
NC_009698	1714458	1715374	23	19	0.73	0.77	23.00	0.60	27	0.24	0	0	0	15.9	17.7	0.23
NZ_CP017107	1181346	1185268	20	63	0.61	0.47	42.52	0.66	114	0.44	-1.4	90	-410	12.2	14	0.15
NC_010001	3379434	3379872	20	6	0.62	0.43	53.80	0.70	12	0.53	-4.3	0	0	0	14	0.14
NC_008711	606878	607176	23	6	0.84	0.28	23.80	0.85	6	0.28	-3.3	0	0	14	17.7	0.26
NC_008060	412091	412542	23	6	0.67	0.24	46.00	0.73	12	0.00	-5.7	0	0	12.2	0	0.21
NC_005945	1182799	1183499	23	12	0.84	0.39	36.73	0.71	9	0.65	-4.8	0	0	12.2	0	0.23
NC_017354	134641	134902	20	4	0.72	0.71	36.00	0.49	7	0.36	0	0	67.7	15.9	15.9	0.26
NC_009074	3872717	3872927	19	4	0.70	0.36	27.67	0.46	4	0.00	-6.5	0	0	12.2	14	0.32
LCZE01000018	322275	322504	23	5	0.49	0.25	28.75	0.51	25	0.39	0	0	0	0	0	0.10
AZVO01000016	60652	61445	26	15	0.42	0.34	28.86	0.39	74	0.33	-5.7	0	0	12.2	0	0.12
NC_010380	1756475	1758729	20	35	0.49	0.46	45.29	0.56	135	0.62	-1.3	100	-50	12.2	0	0.07
NC_007510	2982675	2983459	20	14	0.76	0.22	20.62	0.58	10	0.00	-3.4	0	-18	14	14	0.23
NC_009767	2245979	2246754	22	10	0.60	0.25	53.56	0.61	19	0.56	-1.7	0	0	0	0	0.12
NZ_CP016921	144789	145077	23	5	0.75	0.43	41.50	0.55	7	0.00	-2.9	0	0	15.9	15.9	0.27
NC_015125	868060	868393	20	7	0.76	0.39	30.83	0.41	6	0.00	-1.1	0	0	12.2	0	0.34
LLVT01000001	916642	916982	23	7	0.48	0.31	30.00	0.36	29	0.25	-2.7	0	0	0	0	0.12
NC_004193	2887912	2888255	19	7	0.75	0.66	33.00	0.61	5	0.00	0	0	0	14	17.7	0.23
NC_010175	1058980	1059446	20	8	0.74	0.23	37.86	0.57	6	0.53	-4.5	0	-8.1	15.9	15.9	0.20
AYYE01000893	3850	4041	24	5	0.51	0.50	18.00	0.25	13	0.00	-1	0	0	14	14	0.15
GL383451	61432	61587	26	4	0.53	0.29	17.33	0.16	16	0.00	-2.2	0	0	0	0	0.14
NC_015856	4382491	4386410	32	3	0.62	0.46	49.00	0.45	6	0.39	-3.2	0	0	14	14	0.28
JVLX01000039	20753	21146	34	7	0.50	0.61	26.00	0.38	42	0.00	0	0	229	12.2	0	0.12
NC_004567	1197910	1202642	26	58	0.54	0.50	55.47	0.63	212	0.77	-2.9	0	-109	12.2	0	0.10
NC_013131	4906278	4906708	26	7	0.60	0.27	29.00	0.57	20	0.36	-3.9	0	0	12.2	0	0.22
NC_009480	2246887	2247155	26	5	0.73	0.28	34.75	0.45	6	0.31	-6.9	0	0	17.7	17.7	0.39
LPLL01000085	163847	163996	24	4	0.50	0.34	18.00	0.32	13	0.00	-2	56	0	0	0	0.10
NC_010184	2320623	2321242	22	9	0.76	0.42	46.63	0.73	17	0.68	-1.8	0	0	14	14	0.17
KB976220	2178454	2178674	23	5	0.37	0.34	26.50	0.35	26	0.31	-0.6	0	0	12.2	0	0.06
NZ_CP009613	4108979	4109114	29	2	0.70	0.33	34.00	1.00	9	0.00	-8.9	0	0	12.2	14	0.23
NC_009953	5470909	5471423	28	3	0.79	0.29	86.00	0.63	3	0.00	-5.5	0	18.3	0	14	0.24
NC_006348	3049523	3049886	19	7	0.60	0.40	30.50	0.50	11	0.25	-6.9	0	0	12.2	14	0.22
NC_003155	932230	932578	19	7	0.66	0.38	27.00	0.51	11	0.56	-1.9	0	0	0	14	0.16
KL585394	392952	393144	28	4	0.51	0.28	27.00	0.37	18	0.36	-5.8	0	0	12.2	15.9	0.17
ARZI01000043	17497	17802	25	8	0.38	0.37	15.14	0.35	44	0.23	-7.2	0	0	12.2	0	0.08
NC_010380	1745843	1753569	28	43	0.53	0.45	45.29	0.52	154	0.63	-2.7	100	-82	12.2	0	0.12
NC_009648	1130208	1137295	26	104	0.64	0.43	42.10	0.63	296	0.67	-3.1	0	-179	0	14	0.16
NC_004342	3293986	3294716	37	10	0.57	0.66	32.00	0.54	48	0.00	-4.4	0	0	15.9	15.9	0.21
DS989854	134495	134783	25	5	0.46	0.50	41.00	0.35	20	0.58	-3.4	73	46.2	12.2	0	0.09
JNYP01000002	123884	124080	23	5	0.42	0.27	20.50	0.35	21	0.31	-2.5	0	0	12.2	0	0.09
AMRN01000008	14974	15374	23	6	0.43	0.51	52.60	0.45	19	0.28	0	96	0	0	14	0.08
LHXI01000003	399380	399648	23	6	0.44	0.42	26.20	0.36	20	0.28	-2.2	0	4.3	12.2	14	0.11
NZ_CP016476	172787	173264	28	3	0.72	0.51	47.00	0.36	4	0.00	-4.9	0	0	15.9	15.9	0.43
LCBF01000035	20218	20394	24	4	0.42	0.49	27.00	0.37	15	0.00	-2.9	0	0	12.2	0	0.11
NC_004722	3677314	3678740	20	27	0.57	0.36	34.35	0.64	70	0.63	-6.1	0	0	14	14	0.14
NC_012026	811998	812192	20	4	0.87	0.50	37.00	0.65	3	0.36	0	0	0	15.9	15.9	0.30
NC_017531	4475368	4477745	19	17	0.59	0.32	29.00	0.45	38	0.00	-7.4	0	0	15.9	15.9	0.29
NC_009663	2032815	2033001	23	3	0.90	0.58	49.00	0.52	1	0.00	-0.3	0	0	12.2	15.9	0.45
NC_008027	168728	169266	23	7	0.66	0.22	41.00	0.65	12	0.25	-4.5	0	-20	12.2	0	0.20
ATYW01000003	358893	359449	23	10	0.43	0.24	36.33	0.37	45	0.64	-6.3	0	0	12.2	15.9	0.13
NZ_CP012409	762621	762813	25	3	0.77	0.40	32.00	0.40	9	0.00	-13	0	0	15.9	15.9	0.43
NC_010407	1303030	1303406	29	6	0.74	0.33	29.80	0.53	16	0.00	-10	0	0	12.2	14	0.31
KB913013	4816071	4816313	27	5	0.48	0.39	27.00	0.32	17	0.00	-2	0	0	12.2	0	0.11
NC_016023	2424991	2425482	23	4	0.68	0.57	29.00	0.49	11	0.00	0	0	0	14	14	0.28

Continued on next page

Table S4: Feature Encoding for the False Arrays used in Test Dataset 200, Cont.)

Acc number	Start	End	1	2	3	4	5	6	7	8	9	10	11	12	13	Score
NC¯009926	147523	147707	22	4	0.76	0.31	38.00	0.74	9	0.61	-1.4	0	0	12.2	0	0.14
NC¯004461	2471130	2472496	35	19	0.62	0.50	36.00	0.54	81	0.68	-2.9	0	-16	14	0	0.20
NC 007776	1670003	1670361	23	2	1.00	0.57	19.00	1.00	0	0.00	-2.9	0	0	14	14	0.35
¯ KE386573	327251	327442	24	5	0.43	0.38	18.00	0.36	24	0.00	-1.6	0	19	12.2	14	0.10
NZ CP014955	4134094	4134229	29	2	0.70	0.33	34.00	1.00	9	0.00	-8.9	0	0	12.2	14	0.23
¯NC 011831	4526455	4527433	30	11	0.86	0.31	56.30	0.65	8	0.34	-6.3	0	0	12.2	15.9	0.30
NZ CP013049	4131821	4131956	29	2	0.70	0.33	34.00	1.00	9	0.00	-8.9	0	0	12.2	14	0.23
NC 019695	1606114	1606249	33	2	0.89	0.41	39.00	1.00	1	0.00	0	0	0	15.9	17.7	0.38
CP004378	2371156	2371461	25	8	0.38	0.37	15.14	0.35	44	0.23	-7.2	0	0	12.2	0	0.08
NC¯008312	1551526	1552193	19	17	0.71	0.44	21.50	0.72	30	0.21	-2.1	0	-2.3	12.2	0	0.14
NC 009632	865485	865757	36	4	0.68	0.47	22.67	0.55	14	0.00	-12	0	0	15.9	15.9	0.32
AMLP01000065	7239	7455	25	5	0.50	0.34	23.00	0.38	25	0.31	-5.9	0	-3.2	12.2	14	0.12
NC 014335	4453611	4454857	21	20	0.50	0.42	41.05	0.56	63	0.57	0	0	0	14	14	0.13
CP003345	2284595	2284796	25	4	0.51	0.56	34.00	0.33	15	0.36	0	0	0	12.2	15.9	0.16
NC¯010582	1724457	1727575	26	13	0.47	0.45	40.00	0.52	59	0.57	-3.4	0	-11	14	14	0.09
NC 009921	8122175	8122663	23	8	0.52	0.17	32.29	0.58	36	0.53	-7.8	0	-15	12.2	0	0.14
AFQT01000010	136524	136917	34	7	0.50	0.61	26.00	0.38	42	0.00	0	0	229	12.2	0	0.12
NC¯008555	1844601	1845463	20	14	0.57	0.33	43.69	0.68	27	0.85	-1.8	0	0	12.2	0	0.10
¯NC 007793	2388919	2389118	23	4	0.79	0.36	34.00	0.47	9	0.00	-5.9	0	0	15.9	15.9	0.40
NZ CP012292	9331	9485	35	2	1.00	0.54	19.00	1.00	0	0.00	-6.2	0	0	15.9	15.9	0.39
NC¯007498	2709282	2709622	22	5	0.90	0.34	56.00	0.66	3	0.58	-8.9	0	0	12.2	0	0.30
NC¯008533	1392763	1393660	23	17	0.60	0.49	28.75	0.65	45	0.31	-1.6	0	-0.5	12.2	14	0.18
NC 021554	833589	833801	20	3	0.72	0.50	38.00	0.50	6	0.00	0	0	0	15.9	15.9	0.29
CP002869	3117883	3118071	25	4	0.42	0.56	29.67	0.34	16	0.36	-5.8	0	0	12.2	0	0.12
JQOK01000063	43167	43353	25	4	0.54	0.79	29.00	0.33	15	0.36	0	0	0	12.2	0	0.17
NC¯008784	1254710	1255158	23	9	0.63	0.28	25.38	0.56	21	0.37	-4.4	0	12.4	12.2	0	0.25
NC¯005957	1206404	1206727	23	4	0.86	0.39	40.00	0.59	2	0.36	-4.8	0	0	12.2	0	0.33
NC¯016935	3812993	3813240	21	3	0.79	0.59	35.50	0.33	2	0.39	-9.3	0	0	14	14	0.41
NC 007511	490250	492990	20	51	0.62	0.29	33.28	0.64	88	0.13	-4.5	0	0	12.2	0	0.18

11 Feature Encoding for Spurious Arrays in CRISPRCasFinder data set.

We show in Table S5 the arrays predicted as positive by other CRISPR detection tools, for which we have evidence that they might be false CRISPR arrays. The mapping of numbers to features is again given in Table S2.
See Table S5 in Supplementary 2

12 CRISPRidentify Database and Standalone version

The core of the CRISPRidentify pipeline is implemented in python. The user may download the source code and supporting libraries from our github web page. We recommend to use Conda package to easily set up the python environment whichis necessary for the correct performance of our method. The user is provided with the detailed instructions about each set up step. We also demonstrate how to successfully run our algorithm over the input data. We explain the potential parameters of the search which user might want to set to tailor their search according to their desires.

Table S6: **CRISPR database characteristics.** We ran CRISPRidentify on the comprehensive dataset and aggregated corresponding statistics separately for archaeal and bacterial genomes. Candidates with certainty score of 0.75 or higher belong to the Bona fide category. Cases with the certainty score between 0.75 and 0.4 are marked as Possible. The rest of the results, with the certainty scores below 0.4 are labeled Low score

	Archaea			Bacteria		
No. Genomes	987			27028		
No. Arrays	5884			151062		
Category	Bona fide	Possible	Low score	Bona fide	Possible	Low score
No. Array candidates	1403	1174	2472	37975	47491	65596
Repeat length	30.5	30.8	26.4	30.7	32.8	26.2
Spacer length	37.6	35.6	29.3	33.9	33.2	33.7

Figure S6: **CRISPR database output.** For each repeat consensus sequence, we list all genomic locations of arrays having this consensus repeat, in addition with the organism information. Furthermore, we output the number of these locations and the number of organisms where an array with this consensus repeat is found.

Repeat	Genomic Locations of associated CRISPR Arrays	# Arrays	# Organisms
Consensus Sequence	Organism (Array coordinates)		
1. GTGTTCCCCGCGCCAGCGGGGATAAACCG	• LAPQ01000046 (316713, 318407)<br>• LAPQ01000046 (334399, 336073)<br>• AETS01000059 (44687, 45222)<br>• JHIL01000003 (186692, 187044)	4	3
. . .	. . .	. . .	. . .

13 Hyper Parameters for different ML classifiers

For KNN classifier we varied the number of k in between 1 and 10

For SVM classifier we used different kernels: "poly", "rbf", "sigmoid", "linear"

For the Neural Network classifier we tried different hidden layer representations: [10], [10 100 100], [100 100]

For Random Forest classifier and Extra trees classifier we tried different number of trees in the ensemble (between 1 and 15) as well as different tree length (between 1 and 6)

14 Cas types in the test set

Table S7 Cas types of 550 CRISPR arrays used for the positive test set. Cas types were obtained with CRISPRCasIdentified.

Cas type	# of cases
CAS-I-B	162
CAS-I-E	96
CAS-I-C	54
CAS-II-A	37
CAS-III-A	35
Unknown	32
CAS-I-U	27
CAS-II-C	27
CAS-I-F	26
CAS-III-B	15
CAS-III-D	13
CAS-I-D	6
CAS-I-A	6
CAS-IV-A	4
CAS-V-A	3
CAS-II-B	3
CAS-III-C	3
CAS-VI-B	1

15 CRISPR arrays unique to CRISPRidentify

See CRISPR array cases in Supplementary 3

16 CRISPR arrays with damaged repeat

Table S8 Statistics of cases with damaged repeats in the comprehensive dataset

Category	Number of arrays
Total number of arrays	39378
Arrays with damaged 5' repeat	783
Arrays with damaged 3' repeat	8893
Arrays with damaged 5' and 3' repeats	749
Arrays with severe damaged 5' repeat	257
Arrays with severe damaged 3' repeat	4840
Arrays with severe damaged 3' and 5' repeat	28

Examples of suspicious candidates in CRT results in the dot repeat notation.

```
KI912609
(2863598, 2865967)
AGGTTCGTTT.T.T............  CGCATAAGGGATGCCGCCGCAGGTACATCTCTCGATAGTTT         s:12 i:0 d:0
..........................  CGTGCTCCTACACCGTCAGTATACCATGAACCGTTT              s:0 i:0 d:0
..........................  CGAGACCTCACGTTGCGGCATTATCCGTTAAGTTT               s:0 i:0 d:0
..........................  GAGGTGCGTCGCAATGGTAGAGCTTGAATTTCTTTTGTTT          s:0 i:0 d:0
..........................  CCGTACAACTCCATCAAATTAGGTTCAGCAGATTCCGGTTT         s:0 i:0 d:0
..........................  AACTTCGGCAATTAAGTCTCTTAACTCCTTATCTGTGTTT          s:0 i:0 d:0
..........................  CGTCACGGCTTCTGCATCAGGCGAACTTCCGTTT                s:0 i:0 d:0
..........................  TAAGGGAGAGCCTGTTTAGGCTCCCCCTCTTCCAGTCGTTC         s:0 i:0 d:0
..........................  TAAGGGAGAGCCTGTTTAGGCTCCCCCTCTTCCAGTCGTTC         s:0 i:0 d:0
..........................  CCAGGCTAACAAAGAGATGACGCTCGTACACCGCGTTC            s:0 i:0 d:0
..........................  CCCGGCCAAAATGCGTATATAATGAAGCAAAAACCGCGTTC         s:0 i:0 d:0
..........................  CTCCACCGACATAGCGGCGGCAATGGAGATTCTTGAAAGTTC        s:0 i:0 d:0
..........................  ACGCGAGCTGGGATACGGTTAATGGTGAGGAAGTCCGCACGTTC      s:0 i:0 d:0
..........................  ATGGTCAAGCATTCGTGCCCGACCAAAAGCTGGGGGTTC           s:0 i:0 d:0
..........................  CCTGGGCGGCCAGCTGGTGAAGCGGGAGAAAAACTTGGTTC         s:0 i:0 d:0
..........................  CCAGGCTAACAAAGAGATGACGCTCGTACACCGCGTTC            s:0 i:0 d:0
..........................  CAACAATGCCAACGATTACAGTGGCAGTGTGCACCGTTC           s:0 i:0 d:0
..........................  AGGTTTGCTCACTACGTTGAATAGAGTTTTAGGGGGTTC           s:0 i:0 d:0
..........................  TCTTCATCATCCCAAAACTCCCAATCGCCAAATTGAAGTTC         s:0 i:0 d:0
..........................  GAGTGTTCTTGTATCTGCCGAGCCACATCCCTACGGGTTC          s:0 i:0 d:0
..........................  TCTCCATCTTTCACGCTCAGCTTCAGCGCCAGGGCAATGTTC        s:0 i:0 d:0
..........................  CAACGCAGCGGCTTCGCCTTCCGCGCGGGGGGGATGGGTTC         s:0 i:0 d:0
..........................  CCCGGTGCCTCAATTCCCCGGCTCTCATTACAACCGTTC           s:0 i:0 d:0
..........................  TTGTGCCATAATTGCATTCCGCAATAAGATGATAAATGTTC         s:0 i:0 d:0
..........................  TATCTGTGATATACCGTTTCGCCTAAACTGGCATGGTTC           s:0 i:0 d:0
..........................  TACGAATACTACTCTTCATCCGTTACATCAGTTT                s:0 i:0 d:0
..........................  ACATGATACCCAGGCGAGACGCGTTTCATCATTGTTT             s:0 i:0 d:0
..........................  CGTGCCGATGTAAGACGACGAATTCCTTCCGGTTT               s:0 i:0 d:0
.............T........C...  TGAGCGTGCCAGCACTTCATCTCGATAGTTTTCGTTT             s:2 i:0 d:0
..........................  TCATCCGGATTCTACGGATAGGTCTGCAATTTCGGTTT            s:0 i:0 d:0
..........................  ACTTCAGCAGATCAATCCAACCGATATCTGCGTTT               s:0 i:0 d:0
..........................  CAGGACAAAGACGTAGAGTGGAACGGGGTGCTTAAGTTC           s:0 i:0 d:0
..........................  TAGCCCATTTCCCTAAGCATTTCTTCCCCCTGGTTTAGTTC         s:0 i:0 d:0
..........................  AGCCGCTTATGTACAGCATAGGTGTCCGTGTAAAAGGTTC          s:0 i:0 d:0
..........................  GTGGAGTCGTTCAGGAACAAATAACCGCCAAACCTGGTTC          s:0 i:0 d:0
..........................  GCCGGGCATGCCGAGGGGGAGATGAAAGCCAATGCGTTC           s:0 i:0 d:0
..........................                                                    s:0 i:0 d:0
_____________________________________________________________________________________________
TCAGCGTACCTATGAGGAATTGAAAC
```

```
BAWB01000030.fasta.pkl
(48258, 49487)
GA.ATTCAATTT.TGT.TT.............  AAAACGGAGCTGGTTGCAATATCATTAAAAAGG          s:16 i:0 d:0
.........A...........T.........C  GTTTGAACCAGTCTATTATTTAGAAATGATGGAC         s:3 i:0 d:0
......................A.........  ATGGCGACCTCTCATCGGATTAACAGAACAAGAATT       s:1 i:0 d:0
............A....T..............  TAGGTAGAACAGTAACCATAAATAAAGAAAATAAAA       s:2 i:0 d:0
.....................G..........  GTTCGCCGTTGGTGCTTATTATGGCAAAAAGCAAT        s:1 i:0 d:0
..................CTTGT.........  ATTAAACACACCAAGCTTTTCCCCTAATTCACT          s:5 i:0 d:0
............A.T.................  TTAAGCTCGTTTGGCGTATCAACGTTTATGGACGGA       s:2 i:0 d:0
................................  ACCGCGATAAGTTCATCCAAGAGAACGTTTCTTT         s:0 i:0 d:0
................................  GCATTTAGTCGGCGACATTACCGAAGTTATTGCG         s:0 i:0 d:0
................................  TTCCATTTTTGTTGTTAGACCTGTTTCAATGCTT         s:0 i:0 d:0
........G.......................  TAGAGAATATAATAACCAATAGATAGAATGCAC          s:1 i:0 d:0
................................  AAGGATTTGCACGACATGCGCACCTTTTTAAACAG        s:0 i:0 d:0
................................  TTGAGCGTGCTAAAGCTTGGATCAAGGAGCAGGAAGA      s:0 i:0 d:0
................................  GTGGTCTTAAACGCATCGAACCATTCTTCAAATA         s:0 i:0 d:0
................................  GAAACGCTCATGCTGTCCAGTGGTATCCAATCGAA        s:0 i:0 d:0
................................  CATGACGTTCTGGGGTTGCTCAATCTTTGATAATG        s:0 i:0 d:0
................................  GCAAATTTATTAGGAATTAAAGCAGGTACGGCAAA        s:0 i:0 d:0
................................  CAGCGTAATTATCTGTTTGACTGTCTTTATAAA          s:0 i:0 d:0
................................                                             s:0 i:0 d:0
ATTTCAATCCACGCACTCACAAGGAGTGCGAT


JJOV01000045.fasta.pkl
(6242, 9666)
CA..TC.AT.CACACA..CTTGT...C.T...  TATATTATCAATGTTTATTCTGGAACTTATAA            s:19 i:0 d:0
TA..TC.AT.TACACA...T............  CATCTACATTGTATTTCTGATCCTGTAGATAT            s:13 i:0 d:0
GGA.TTCAATC.....................  CAGTGATTTGTCCTTCGTCCTGTACCTGCAGG            s:10 i:0 d:0
................................  GCTTATATCGTTTACAACCGTAACGTCGTTTGT           s:0 i:0 d:0
................................  TTTTACAACAACACGATCATAGGGACCTCAAACG          s:0 i:0 d:0
................................  ATCAGTTAAAGGGCAACCCTCATCTATCCATTTTT         s:0 i:0 d:0
................................  CTCGAATCTCATGAAGGATGGGTACAAACCACAA          s:0 i:0 d:0
................................  AAATGTATCTGCTTTGCCTATGGCGTCCATTAAC          s:0 i:0 d:0
................................  CTGAATGTCATTAACGGTGATATTGATGCCTACA          s:0 i:0 d:0
................................  AAAATATACAGGCTAAATTTCTACCTTGATTTTTT         s:0 i:0 d:0
................................  TGCGAGGCATATCCCGTTTCCGAGGTAGCAGATA          s:0 i:0 d:0
................................  AAAATACCTTATTTTGTAGTATCCGTTTAAGGATT         s:0 i:0 d:0
................................  TACTTTTCCCTCAACTGACTACACCGGCTTAACT          s:0 i:0 d:0
................................  TTGCCGTTCATTATCTGAAAGCGCTTTACACATCTTT       s:0 i:0 d:0
................................  CGTTTTTATAAACGGCCCCATTCAAAACACTAATTA        s:0 i:0 d:0
................................  TTTAGAGTAAGCTGGAGTTTTCCCCCAGCTGCAAAAAC      s:0 i:0 d:0
................................  CATTGATTCGGCTGTTTCAGCGTTATCATTTT            s:0 i:0 d:0
................................  TTTCTACCTCAAACCAGTATAGCCCATCAGGGTA          s:0 i:0 d:0
................................  TAAAGTGCGGGCAAAATTCATGAGCTTCATCAAG          s:0 i:0 d:0
................................  GAGGATTTCCGGTATGGAGTACTAAAACAGTTCCT         s:0 i:0 d:0
................................  ATTCTGGAAAAGTGAATACACATAAAAACACTTTAACC      s:0 i:0 d:0
................................  TTGCTCATCTTTGGTTATCCTCCTCTAGCCCCTTGA        s:0 i:0 d:0
................................  ATTCTTAGAACGCATTTTCTACCGTCCATAATTT          s:0 i:0 d:0
................................  CAGGAGATTTCAGGATAGTAAAGACAGATGCAAGT         s:0 i:0 d:0
................................  GTTAAAAATATCTGGCTTTCCCGTCAGGGCAGAAG         s:0 i:0 d:0
................................  CTCAGGCATTTTGATGTATTGGGCTGCATGTGTGTAA       s:0 i:0 d:0
................................  TACTGGATTAAGAGCGATATGGTTAGATTCTACAG         s:0 i:0 d:0
................................  GAGATATTGTGATAAAATAGATGAATTGGAGAAAA         s:0 i:0 d:0
................................  GAGGGGCCTGCAAGACCGGCAATGTCTGAAGAA           s:0 i:0 d:0
................................  CAGACTTCTCCATTGGTTGTATAGATATGAAATT          s:0 i:0 d:0
................................  GCCATACCACTTATAACAAAGTGGGAGTCCTGGGAG        s:0 i:0 d:0
................................  AAGGGAAATCTGTAAACGTACTAATGAACCATTCC         s:0 i:0 d:0
................................  CGCTGAAATTAACGCCAGGAATGCACCAATAAAC          s:0 i:0 d:0
................................  TCCATAATATGTTTGGATATACCCCGAGTGCAAAT         s:0 i:0 d:0
................................  TATTGTAATATCCTGAGATTTGTCTTCATATGAA          s:0 i:0 d:0
................................  AACATGGATCTTGTAAGGTCAATGAGGGTTAATCTC        s:0 i:0 d:0
................................  ACTTCGACATCATATGATGGTCGGGTTTTCTG            s:0 i:0 d:0
................................  CTTATCTTTGATTTCTCCGGTCCATTCATCGGT           s:0 i:0 d:0
................................  TACCTTTTATTTTGACGCTACGGTCATAAGTTAT          s:0 i:0 d:0
................................  CTTCAAAAGCCGTTTAAATCACTCCTCCCAAGTT          s:0 i:0 d:0
................................  GGGAATAAAGCCTCTTTATCATGGTGCAACATGCAA        s:0 i:0 d:0
................................  ATTTGTTTTTCAGCGATCTTGTTAAATCCTTTT           s:0 i:0 d:0
................................  AAGATCAAGCATTTCAGAGAGTAGATTCATTCATT         s:0 i:0 d:0
................................  AGCAGGTTCTTCATTGAGTCTTTTAAACTGTCCTC         s:0 i:0 d:0
................................  ATTCTCTATTTATGAAAACAGAACAGGCATAAGCA         s:0 i:0 d:0
................................  ACTTCAGCTACACCTTCATAGCGGTCATTGTTAA          s:0 i:0 d:0
................................  ATTATTCCACCTCAAATATCTTATTAAATTGAGCCA        s:0 i:0 d:0
................................  ATCTGATGTTGGAGAGATTTAATCTTACCAGTTCA         s:0 i:0 d:0
................................  CTCTCTAATCTGCTTAGAATGCCCTTCGGATTGAT         s:0 i:0 d:0
................................  ATAGGTGGAAAGAAAGATCGAGGACGTATGAAG           s:0 i:0 d:0
................................  AACTTCAGCTACACCTTCATAGCGGTCATTATTAA         s:0 i:0 d:0
................................                                              s:0 i:0 d:0

ATTTCAATCCACACACCCGCGAAGGGTGCGAC
```

Example of spurious CRISPRCasFinder result:

```
CRISPRCasFinder output
# DR: CTTATCTGCCGCATAGGCAGCTTAGAAA DR_length: 28 Number_of_spacers: 9
#=========================================================================
Spacer_begin_position Spacer_length Spacer_sequence
               727655            32 AGCGCAATTCGAAGCTGGGTAAGAAGGCTGAC

               727715            32 TTGATGTCACACTTCACGAACCTCGAGGAGTC

               727775            32 GTGTCAAGATTATCCCAGGTCTGTTCCCGTAC

               727835            32 TGCTGCAGGTCATAGTTGCTTGGCCGGTCCTG

               727895            32 TTATTGAATGCACGATTCAGCAGCTTCTTTTC

               727955            32 GCCTTGAGCAGCTGGCAGAGGAGAAGGAAGGT

               728015            32 AGTGGGAGGGCTTGGAACGACGAACGGTTCCA

               728075            32 GTCTCATCCGCCGCCGTAAAGCGCGCAAACGA

               728135            32 GTGGCGGTAGCAAACATGATTGCACGCCACAA

#=========================================================================
########################################
```

Dot representation of the same array

```
 ............................  AGCGCAATTCGAAGCTGGGTAAGAAGGCTGAC      s:0 i:0 d:0
 .................T..........  TTGATGTCACACTTCACGAACCTCGAGGAGTC      s:1 i:0 d:0
 ............................  GTGTCAAGATTATCCCAGGTCTGTTCCCGTAC      s:0 i:0 d:0
 ...........C................  TGCTGCAGGTCATAGTTGCTTGGCCGGTCCTG      s:1 i:0 d:0
 ...........C................  TTATTGAATGCACGATTCAGCAGCTTCTTTTC      s:1 i:0 d:0
 ............................  GCCTTGAGCAGCTGGCAGAGGAGAAGGAAGGT      s:0 i:0 d:0
 ............................  AGTGGGAGGGCTTGGAACGACGAACGGTTCCA      s:0 i:0 d:0
 ............................  GTCTCATCCGCCGCCGTAAAGCGCGCAAACGA      s:0 i:0 d:0
 C..........C................  GTGGCGGTAGCAAACATGATTGCACGCCACAA      s:2 i:0 d:0
 C..........C....ATG.G.A.ATCC                                        s:11 i:0 d:0
____________________________________________________________________________________________________
     GTTATCTGCCGTATAGGCAGCTTAGAAA
```

Comparsion CRISPRDeted and CRISPRidentify results (first representation corresponds to CRISPRidentify the second corresponds to CRISPRDetect):

```
CP003355

1647332   ........................ ... .....  TTGTTTCGATGCTATATTTATACGCATCAATA          s:0 i:0 d:0
1647396   ........................ ... .....  AAGGGAGGGGATTTTCAGACTCAGTTTAGACTATAC      s:0 i:0 d:0
1647464   ........................ ... .....  ATTACAGGGGCAGGGAGGTACAGAAGATAGGAGGTAC     s:0 i:0 d:0
1647533   ........................ ... .....  ACTAGAGCAATGAGCATTAACGGGATTCCAATCA        s:0 i:0 d:0
1647599   ........................ ... .....  AAGGGAGGGGATTTTCAGACTCAGTTTAGACTATAC      s:0 i:0 d:0
1647667   ........................ ... .....  ATTACAGGGGCAGGGAGGTACAGAAGATAGGAGGTAC     s:0 i:0 d:0
1647736   ........................ ... .....  ACTAGAGCAATGAGCATTAACGGGATTCCAATCA        s:0 i:0 d:0
1647802   ........................ ... .....  CAATTAAGCCGACCGCCATATAGCGTGGCTAT          s:0 i:0 d:0
1647866   ...............A..-A..T.A...GC....                                            s:4 i:2 d:1
__________________________________________________________________________________________________________
          GTCGCATCCTACGCGGATGCGTGG ATT GAAAT                                            s:4 i:2 d:1


  Position Repeat         %id     Spacer     Repeat_Sequence                  Spacer_Sequence
           Insertion/Deletion
========== ======     ======     ======     ================================ =====================================
           ==================
   1647332     32      100.0         32     ................................ TTGTTTCGATGCTATATTTATACGCATCAATA
   1647396     32      100.0         36     ................................ AAGGGAGGGGATTTTCAGACTCAGTTTAGACTATAC
   1647464     32      100.0         37     ................................ ATTACAGGGGCAGGGAGGTACAGAAGATAGGAGGTAC
   1647533     32      100.0         34     ................................ ACTAGAGCAATGAGCATTAACGGGATTCCAATCA
   1647599     32      100.0         36     ................................ AAGGGAGGGGATTTTCAGACTCAGTTTAGACTATAC
   1647667     32      100.0         37     ................................ ATTACAGGGGCAGGGAGGTACAGAAGATAGGAGGTAC
   1647736     32      100.0         34     ................................ ACTAGAGCAATGAGCATTAACGGGATTCCAATCA
   1647802     32      100.0          0     ................................ |
========== ======     ======     ======     ================================ =====================================
           ==================
         8     32      100.0         35     GTCGCATCCTACGCGGATGCGTGGATTGAAAT
```

```
JYPQ01000006

5322    ......... ............. ......  TCGTATCCCTGCAGCTCCAGCGCCCCTTCCTT       s:0 i:0 d:0
5383    ......... ............. ......  GTGTGATCTCATTTGGCGAGGGGTGCGTAAAA       s:0 i:0 d:0
5444    ......... ............. ......  AAGAGCGTCATATGCCTGCAAGGCAAAGACCA       s:0 i:0 d:0
5505    ......... ............. ......  TTCGTTAATTATATTACAATACAAATCATAAC       s:0 i:0 d:0
5566    .........C............. ......  AGAGCGGCACTTACCTTAGTCCGCCGAACGCC       s:0 i:1 d:0
5628    ......... ............. ......  ACAAAAATCGCCGACGGCATTCGTACTGGTCA       s:0 i:0 d:0
5689    ......... ............. ......  CGGTGCCCCATATTGGCTATATGCTGACGCAA       s:0 i:0 d:0
5750    ......... ............. ......  CCAGTATCGGTAGCGCGGGTGAGGCGGCGGCC       s:0 i:0 d:0
5811    ......... ............. ......  TATTTTCTCGTGTGGCGGTCCATTGGCCGCTA       s:0 i:0 d:0
5872    ......... ............. ......  CTCATAGCTCGGATCACCGCGATATACCGGATC      s:0 i:0 d:0
5934    ......... ............. ......  GGAGGACGCTACTATCGGATTCGCTCCGGGGA       s:0 i:0 d:0
5995    ......... ............. ......  GGCGGCAATATCCATCACTCAGCACCAGGGAA       s:0 i:0 d:0
6056    ......... ............. ......  GGGTCAAATATGGGGTTATTGGGAGGGAGCGG       s:0 i:0 d:0
6117    ......... ............. ......  CCCCTACAGTTGCCACCGCGTACAACTCAACA       s:0 i:0 d:0
6178    ......... ...T......... ......  CCATACCATTTCTACTGGCGCAACGATTTTTG       s:1 i:0 d:0
6239    ......... ......A.AA....A--G...                                        s:4 i:1 d:2
_____________________________________________________________________________________________
        GTGTTCCCC GCGCCAGCGGGAT AAACCG                                         s:5 i:2 d:2


  Position Repeat         %id     Spacer     Repeat_Sequence                  Spacer_Sequence
           Insertion/Deletion
========== ======      ======     ======     =============================    =================================
           ==================
      5322     29      100.0         32      .............................    TCGTATCCCTGCAGCTCCAGCGCCCCTTCCTT
      5383     29      100.0         32      .............................    GTGTGATCTCATTTGGCGAGGGGTGCGTAAAA
      5444     29      100.0         32      .............................    AAGAGCGTCATATGCCTGCAAGGCAAAGACCA
      5505     29      100.0         32      .............................    TTCGTTAATTATATTACAATACAAATCATAAC
      5566     29      100.0         32      .............................    AGAGCGGCACTTACCTTAGTCCGCCGAACGCC           C [5575]
      5628     29      100.0         32      .............................    ACAAAAATCGCCGACGGCATTCGTACTGGTCA
      5689     29      100.0         32      .............................    CGGTGCCCCATATTGGCTATATGCTGACGCAA
      5750     29      100.0         32      .............................    CCAGTATCGGTAGCGCGGGTGAGGCGGCGGCC
      5811     29      100.0         32      .............................    TATTTTCTCGTGTGGCGGTCCATTGGCCGCTA
      5872     29      100.0         33      .............................    CTCATAGCTCGGATCACCGCGATATACCGGATC
      5934     29      100.0         32      .............................    GGAGGACGCTACTATCGGATTCGCTCCGGGGA
      5995     29      100.0         32      .............................    GGCGGCAATATCCATCACTCAGCACCAGGGAA
      6056     29      100.0         32      .............................    GGGTCAAATATGGGGTTATTGGGAGGGAGCGG
      6117     29      100.0          0      .............................    |
========== ======      ======     ======     =============================    =================================
           ==================
        14     29      100.0         32      GTGTTCCCCGCGCCAGCGGGATAAACCG
```

17 CRISPR arrays with identical spacers

On one hand, identical spacers might indicate that the considered candidate is a false CRISPR array. On the other hand it has been shown that identical spacers might be present in a functional CRISPR array [7]. In our approach we provide the user with a flexible set of options: if the user is interested in filtering out the cases with the same spacers occurring more than once we allow them to do so running the tool with the (see User guide). In this case the user should input the maximum number of identical spacers in the array. On top of that we allow the user to filter out cases where identical spacers occur sequentially forming a "cluster" (see User guide). If neither option is applied our approach will not filter out the candidate necessarily but might do it based on the corresponding ML model and the value of the spacer similarity.

We also provide full description of found arrays in the Supplementary Materials 4.

Table S9 Identical spacers in the comprehensive dataset

Category	Number of arrays
Total number of arrays	39378
Arrays with no identical spacers	35543
Arrays with one pair of identical spacers	934
Arrays with two pairs of identical spacers	362
Arrays with three pairs of identical spacers	123
Arrays with one triplet of identical spacers	9
Arrays with two triplets of identical spacers	15
Arrays with three triplets of identical spacers	1
Arrays with cluster of two identical spacers	2279
Arrays with cluster of three identical spacers	112

Table S10 Identical spacers in the train set

Category	Number of arrays
Total number of arrays	1000
Arrays with no identical spacers	807
Arrays with one pair of identical spacers	44
Arrays with two pairs of identical spacers	27
Arrays with three pairs of identical spacers	12
Arrays with one triplet of identical spacers	0
Arrays with two triplets of identical spacers	4
Arrays with three triplets of identical spacers	0
Arrays with cluster of two identical spacers	94

Arrays with cluster three identical spacers	12

Table S11 Identical spacers in the test set

Category	Number of arrays
Total number of arrays	550
Arrays with no identical spacers	459
Arrays with one pair of identical spacers	21
Arrays with two pairs of identical spacers	16
Arrays with three pairs of identical spacers	2
Arrays with one triplet of identical spacers	0
Arrays with two triplets of identical spacers	0
Arrays with three triplets of identical spacers	0
Arrays with cluster of two identical spacers	48
Arrays with cluster three identical spacers	4

Here we present six spurious arrays provided by CRT (2 arrays), CRISPRDetect(2 arrays) and CRISPRCasFinder (2 arrays).

```
CRISPR 1   Range: 10667 - 12383
POSITION REPEAT                               SPACER
-------- -----------------------------------        -----------------------------------
10667           ATGTAGATGTATTCCAGTATAATAAGGATTAAGAC        GCGAATCTGTTCTTCGGCAGCTTTCCAGTACGTT [ 35, 34 ]
10736           ATGTAGATGTATTCCAGTATAATAAGGATTAAGAC        GCGAATCTGTTCTTCGGCAGCTTTCCAGTACGTT [ 35, 34 ]
10805           ATGTAGATGTATTCCAGTATAATAAGGATTAAGAC        GCGAATCTGTTCTTCGGCAGCTTTCCAGTACGTT [ 35, 34 ]
10874           ATGTAGATGTATTCCAGTATAATAAGGATTAAGAC        GCGAATCTGTTCTTCGGCAGCTTTCCAGTACGTT [ 35, 34 ]
10943           ATGTAGATGTATTCCAGTATAATAAGGATTAAGAC        GCGAATCTGTTCTTCGGCAGCTTTCCAGTACGTT [ 35, 34 ]
11012           ATGTAGATGTATTCCAGTATAATAAGGATTAAGAC        GCGAATCTGTTCTTCGGCAGCTTTCCAGTACGTT [ 35, 34 ]
11081           ATGTAGATGTATTCCAGTATAATAAGGATTAAGAC        ACTTTGGCGAAGACCGCGGCGTTGCGGTACTTAACG         [ 35, 36 ]
11152           ATGTAGATGTATTCCAGTATAATAAGGATTAAGAC        AAACTTGGTCCAAGTTGCAGGATCACTATTATAAAGTT       [ 35, 38 ]
11225           ATGTAGATGTATTCCAGTATAATAAGGATTAAGAC        ACACGACCACACCATAAAAAGCGCGGCGATAATT [ 35, 34 ]
11294           ATGTAGATGTATTCCAGTATAATAAGGATTAAGAC        GATAAATATTTCCATATTCTTGGTTTTTAAGTGAA          [ 35, 35 ]
11364           ATGTAGATGTATTCCAGTATAATAAGGATTAAGAC        ATTTCCTCACCAGAACATGAGGAGAACATTACAA [ 35, 34 ]
11433           ATGTAGATGTATTCCAGTATAATAAGGATTAAGAC        GTAATACCTTTCAGTAACCGCCACCTTAGAAAATTGT        [ 35, 37 ]
11505           ATGTAGATGTATTCCAGTATAATAAGGATTAAGAC        AGTTCGTCTTGGATTTCTTCCGCAACGATTGT   [ 35, 32 ]
11572           ATGTAGATGTATTCCAGTATAATAAGGATTAAGAC        TCACGGCGGTCTTCTAAACGTTCAGCCTCGGCGGT          [ 35, 35 ]
11642           ATGTAGATGTATTCCAGTATAATAAGGATTAAGAC        AAATTATCCTCGTTGCACAGGACTATAAGACAGC [ 35, 34 ]
11711           ATGTAGATGTATTCCAGTATAATAAGGATTAAGAC        GTTAAGTGGTTAACCATTTGGCTAAGACGGTAAGCG         [ 35, 36 ]
```

```
11782           ATGTAGATGTATTCCAGTATAATAAGGATTAAGAC       GTTAAGTGGTTAACCATTTGGCTAAGACGGTAAGCG       [ 35, 36 ]
11853           ATGTAGATGTATTCCAGTATAATAAGGATTAAGAC       GTTAAGTGGTTAACCATTTGGCTAAGACGGTAAGCG       [ 35, 36 ]
11924           ATGTAGATGTATTCCAGTATAATAAGGATTAAGAC       GTTAAGTGGTTAACCATTTGGCTAAGACGGTAAGCG       [ 35, 36 ]
11995           ATGTAGATGTATTCCAGTATAATAAGGATTAAGAC       GTTAAGTGGTTAACCATTTGGCTAAGACGGTAAGCG       [ 35, 36 ]
12066           ATGTAGATGTATTCCAGTATAATAAGGATTAAGAC       GTTAAGTGGTTAACCATTTGGCTAAGACGGTAAGCG       [ 35, 36 ]
12137           ATGTAGATGTATTCCAGTATAATAAGGATTAAGAC       GTTAAGTGGTTAACCATTTGGCTAAGACGGTAAGCG       [ 35, 36 ]
12208           ATGTAGATGTATTCCAGTATAATAAGGATTAAGAC       ACTTTTTTCAATCCATAGCCTTTGACTGTATTAGT        [ 35, 35 ]
12278           ATGTAGATGTATTCCAGTATAATAAGGATTAAGAC       CAATGCCCAGAAATACTGCAAACTTTCCTTATTAGC       [ 35, 36 ]
12349           ATGTAGATGTATTCCAATATAAAAATTTAACTACA
-------- -----------------------------------       -----------------------------------
Repeats: 25     Average Length: 35                Average Length: 35


CRISPR 1   Range: 764229 - 765839
POSITION REPEAT                           SPACER
-------- ----------------------------     -------------------------------------
764229          TGTTTTGGAACCATTCGAAACAGCACAG      CTCTAAAACAACGTTGTCATATCCGCTCGTTCCCTCGT     [ 28, 38 ]
764295          AGTTTTGGAACCATTCGAAACAGCACAG      CTCTAAAACAACGTTGTCATATCCGCTCGTTCCCTCGT     [ 28, 38 ]
764361          AGTTTTGGAACCATTCGAAACAGCACAG      ATCTAAAACCTCTTGCTTGGAAATTATGTCCACAGCTA     [ 28, 38 ]
764427          TGTTTTGGAACCATTCGAAACAGCACAG      ATCTAAAACCTCTTGCTTGGAAATTATGTCCACAGCTA     [ 28, 38 ]
764493          TGTTTTGGAACCATTCGAAACAGCACAG      ATCTAAAACCTCTTGCTTGGAAATTATGTCCACAGCTA     [ 28, 38 ]
764559          TGTTTTGGAACCATTCGAAACAGCACAG      ATCTAAAACCTCTTGCTTGGAAATTATGTCCACAGCTA     [ 28, 38 ]
764625          TGTTTTGGAACCATTCGAAACAGCACAG      CTCTAAAACAAACATAATGAAACTTTAACGGCTTCTGG     [ 28, 38 ]
764691          TGTTTTGGAACCATTCGAAACAGCACAG      ATCTAAAACCTCTTGCTTGGAAATTATGTCCACAGCTA     [ 28, 38 ]
764757          TGTTTTGGAACCATTCGAAACAGCACAG      ATCTAAAACCTCTTGCTTGGAAATTATGTCCACAGCTA     [ 28, 38 ]
764823          TGTTTTGGAACCATTCGAAACAGCACAG      ATCTAAAACCTCTTGCTTGGAAATTATGTCCACAGCTA     [ 28, 38 ]
764889          TGTTTTGGAACCATTCGAAACAGCACAG      ATCTAAAACCTCTTGCTTGGAAATTATGTCCACAGCTA     [ 28, 38 ]
764955          TGTTTTGGAACCATTCGAAACAGCACAG      CTCTAAAACAAACATAATGAAACTTTAACGGCTTCTGG     [ 28, 38 ]
765021          TGTTTTGGAACCATTCGAAACAGCACAG      ATCTAAAACCTCTTGCTTGGAAATTATGTCCACAGCTA     [ 28, 38 ]
765087          TGTTTTGGAACCATTCGAAACAGCACAG      ATCTAAAACCTCTTGCTTGGAAATTATGTCCACAGCTA     [ 28, 38 ]
765153          TGTTTTGGAACCATTCGAAACAGCACAG      ATCTAAAACCTCTTGCTTGGAAATTATGTCCACAGCTC     [ 28, 38 ]
765219          TGTTTTGGAACCATTCGAAACAGCACAG      CTCTAAAACCGTATACTGATGTTGCTGGCAACTGTCA      [ 28, 37 ]
765284          TGTTTTGGAACCATTCGAAACAGCACAG      CTCTAAAACTTGAAAGGGCAGTTTTACGAATGTGCTAC     [ 28, 38 ]
765350          GGTTTTGGAACCATTCGAAACAGCACAG      CTCTAAAACCTCTTGCTTGGAAATTATGTCCACAGCTA     [ 28, 38 ]
765416          TGTTTTGGAACCATTCGAAACAGCACAG      ATCTAAAACCTCTTGCTTGGAAATTATGTCCACAGCTA     [ 28, 38 ]
765482          TGTTTTGGAACCATTCGAAACAGCACAG      ATCTAAAACCTCTTGCTTGGAAATTATGTCCACAGCTA     [ 28, 38 ]
765548          TGTTTTGGAACCATTCGAAACAGCACAG      CTCTAAAACTATCGTACCAATTGAGTTCAATCCAGCTC     [ 28, 38 ]
765614          TGTTTTGGAACCATTCGAAACAGCACAG      CTCTAAAACAAACATAATGAAACTTTAACGGCTTCTGG     [ 28, 38 ]
765680          TGTTTTGGAACCATTCGAAACAGCACAG      CTCTAAAACCTCTTGCTTGGAAATTATGTCCACAGCTA     [ 28, 38 ]
765746          TGTTTTGGAACCATTCGAAACAGCACAG      CTCTAAAACTGCTCGCCGTCCAAAACTTCGCCCGTTTC     [ 28, 38 ]
765812          TGTTTTGGAACCATTCGAAACAGCACAG
-------- ----------------------------     -------------------------------------
Repeats: 25     Average Length: 28                Average Length: 37


 Position        Repeat      %id   Spacer   Repeat_Sequence                         Spacer_Sequence
        Insertion/Deletion
==========       ======   ======   ======   =====================================   ==========================================
        ==================
   153388            37    100.0       35   .....................................   ATTGTGACTATCAGCGTCGCTATCTCCATCAATCC
   153460            37    100.0       38   .....................................   AAGGCACAGGATTACTTGGCCTTCGAAATAGAGGACTT
   153535            37    100.0       37   .....................................   CCCTTACTTTCGGTTCTGCTCATTATTCCTGGTTGGT
   153609            37    100.0       37   .....................................   CCCTTACTTTCGGTTCTGCTCATTATTCCTGGTTGGT
   153683            37    100.0       37   .....................................   CCCTTACTTTCGGTTCTGCTCATTATTCCTGGTTGGT
   153757            37    100.0       36   .....................................   CCCTTCACGTTATGCGGACAATGGCGTGAAAGGTCA
   153830            37    100.0       36   .....................................   GGCTAAACTGTACCTGGCCAGCACACCCACGGTAGA
   153903            37    100.0       37   .....................................   TTCGCTATAGTCGTTGGGGAGAATTGGGATATAGCGG
   153977            37    100.0       37   .....................................   TTCGCTATAGTCGTTGGGGAGAATTGGGATATAGCGG
   154051            37    100.0       37   .....................................   TTCGCTATAGTCGTTGGGGAGAATTGGGATATAGCGG
   154125            37    100.0       37   .....................................   TTCGCTATAGTCGTTGGGGAGAATTGGGATATAGCGG
   154199            37    100.0       37   .....................................   TTCGCTATAGTCGTTGGGGAGAATTGGGATATAGCGG
   154273            37    100.0       37   .....................................   TTCGCTATAGTCGTTGGGGAGAATTGGGATATAGCGG
   154347            37    100.0       37   .....................................   TTCGCTATAGTCGTTGGGGAGAATTGGGATATAGCGG
```

```
    154421           37    100.0       37    .....................................      TTCGCTATAGTCGTTGGGGAGAATTGGGATATAGCGG
    154495           37    100.0       37    .....................................      TTATTGCACGCTAATAACAACAATTCACAGAACGCCA
    154569           37    100.0       36    .....................................      TATTACGGAGGCCGATCTTACAGGGGCCGATCTTAC
    154642           37    100.0       37    .....................................      TTCATCAAACGGAGCTACTAACGAGCGTTCATAACCA
    154716           37    100.0       37    .....................................      TCCTACTTCTACTTGAAATAGATTCCAATATGTATTA
    154790           37    100.0       42    .....................................      ACTCGGAAAAGAGGATGAACGCCACCTCTGGCCCTTCATGCT
    154869           37    100.0        0    .....................................      |
==========       ======   ======   ======    =====================================      ==========================================
        ==================
        21           37    100.0       37    GTTTCAATCCATCTAATCTGGAAATTGGTATTCGAAG


  Position       Repeat      %id   Spacer   Repeat_Sequence                            Spacer_Sequence
        Insertion/Deletion
==========       ======   ======   ======    ====================================       ========================================
        ==================
         1           36    100.0       37    ....................................       AGGCTGAAATTGAAGCCGGAAATGACGACGCATTGGT
        74           36    100.0       39    ....................................       CTGATGATTGGTCGGCGTATGACGTGCTACTGAGGTGTT
       149           36    100.0       39    ....................................       CTGATGATTGGTCGGCGTATGACGTGCTACTGAGGTGTT
       224           36    100.0       39    ....................................       CTGATGATTGGTCGGCGTATGACGTGCTACTGAGGTGTT
       299           36    100.0       36    ....................................       TTGGTCAAAAGCTGTCGCCCAAGCATGAGGCAAAAA
       371           36    100.0       38    ....................................       GTGCAAGAATTCCGGGTTGCAGTGCAACACGGTTTTAA
       445           36    100.0       38    ....................................       GTGCAAGAATTCCGGGTTGCAGTGCAACACGGTTTTAA
       519           36    100.0        0    ....................................       |
==========       ======   ======   ======    ====================================       ========================================
        ==================
         8           36    100.0       38    GTTTCCGTCCCCTCTCGGGGTTTTGGGTCTGACGAC


#==========================================================================
# Crispr Rank in the sequence: 1
# Crispr_begin_position: 108281             Crispr_end_position: 110089
# DR: CGGTTTATCCCCGCTGGCGCGGGGAACAC         DR_length: 29    Number_of_spacers: 28
#==========================================================================
Spacer_begin_position      Spacer_length    Spacer_sequence
               108310                 32    TTTTCAGCCCTTGCCGACTGCGGAACGCCCCT

               108371                 32    ATGGATGGCTCATATCCGGTTTCTGTTTCTGC

               108432                 32    GCGAAATAGTGGGGAAAAACCCCTGGTTAACC

               108493                 32    TAGGCCTTGATACCATCGCTCGCACCTCGTCA

               108554                 32    TTCATATTCCAGAAAATGCCTGGGTGATGATC

               108615                 41    GTGANNNNNNNNNNNNNNNNNNNNNNNNNNNNNNNNNNNNN

               108685                 32    ATATTGATACTGTTCTCAAGCGCCGTTTTAGC

               108746                 32    TGGCGCCAGAAATATTCATGATCATCGGGATT

               108807                 32    TTCATATTCCAGAAAATGCCTGGGTGATGATC

               108868                 32    TTCATATTCCAGAAAATGCCTGGGTGATGATC

               108929                 72    GTGANNNNNNNNNNNNNNNNNNNNNNNNNNNNNNNNNNNNNNNNNNNNNNNNNNNNNNNNNNNNNNNTATGATC

               109030                 32    GTGGTATCTCCTGTCGACCTTGCCGTTAAAAT

               109091                 32    TATAACTGGCGGGTTTTAGTGTCGTTATAAAA

               109152                 32    TCAGGCTTGACGACAGTCTCAAGCCACTCCTG
```

```
            109213                32    GACCGCGCGGGCTATCGTTCAGGACTATTTTT

            109274                32    CTCTGTTACACCGTTATGCACAGACCACACAG

            109335                32    TGGAAGATATTGAAAGCGCCCAATCTTCCCAG

            109396                32    CTCTGTTACACCGTTATGCACGAACCACACAG

            109457                32    GTTAATGCAAGCGCCATTAACAAGAAAATGAC

            109518                32    CTCTGTTACACCGTTATGCACAGACCACACAG

            109579                67    AGCTCCGGCAGGGTCATGTCCCGGCAGGTGCGCGGTTTATCCCCGCTGATATGCACAGACCACACAG

            109675                32    AGCTCCGGCAGGGTCATGTCCCGGCAGGTGCG

            109736                32    AGCTCCGGCAGGGTCATGTCCCGGCAGGTGCG

            109797                32    GTTGTGACGAGTTCGTCGTCTTTTTTTTCGCT

            109858                32    AGCTCCGGCAGGGTCATGTCCCGGCAGGTGCG

            109919                32    AGCTCCGGCAGGGTCATGTCCCGGCAGGTGCG

            109980                32    AGCTCCGGCAGGGTCATGTCCCGGCAGGTGCG

            110041                19    TAAAGTGTCTGAAGGCTAC

#==========================================================================



#==========================================================================
# Crispr Rank in the sequence: 1
# Crispr_begin_position: 1          Crispr_end_position: 554
# DR: GTTTCCGTCCCCTCTCGGGGTTTTGGGTCTGACGAC  DR_length: 36    Number_of_spacers: 7
#==========================================================================
Spacer_begin_position      Spacer_length    Spacer_sequence
                    37                37    AGGCTGAAATTGAAGCCGGAAATGACGACGCATTGGT

                   110                39    CTGATGATTGGTCGGCGTATGACGTGCTACTGAGGTGTT

                   185                39    CTGATGATTGGTCGGCGTATGACGTGCTACTGAGGTGTT

                   260                39    CTGATGATTGGTCGGCGTATGACGTGCTACTGAGGTGTT

                   335                36    TTGGTCAAAAGCTGTCGCCCAAGCATGAGGCAAAAA

                   407                38    GTGCAAGAATTCCGGGTTGCAGTGCAACACGGTTTTAA

                   481                38    GTGCAAGAATTCCGGGTTGCAGTGCAACACGGTTTTAA

#==========================================================================
```

18 Distributions of arrays with different scores

Figure S7 Distribution of number of repeats in bona fide CRISPR arrays and CRISPR array looking structures obtained on the comprehensive dataset.

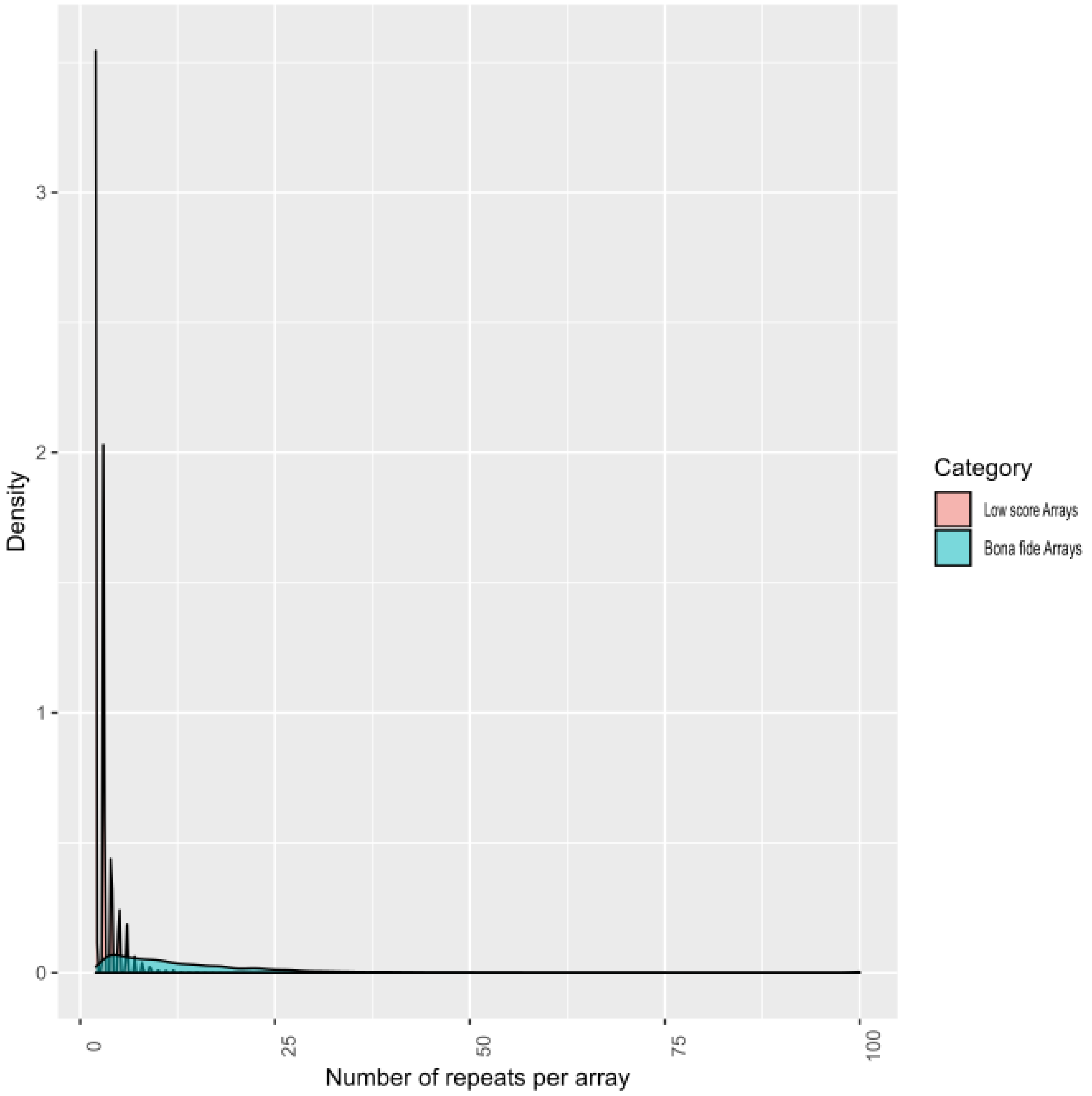

Figure S8 Distribution of repeat length in bona fide CRISPR arrays and CRISPR array looking structures obtained on the comprehensive dataset.

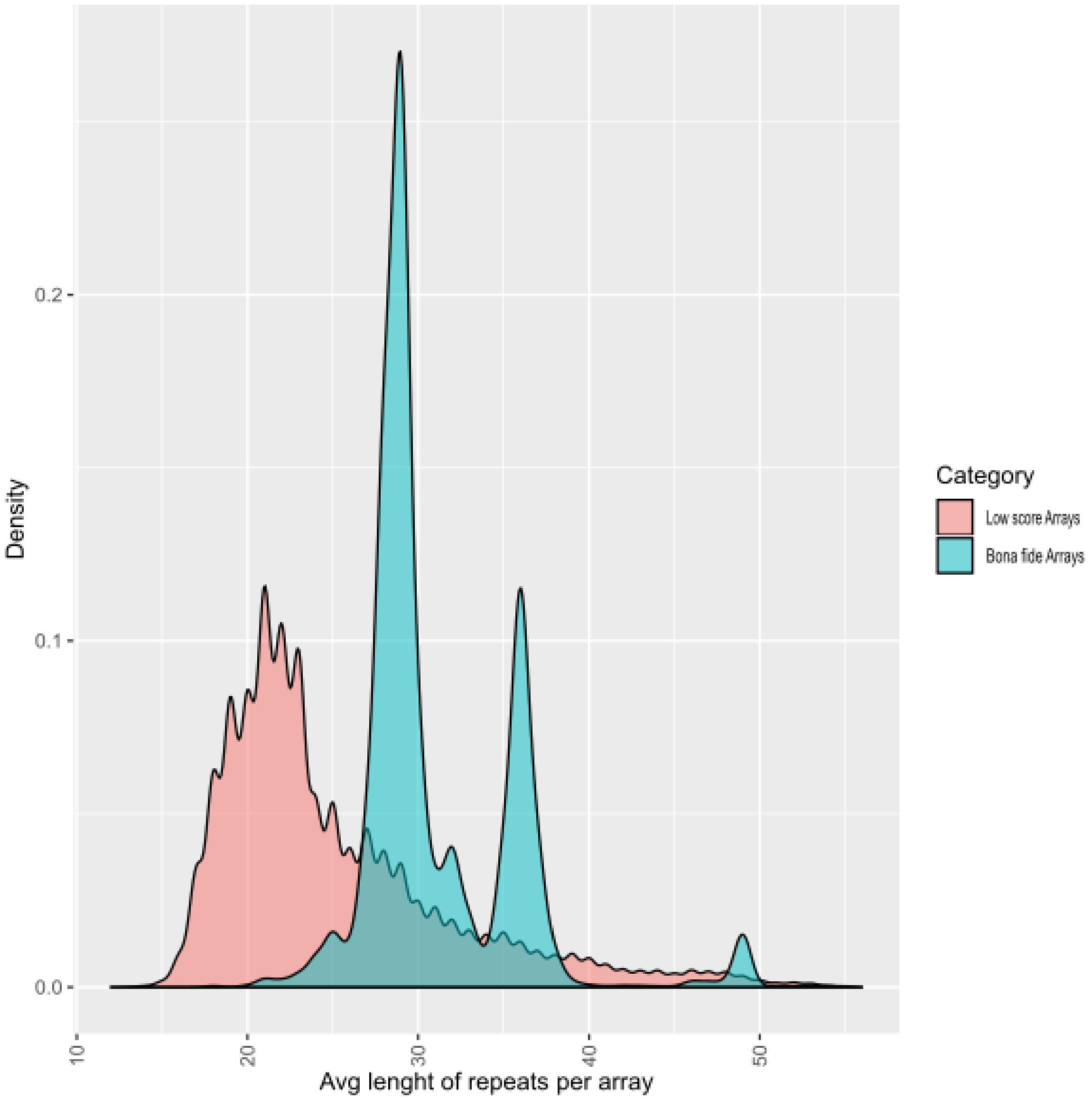

Figure S9 Distribution of spacer length in bona fide CRISPR arrays and CRISPR array looking structures obtained on the comprehensive dataset.

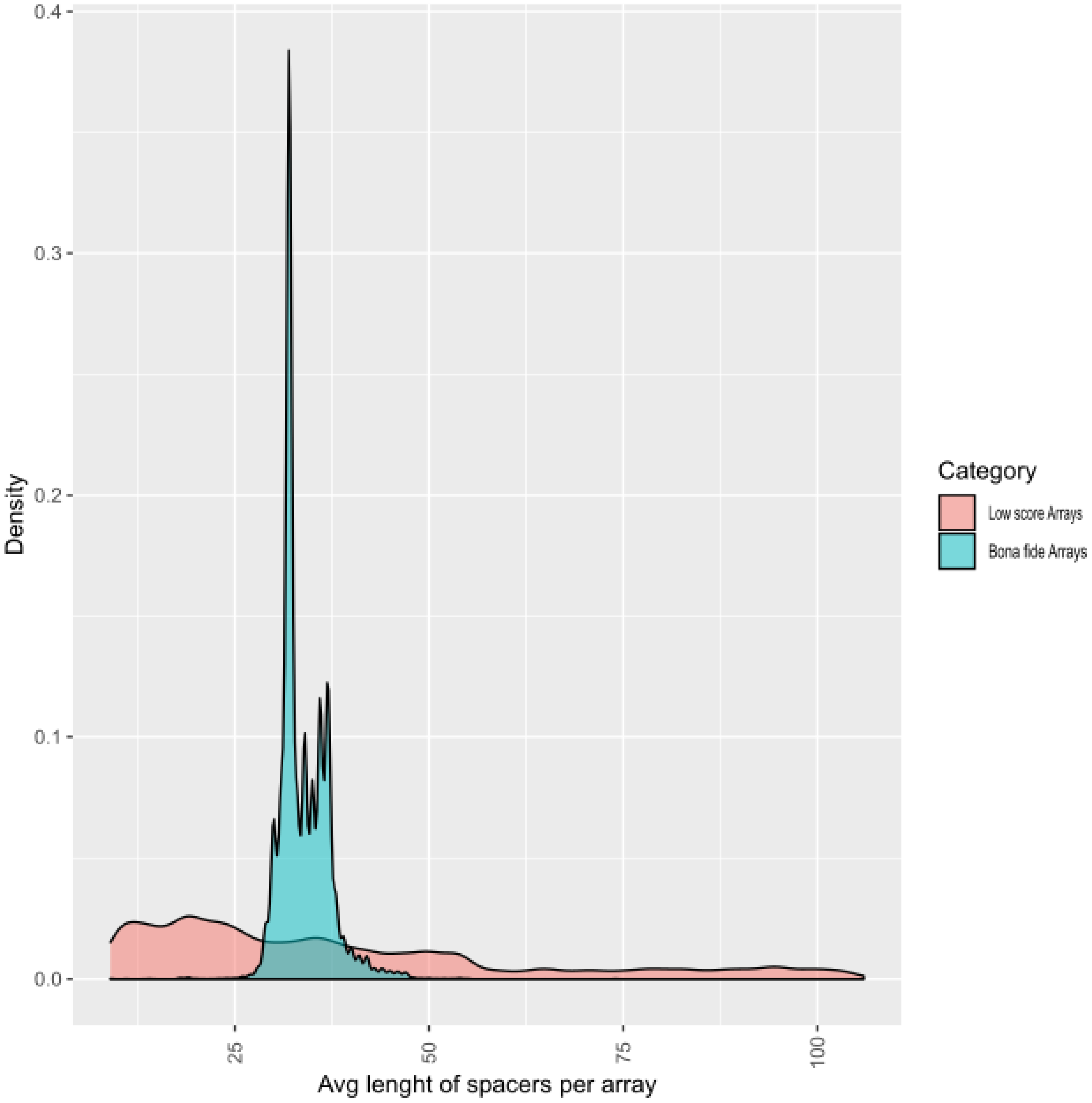

Figure S10 Distribution of repeat length in bona fide CRISPR arrays and CRISPR array looking structures obtained on the comprehensive dataset in Archaea.

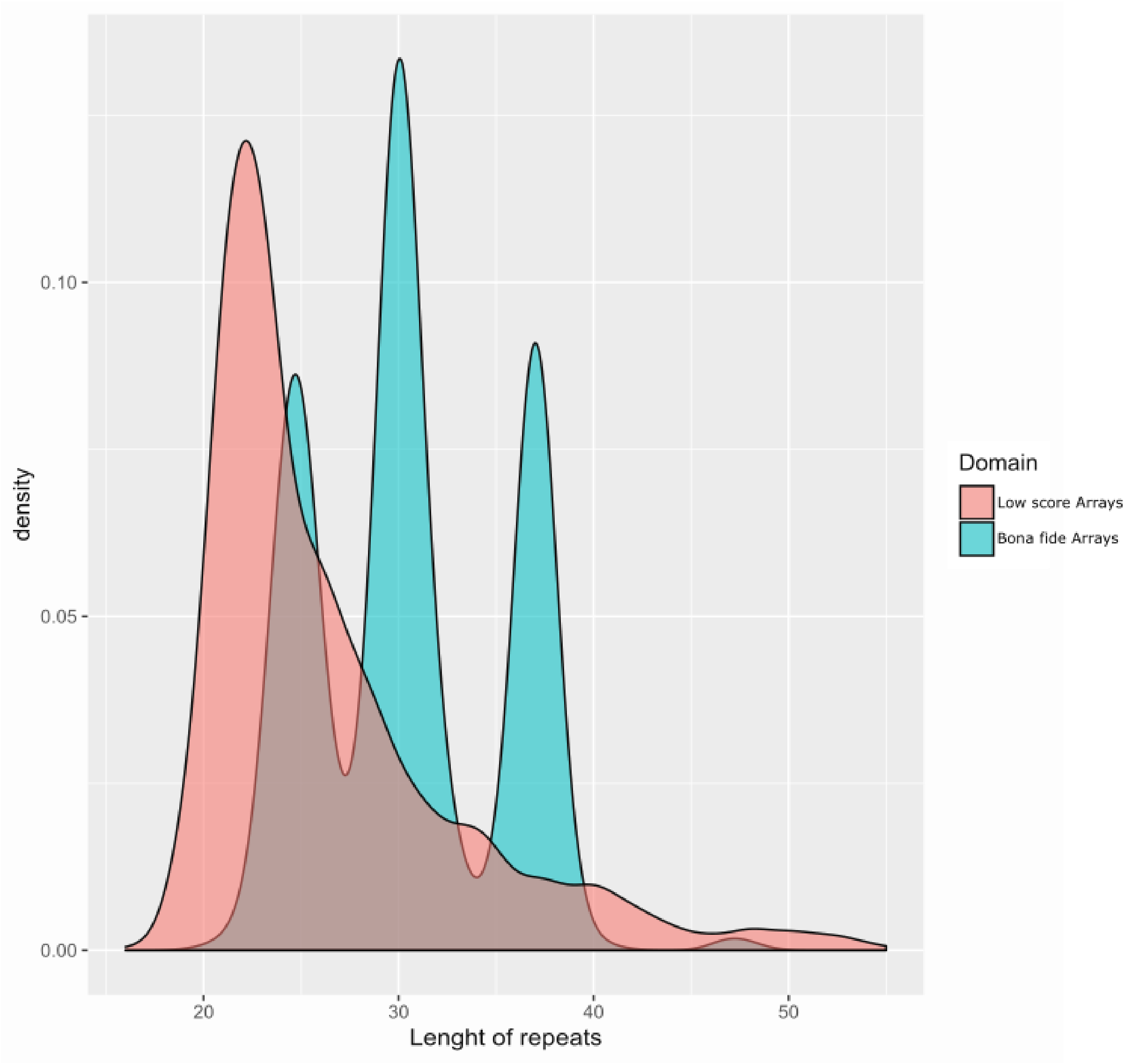

Figure S11 Distribution of repeat length in bona fide CRISPR arrays and CRISPR array looking structures obtained on the comprehensive dataset in Bacteria.

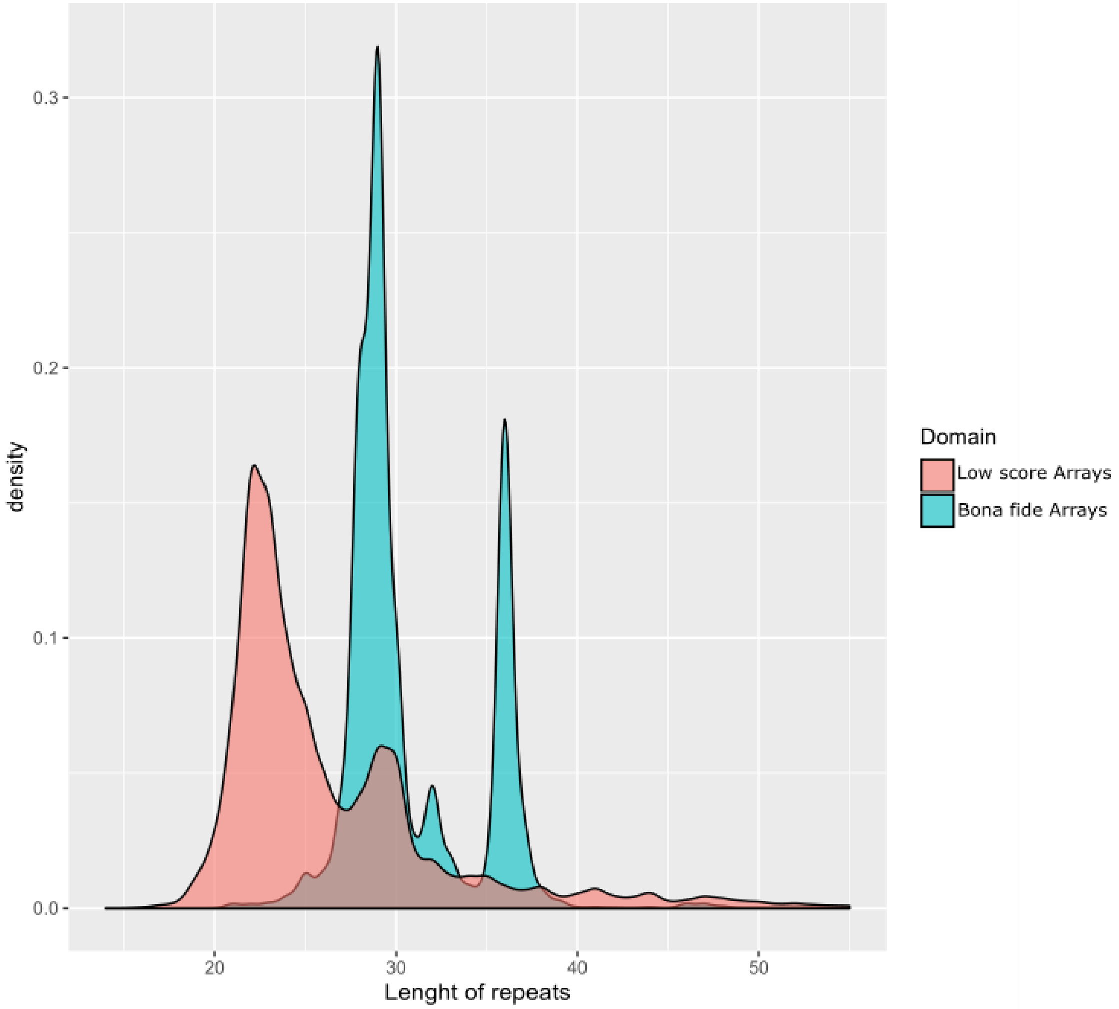

19 Miss-identification cases

Here we present the visual distribution of characteristics behind the false arrays described in Table S4 and reported by CRT, CRISPRCasFinder and CRISPRDetect.

Figure S12 Distribution of repeat length for false predicted CRISPR arrays

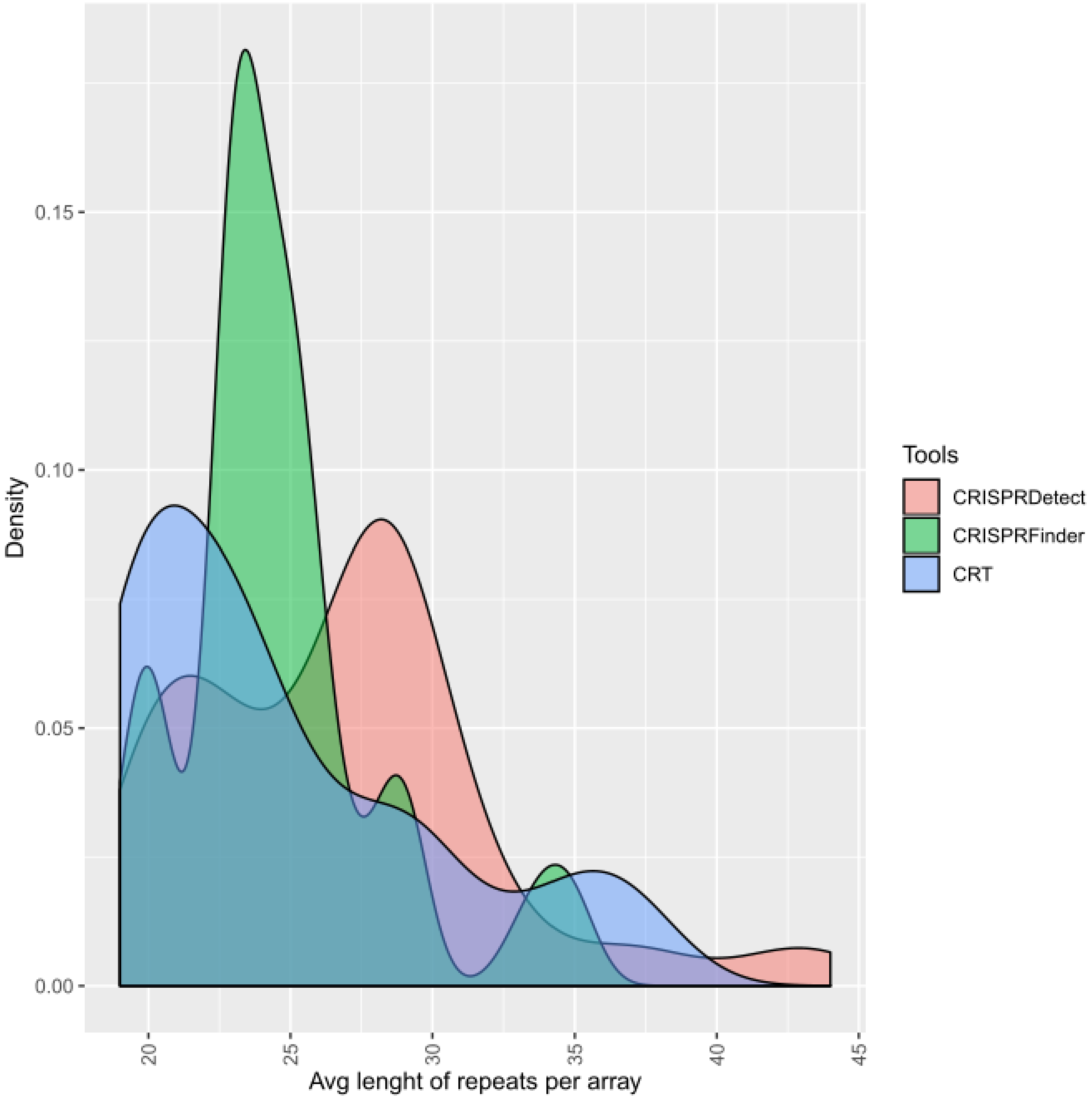

Figure S13 Distribution of number of repeats for false predicted CRISPR arrays

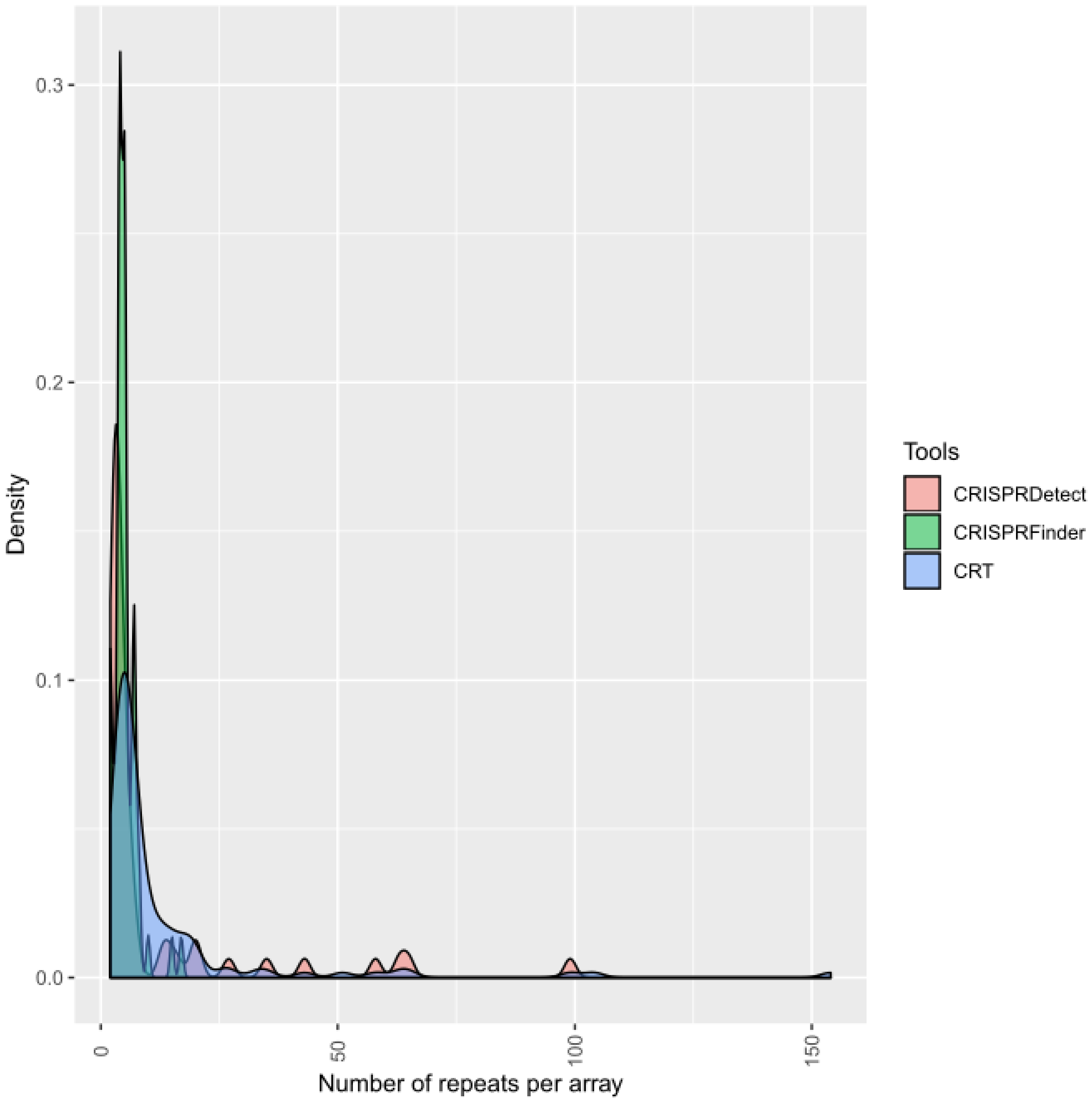

Figure S14 Distribution of repeats similarity for false predicted CRISPR arrays

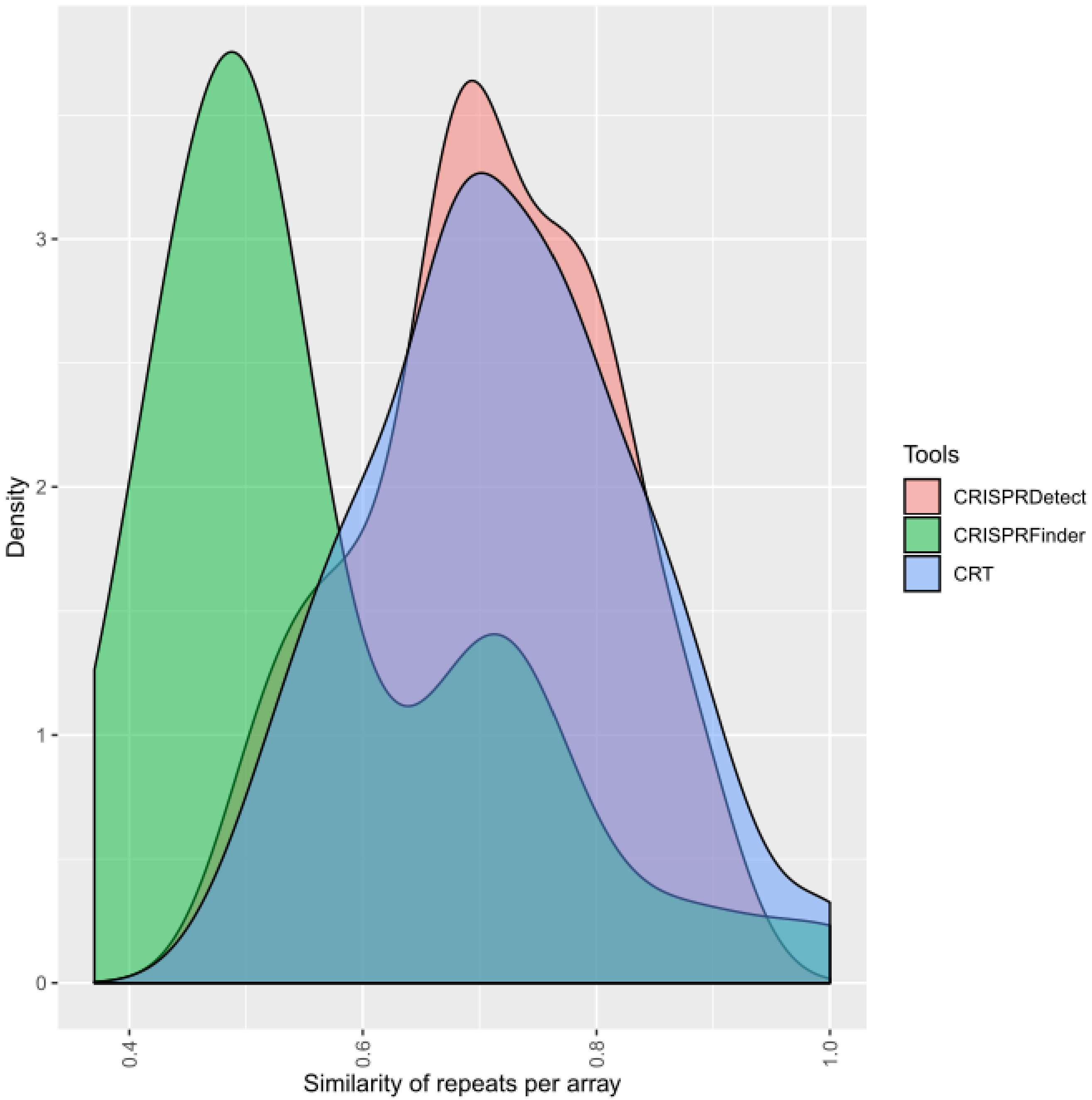

Figure S15 Distribution of spacers similarity for false predicted CRISPR arrays

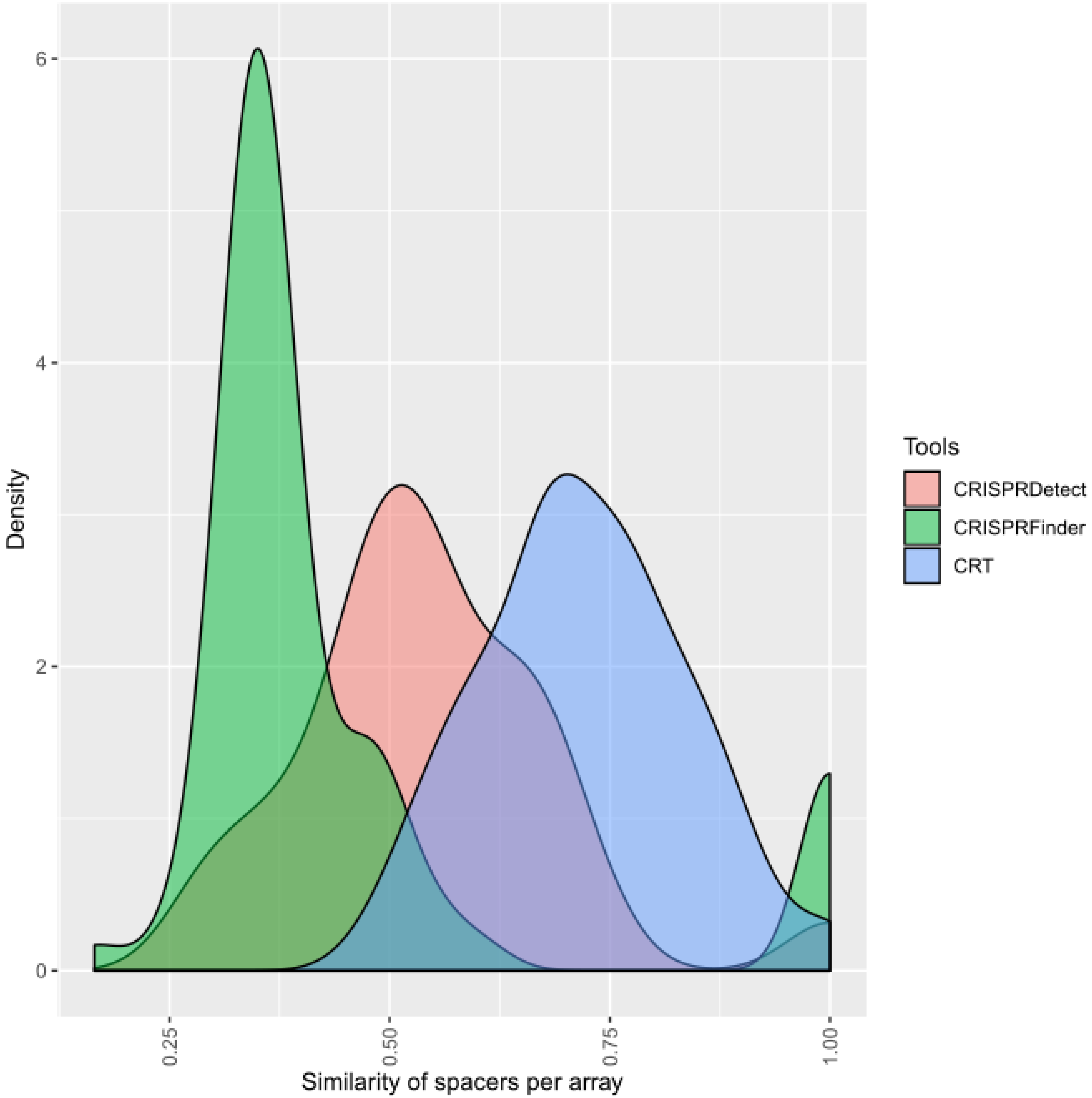

Figure S16 Distribution of spacers length for false predicted CRISPR arrays

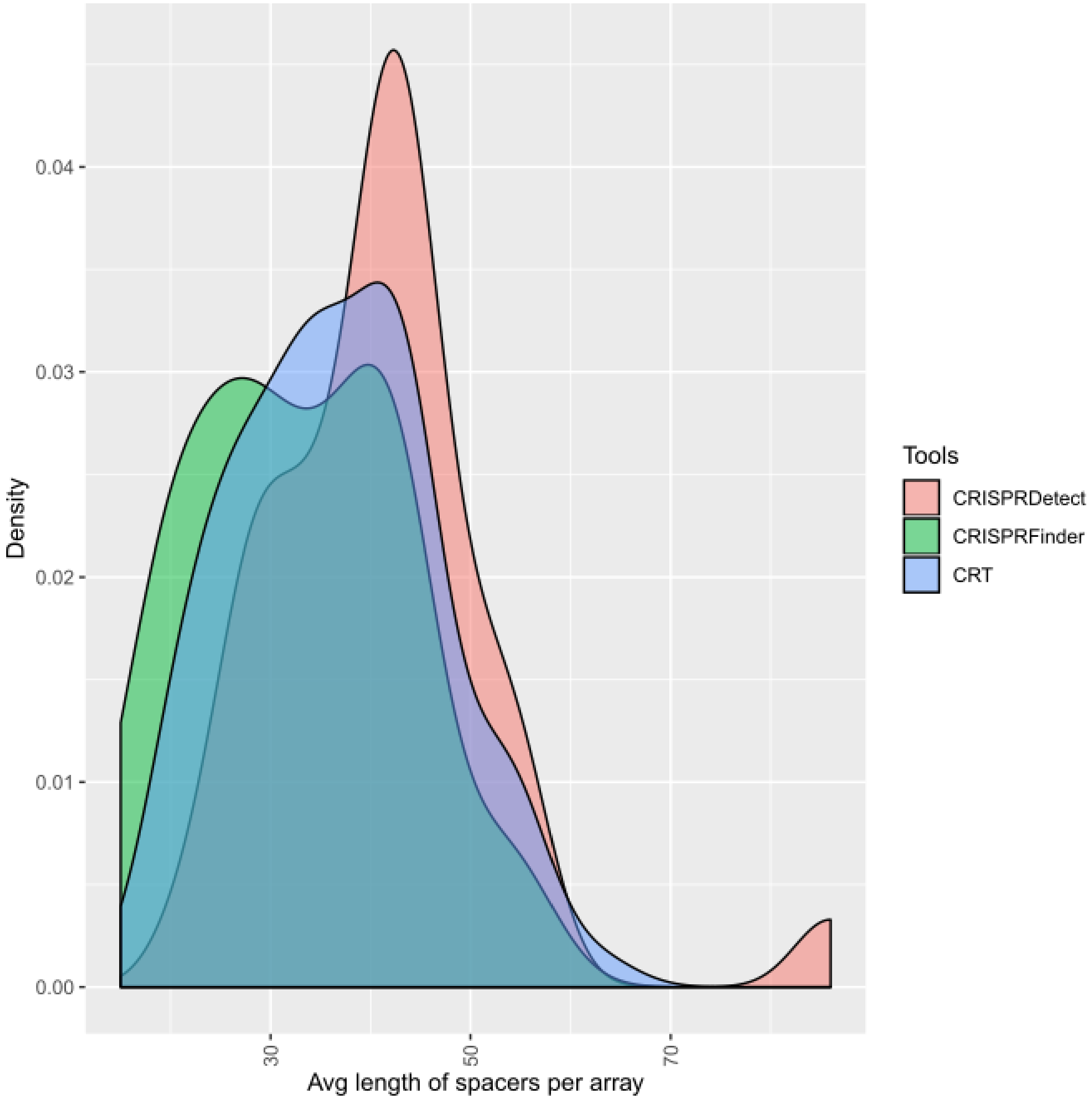

20 Array – Cas genes orientation

Here we studied how frequently the CRISPR arrays and the corresponding CRISPR-cas gene cassette share the same orientation. For that reason we took the database of reported by CRISPRidentify CRISPR arrays and complemented it with the CRISPR-cas gene cassettes information [6]. For each cassette we only took into account the closest CRISPR array. We also computed the orientation for each array using CRISPRStrand. We figured out that in 5204 cases the CRISP array and the corresponding CRISPR-cas gene cassette located on the same strand. And only in 1436 cases the direction was different.

References

[1] Siguier, P., Gourbeyre, E., and Chandler, M. (09, 2014) Bacterial insertion sequences: their genomic impact and diversity. *FEMS Microbiology Reviews,* **38**(5), 865–891.

[2] Siguier, P., Perochon, J., Lestrade, L., Mahillon, J., and Chandler, M. (01, 2006) ISfinder: the reference centre for bacterial insertion sequences. *Nucleic Acids Research,* **34**, D32–D36.

[3] Leplae, R., Lima-Mendez, G., and Toussaint, A. (11, 2009) ACLAME: A CLAssification of Mobile genetic Elements, update 2010. *Nucleic Acids Research,* **38**, D57–D61.

[4] Enright, A. J., van Dongen, S., and Ouzounis, C. A. (April, 2002) An Efficient Algorithm for Large-Scale Detection of Protein Families. *Nucleic Acids Research,* **30**(7), 1575–1584.

[5] Finn, R. D., Clements, J., and Eddy, S. R. (2011) HMMER web server: interactive sequence similarity searching. *Nucleic acids research,* **39**(suppl_2), W29–W37.

[6] Makarova, K. S., Wolf, Y. I., Iranzo, J., Shmakov, S., Alkhnbashi, O. S., Costa, F., Shah, S. A., Saunders, S. J., Barrangou, R., Brouns, S. J. J., Charpentier, E., Haft, D. H., Horvath, P., Moineau, S., Mojica, F. J. M., Terns, R. M., Terns, M. P., White, M. F., Yakunin, A. F., Garrett, R. A., van der Oost, J., Backofen, R., and Koonin, E. V. (November, 2019) Evolutionary classification of CRISPRCas systems: a burst of class 2 and derived variants. *Nature Reviews Microbiology,* **xx**(xx), xx–xx.

[7] Zhang, Q., & Ye, Y. (2017). Not all predicted CRISPR-Cas systems are equal: isolated cas genes and classes of CRISPR like elements. BMC bioinformatics, 18(1), 92.
