# Supplementary material for: CRISPRidentify: identification of CRISPR arrays using machine learning approach
Source: Nucleic Acids Res. 2020 Dec 8;49(4):e20. doi: 10.1093/nar/gkaa1158 (PMC7913763; doi:10.1093/nar/gkaa1158)
Supplement: gkaa1158_Supplemental_Files [file gkaa1158_supplemental_files.zip › CRISPRidentify_Suppl-2.pdf]

CRISPRidentify: Identification of CRISPR arrays using machine learning approach – Supplementary Material 2

Alexander Mitrofanov, Omer S Alkhnbashi, Sergey Shmakov, Kira M. Makarova, Eugene V. Koonin and Rolf Backofen

Table S5: Feature Encoding for 1300 Spurious Arrays in the CRISPRCasFinder dataset

Acc_number	Start	End	1	2	3	4	5	6	7	8	9	10	11	12	13	Tool
NC_022116	6451277	6451670	28	7	0.70	0.23	33.00	0.39	13.00	0.00	-13.40	0.00	0.00	15.90	15.90	CRT
NC_011761	934674	934937	31	5	0.69	0.30	27.25	0.28	12.00	0.31	-10.40	0.00	0.00	14.00	15.90	CRT
NC_009776	915468	915892	26	7	0.71	0.55	40.50	0.30	11.00	0.00	-3.10	0.00	0.00	19.60	23.30	CRT
NC_009776	952490	952852	26	6	0.77	0.54	41.40	0.32	7.00	0.00	-2.20	0.00	0.00	21.40	27.00	CRT
NC_016640	5115436	5115593	23	4	0.74	0.49	22.00	0.31	7.00	0.00	-3.50	0.00	0.00	12.20	14.00	CRT
NC_009515	1177354	1177916	29	8	0.58	0.71	47.29	0.50	31.00	0.00	-3.20	0.00	0.00	17.70	17.70	CRT
NC_013887	926377	926684	31	5	0.77	0.62	38.25	0.32	8.00	0.39	-3.50	0.00	0.00	19.60	25.10	CRT
NC_017584	994969	995804	35	13	0.68	0.36	31.75	0.44	34.00	0.16	-7.30	0.00	0.00	14.00	15.90	CRT
NZ_CP014232	625014	625414	37	6	0.80	0.49	35.80	0.32	11.00	0.28	-8.70	0.00	0.00	12.20	15.90	CRT
NZ_CP014232	625744	626575	37	12	0.77	0.49	35.27	0.34	18.00	0.17	-8.70	0.00	0.00	12.20	15.90	CRT
NZ_CP014232	639681	641010	24	19	0.78	0.47	48.56	0.44	16.00	0.12	-0.70	0.00	0.00	12.20	0.00	CRT
NZ_CP014232	1225367	1225878	24	9	0.73	0.24	37.00	0.39	11.00	0.00	-13.00	0.00	0.00	19.60	19.60	CRT
NC_015562	738969	739414	23	7	0.79	0.73	47.50	0.36	11.00	0.25	-5.60	0.00	0.00	30.70	30.70	CRT
NC_015562	1260296	1260641	37	5	0.79	0.67	40.25	0.30	10.00	0.31	-5.60	0.00	0.00	32.50	32.50	CRT
NC_015562	1515368	1515804	30	7	0.72	0.60	37.83	0.36	29.00	0.36	-3.90	0.00	0.00	25.10	27.00	CRT
NC_017551	3161011	3161331	36	5	0.74	0.56	35.25	0.27	17.00	0.00	-9.30	0.00	0.00	15.90	0.00	CRT
NC_017551	3162190	3162512	36	5	0.73	0.54	35.75	0.35	15.00	0.31	-9.30	0.00	0.00	15.90	0.00	CRT
NC_013131	4438676	4439802	29	19	0.75	0.21	32.00	0.33	32.00	0.00	-12.90	0.00	0.00	23.30	23.30	CRT
NC_013131	4441939	4442333	29	7	0.61	0.25	32.00	0.34	15.00	0.00	-12.90	0.00	0.00	27.00	27.00	CRT
NC_013131	9339631	9339791	26	4	0.70	0.34	19.00	0.59	10.00	0.00	-5.10	0.00	-2.70	0.00	15.90	CRT
NC_014720	2788771	2789325	30	9	0.76	0.50	35.63	0.30	12.00	0.21	-5.60	0.00	0.00	19.60	28.80	CRT
NC_013716	3224982	3225436	29	8	0.69	0.35	31.86	0.32	18.00	0.00	-15.10	0.00	0.00	47.30	47.30	CRT
NC_018224	305689	305981	32	5	0.78	0.48	33.25	0.33	7.00	0.00	-10.20	0.00	0.00	39.90	39.90	CRT
NC_015711	2109248	2109933	25	13	0.55	0.31	30.08	0.56	36.00	0.16	-3.70	0.00	0.00	15.90	15.90	CRT
NC_015711	5327173	5327393	35	4	0.65	0.33	27.00	0.39	13.00	0.36	-9.00	0.00	0.00	12.20	0.00	CRT
NC_015850	1234131	1235254	28	19	0.77	0.27	32.89	0.31	33.00	0.00	-13.10	0.00	0.00	21.40	21.40	CRT
NC_015850	1236314	1236999	28	12	0.75	0.27	31.82	0.30	21.00	0.00	-13.10	73.67	0.00	19.60	19.60	CRT
NC_018524	313188	313459	28	5	0.76	0.23	33.00	0.31	6.00	0.00	-14.30	0.00	0.00	25.10	25.10	CRT
NC_018524	2251001	2251573	23	10	0.76	0.23	38.11	0.39	13.00	0.00	-8.10	0.00	0.00	12.20	15.90	CRT
NC_018524	2270308	2271179	25	15	0.66	0.25	35.50	0.36	30.00	0.00	-14.30	85.38	0.00	25.10	25.10	CRT
NC_018524	4408931	4409144	29	4	0.79	0.25	32.67	0.34	3.00	0.00	-12.70	0.00	0.00	12.20	15.90	CRT
NC_018524	4593160	4593371	29	4	0.79	0.20	32.00	0.35	5.00	0.00	-14.30	0.00	0.00	25.10	25.10	CRT
NC_014205	716213	716498	25	5	0.80	0.68	40.25	0.35	6.00	0.00	0.00	0.00	0.00	27.00	27.00	CRT
NC_020449	226242	226799	30	9	0.75	0.62	36.00	0.53	14.00	0.00	-4.50	0.00	0.00	14.00	14.00	CRT
NC_021287	507941	508761	28	14	0.69	0.33	33.00	0.33	30.00	0.00	-11.40	0.00	0.00	12.20	14.00	CRT
NC_007413	2727350	2727599	37	4	0.78	0.47	34.00	0.34	10.00	0.00	-7.40	0.00	0.00	15.90	15.90	CRT
NC_010516	1031479	1031901	28	7	0.77	0.81	37.83	0.38	7.00	0.25	-1.70	0.00	0.00	14.00	14.00	CRT
NC_010516	2308515	2308939	30	7	0.67	0.77	35.83	0.36	18.00	0.25	0.00	0.00	0.00	14.00	14.00	CRT
NC_019757	4384963	4385214	28	4	0.67	0.44	46.67	0.42	14.00	0.00	-11.30	0.00	0.00	21.40	21.40	CRT
NC_019757	4570699	4571580	24	14	0.73	0.71	42.00	0.61	22.00	0.00	0.00	0.00	0.00	14.00	14.00	CRT
NC_014374	1496055	1496411	24	6	0.68	0.58	42.60	0.32	15.00	0.28	-3.20	0.00	0.00	17.70	17.70	CRT
NZ_CP007493	1353975	1354198	26	4	0.78	0.57	40.00	0.31	10.00	0.00	-4.30	0.00	0.00	23.30	25.10	CRT

NC_018227	2286076	2286564	27	8	0.70	0.44	39.00	0.46	15.00	0.00	-2.70	0.00	0.00	12.20	14.00	CRT
NZ_CP014688	337638	338215	29	10	0.62	0.35	32.00	0.29	27.00	0.00	-12.90	0.00	0.00	27.00	27.00	CRT
NZ_CP018139	3485490	3485817	23	6	0.72	0.25	38.00	0.36	7.00	0.00	-8.50	0.00	0.00	32.50	32.50	CRT
NZ_CP014214	1153895	1155996	29	35	0.71	0.41	31.97	0.30	80.00	0.00	-12.80	0.00	0.00	27.00	27.00	CRT
NC_010085	405794	407058	29	23	0.68	0.54	27.18	0.57	96.00	0.38	-1.30	0.00	0.00	17.70	17.70	CRT
NC_018870	2447887	2448107	33	4	0.79	0.57	29.67	0.28	14.00	0.36	-2.50	0.00	0.00	45.40	60.20	CRT
NC_000909	1049373	1050302	30	14	0.77	0.66	39.23	0.35	38.00	0.44	-3.70	0.00	0.00	49.10	49.10	CRT
NC_000909	1456715	1457335	30	9	0.78	0.62	43.88	0.40	25.00	0.00	-3.80	0.00	0.00	25.10	27.00	CRT
NC_000909	1570374	1570895	30	8	0.78	0.61	40.29	0.37	22.00	0.23	-4.10	0.00	0.00	25.10	27.00	CRT
NZ_CP012159	11283469	11283808	25	8	0.70	0.35	20.00	0.39	19.00	0.00	-3.50	0.00	1.00	14.00	14.00	CRT
NC_012032	5195607	5195974	32	6	0.74	0.20	35.20	0.49	10.00	0.28	-13.80	0.00	0.00	15.90	15.90	CRT
NC_019771	4586460	4587628	29	15	0.56	0.53	52.43	0.61	93.00	0.14	-2.10	0.00	0.00	14.00	14.00	CRT
NC_014656	4051	4445	29	7	0.73	0.29	32.00	0.31	14.00	0.00	-13.20	0.00	0.00	21.40	21.40	CRT
NC_000918	156460	156768	31	5	0.75	0.57	38.50	0.36	18.00	0.39	-3.70	0.00	0.00	17.70	19.60	CRT
NC_000918	1226626	1226861	31	4	0.80	0.59	37.33	0.30	11.00	0.36	-4.20	0.00	0.00	23.30	25.10	CRT
NC_021658	5329135	5329478	26	8	0.62	0.27	19.43	0.42	20.00	0.00	-3.90	0.00	0.00	12.20	0.00	CRT
NZ_CP009503	1978762	1980014	35	19	0.76	0.68	32.67	0.61	31.00	0.00	-4.90	0.00	0.00	19.60	19.60	CRT
NC_021044	3437922	3438683	27	13	0.58	0.63	34.25	0.37	47.00	0.16	-1.10	99.57	0.00	15.90	15.90	CRT
NC_013407	1372554	1373277	29	11	0.71	0.63	40.50	0.35	44.00	0.18	-3.70	0.00	0.00	21.40	21.40	CRT
NC_019695	3355660	3356061	34	6	0.71	0.65	39.60	0.31	23.00	0.00	-5.50	0.00	0.00	17.70	28.80	CRT
NC_019697	5813408	5813854	30	7	0.74	0.57	39.50	0.51	13.00	0.00	-3.70	0.00	0.00	19.60	19.60	CRT
NC_007426	165989	166281	30	5	0.79	0.47	35.75	0.33	13.00	0.31	-8.50	0.00	0.00	56.50	56.50	CRT
NZ_CP012268	3202305	3202752	28	8	0.79	0.45	32.00	0.30	10.00	0.00	-9.10	0.00	0.00	41.70	41.70	CRT
NC_019776	3183041	3183587	37	8	0.77	0.72	35.86	0.29	28.00	0.38	-3.50	0.00	0.00	27.00	38.10	CRT
NC_013715	1376010	1376758	27	11	0.79	0.41	45.20	0.40	17.00	0.00	-2.40	0.00	0.00	14.00	14.00	CRT
NC_013715	1380329	1382396	34	29	0.79	0.53	38.64	0.32	63.00	0.19	-9.00	0.00	0.00	17.70	17.70	CRT
NC_013922	1028405	1028630	34	4	0.78	0.38	30.00	0.57	11.00	0.61	-0.70	0.00	-4.20	14.00	0.00	CRT
NC_013922	1647603	1649414	30	28	0.69	0.49	36.00	0.32	61.00	0.36	-4.70	0.00	0.00	30.70	30.70	CRT
NZ_CP016077	4342575	4342786	29	4	0.75	0.23	32.00	0.35	5.00	0.00	-13.80	0.00	0.00	25.10	25.10	CRT
NZ_CP014859	1316441	1316647	28	4	0.75	0.24	31.67	0.33	10.00	0.00	-13.00	0.00	0.00	19.60	19.60	CRT
NZ_CP011350	697170	697533	34	6	0.67	0.58	32.00	0.35	16.00	0.00	-11.40	0.00	0.00	25.10	30.70	CRT
NC_017030	859639	860354	27	11	0.69	0.43	41.90	0.48	32.00	0.18	-3.70	0.00	0.00	14.00	14.00	CRT
NC_017030	2120434	2121357	31	14	0.69	0.42	37.69	0.45	45.00	0.24	-13.70	0.00	0.00	12.20	0.00	CRT
NC_017030	6372838	6373070	23	5	0.62	0.17	29.50	0.36	8.00	0.58	-4.20	0.00	0.00	14.00	15.90	CRT
NC_017030	6376293	6376717	26	6	0.72	0.30	53.80	0.61	19.00	0.38	-7.60	0.00	11.70	12.20	0.00	CRT
NC_017030	7428733	7429047	35	5	0.63	0.29	35.00	0.42	21.00	0.31	-13.70	0.00	0.00	15.90	15.90	CRT
NC_017030	7950265	7950740	26	7	0.53	0.31	49.00	0.56	21.00	0.39	-4.00	0.00	0.00	17.70	17.70	CRT
NC_017030	9234355	9234571	24	4	0.73	0.42	40.33	0.56	8.00	0.36	-5.10	0.00	0.00	0.00	0.00	CRT
NC_021009	711854	712221	29	6	0.72	0.57	38.80	0.34	12.00	0.38	0.00	0.00	0.00	30.70	54.70	CRT
NC_015151	1482074	1482364	24	5	0.77	0.79	42.75	0.33	8.00	0.00	-2.50	0.00	0.00	17.70	34.40	CRT
NC_015573	488724	489023	30	5	0.77	0.55	37.50	0.29	15.00	0.39	-5.60	0.00	0.00	32.50	56.50	CRT
NC_015573	806823	807245	27	7	0.63	0.59	39.00	0.33	13.00	0.25	-2.60	0.00	0.00	27.00	34.40	CRT
NC_015573	811112	814237	30	47	0.65	0.61	37.30	0.32	103.00	0.38	-4.80	83.64	0.00	27.00	34.40	CRT
NC_015573	2965685	2966083	32	6	0.78	0.43	41.40	0.38	9.00	0.00	-4.30	0.00	0.00	27.00	27.00	CRT
NC_016109	7412627	7412864	25	5	0.77	0.09	28.25	0.39	7.00	0.58	-8.30	0.00	0.00	17.70	17.70	CRT
NZ_CP006965	954069	957347	29	49	0.75	0.77	38.71	0.37	177.00	0.00	-2.30	0.00	0.00	27.00	43.60	CRT
NC_009033	394513	394921	25	7	0.78	0.70	39.00	0.31	12.00	0.25	0.00	0.00	0.00	21.40	25.10	CRT

NC_021191	7625213	7625564	24	7	0.72	0.54	30.67	0.46	11.00	0.56	-1.20	0.00	0.00	14.00	14.00	CRT
NZ_CP015435	2507434	2508131	23	11	0.67	0.27	44.50	0.70	19.00	0.71	-2.90	0.00	0.00	12.20	0.00	CRT
NC_014122	109860	110089	30	4	0.80	0.58	36.67	0.34	9.00	0.36	-6.20	0.00	0.00	15.90	21.40	CRT
NC_010175	5185240	5185607	32	6	0.74	0.20	35.20	0.49	10.00	0.28	-13.80	0.00	0.00	15.90	15.90	CRT
NC_012880	3904535	3904749	31	4	0.79	0.38	30.33	0.28	12.00	0.00	-12.80	0.00	0.00	32.50	32.50	CRT
NC_014222	681099	681401	24	5	0.60	0.88	45.75	0.47	13.00	0.00	0.00	0.00	0.00	17.70	17.70	CRT
NC_017527	1553121	1553389	29	6	0.69	0.44	19.00	0.45	14.00	0.00	-2.50	0.00	0.00	14.00	14.00	CRT
NC_019429	39846	40243	37	6	0.73	0.64	35.20	0.31	25.00	0.28	-7.20	0.00	0.00	23.30	34.40	CRT
NZ_CP016463	264278	264732	28	8	0.75	0.28	33.00	0.36	14.00	0.00	-14.00	0.00	0.00	14.00	17.70	CRT
NC_013315	1712097	1712386	29	5	0.79	0.81	36.25	0.34	7.00	0.39	-4.00	0.00	0.00	36.20	36.20	CRT
NC_011529	828034	828603	27	9	0.73	0.59	40.88	0.31	23.00	0.00	-1.10	53.66	0.00	14.00	14.00	CRT
NC_014734	223908	225657	36	27	0.79	0.62	29.92	0.31	34.00	0.00	-5.20	94.61	0.00	21.40	21.40	CRT
NC_006582	3820899	3821324	32	7	0.78	0.53	33.67	0.35	11.00	0.00	-11.50	0.00	0.00	25.10	34.40	CRT
NZ_CP016784	2229654	2230019	32	6	0.72	0.55	34.80	0.34	12.00	0.00	-11.50	67.37	0.00	25.10	34.40	CRT
NZ_CP012196	1361763	1361982	23	4	0.76	0.55	42.67	0.34	6.00	0.00	-3.90	0.00	0.00	21.40	21.40	CRT
NC_013790	433656	434459	24	11	0.61	0.68	54.00	0.52	24.00	0.00	0.00	0.00	0.00	14.00	14.00	CRT
NC_015672	1709894	1710225	36	5	0.78	0.60	38.00	0.33	13.00	0.00	-1.10	0.00	0.00	14.00	15.90	CRT
NC_021149	1707403	1707674	29	5	0.70	0.28	31.75	0.30	9.00	0.00	-14.30	0.00	0.00	28.80	28.80	CRT
NZ_CP019041	673834	674127	24	5	0.67	0.42	43.50	0.53	6.00	0.58	-5.80	0.00	0.00	12.20	0.00	CRT
NC_019962	117407	117691	30	4	0.79	0.22	55.00	0.76	10.00	0.00	-7.20	0.00	0.00	14.00	14.00	CRT
NZ_CP012898	2754514	2754782	26	4	0.62	0.50	55.00	0.48	10.00	0.00	0.00	0.00	0.00	0.00	15.90	CRT
NC_013757	470126	470286	26	4	0.65	0.22	19.00	0.82	11.00	0.00	-6.70	0.00	5.20	12.20	0.00	CRT
NC_013757	1755147	1755475	29	5	0.63	0.15	46.00	0.40	18.00	0.58	-11.70	0.00	0.00	12.20	14.00	CRT
NC_013757	2230306	2230610	27	4	0.63	0.20	65.67	0.51	18.00	0.36	-4.60	0.00	0.00	12.20	14.00	CRT
NC_013757	2651394	2651836	23	7	0.76	0.08	47.00	0.48	9.00	0.56	-8.60	0.00	-6.20	14.00	14.00	CRT
NC_013757	2717404	2718032	32	10	0.71	0.33	34.33	0.71	35.00	0.52	-7.60	0.00	0.10	17.70	17.70	CRT
NC_009925	1968155	1968460	30	5	0.79	0.49	39.00	0.63	5.00	0.00	0.00	0.00	0.00	14.00	14.00	CRT
NC_009925	2784991	2785436	26	9	0.70	0.29	26.50	0.62	16.00	0.50	-5.20	100.00	0.00	12.20	0.00	CRT
NC_009925	3113974	3114371	23	8	0.63	0.36	30.57	0.67	19.00	0.38	-4.70	0.00	0.00	14.00	15.90	CRT
NC_009792	2401036	2401286	37	4	0.62	0.36	34.33	0.64	41.00	0.36	-19.10	0.00	0.00	12.20	0.00	CRT
NC_009792	3347776	3348038	37	5	0.72	0.46	19.50	0.88	20.00	0.00	-7.60	0.00	0.00	14.00	0.00	CRT
NC_009792	3568889	3569585	32	9	0.77	0.25	51.13	0.77	26.00	0.00	-17.50	72.07	0.00	15.90	15.90	CRT
NC_009792	3901376	3901552	30	4	0.72	0.48	19.00	0.73	14.00	0.00	-5.60	0.00	0.00	12.20	0.00	CRT
NC_009792	4116007	4116231	29	5	0.68	0.40	20.00	0.73	19.00	0.00	-5.60	0.00	0.00	12.20	17.70	CRT
NC_014472	847	1235	35	5	0.77	0.45	53.50	0.57	7.00	0.39	-6.40	0.00	0.00	0.00	0.00	CRT
NC_014472	112363	115797	33	40	0.64	0.42	54.23	0.72	112.00	0.48	-7.70	99.70	-52.30	12.20	0.00	CRT
NC_014472	118292	119070	23	10	0.51	0.44	61.00	0.64	26.00	0.59	-2.90	0.00	0.00	12.20	0.00	CRT
NC_014472	1860380	1860854	28	7	0.60	0.62	46.50	0.42	19.00	0.00	-3.30	0.00	0.00	14.00	15.90	CRT
NC_014472	3239045	3239250	26	5	0.75	0.55	19.00	0.86	6.00	0.00	-1.40	95.30	0.00	12.20	14.00	CRT
NC_008611	723056	723354	25	5	0.49	0.20	43.50	0.36	21.00	0.31	-7.00	0.00	0.00	12.20	0.00	CRT
NC_008611	1689782	1690013	25	5	0.47	0.33	26.75	0.37	23.00	0.31	-7.00	0.00	0.00	12.20	0.00	CRT
NC_008611	5030987	5031379	33	7	0.54	0.37	27.00	0.41	39.00	0.00	-6.20	0.00	0.00	14.00	0.00	CRT
NC_014330	1793735	1793967	26	5	0.68	0.78	25.75	0.55	15.00	0.31	0.00	0.00	0.00	17.70	19.60	CRT
NC_015312	349721	350815	30	15	0.69	0.19	46.07	0.73	54.00	0.59	-11.10	0.00	-36.10	14.00	14.00	CRT
NC_015312	2011360	2011614	24	4	0.79	0.29	53.00	0.97	7.00	0.00	-0.60	0.00	0.00	12.20	15.90	CRT
NC_015312	2146119	2146448	23	6	0.46	0.22	38.40	0.53	24.00	0.28	-11.20	0.00	0.00	14.00	14.00	CRT
NC_015312	2262291	2262451	26	4	0.70	0.29	19.00	0.69	6.00	0.00	-14.30	0.00	0.00	12.20	0.00	CRT

NC_015312	2467122	2467270	23	4	0.69	0.39	19.00	0.59	6.00	0.00	-1.40	0.00	11.00	14.00	14.00	CRT
NC_015312	2971850	2972068	27	4	0.71	0.24	37.00	0.64	5.00	0.36	-4.00	0.00	0.00	12.20	0.00	CRT
NC_015312	5056346	5056778	24	7	0.59	0.11	44.17	0.65	20.00	0.74	-12.40	0.00	0.00	14.00	14.00	CRT
NC_013729	3468121	3468812	23	13	0.71	0.23	32.75	0.60	23.00	0.69	-2.80	0.00	0.00	15.90	15.90	CRT
NC_013729	3469131	3469381	35	5	0.67	0.35	19.00	1.00	15.00	0.00	-6.90	0.00	0.00	14.00	17.70	CRT
NC_013729	5229025	5229233	38	4	0.75	0.24	19.00	0.52	13.00	0.00	-15.50	0.00	-4.60	12.20	0.00	CRT
NC_013729	7032337	7032827	23	8	0.72	0.22	43.86	0.75	13.00	0.53	-5.80	0.00	0.00	0.00	0.00	CRT
NZ_CP007567	1175464	1175734	31	4	0.77	0.39	49.00	0.85	8.00	0.00	-9.00	0.00	0.00	12.20	14.00	CRT
NZ_CP007567	3872810	3873080	25	5	0.64	0.35	36.50	0.60	10.00	0.31	-3.60	0.00	0.00	12.20	0.00	CRT
NZ_CP007567	4728720	4728931	29	4	0.78	0.31	32.00	0.29	6.00	0.00	-11.70	0.00	0.00	17.70	17.70	CRT
NZ_CP019701	2197783	2198013	32	4	0.76	0.38	34.33	0.30	6.00	0.00	-13.30	0.00	0.00	30.70	36.20	CRT
NZ_CP009110	1159813	1160008	23	5	0.64	0.20	20.25	0.40	13.00	0.00	-6.60	0.00	0.00	14.00	14.00	CRT
NZ_CP009110	1427439	1427694	26	4	0.77	0.22	50.67	0.56	15.00	0.36	-9.30	0.00	0.00	17.70	17.70	CRT
NZ_CP009110	1570502	1570975	24	7	0.74	0.24	51.00	0.85	9.00	0.00	-6.90	0.00	0.00	12.20	17.70	CRT
NZ_CP009110	6052181	6052333	24	4	0.65	0.13	19.00	0.62	11.00	0.00	-8.60	0.00	0.00	15.90	15.90	CRT
NZ_CP009110	7201401	7201689	25	5	0.53	0.27	41.00	0.57	16.00	0.00	-1.90	0.00	0.00	15.90	15.90	CRT
NC_016816	3919949	3920307	23	8	0.54	0.35	25.00	0.36	20.00	0.00	-5.40	0.00	0.00	17.70	17.70	CRT
NC_016816	3920333	3920595	23	6	0.58	0.34	25.00	0.41	14.00	0.00	-5.40	0.00	0.00	17.70	17.70	CRT
NC_016816	4559900	4560107	28	4	0.59	0.43	32.00	0.49	15.00	0.36	-4.30	0.00	0.00	15.90	15.90	CRT
NC_008212	258074	258660	23	10	0.54	0.38	39.67	0.52	36.00	0.73	0.00	0.00	0.00	0.00	15.90	CRT
NC_008212	1383663	1383887	25	4	0.62	0.57	41.67	0.37	7.00	0.00	-6.00	0.00	0.00	34.40	34.40	CRT
NC_008212	2272168	2272406	23	5	0.61	0.61	31.00	0.67	14.00	0.00	-0.90	0.00	0.00	12.20	0.00	CRT
NC_013222	921325	921600	27	4	0.57	0.30	56.00	0.50	10.00	0.00	-2.90	0.00	0.00	14.00	14.00	CRT
NC_012225	245810	246484	33	8	0.64	0.75	58.71	0.51	27.00	0.33	-2.30	0.00	0.00	14.00	17.70	CRT
NC_012225	1412590	1412828	32	4	0.63	0.57	37.00	0.47	19.00	0.36	-6.60	0.00	3.60	14.00	14.00	CRT
NC_012225	2383014	2383336	38	6	0.75	0.67	19.00	0.85	16.00	0.00	-1.70	0.00	-3.00	15.90	15.90	CRT
NZ_CP014774	2389603	2389916	26	5	0.72	0.26	46.00	0.73	7.00	0.00	-8.20	0.00	13.20	14.00	14.00	CRT
NZ_CP014774	3129040	3129240	32	4	0.59	0.45	24.33	0.37	29.00	0.00	-3.50	0.00	0.00	12.20	14.00	CRT
NZ_CP007056	386501	386753	37	4	0.75	0.48	35.00	0.34	13.00	0.00	-3.50	0.00	0.00	19.60	21.40	CRT
NC_016640	1590010	1590282	28	6	0.90	0.40	21.00	0.77	2.00	0.28	-8.90	0.00	0.00	14.00	15.90	CRT
NC_016640	6082559	6082923	37	6	0.81	0.49	28.60	0.78	25.00	0.28	-8.80	0.00	0.00	12.20	0.00	CRT
NC_015711	2379451	2379923	30	7	0.91	0.34	43.83	0.64	5.00	0.00	-9.10	0.00	0.00	14.00	14.00	CRT
NC_019693	5228788	5229068	37	5	0.90	0.47	24.00	0.68	5.00	0.39	-9.50	62.11	0.00	14.00	0.00	CRT
NZ_CP009515	3252411	3252961	35	7	0.90	0.51	51.00	0.86	27.00	0.87	-8.30	0.00	-12.90	12.20	0.00	CRT
NZ_CP009503	1977204	1977668	33	9	0.95	0.60	21.00	0.63	16.00	0.00	0.00	0.00	-4.20	0.00	0.00	CRT
NC_010175	2273592	2273968	23	6	0.94	0.36	47.80	0.75	1.00	0.28	-5.50	0.00	0.00	14.00	14.00	CRT
NC_011831	3268990	3269501	24	7	1.00	0.46	57.33	0.80	0.00	0.36	-5.10	0.00	0.00	0.00	0.00	CRT
NC_013757	408010	408875	38	13	0.87	0.09	31.00	0.75	26.00	0.61	-21.40	0.00	0.00	15.90	15.90	CRT
NC_013757	3588958	3589433	26	11	0.86	0.21	19.00	1.00	8.00	0.00	-9.10	0.00	0.00	12.20	0.00	CRT
NC_013757	4870455	4870687	23	6	0.88	0.34	19.00	1.00	2.00	0.00	0.00	0.00	0.00	12.20	0.00	CRT
NC_009792	1148936	1149218	28	6	0.86	0.35	23.00	0.71	4.00	0.00	-16.40	0.00	0.00	12.20	0.00	CRT
NC_009792	2805460	2805720	27	4	0.89	0.35	51.00	0.98	2.00	0.00	-10.50	0.00	0.00	0.00	0.00	CRT
NC_009792	3462661	3462941	38	5	0.89	0.42	22.75	0.78	9.00	0.31	-5.20	0.00	0.00	12.20	0.00	CRT
NC_009792	3602395	3602576	23	4	0.83	0.41	30.00	0.62	4.00	0.00	-3.50	0.00	0.00	12.20	14.00	CRT
NZ_CP008953	47752	48062	23	5	0.86	0.31	49.00	0.83	6.00	0.00	-3.50	0.00	0.00	14.00	14.00	CRT
NC_014330	2065358	2065695	38	5	0.87	0.69	37.00	0.80	4.00	0.00	0.00	0.00	-23.60	15.90	23.30	CRT
NC_015312	1956110	1956344	25	5	0.86	0.26	27.50	0.82	3.00	0.31	-5.70	0.00	3.00	12.20	0.00	CRT

NC_013729	5376379	5376655	36	4	0.81	0.29	44.33	0.86	9.00	0.00	-4.90	0.00	0.00	12.20	17.70	CRT
NC_009481	914034	914218	32	4	0.82	0.41	19.00	1.00	4.00	0.00	-5.30	0.00	-2.80	12.20	15.90	CRT
NC_008212	104916	105325	38	7	0.83	0.46	24.00	0.91	9.00	0.25	-6.60	0.00	0.00	14.00	15.90	CRT
NC_013222	21786	22066	35	5	0.88	0.49	26.50	0.73	3.00	0.39	-3.60	0.00	0.00	14.00	0.00	CRT
NC_012225	57	703	30	10	0.86	0.67	38.56	0.83	13.00	0.00	0.00	0.00	0.00	14.00	14.00	CRT
NC_012225	2381335	2381630	26	7	0.86	0.71	19.00	0.64	5.00	0.00	-2.40	0.00	-8.80	14.00	14.00	CRT
NC_009635	1121706	1123032	37	19	0.99	0.65	34.67	0.37	1.00	0.33	-8.10	0.00	0.00	32.50	32.50	CRT
NC_002578	846049	849106	29	47	0.99	0.72	36.85	0.32	3.00	0.38	-1.80	0.00	0.00	21.40	23.30	CRT
NZ_CP014140	1750499	1751850	30	21	0.97	0.66	36.10	0.31	2.00	0.31	-2.60	0.00	0.00	34.40	45.40	CRT
NZ_CP014140	3202203	3202892	29	11	0.99	0.72	37.10	0.31	1.00	0.38	-0.30	0.00	0.00	25.10	25.10	CRT
NZ_CP014140	3215204	3218530	29	51	0.99	0.66	36.96	0.31	3.00	0.36	0.00	80.12	0.00	21.40	21.40	CRT
NC_017275	740059	742089	24	35	0.96	0.71	35.03	0.32	9.00	0.33	0.00	0.00	0.00	15.90	15.90	CRT
NC_017275	753266	754065	24	14	0.96	0.71	35.69	0.34	5.00	0.34	0.00	0.00	0.00	15.90	15.90	CRT
NZ_CP010905	1419380	1420263	29	14	0.98	0.76	36.77	0.36	1.00	0.37	-2.30	0.00	0.00	54.70	54.70	CRT
NZ_CP010905	1740903	1741458	29	9	1.00	0.83	36.88	0.37	0.00	0.37	-4.00	0.00	0.00	36.20	36.20	CRT
NZ_CP010905	1920107	1921120	29	16	0.98	0.79	36.67	0.35	1.00	0.38	-2.10	62.46	0.00	47.30	47.30	CRT
NZ_CP010905	2647541	2648492	29	15	0.96	0.79	36.93	0.33	4.00	0.38	0.00	0.00	0.00	30.70	30.70	CRT
NZ_CP010905	2892089	2892514	29	7	1.00	0.83	37.17	0.33	0.00	0.36	0.00	0.00	0.00	36.20	36.20	CRT
NZ_CP010905	3440140	3441286	29	18	0.96	0.79	36.76	0.32	4.00	0.34	0.00	0.00	0.00	52.80	52.80	CRT
NC_006624	468981	470566	30	24	0.96	0.68	37.65	0.31	8.00	0.32	-0.90	0.00	0.00	32.50	54.70	CRT
NC_021353	930280	937725	31	111	1.00	0.74	36.41	0.32	0.00	0.37	-1.90	0.00	0.00	17.70	17.70	CRT
NC_013205	38911	40569	37	23	0.91	0.37	36.73	0.30	16.00	0.37	-13.30	54.47	0.00	15.90	15.90	CRT
NC_013205	65468	66899	37	20	0.96	0.37	36.42	0.31	10.00	0.38	-13.00	0.00	0.00	15.90	15.90	CRT
NC_013205	2945305	2946295	36	14	0.98	0.36	37.46	0.32	3.00	0.34	-13.10	0.00	0.00	14.00	0.00	CRT
NC_012804	208374	208937	30	9	0.84	0.62	36.75	0.30	51.00	0.37	-0.80	0.00	0.00	28.80	56.50	CRT
NC_012804	1221278	1222718	30	22	0.97	0.63	37.19	0.30	4.00	0.31	-0.20	0.00	0.00	28.80	56.50	CRT
NC_013966	204975	207584	30	40	1.00	0.50	36.15	0.33	1.00	0.36	-7.10	0.00	0.00	56.50	56.50	CRT
NC_013966	217813	218566	30	12	0.88	0.50	35.82	0.32	7.00	0.37	-10.10	0.00	0.00	56.50	56.50	CRT
NC_015931	492539	492783	25	4	0.93	0.67	48.33	0.27	3.00	0.36	-0.50	0.00	0.00	21.40	25.10	CRT
NC_007681	490169	494226	29	62	0.98	0.79	37.05	0.36	19.00	0.37	-0.70	97.83	0.00	17.70	17.70	CRT
NC_007681	1091284	1095036	30	57	1.00	0.83	36.48	0.35	0.00	0.39	0.00	87.08	0.00	19.60	25.10	CRT
NC_007514	2511697	2513118	30	22	1.00	0.70	36.29	0.32	0.00	0.36	-2.30	69.87	0.00	19.60	25.10	CRT
NC_018664	704762	706990	30	34	0.95	0.78	36.64	0.34	14.00	0.39	-0.30	83.37	0.00	19.60	19.60	CRT
NC_018664	2342944	2343634	30	11	0.93	0.78	36.10	0.32	5.00	0.39	0.00	0.00	0.00	17.70	17.70	CRT
NC_016640	2903090	2904079	35	14	0.90	0.66	38.46	0.32	12.00	0.30	-1.90	0.00	0.00	14.00	15.90	CRT
NC_016640	5189874	5191453	35	23	1.00	0.57	35.23	0.31	0.00	0.33	-4.90	0.00	0.00	14.00	19.60	CRT
NC_013887	60125	60377	32	4	0.83	0.59	41.67	0.41	16.00	0.36	-3.80	0.00	0.00	25.10	27.00	CRT
NC_013887	149917	150764	31	13	0.95	0.59	37.08	0.32	6.00	0.38	-3.80	0.00	0.00	25.10	27.00	CRT
NC_013887	321361	322389	30	16	0.85	0.63	36.60	0.31	13.00	0.38	-3.10	0.00	0.00	36.20	36.20	CRT
NC_013887	1004051	1004623	30	9	0.87	0.63	37.88	0.36	6.00	0.31	-3.10	0.00	0.00	36.20	36.20	CRT
NC_013887	1021672	1022736	30	16	0.89	0.62	39.00	0.35	15.00	0.36	-3.10	0.00	0.00	36.20	36.20	CRT
NC_013887	1507562	1508968	30	21	0.91	0.63	38.85	0.34	22.00	0.36	-3.10	85.14	0.00	36.20	36.20	CRT
NC_013887	1693281	1694227	31	14	0.92	0.62	39.46	0.33	8.00	0.30	-3.50	0.00	0.00	19.60	25.10	CRT
NC_017584	213598	214173	37	8	0.97	0.44	40.00	0.33	1.00	0.44	-15.60	0.00	0.00	17.70	17.70	CRT
NC_017584	2715965	2716300	37	5	0.90	0.44	37.75	0.31	2.00	0.39	-15.30	0.00	0.00	17.70	17.70	CRT
NC_008698	1227253	1228098	24	14	1.00	0.63	39.23	0.33	0.00	0.34	-3.40	0.00	0.00	17.70	17.70	CRT
NC_008698	1253733	1254901	24	20	0.99	0.63	36.26	0.31	2.00	0.38	-3.40	0.00	0.00	17.70	17.70	CRT

NC_008698	1260196	1260642	24	8	0.93	0.62	36.43	0.34	2.00	0.38	-3.40	0.00	0.00	17.70	17.70	CRT
NC_008698	1269389	1270964	24	27	1.00	0.63	35.69	0.29	0.00	0.37	0.00	0.00	0.00	17.70	17.70	CRT
NC_008698	1271260	1273333	24	35	0.99	0.63	36.29	0.30	2.00	0.42	-3.40	66.09	0.00	17.70	17.70	CRT
NC_008698	1281906	1282413	24	9	1.00	0.63	36.50	0.32	0.00	0.39	-3.40	0.00	0.00	17.70	17.70	CRT
NC_013410	1895255	1895660	37	6	0.95	0.56	36.80	0.36	4.00	0.38	-6.80	0.00	0.00	15.90	15.90	CRT
NC_013410	1902105	1902585	37	7	0.93	0.53	37.00	0.32	6.00	0.36	-7.20	88.31	0.00	15.90	15.90	CRT
NZ_CP014232	643050	643669	37	9	0.84	0.48	35.88	0.32	8.00	0.37	-8.70	0.00	0.00	15.90	15.90	CRT
NC_017941	1229454	1230599	30	18	0.95	0.50	35.65	0.31	10.00	0.34	-6.80	0.00	0.00	39.90	39.90	CRT
NC_017941	1259098	1260440	30	21	1.00	0.50	35.65	0.32	0.00	0.31	-6.80	0.00	0.00	39.90	39.90	CRT
NC_017941	2102185	2103887	30	26	0.96	0.50	36.92	0.32	15.00	0.42	-6.80	0.00	0.00	39.90	39.90	CRT
NC_000917	398369	401590	30	48	0.99	0.63	37.91	0.32	1.00	0.32	-2.50	53.90	0.00	25.10	56.50	CRT
NC_009715	870982	871542	30	9	0.97	0.70	36.38	0.31	1.00	0.37	-1.50	0.00	0.00	15.90	19.60	CRT
NC_009715	878204	878430	30	4	0.93	0.75	35.67	0.32	4.00	0.36	-1.40	0.00	0.00	15.90	19.60	CRT
NC_009674	2126031	2126385	29	6	0.92	0.66	36.20	0.33	2.00	0.38	0.00	0.00	0.00	21.40	21.40	CRT
NC_009674	2135668	2136221	29	9	0.93	0.66	36.63	0.30	7.00	0.39	0.00	0.00	0.00	21.40	21.40	CRT
NC_015562	7992	8691	37	10	0.89	0.66	36.67	0.35	12.00	0.38	-8.70	0.00	0.00	32.50	32.50	CRT
NC_015562	295799	296134	37	5	0.86	0.67	37.75	0.32	4.00	0.31	-6.70	0.00	0.00	32.50	32.50	CRT
NC_015562	592560	593660	31	16	1.00	0.58	40.33	0.35	0.00	0.48	-7.80	0.00	0.00	27.00	28.80	CRT
NC_015562	952222	953000	37	11	0.88	0.67	37.20	0.34	11.00	0.39	-5.90	90.80	0.00	32.50	32.50	CRT
NC_015562	1045115	1045552	30	7	0.88	0.59	38.00	0.32	13.00	0.36	-7.80	0.00	0.00	25.10	27.00	CRT
NC_015562	1182094	1182647	37	8	0.84	0.67	36.86	0.32	22.00	0.38	-5.90	0.00	0.00	32.50	32.50	CRT
NC_015562	1280412	1280888	37	7	0.96	0.67	36.33	0.32	2.00	0.39	-6.00	0.00	0.00	32.50	32.50	CRT
NC_015562	1346391	1346892	31	8	0.88	0.59	36.29	0.36	18.00	0.33	-7.80	0.00	0.00	27.00	28.80	CRT
NC_015562	1491525	1492727	31	18	0.92	0.56	37.94	0.33	19.00	0.36	-3.90	0.00	0.00	25.10	27.00	CRT
NZ_CP006019	735906	739414	30	53	0.98	0.70	36.90	0.32	14.00	0.39	-0.70	0.00	0.00	28.80	45.40	CRT
NZ_CP006019	1003934	1005710	30	27	0.93	0.73	37.19	0.32	12.00	0.36	-0.70	0.00	0.00	27.00	43.60	CRT
NZ_CP006019	1426423	1428518	30	32	0.97	0.70	36.65	0.31	7.00	0.38	-2.00	0.00	0.00	34.40	51.00	CRT
NC_011026	2683900	2694522	30	161	1.00	0.63	36.21	0.31	3.00	0.36	-2.80	98.14	0.00	25.10	27.00	CRT
NC_011026	2813147	2819595	30	98	0.98	0.63	36.18	0.31	8.00	0.36	-2.80	96.57	0.00	25.10	27.00	CRT
NZ_CP015577	552511	553395	30	14	0.95	0.77	35.77	0.35	6.00	0.30	-3.00	0.00	0.00	17.70	19.60	CRT
NC_005090	1531926	1533495	37	22	1.00	0.57	36.00	0.32	0.00	0.33	-4.30	0.00	0.00	12.20	15.90	CRT
NC_014720	2663461	2675104	29	177	0.99	0.66	36.99	0.33	8.00	0.36	0.00	0.00	0.00	25.10	27.00	CRT
NC_014720	2725100	2725725	30	10	0.94	0.50	36.22	0.30	5.00	0.38	-7.20	0.00	0.00	19.60	34.40	CRT
NC_014720	2735113	2736658	30	24	0.95	0.51	35.91	0.31	7.00	0.32	-7.20	0.00	0.00	19.60	34.40	CRT
NC_014720	2745478	2746034	30	9	1.00	0.50	35.88	0.29	0.00	0.31	-7.20	0.00	0.00	19.60	34.40	CRT
NC_015416	2747357	2747982	37	9	1.00	0.59	36.63	0.33	0.00	0.37	-2.70	0.00	0.00	34.40	51.00	CRT
NC_015416	2749613	2750460	37	12	1.00	0.59	36.73	0.32	0.00	0.38	-2.70	79.20	0.00	34.40	51.00	CRT
NC_015416	2856874	2860348	37	47	1.00	0.57	37.74	0.32	0.00	0.34	-5.20	81.84	0.00	21.40	23.30	CRT
NC_018224	842751	845139	30	37	1.00	0.40	35.53	0.32	1.00	0.31	-9.00	89.10	0.00	39.90	39.90	CRT
NC_018224	2861125	2862408	30	20	0.96	0.43	36.00	0.33	4.00	0.32	-9.50	0.00	0.00	51.00	51.00	CRT
NC_011295	1263870	1264966	30	17	0.98	0.60	36.69	0.31	1.00	0.38	-2.00	97.78	0.00	23.30	45.40	CRT
NC_014804	1334169	1334873	30	11	0.97	0.66	37.50	0.30	1.00	0.34	-0.20	0.00	0.00	39.90	56.50	CRT
NC_015711	2377930	2379191	36	18	1.00	0.31	36.12	0.34	0.00	0.39	-12.40	0.00	0.00	21.40	21.40	CRT
NC_015711	2680594	2682334	37	25	0.99	0.33	34.00	0.35	1.00	0.30	-20.50	79.10	0.00	23.30	25.10	CRT
NZ_CP012098	3066058	3066820	30	12	0.93	0.80	36.64	0.30	8.00	0.38	0.00	82.58	0.00	19.60	27.00	CRT
NC_018524	4595354	4595620	24	5	0.91	0.22	36.75	0.37	1.00	0.31	-14.30	0.00	0.00	25.10	25.10	CRT
NC_017034	1139126	1145088	37	82	1.00	0.62	36.16	0.30	1.00	0.36	-7.70	97.86	0.00	28.80	28.80	CRT

NC_020449	1486615	1498952	38	167	1.00	0.68	36.10	0.31	2.00	0.34	-2.30	75.73	0.00	32.50	32.50	CRT
NZ_CP011266	1304078	1304553	37	7	0.91	0.74	36.17	0.33	4.00	0.36	-1.50	0.00	0.00	27.00	27.00	CRT
NC_015703	4509667	4510731	37	15	1.00	0.59	36.43	0.33	0.00	0.38	-5.40	72.13	0.00	19.60	19.60	CRT
NC_015703	5772288	5784494	30	185	1.00	0.67	36.18	0.31	4.00	0.35	-0.90	99.22	0.00	30.70	56.50	CRT
NC_014721	2666352	2668033	30	26	0.97	0.50	36.08	0.31	6.00	0.36	-6.10	0.00	0.00	19.60	28.80	CRT
NC_014721	2669623	2673685	30	62	0.91	0.49	36.11	0.33	31.00	0.35	-6.10	0.00	0.00	19.60	28.80	CRT
NC_014721	2708850	2712824	29	61	0.99	0.62	36.77	0.33	3.00	0.38	0.00	94.77	0.00	19.60	25.10	CRT
NC_007413	4821250	4823752	37	35	0.99	0.60	35.53	0.31	6.00	0.35	-8.00	80.25	0.00	23.30	34.40	CRT
NC_007413	5227213	5229282	37	29	0.99	0.65	35.61	0.30	2.00	0.33	-8.10	62.23	0.00	21.40	28.80	CRT
NC_013158	1415670	1419119	30	53	0.97	0.40	35.77	0.34	15.00	0.31	-5.00	89.23	0.00	45.40	45.40	CRT
NC_014098	2703853	2705077	37	17	1.00	0.27	37.25	0.34	0.00	0.37	-17.30	0.00	0.00	14.00	17.70	CRT
NC_014098	2750070	2768077	30	266	1.00	0.50	37.84	0.32	3.00	0.31	-1.60	93.40	0.00	30.70	36.20	CRT
NC_014098	2770123	2778650	30	127	0.99	0.50	37.44	0.32	17.00	0.31	-1.60	85.70	0.00	30.70	36.20	CRT
NZ_CP012152	724013	724309	30	5	0.93	0.63	36.75	0.29	4.00	0.39	-0.60	0.00	0.00	51.00	56.50	CRT
NZ_CP012152	1992529	1995072	29	39	0.97	0.62	37.18	0.33	12.00	0.37	0.00	0.00	0.00	27.00	27.00	CRT
NC_019757	1088841	1090259	37	20	1.00	0.65	35.74	0.32	0.00	0.32	-8.40	50.23	0.00	23.30	34.40	CRT
NC_019757	1101502	1103134	37	23	0.99	0.59	35.55	0.31	1.00	0.33	-7.80	0.00	0.00	23.30	28.80	CRT
NC_019757	3829839	3832320	37	35	1.00	0.65	34.91	0.32	0.00	0.30	-7.80	97.70	0.00	23.30	34.40	CRT
NC_019757	4707333	4708567	36	17	0.87	0.65	38.94	0.34	17.00	0.38	-8.70	0.00	0.00	15.90	15.90	CRT
NC_019757	4718802	4719567	36	11	0.90	0.63	37.00	0.31	14.00	0.38	-8.70	0.00	0.00	15.90	15.90	CRT
NC_019757	5966534	5968600	37	29	1.00	0.65	35.50	0.33	0.00	0.33	-7.80	0.00	0.00	23.30	34.40	CRT
NC_019757	6781509	6782133	37	9	0.89	0.65	36.50	0.32	19.00	0.37	-7.30	54.99	0.00	23.30	34.40	CRT
NC_019757	6914375	6915668	37	18	0.97	0.48	36.94	0.33	3.00	0.38	-9.20	0.00	0.00	30.70	30.70	CRT
NZ_CP017006	8	5416	24	91	0.99	0.67	35.83	0.31	9.00	0.30	0.00	0.00	0.00	15.90	15.90	CRT
NZ_CP017006	2666936	2674015	24	119	1.00	0.67	35.80	0.31	2.00	0.34	0.00	0.00	0.00	15.90	15.90	CRT
NC_020055	118127	118736	36	9	0.93	0.59	35.75	0.32	7.00	0.31	-5.10	0.00	0.00	19.60	19.60	CRT
NZ_CP016804	1201311	1203838	30	39	1.00	0.50	35.74	0.33	1.00	0.31	-5.00	0.00	0.00	39.90	39.90	CRT
NC_015388	87673	94203	37	90	0.98	0.62	35.97	0.32	7.00	0.30	-3.00	51.40	0.00	39.90	39.90	CRT
NC_015388	1934754	1935816	36	15	1.00	0.56	37.36	0.32	0.00	0.33	-2.50	0.00	0.00	14.00	17.70	CRT
NZ_CP014265	463459	466725	30	50	0.98	0.77	36.06	0.34	13.00	0.34	-0.50	73.08	0.00	28.80	49.10	CRT
NZ_CP014265	466896	467323	30	7	1.00	0.77	36.33	0.34	0.00	0.36	-0.50	0.00	0.00	28.80	49.10	CRT
NZ_CP014265	467360	477912	30	160	0.99	0.77	36.18	0.34	15.00	0.36	-0.50	89.96	0.00	28.80	49.10	CRT
NC_019779	751854	754744	37	40	1.00	0.62	36.18	0.31	0.00	0.36	-9.80	50.65	0.00	19.60	25.10	CRT
NC_019779	769221	777947	37	120	1.00	0.62	36.03	0.32	0.00	0.35	-10.20	0.00	0.00	19.60	25.10	CRT
NC_019779	1442117	1459695	36	237	1.00	0.67	38.33	0.32	0.00	0.31	-5.40	62.59	0.00	30.70	30.70	CRT
NC_019779	2090436	2094051	37	50	0.98	0.62	36.04	0.31	9.00	0.37	-9.50	0.00	0.00	19.60	19.60	CRT
NC_014152	2550336	2554327	30	60	0.97	0.63	37.15	0.31	10.00	0.37	-1.00	0.00	0.00	34.40	56.50	CRT
NC_014152	2567569	2571265	30	56	0.97	0.63	36.67	0.31	6.00	0.39	-1.00	0.00	0.00	34.40	56.50	CRT
NC_014152	2572406	2572637	31	4	0.85	0.61	36.00	0.26	6.00	0.36	-1.40	0.00	0.00	34.40	58.40	CRT
NC_014152	2575925	2582292	30	95	1.00	0.63	37.43	0.31	4.00	0.34	-1.00	52.31	0.00	34.40	56.50	CRT
NC_009135	742879	744906	37	28	1.00	0.73	36.74	0.32	0.00	0.37	-1.80	0.00	0.00	69.40	69.40	CRT
NC_000961	994360	994785	30	7	0.90	0.73	36.00	0.32	4.00	0.39	0.00	0.00	0.00	54.70	54.70	CRT
NC_000961	1117805	1118970	30	18	0.99	0.73	36.82	0.31	4.00	0.34	-2.00	0.00	0.00	56.50	56.50	CRT
NZ_CP007493	1309077	1309292	25	4	0.83	0.71	38.67	0.37	3.00	0.36	0.00	0.00	0.00	19.60	21.40	CRT
NZ_CP007493	1408046	1408328	25	5	0.83	0.71	39.50	0.28	4.00	0.31	0.00	0.00	0.00	19.60	19.60	CRT
NZ_CP009501	1021519	1026905	37	74	1.00	0.65	36.29	0.31	4.00	0.38	-3.10	96.62	0.00	38.10	38.10	CRT
NZ_CP009501	2418131	2420381	37	31	1.00	0.67	36.80	0.32	1.00	0.38	-3.10	99.31	0.00	38.10	38.10	CRT

NZ_CP009501	3029861	3031065	37	17	1.00	0.65	36.00	0.30	0.00	0.37	-3.10	0.00	0.00	38.10	38.10	CRT
NZ_CP009501	3031071	3033895	37	39	0.98	0.65	36.37	0.31	8.00	0.39	-3.10	0.00	0.00	38.10	38.10	CRT
NC_019977	1144393	1145732	37	18	0.94	0.63	39.65	0.32	10.00	0.48	-3.40	51.33	0.00	38.10	38.10	CRT
NC_019977	1149272	1149838	37	8	0.97	0.63	38.71	0.35	4.00	0.38	-3.10	0.00	0.00	32.50	32.50	CRT
NC_019693	6251489	6253282	37	25	0.97	0.57	36.21	0.30	5.00	0.38	-11.00	92.54	0.00	27.00	27.00	CRT
NC_019693	7038938	7043138	37	58	0.96	0.54	36.05	0.31	20.00	0.35	-9.20	84.42	0.00	19.60	23.30	CRT
NC_019693	7504261	7509286	37	70	0.98	0.54	35.30	0.32	13.00	0.31	-9.20	90.73	0.00	19.60	23.30	CRT
NC_018227	2627396	2638039	36	146	0.99	0.56	37.16	0.31	20.00	0.36	-12.30	66.98	0.00	15.90	15.90	CRT
NC_005877	1540318	1545873	30	83	1.00	0.77	37.39	0.33	2.00	0.34	-0.80	74.89	0.00	23.30	28.80	CRT
NZ_CP009524	282078	284324	37	31	0.99	0.62	36.67	0.33	1.00	0.38	-3.10	0.00	0.00	38.10	38.10	CRT
NZ_CP009524	716265	727114	37	149	1.00	0.62	36.06	0.31	3.00	0.36	-2.60	97.67	0.00	38.10	38.10	CRT
NZ_CP009520	284092	288079	37	55	0.98	0.62	36.17	0.31	19.00	0.33	-3.10	0.00	0.00	38.10	38.10	CRT
NZ_CP009520	737419	742144	37	65	1.00	0.62	36.27	0.32	1.00	0.38	-2.60	0.00	0.00	38.10	38.10	CRT
NZ_CP009520	3545403	3546205	37	11	0.96	0.48	39.60	0.32	18.00	0.45	-4.80	0.00	0.00	14.00	0.00	CRT
NC_019753	669542	671689	36	29	0.99	0.42	39.43	0.34	2.00	0.41	-11.30	0.00	0.00	27.00	27.00	CRT
NZ_CP009508	2768489	2769988	37	21	0.99	0.62	36.15	0.32	2.00	0.38	-2.30	98.36	0.00	36.20	36.20	CRT
NZ_CP009508	2819194	2822674	37	48	0.99	0.62	36.28	0.31	5.00	0.39	-2.60	0.00	0.00	38.10	38.10	CRT
NZ_CP009508	4282906	4287833	37	68	0.99	0.65	36.00	0.31	2.00	0.35	-2.60	0.00	0.00	38.10	38.10	CRT
NZ_CP014672	1533576	1535710	30	33	1.00	0.77	35.78	0.31	0.00	0.33	-0.30	0.00	0.00	17.70	25.10	CRT
NC_007355	356467	359809	30	51	0.98	0.60	36.26	0.33	3.00	0.36	-4.20	0.00	0.00	19.60	27.00	CRT
NC_007355	4007239	4009012	36	25	0.90	0.56	36.42	0.31	19.00	0.39	-7.00	57.86	0.00	17.70	19.60	CRT
NC_018870	1915104	1923411	37	113	1.00	0.54	36.85	0.32	1.00	0.38	-6.50	95.71	0.00	30.70	30.70	CRT
NC_018870	2204611	2211549	30	105	1.00	0.57	36.43	0.31	0.00	0.38	-3.90	0.00	0.00	38.10	51.00	CRT
NC_018870	2439391	2440146	30	12	0.92	0.58	36.00	0.30	10.00	0.37	-2.60	0.00	0.00	41.70	56.50	CRT
NC_018870	2443574	2446006	30	37	0.91	0.57	36.75	0.31	30.00	0.37	-2.60	0.00	0.00	41.70	56.50	CRT
NC_018870	2449704	2451060	30	21	0.96	0.60	36.35	0.30	10.00	0.38	-2.50	0.00	0.00	45.40	56.50	CRT
NC_018870	2453564	2455403	30	28	0.97	0.60	37.04	0.30	11.00	0.39	-2.50	0.00	0.00	45.40	56.50	CRT
NC_018870	2455676	2458157	30	38	0.96	0.60	36.27	0.30	19.00	0.37	-2.50	0.00	0.00	45.40	56.50	CRT
NC_018870	2460040	2463469	30	52	0.97	0.60	36.67	0.31	22.00	0.39	-2.50	97.95	0.00	45.40	56.50	CRT
NC_018870	2463742	2464503	30	12	0.93	0.60	36.55	0.27	10.00	0.37	-2.50	0.00	0.00	45.40	56.50	CRT
NC_015865	131168	133209	29	31	0.99	0.55	38.10	0.32	4.00	0.32	-3.40	0.00	0.00	14.00	14.00	CRT
NC_015865	137547	140105	29	39	0.98	0.55	37.58	0.32	4.00	0.34	-2.40	61.82	0.00	14.00	15.90	CRT
NC_015865	345768	346601	30	13	0.91	0.63	37.00	0.30	14.00	0.36	-0.20	0.00	0.00	28.80	56.50	CRT
NC_000909	351694	352468	30	12	0.94	0.57	37.73	0.32	3.00	0.37	-4.20	50.00	0.00	25.10	25.10	CRT
NC_000909	428572	429636	30	16	0.93	0.61	39.00	0.37	13.00	0.32	-3.00	0.00	0.00	47.30	47.30	CRT
NC_000909	501491	501989	30	8	0.90	0.61	37.00	0.25	6.00	0.33	-4.10	0.00	0.00	25.10	27.00	CRT
NC_000909	507264	508115	30	13	0.89	0.62	38.50	0.35	15.00	0.36	-3.70	0.00	0.00	52.80	52.80	CRT
NC_000909	1034820	1035754	31	14	0.86	0.62	38.54	0.35	25.00	0.37	-4.10	0.00	0.00	27.00	28.80	CRT
NC_000909	1267046	1267714	31	10	0.96	0.59	39.89	0.34	3.00	0.38	-4.10	0.00	0.00	25.10	27.00	CRT
NC_000909	1575519	1576383	30	13	0.90	0.70	39.58	0.35	9.00	0.31	-2.70	0.00	0.00	51.00	51.00	CRT
NC_019892	474123	487675	36	189	0.99	0.36	35.90	0.33	11.00	0.33	-8.10	99.49	0.00	12.20	15.90	CRT
NC_019892	5278274	5282042	37	52	0.96	0.30	36.18	0.33	10.00	0.36	-16.70	98.84	0.00	15.90	15.90	CRT
NC_019892	5291494	5297044	37	76	0.98	0.36	36.52	0.33	20.00	0.38	-14.70	99.77	0.00	15.90	15.90	CRT
NC_019892	5318064	5322402	37	60	0.98	0.35	35.92	0.33	17.00	0.41	-14.70	99.75	0.00	15.90	15.90	CRT
NC_012883	1328782	1330412	30	25	0.98	0.73	36.71	0.30	7.00	0.39	0.00	0.00	0.00	39.90	56.50	CRT
NZ_CP012159	4524009	4524861	37	12	0.98	0.35	37.18	0.33	1.00	0.37	-12.20	0.00	0.00	19.60	25.10	CRT
NZ_CP012159	7095638	7099260	36	51	0.96	0.39	35.74	0.33	13.00	0.36	-6.30	0.00	0.00	15.90	0.00	CRT

NZ_CP012159	7109486	7114398	36	69	0.99	0.39	35.72	0.34	10.00	0.38	-7.90	99.05	0.00	15.90	0.00	CRT
NZ_CP012159	8802990	8803833	37	12	1.00	0.35	36.36	0.36	0.00	0.33	-12.20	0.00	0.00	19.60	25.10	CRT
NZ_CP011267	273569	274405	30	13	0.92	0.44	37.25	0.31	9.00	0.36	0.00	0.00	0.00	17.70	17.70	CRT
NZ_CP011267	795283	796727	30	22	1.00	0.73	37.38	0.33	0.00	0.33	-1.00	0.00	0.00	36.20	39.90	CRT
NC_012032	3958399	3986066	37	376	1.00	0.54	36.68	0.31	31.00	0.40	-6.40	98.82	0.00	15.90	15.90	CRT
NC_012032	4543861	4550058	37	84	0.95	0.46	37.23	0.33	45.00	0.34	-9.60	99.91	0.00	17.70	19.60	CRT
NC_012034	151654	160400	29	134	1.00	0.66	36.55	0.32	1.00	0.39	-2.70	69.61	0.00	25.10	27.00	CRT
NC_012034	164244	165518	29	20	0.92	0.66	36.58	0.34	17.00	0.39	-2.70	0.00	0.00	25.10	27.00	CRT
NC_012034	2803920	2807402	30	53	0.95	0.53	36.40	0.33	14.00	0.37	-5.60	0.00	0.00	19.60	34.40	CRT
NC_012034	2828426	2831032	30	40	0.97	0.53	36.08	0.32	11.00	0.32	-7.20	0.00	0.00	19.60	32.50	CRT
NC_012034	2856808	2857631	30	13	0.99	0.50	36.17	0.32	1.00	0.31	-7.20	0.00	0.00	19.60	34.40	CRT
NC_015954	190284	194678	37	61	0.99	0.46	35.63	0.33	3.00	0.30	-3.50	0.00	0.00	19.60	21.40	CRT
NC_019771	57665	59539	37	26	0.99	0.70	36.52	0.31	2.00	0.38	-9.80	0.00	0.00	23.30	34.40	CRT
NC_019771	1761752	1763483	37	24	1.00	0.68	36.70	0.32	2.00	0.39	-7.40	0.00	0.00	23.30	34.40	CRT
NC_019771	3250545	3251389	37	12	1.00	0.38	36.45	0.33	0.00	0.37	-14.40	0.00	0.00	19.60	19.60	CRT
NC_019771	4752371	4758593	37	84	0.99	0.57	37.53	0.32	4.00	0.36	-11.20	0.00	0.00	28.80	28.80	CRT
NC_019771	5001036	5003639	37	36	0.99	0.70	36.34	0.32	2.00	0.37	-9.00	0.00	0.00	23.30	34.40	CRT
NC_019771	5738603	5740678	37	29	1.00	0.68	35.82	0.32	0.00	0.35	-6.70	0.00	0.00	23.30	34.40	CRT
NC_019771	5832140	5832391	36	4	1.00	0.47	36.00	0.37	0.00	0.36	-12.10	0.00	0.00	19.60	23.30	CRT
NZ_CP009515	2521336	2523208	37	26	1.00	0.59	36.44	0.31	0.00	0.38	-2.60	84.55	0.00	38.10	38.10	CRT
NZ_CP009515	2704979	2710429	37	74	0.98	0.62	37.16	0.33	11.00	0.38	-2.00	82.20	0.00	41.70	41.70	CRT
NZ_CP009515	2718618	2719924	37	18	0.96	0.62	37.71	0.34	12.00	0.39	-2.00	0.00	0.00	41.70	41.70	CRT
NZ_CP009515	2720506	2722226	37	24	0.98	0.60	36.22	0.31	3.00	0.37	-2.60	91.36	0.00	38.10	38.10	CRT
NZ_CP009515	2722475	2723822	37	19	1.00	0.59	35.83	0.33	0.00	0.36	-2.60	0.00	0.00	38.10	38.10	CRT
NZ_CP009515	2980927	2983739	37	39	1.00	0.60	36.05	0.31	1.00	0.37	-3.10	0.00	0.00	38.10	38.10	CRT
NZ_CP009515	2984293	2984766	37	7	0.98	0.59	35.83	0.29	2.00	0.36	-3.10	0.00	0.00	38.10	38.10	CRT
NZ_CP009515	3273503	3275585	37	29	1.00	0.62	36.07	0.31	0.00	0.36	-3.30	89.96	0.00	25.10	28.80	CRT
NC_019751	316560	317384	35	12	1.00	0.69	36.82	0.35	0.00	0.37	-5.40	0.00	0.00	17.70	17.70	CRT
NC_019751	324319	325428	35	16	1.00	0.66	36.67	0.32	0.00	0.32	-5.20	0.00	0.00	15.90	15.90	CRT
NC_019751	328043	328873	37	12	0.93	0.67	35.18	0.33	6.00	0.33	-7.10	94.13	0.00	17.70	17.70	CRT
NC_019751	1621155	1621703	37	8	0.88	0.49	36.14	0.32	6.00	0.33	-18.40	0.00	0.00	17.70	17.70	CRT
NC_019751	2038085	2039287	37	17	1.00	0.65	35.88	0.32	0.00	0.31	-7.80	0.00	0.00	23.30	28.80	CRT
NC_017461	13013	14144	24	19	1.00	0.67	37.56	0.33	0.00	0.33	-4.60	0.00	0.00	25.10	34.40	CRT
NC_017461	55133	56194	24	18	0.96	0.71	37.06	0.31	3.00	0.36	0.00	0.00	0.00	25.10	45.40	CRT
NC_017461	61768	62836	24	18	0.99	0.71	37.47	0.32	1.00	0.30	-1.30	0.00	0.00	25.10	45.40	CRT
NC_010803	499776	508012	30	124	1.00	0.70	36.72	0.31	2.00	0.39	-1.90	99.70	0.00	21.40	27.00	CRT
NC_010803	1009935	1011874	35	27	0.98	0.69	38.27	0.31	6.00	0.30	-0.70	63.47	0.00	21.40	25.10	CRT
NC_013162	22567	22754	23	4	0.84	0.60	32.00	0.40	2.00	0.61	0.00	0.00	0.00	15.90	17.70	CRT
NC_000918	244561	244791	29	4	1.00	0.55	38.33	0.30	0.00	0.36	-1.30	0.00	0.00	17.70	19.60	CRT
NC_000918	279264	279555	29	5	1.00	0.62	36.75	0.38	0.00	0.39	-1.20	0.00	0.00	14.00	15.90	CRT
NC_000918	1379598	1379894	30	5	0.84	0.57	36.75	0.30	9.00	0.39	-1.90	0.00	0.00	17.70	19.60	CRT
NC_021658	610407	614540	36	57	0.99	0.31	37.18	0.37	4.00	0.38	-16.10	99.90	0.00	15.90	15.90	CRT
NC_021658	9167757	9170662	36	40	0.98	0.31	37.59	0.35	6.00	0.44	-15.10	95.80	0.00	15.90	15.90	CRT
NZ_CP007264	182361	184798	30	37	0.98	0.63	36.89	0.31	6.00	0.33	-0.20	0.00	0.00	28.80	56.50	CRT
NZ_CP007264	451542	455179	30	55	1.00	0.63	36.81	0.31	0.00	0.34	-0.30	88.84	0.00	34.40	51.00	CRT
NZ_CP007264	705938	708770	30	43	0.98	0.60	36.74	0.31	11.00	0.36	-0.30	0.00	0.00	28.80	56.50	CRT
NZ_CP007264	1461759	1462999	30	19	0.99	0.60	37.28	0.31	1.00	0.33	-0.90	0.00	0.00	27.00	54.70	CRT

NC_007181	1650669	1658594	24	133	1.00	0.71	35.86	0.32	1.00	0.35	0.00	0.00	0.00	15.90	15.90	CRT
NC_007181	1670962	1675586	24	78	0.98	0.71	35.75	0.32	5.00	0.32	0.00	0.00	0.00	15.90	15.90	CRT
NC_007181	1786307	1786584	25	5	0.81	0.62	38.25	0.34	6.00	0.31	-7.60	0.00	0.00	28.80	47.30	CRT
NZ_CP009503	3179668	3183450	37	52	0.99	0.62	36.45	0.31	5.00	0.37	-2.60	97.34	0.00	38.10	38.10	CRT
NZ_CP009503	3192212	3196902	30	71	1.00	0.63	36.59	0.32	0.00	0.39	-2.70	0.00	0.00	25.10	32.50	CRT
NC_014652	2613496	2618702	29	80	1.00	0.69	36.54	0.32	1.00	0.39	0.00	0.00	0.00	19.60	21.40	CRT
NC_014652	2686105	2687448	30	21	0.94	0.54	35.70	0.33	7.00	0.31	-5.60	98.62	0.00	19.60	34.40	CRT
NC_014652	2722390	2725707	29	51	0.99	0.65	36.78	0.32	1.00	0.37	0.00	0.00	0.00	25.10	27.00	CRT
NC_021044	1272488	1272791	31	5	0.91	0.70	37.25	0.33	3.00	0.31	-1.90	0.00	0.00	27.00	36.20	CRT
NC_021044	1277517	1278040	30	8	0.95	0.70	40.57	0.38	4.00	0.33	-1.50	0.00	0.00	27.00	36.20	CRT
NC_021044	1279053	1279340	29	5	0.96	0.70	35.75	0.34	2.00	0.31	-1.50	0.00	0.00	27.00	36.20	CRT
NC_011567	804177	809818	29	86	1.00	0.62	37.04	0.31	0.00	0.38	-1.30	58.49	0.00	27.00	27.00	CRT
NC_011567	812915	816909	29	61	0.99	0.62	37.10	0.32	5.00	0.38	-1.30	60.55	0.00	27.00	27.00	CRT
NC_013407	226214	226449	31	4	0.90	0.62	37.33	0.32	4.00	0.36	-3.70	0.00	0.00	21.40	21.40	CRT
NC_013407	293401	294444	30	16	0.91	0.61	37.60	0.34	7.00	0.38	-3.70	73.31	0.00	21.40	21.40	CRT
NC_013407	299658	300282	30	10	0.87	0.64	36.11	0.35	9.00	0.54	-3.80	0.00	0.00	32.50	32.50	CRT
NC_013407	443197	444230	30	16	0.89	0.60	36.93	0.37	11.00	0.36	-3.70	0.00	0.00	21.40	21.40	CRT
NC_013407	450018	451283	30	19	0.93	0.58	38.67	0.34	12.00	0.48	-3.70	0.00	0.00	21.40	21.40	CRT
NC_013407	590203	590633	30	7	0.95	0.61	36.83	0.27	3.00	0.39	-3.70	0.00	0.00	21.40	21.40	CRT
NC_013407	743131	744904	30	27	0.89	0.61	37.08	0.35	31.00	0.39	-3.70	99.94	0.00	21.40	21.40	CRT
NC_013407	763499	764628	30	17	0.96	0.60	38.75	0.36	4.00	0.31	-3.70	61.29	0.00	21.40	21.40	CRT
NC_013407	886913	888224	30	20	0.96	0.61	37.47	0.35	5.00	0.39	-3.70	0.00	0.00	21.40	21.40	CRT
NC_013407	934670	935162	30	8	0.89	0.65	36.14	0.36	7.00	0.38	-3.70	0.00	0.00	21.40	21.40	CRT
NC_013407	1092182	1092483	30	5	1.00	0.63	38.00	0.38	0.00	0.39	-3.70	0.00	0.00	23.30	23.30	CRT
NC_013407	1134676	1135852	30	18	1.00	0.60	37.47	0.37	0.00	0.38	-3.70	92.15	0.00	21.40	21.40	CRT
NC_013407	1708464	1709448	30	15	0.92	0.60	38.21	0.32	6.00	0.36	-3.70	91.30	0.00	21.40	21.40	CRT
NZ_CP009149	152747	153244	31	8	0.98	0.61	35.71	0.34	2.00	0.33	-3.00	0.00	0.00	41.70	41.70	CRT
NZ_CP009149	160575	161019	30	7	1.00	0.60	39.17	0.36	0.00	0.36	-4.20	0.00	0.00	47.30	47.30	CRT
NZ_CP009149	287107	288529	30	21	0.89	0.60	39.65	0.34	14.00	0.31	-4.20	0.00	0.00	41.70	41.70	CRT
NZ_CP009149	320136	321364	30	18	0.90	0.63	40.53	0.37	16.00	0.47	-4.20	0.00	0.00	49.10	49.10	CRT
NZ_CP009149	432544	433077	30	8	0.97	0.60	42.00	0.34	1.00	0.53	-4.20	0.00	0.00	47.30	47.30	CRT
NZ_CP009149	769284	770276	30	15	0.91	0.60	38.79	0.34	7.00	0.38	-4.20	0.00	0.00	47.30	47.30	CRT
NZ_CP009149	784070	784580	30	8	0.87	0.61	38.71	0.38	10.00	0.33	-4.20	0.00	0.00	41.70	41.70	CRT
NZ_CP009149	974312	974826	30	8	0.96	0.60	39.29	0.31	1.00	0.33	-3.00	0.00	0.00	41.70	41.70	CRT
NZ_CP009149	1464714	1465475	30	12	0.96	0.61	36.55	0.33	5.00	0.33	-4.20	0.00	0.00	47.30	47.30	CRT
NC_014408	224060	226411	30	36	1.00	0.70	36.34	0.29	0.00	0.38	-3.00	90.23	0.00	25.10	49.10	CRT
NC_019695	3356802	3360948	37	57	1.00	0.68	36.39	0.31	2.00	0.35	-3.30	55.15	0.00	23.30	34.40	CRT
NC_019776	1064438	1065555	37	15	1.00	0.51	40.21	0.32	0.00	0.37	-9.80	83.52	0.00	28.80	28.80	CRT
NC_019776	3180046	3182767	37	38	0.99	0.73	35.57	0.33	8.00	0.31	-3.50	0.00	0.00	27.00	38.10	CRT
NC_019776	4073710	4074460	37	10	1.00	0.49	42.33	0.33	0.00	0.38	-8.10	0.00	0.00	28.80	28.80	CRT
NC_019776	4113500	4114042	37	8	1.00	0.70	35.29	0.35	0.00	0.33	-0.60	0.00	0.00	21.40	34.40	CRT
NC_014962	3141389	3167461	36	361	1.00	0.36	36.33	0.32	15.00	0.38	-14.40	98.99	0.00	19.60	19.60	CRT
NZ_CP015520	1383163	1384693	30	23	0.95	0.64	38.23	0.32	9.00	0.42	-0.90	0.00	0.00	27.00	56.50	CRT
NZ_CP015520	1845174	1845926	30	12	0.89	0.65	35.73	0.30	12.00	0.42	-0.20	66.27	0.00	27.00	54.70	CRT
NZ_CP008887	446433	449955	30	53	0.98	0.63	37.17	0.31	6.00	0.35	-0.20	54.52	0.00	28.80	56.50	CRT
NZ_CP008887	1220049	1224832	30	72	0.99	0.63	36.96	0.30	5.00	0.34	-0.20	0.00	0.00	28.80	56.50	CRT
NZ_CP008887	1720540	1721978	30	22	1.00	0.63	37.10	0.30	1.00	0.31	-0.50	0.00	0.00	34.40	51.00	CRT

NC_002939	74806	77627	37	39	0.98	0.38	36.29	0.31	15.00	0.38	-14.90	82.82	0.00	15.90	15.90	CRT
NC_007503	1926257	1930135	30	59	0.92	0.63	36.36	0.31	49.00	0.36	-2.80	0.00	0.00	27.00	51.00	CRT
NC_007503	1944006	1949572	30	84	1.00	0.63	36.71	0.31	15.00	0.38	-2.80	0.00	0.00	23.30	56.50	CRT
NC_019792	3413161	3421255	30	124	0.99	0.43	35.57	0.32	9.00	0.33	-8.50	66.85	0.00	51.00	51.00	CRT
NC_013156	281438	282884	30	22	1.00	0.57	37.48	0.32	0.00	0.33	-3.00	0.00	0.00	36.20	36.20	CRT
NC_013156	347389	347890	30	8	0.89	0.58	37.43	0.34	7.00	0.38	-4.20	0.00	0.00	30.70	30.70	CRT
NC_013156	531679	532047	30	6	0.98	0.63	37.80	0.32	1.00	0.38	-3.00	0.00	0.00	54.70	54.70	CRT
NC_013156	675146	676254	30	17	0.98	0.60	37.44	0.31	1.00	0.35	-4.20	99.09	0.00	32.50	32.50	CRT
NC_013156	717618	718580	30	15	0.91	0.61	36.64	0.35	11.00	0.39	-4.20	0.00	0.00	32.50	32.50	CRT
NC_013156	1343405	1343703	30	5	1.00	0.60	37.25	0.30	0.00	0.31	-4.20	0.00	0.00	43.60	43.60	CRT
NC_018012	4881006	4881922	37	13	0.92	0.53	36.33	0.33	16.00	0.36	-7.80	0.00	0.00	32.50	32.50	CRT
NZ_CP009505	2794713	2800216	37	76	0.98	0.60	35.89	0.31	10.00	0.31	-3.30	0.00	0.00	38.10	38.10	CRT
NZ_CP009505	3675722	3677661	37	27	0.98	0.59	36.19	0.31	3.00	0.37	-3.40	0.00	0.00	38.10	38.10	CRT
NZ_CP009505	3677799	3678130	37	5	1.00	0.59	36.75	0.34	0.00	0.39	-3.40	0.00	0.00	38.10	38.10	CRT
NZ_CP009505	3680371	3688683	37	114	1.00	0.59	36.24	0.30	0.00	0.37	-3.40	95.54	0.00	38.10	38.10	CRT
NZ_CP009505	3702192	3705176	37	41	0.98	0.60	36.70	0.32	12.00	0.38	-3.20	95.25	0.00	41.70	41.70	CRT
NZ_CP009505	3717699	3718685	37	14	0.99	0.59	36.08	0.32	1.00	0.34	-3.20	69.13	0.00	41.70	41.70	CRT
NZ_CP009505	3725555	3726178	37	9	0.90	0.62	36.38	0.31	14.00	0.37	-2.60	0.00	0.00	38.10	38.10	CRT
NZ_CP016077	2484978	2485831	38	12	0.93	0.24	36.18	0.40	8.00	0.33	-23.30	0.00	0.00	15.90	15.90	CRT
NZ_CP016077	3133043	3134042	37	14	0.89	0.27	37.08	0.35	9.00	0.34	-22.60	0.00	0.00	15.90	15.90	CRT
NZ_CP016077	4231183	4231955	37	11	0.92	0.30	36.60	0.36	10.00	0.38	-17.20	58.20	0.00	14.00	15.90	CRT
NZ_CP016077	4266176	4267245	37	15	0.86	0.26	36.79	0.37	19.00	0.36	-19.20	0.00	0.00	15.90	15.90	CRT
NC_014378	892812	893358	37	8	0.99	0.75	35.86	0.32	1.00	0.33	-3.60	0.00	0.00	23.30	27.00	CRT
NZ_CP014859	364885	365495	37	9	0.90	0.28	34.75	0.41	13.00	0.50	-19.60	0.00	0.00	19.60	23.30	CRT
NZ_CP014859	1200685	1201380	37	10	0.96	0.25	36.22	0.38	3.00	0.38	-23.60	0.00	0.00	17.70	0.00	CRT
NZ_CP014859	3928451	3929219	37	11	0.93	0.32	36.20	0.34	4.00	0.34	-19.70	70.91	0.00	17.70	19.60	CRT
NZ_CP014352	1693660	1699112	36	76	0.99	0.36	36.23	0.34	3.00	0.36	-17.30	93.09	0.00	15.90	15.90	CRT
NZ_CP014352	1700309	1701722	36	20	0.93	0.37	36.53	0.34	7.00	0.38	-17.30	97.59	0.00	15.90	15.90	CRT
NC_015975	1047155	1048214	36	15	0.96	0.59	37.14	0.36	7.00	0.38	-7.30	91.81	0.00	14.00	15.90	CRT
NC_015975	1066225	1068633	30	37	0.99	0.73	36.08	0.32	7.00	0.36	0.00	0.00	0.00	28.80	28.80	CRT
NZ_CP009516	2392664	2394112	37	20	0.96	0.62	37.32	0.35	4.00	0.38	-2.80	0.00	0.00	36.20	39.90	CRT
NZ_CP009516	2400559	2403809	37	45	1.00	0.62	36.05	0.31	0.00	0.36	-2.60	93.39	0.00	38.10	38.10	CRT
NZ_CP007140	457101	457669	30	9	0.97	0.63	37.38	0.30	2.00	0.31	-0.80	0.00	0.00	28.80	56.50	CRT
NZ_CP007140	604299	606074	30	27	0.96	0.63	37.15	0.29	4.00	0.39	-0.80	96.75	0.00	28.80	56.50	CRT
NZ_CP007140	1798198	1800241	30	31	0.98	0.63	37.13	0.30	4.00	0.32	-0.50	0.00	0.00	34.40	51.00	CRT
NZ_CP012946	1769400	1770632	37	17	0.96	0.43	37.75	0.34	13.00	0.31	-10.80	0.00	0.00	15.90	15.90	CRT
NZ_CP012946	2294650	2296180	37	21	0.95	0.35	37.70	0.36	20.00	0.31	-14.80	0.00	0.00	15.90	15.90	CRT
NC_015573	484645	486681	30	31	1.00	0.53	36.90	0.32	0.00	0.39	-5.60	96.53	0.00	32.50	56.50	CRT
NC_015573	642189	644638	29	38	0.93	0.49	36.43	0.31	9.00	0.38	-1.60	98.05	0.00	15.90	17.70	CRT
NC_015573	2972297	2976244	37	54	0.99	0.43	36.79	0.31	10.00	0.39	-5.10	86.56	0.00	27.00	27.00	CRT
NC_015573	2990697	2994950	37	58	0.99	0.43	36.98	0.31	3.00	0.39	-4.10	0.00	0.00	27.00	27.00	CRT
NC_009633	2274931	2278995	30	62	0.99	0.73	36.15	0.33	4.00	0.38	-0.80	0.00	0.00	21.40	21.40	CRT
NC_017079	1976690	2011840	37	482	1.00	0.54	36.00	0.32	2.00	0.33	-3.40	99.93	0.00	14.00	0.00	CRT
NC_017079	2013095	2018981	37	81	0.99	0.54	36.13	0.32	5.00	0.35	-3.40	99.16	0.00	14.00	0.00	CRT
NZ_CP015438	714007	715568	30	24	0.98	0.63	36.61	0.31	2.00	0.39	-0.60	77.75	0.00	51.00	56.50	CRT
NZ_CP015435	212163	213591	30	22	1.00	0.73	36.62	0.32	0.00	0.37	-1.90	0.00	0.00	45.40	51.00	CRT
NZ_CP015435	235572	236134	30	9	0.92	0.71	36.63	0.33	4.00	0.37	-4.50	0.00	16.40	39.90	45.40	CRT

NZ_CP015435	257562	258193	30	10	1.00	0.70	36.89	0.31	0.00	0.36	-4.50	0.00	0.00	39.90	45.40	CRT	
NZ_CP015435	275281	275981	30	11	0.97	0.70	37.10	0.31	1.00	0.34	-4.50	0.00	0.00	39.90	45.40	CRT	
NZ_CP015435	296036	296599	30	9	0.85	0.67	36.75	0.33	9.00	0.37	-3.40	0.00	0.00	28.80	45.40	CRT	
NZ_CP015435	308388	310205	30	28	0.97	0.74	36.22	0.33	6.00	0.36	-0.60	0.00	0.00	34.40	51.00	CRT	
NZ_CP015435	1109847	1110145	30	5	1.00	0.63	37.25	0.26	0.00	0.31	-0.50	0.00	0.00	41.70	47.30	CRT	
NZ_CP015435	2182939	2184772	30	28	0.95	0.67	36.81	0.32	5.00	0.38	0.00	0.00	0.00	34.40	51.00	CRT	
NZ_CP015435	2193741	2195969	30	34	0.97	0.67	36.64	0.32	6.00	0.38	-1.50	0.00	0.00	45.40	51.00	CRT	
NZ_CP015435	2325542	2325904	30	6	1.00	0.73	36.60	0.34	0.00	0.38	0.00	94.82	0.00	45.40	51.00	CRT	
NC_016025	420934	428142	36	101	0.99	0.56	35.73	0.32	11.00	0.33	-8.80	99.67	0.00	14.00	0.00	CRT	
NC_016025	984883	988627	36	52	0.97	0.56	36.73	0.34	31.00	0.37	-9.30	99.50	0.00	15.90	15.90	CRT	
NC_014122	235755	236383	30	10	0.92	0.54	36.56	0.37	5.00	0.38	-5.00	0.00	0.00	15.90	21.40	CRT	
NC_014122	245939	247616	37	23	0.99	0.59	37.59	0.35	1.00	0.38	-5.60	0.00	0.00	32.50	32.50	CRT	
NC_014122	531383	532158	37	11	1.00	0.59	36.90	0.32	0.00	0.39	-5.10	0.00	0.00	32.50	32.50	CRT	
NC_014122	604543	605764	37	17	1.00	0.62	37.06	0.33	0.00	0.37	-5.60	0.00	0.00	32.50	32.50	CRT	
NC_014122	767940	768493	37	8	0.97	0.63	36.86	0.33	3.00	0.38	-5.60	0.00	0.00	32.50	32.50	CRT	
NC_014122	825946	826802	30	13	0.87	0.54	38.92	0.33	14.00	0.36	-5.00	0.00	0.00	15.90	21.40	CRT	
NC_014122	1082729	1083283	37	8	0.95	0.60	37.00	0.32	7.00	0.38	-5.60	0.00	0.00	32.50	32.50	CRT	
NC_014122	1111056	1111998	37	13	1.00	0.59	38.50	0.33	0.00	0.46	-5.60	0.00	0.00	32.50	32.50	CRT	
NC_010175	3962639	3975982	37	182	0.99	0.54	36.52	0.31	13.00	0.40	-6.40	77.18	0.00	15.90	15.90	CRT	
NC_010175	4533814	4540010	37	84	0.96	0.46	37.22	0.33	34.00	0.34	-9.60	99.91	0.00	17.70	19.60	CRT	
NC_014960	757015	762020	37	69	0.98	0.48	36.07	0.31	12.00	0.33	-8.00	62.84	0.00	15.90	19.60	CRT	
NC_003228	2998004	2998546	29	9	0.97	0.75	35.25	0.31	4.00	0.31	0.00	0.00	0.00	23.30	30.70	CRT	
NC_009634	127424	127680	37	4	0.97	0.61	36.33	0.36	1.00	0.36	-4.40	0.00	0.00	27.00	27.00	CRT	
NC_009634	262748	270115	37	101	1.00	0.68	36.31	0.33	6.00	0.37	-3.40	95.05	0.00	28.80	28.80	CRT	
NC_008553	670839	677982	37	98	1.00	0.54	36.27	0.31	0.00	0.41	-1.70	87.99	0.00	39.90	69.40	CRT	
NC_008553	1696036	1700457	37	60	1.00	0.51	37.32	0.32	1.00	0.33	-6.80	98.23	0.00	19.60	25.10	CRT	
NC_014222	339765	342408	37	36	0.99	0.65	37.49	0.34	5.00	0.38	-3.80	0.00	0.00	38.10	38.10	CRT	
NC_014222	539822	541857	37	28	1.00	0.68	37.04	0.34	1.00	0.38	-3.80	68.65	0.00	38.10	38.10	CRT	
NC_014222	582647	594279	31	172	1.00	0.71	36.85	0.33	5.00	0.39	0.00	80.39	0.00	28.80	30.70	CRT	
NC_019429	14346	14672	37	5	0.86	0.62	35.50	0.28	9.00	0.31	-6.10	0.00	0.00	23.30	34.40	CRT	
NC_013201	31920	34849	30	45	0.98	0.47	35.91	0.32	3.00	0.33	-3.60	0.00	0.00	51.00	51.00	CRT	
NC_013315	1504069	1504755	29	11	0.97	0.79	36.80	0.34	2.00	0.38	-2.10	0.00	0.00	52.80	52.80	CRT	
NC_013315	1615439	1615732	29	5	0.82	0.82	37.25	0.37	3.00	0.31	-4.00	0.00	0.00	36.20	36.20	CRT	
NC_013315	1709876	1710232	29	6	0.84	0.79	36.60	0.37	5.00	0.38	-0.10	0.00	0.00	30.70	30.70	CRT	
NC_013315	3244625	3246042	29	22	0.97	0.79	37.14	0.34	4.00	0.31	0.00	0.00	0.00	52.80	52.80	CRT	
NC_013315	3491430	3492052	29	10	0.90	0.83	37.00	0.33	4.00	0.38	-0.80	0.00	0.00	19.60	19.60	CRT	
NC_011529	728608	730178	30	24	1.00	0.70	37.00	0.31	1.00	0.32	-3.20	0.00	0.00	34.40	56.50	CRT	
NC_011529	818816	819904	29	17	0.95	0.55	37.25	0.30	5.00	0.31	-4.00	0.00	0.00	15.90	15.90	CRT	
NZ_CP008874	840840	844503	30	56	0.99	0.40	36.07	0.32	1.00	0.35	-6.60	0.00	0.00	39.90	39.90	CRT	
NC_013385	694576	695935	30	21	0.95	0.56	36.50	0.32	8.00	0.38	-3.20	0.00	0.00	34.40	56.50	CRT	
NC_013385	818981	820731	30	27	0.98	0.53	36.19	0.32	2.00	0.30	-8.20	0.00	0.00	28.80	51.00	CRT	
NC_013385	925686	927039	30	21	1.00	0.57	36.20	0.31	0.00	0.31	-3.20	0.00	0.00	34.40	56.50	CRT	
NC_013385	927177	928403	30	19	1.00	0.57	36.50	0.31	0.00	0.38	-3.20	0.00	0.00	34.40	56.50	CRT	
NC_013385	928543	929635	30	17	1.00	0.57	36.44	0.32	0.00	0.38	-3.20	0.00	0.00	34.40	56.50	CRT	
NC_013385	991878	993835	30	30	1.00	0.57	36.48	0.32	0.00	0.38	-3.20	0.00	0.00	34.40	56.50	CRT	
NC_013385	1042896	1043922	30	16	0.99	0.54	36.47	0.33	1.00	0.39	-4.40	0.00	0.00	21.40	43.60	CRT	
NC_013385	1775088	1775565	37	7	1.00	0.46	36.50	0.31	0.00	0.39	-9.30	0.00	0.00	23.30	23.30	CRT	

NZ_CP009552	977342	977572	30	4	1.00	0.63	37.00	0.41	0.00	0.36	-2.70	0.00	0.00	25.10	32.50	CRT
NZ_CP009552	1354028	1355001	30	15	0.98	0.63	37.43	0.32	1.00	0.33	-2.70	0.00	0.00	19.60	34.40	CRT
NZ_CP009552	1644186	1644488	30	5	1.00	0.63	38.25	0.37	0.00	0.31	-2.70	0.00	0.00	25.10	32.50	CRT
NC_009337	1461368	1461601	32	4	1.00	0.34	35.33	0.33	0.00	0.36	-14.60	0.00	0.00	47.30	52.80	CRT
NC_013939	514731	516696	30	30	0.96	0.64	36.76	0.34	6.00	0.37	-0.60	0.00	0.00	34.40	56.50	CRT
NC_013939	526129	527564	30	22	0.93	0.64	36.95	0.34	10.00	0.37	0.00	0.00	0.00	39.90	56.50	CRT
NC_015636	103466	105101	30	25	1.00	0.67	36.92	0.33	0.00	0.38	-2.00	91.77	0.00	27.00	34.40	CRT
NC_015636	206578	207146	30	9	0.98	0.67	37.38	0.34	2.00	0.31	-4.40	0.00	0.00	25.10	32.50	CRT
NC_015636	224644	225432	30	12	1.00	0.67	39.00	0.35	0.00	0.52	-4.40	81.03	9.70	27.00	34.40	CRT
NC_015636	225510	230898	30	81	0.99	0.67	36.99	0.34	2.00	0.37	-4.40	89.09	0.00	27.00	34.40	CRT
NC_015636	1302586	1304621	30	31	0.98	0.67	36.87	0.35	10.00	0.39	-2.00	95.98	0.00	27.00	34.40	CRT
NC_015636	1529273	1531189	30	29	1.00	0.67	37.39	0.37	1.00	0.38	-4.40	97.66	0.00	27.00	34.40	CRT
NC_015636	1629402	1633832	30	66	0.99	0.67	37.71	0.34	4.00	0.33	-4.40	92.09	0.00	27.00	34.40	CRT
NZ_CP012024	1857304	1857532	30	4	0.86	0.75	36.33	0.36	6.00	0.36	0.00	0.00	0.00	39.90	45.40	CRT
NZ_CP012024	1859953	1860511	30	9	0.92	0.76	36.13	0.31	11.00	0.37	0.00	0.00	0.00	39.90	45.40	CRT
NZ_CP012024	1914384	1914813	30	7	0.97	0.74	36.67	0.32	3.00	0.39	0.00	0.00	0.00	45.40	51.00	CRT
NZ_CP012024	2794821	2796951	29	33	1.00	0.66	36.69	0.32	0.00	0.39	0.00	66.52	0.00	21.40	21.40	CRT
NZ_CP012024	2797028	2798305	29	20	1.00	0.66	36.74	0.31	0.00	0.39	0.00	0.00	0.00	21.40	21.40	CRT
NC_003552	2379255	2381198	37	27	0.97	0.59	36.35	0.33	14.00	0.36	-3.10	0.00	0.00	38.10	38.10	CRT
NC_003552	4523448	4525551	30	32	0.96	0.64	36.90	0.32	14.00	0.37	-2.70	0.00	0.00	25.10	32.50	CRT
NC_018018	11266	12758	37	21	1.00	0.68	35.80	0.34	1.00	0.36	-2.40	0.00	0.00	32.50	32.50	CRT
NC_018018	205432	206414	37	14	0.98	0.68	35.77	0.33	1.00	0.37	-3.90	0.00	0.00	27.00	27.00	CRT
NC_018018	1678085	1679372	37	18	0.97	0.68	36.59	0.33	6.00	0.38	-2.40	0.00	0.00	32.50	32.50	CRT
NC_018024	1096374	1098139	30	27	1.00	0.63	36.77	0.30	0.00	0.39	-2.50	0.00	0.00	23.30	39.90	CRT
NC_018024	1107551	1113295	30	87	1.00	0.60	36.45	0.30	2.00	0.37	-2.50	97.29	0.00	28.80	39.90	CRT
NC_018024	1604244	1609325	30	77	1.00	0.60	36.47	0.31	0.00	0.38	-2.30	98.31	0.00	23.30	34.40	CRT
NC_018178	929685	930781	30	17	0.98	0.73	36.69	0.30	4.00	0.38	-1.10	0.00	0.00	21.40	27.00	CRT
NC_018178	2661872	2663357	30	23	0.96	0.72	36.18	0.31	13.00	0.35	-2.50	0.00	0.00	21.40	23.30	CRT
NC_015680	556584	557703	30	17	0.95	0.66	38.13	0.31	11.00	0.31	-0.80	68.60	0.00	45.40	56.50	CRT
NC_015680	693109	695095	30	30	0.99	0.67	37.48	0.30	1.00	0.31	-0.80	0.00	0.00	45.40	56.50	CRT
NC_013854	3035846	3038413	37	35	0.98	0.46	37.44	0.33	13.00	0.32	-9.80	0.00	0.00	15.90	15.90	CRT
NC_013854	3158606	3163922	37	72	0.98	0.35	37.37	0.33	18.00	0.36	-17.30	0.00	0.00	15.90	15.90	CRT
NZ_CP012072	1116397	1119038	37	36	0.93	0.49	37.43	0.32	16.00	0.33	-9.90	59.92	0.00	12.20	15.90	CRT
NC_021355	134105	150352	30	246	0.99	0.77	36.20	0.33	5.00	0.36	-0.50	71.88	0.00	23.30	43.60	CRT
NC_003901	679197	682529	37	46	0.96	0.62	36.24	0.31	8.00	0.36	-2.60	66.04	0.00	38.10	38.10	CRT
NC_003901	4089310	4095187	37	81	0.98	0.62	36.01	0.30	9.00	0.33	-2.60	80.00	0.00	38.10	38.10	CRT
NC_011831	688004	688482	37	7	0.93	0.53	36.67	0.35	7.00	0.39	-10.00	0.00	0.00	17.70	19.60	CRT
NC_011831	689334	700642	37	154	1.00	0.54	36.67	0.31	4.00	0.38	-8.10	97.88	0.00	15.90	19.60	CRT
NC_000916	983373	991584	30	124	1.00	0.70	36.52	0.29	0.00	0.38	-2.50	76.69	0.00	25.10	49.10	CRT
NC_000916	992203	992438	29	4	0.81	0.72	40.00	0.33	10.00	0.36	0.00	0.00	0.00	15.90	19.60	CRT
NC_000916	1472442	1475513	30	47	1.00	0.70	36.13	0.29	0.00	0.39	-3.00	96.80	0.00	25.10	49.10	CRT
NC_017459	403466	406637	37	44	0.98	0.54	35.91	0.32	9.00	0.35	-4.30	0.00	0.00	19.60	27.00	CRT
NC_017459	1403321	1404737	30	22	0.96	0.57	36.05	0.30	6.00	0.36	-6.80	0.00	0.00	38.10	38.10	CRT
NZ_CP019286	203519	203684	24	4	0.68	0.42	23.33	0.29	9.00	0.36	-1.00	0.00	0.00	12.20	0.00	CRISPRCasFinder
NZ_CP010905	3288485	3288696	26	4	0.68	0.52	36.00	0.41	13.00	0.61	-0.70	0.00	0.00	15.90	15.90	CRISPRCasFinder
NZ_CP010905	1110217	1110443	29	4	0.70	0.78	37.00	0.34	11.00	0.00	-2.30	0.00	0.00	54.70	54.70	CRISPRCasFinder
NZ_CP010905	3381349	3381576	29	4	0.70	0.78	37.00	0.34	11.00	0.00	0.00	0.00	0.00	47.30	47.30	CRISPRCasFinder

NZ_CP010905	3179342	3179631	25	5	0.48	0.72	41.00	0.44	17.00	0.00	0.00	0.00	0.00	12.20	0.00	CRISPRCasFinder
NZ_CP010905	3124102	3124427	26	6	0.49	0.64	34.00	0.37	25.00	0.00	0.00	0.00	103.00	14.00	14.00	CRISPRCasFinder
NZ_CP018201	1402516	1402895	26	7	0.61	0.29	33.00	0.34	21.00	0.25	-7.60	0.00	0.00	12.20	0.00	CRISPRCasFinder
NC_011832	1642478	1642689	29	4	0.58	0.41	31.67	0.39	12.00	0.00	-12.80	0.00	0.00	17.70	17.70	CRISPRCasFinder
NC_021355	38041	38595	27	10	0.61	0.85	31.67	0.38	44.00	0.71	-0.50	0.00	0.00	14.00	14.00	CRISPRCasFinder
NC_021355	1138302	1138505	24	4	0.64	0.86	36.00	0.40	13.00	0.61	0.00	0.00	0.00	0.00	0.00	CRISPRCasFinder
NC_017030	2472609	2473114	26	8	0.63	0.41	42.43	0.50	18.00	0.00	-12.70	0.00	0.00	0.00	0.00	CRISPRCasFinder
NC_014962	4741898	4742297	24	7	0.54	0.32	38.50	0.47	22.00	0.36	-2.00	62.24	76.30	12.20	0.00	CRISPRCasFinder
NC_018524	4408931	4409149	34	4	0.71	0.27	27.67	0.27	9.00	0.00	-13.10	0.00	0.00	12.20	15.90	CRISPRCasFinder
NC_018524	4593160	4593371	29	4	0.79	0.20	32.00	0.35	5.00	0.00	-14.30	0.00	0.00	25.10	25.10	CRISPRCasFinder
NC_018524	2250995	2251569	25	10	0.77	0.25	36.11	0.35	10.00	0.19	-11.40	0.00	0.00	12.20	15.90	CRISPRCasFinder
NC_018524	2270308	2271122	29	14	0.68	0.23	31.46	0.34	31.00	0.15	-14.50	84.92	0.00	25.10	25.10	CRISPRCasFinder
NC_018524	2357492	2357779	23	6	0.52	0.22	29.80	0.47	19.00	0.59	-4.30	0.00	0.00	14.00	14.00	CRISPRCasFinder
NC_018524	2357840	2358003	23	4	0.43	0.20	24.00	0.45	18.00	0.36	-1.10	0.00	0.00	0.00	0.00	CRISPRCasFinder
NC_018524	313187	313459	29	5	0.75	0.22	32.00	0.29	8.00	0.00	-14.30	0.00	0.00	25.10	25.10	CRISPRCasFinder
NC_010516	1031477	1031901	30	7	0.74	0.79	35.83	0.38	13.00	0.36	-1.70	0.00	0.00	14.00	14.00	CRISPRCasFinder
NC_010516	1897877	1898094	23	4	0.40	0.87	42.00	0.43	18.00	0.00	-0.50	0.00	0.00	14.00	14.00	CRISPRCasFinder
NC_010516	2308515	2308939	30	7	0.67	0.77	35.83	0.36	18.00	0.25	0.00	0.00	0.00	14.00	14.00	CRISPRCasFinder
NC_016640	757692	757954	26	5	0.50	0.45	33.25	0.35	22.00	0.58	-3.00	0.00	0.00	15.90	15.90	CRISPRCasFinder
NC_014408	1634338	1634566	24	5	0.69	0.58	27.25	0.34	8.00	0.31	0.00	0.00	0.00	12.20	14.00	CRISPRCasFinder
NC_000909	471437	471686	29	4	0.67	0.71	44.67	0.30	8.00	0.36	0.00	0.00	0.00	17.70	17.70	CRISPRCasFinder
NC_000909	858112	858344	30	4	0.72	0.65	37.67	0.27	8.00	0.00	-3.80	0.00	0.00	27.00	27.00	CRISPRCasFinder
NC_014374	1496055	1496411	24	6	0.68	0.58	42.60	0.32	15.00	0.28	-3.20	0.00	0.00	17.70	17.70	CRISPRCasFinder
NC_022116	6451812	6452083	29	5	0.64	0.22	31.75	0.34	12.00	0.00	-12.10	0.00	0.00	15.90	15.90	CRISPRCasFinder
NC_022116	6446642	6447640	29	17	0.59	0.23	31.56	0.33	66.00	0.00	-12.10	0.00	0.00	21.40	21.40	CRISPRCasFinder
NC_022116	6448465	6448737	29	5	0.79	0.23	32.00	0.32	11.00	0.00	-12.10	0.00	0.00	21.40	21.40	CRISPRCasFinder
NC_022116	6451277	6451672	29	7	0.69	0.22	32.00	0.39	15.00	0.00	-13.40	0.00	0.00	15.90	15.90	CRISPRCasFinder
NC_022116	6454295	6454563	24	5	0.55	0.26	37.00	0.40	12.00	0.00	-12.90	0.00	0.00	27.00	27.00	CRISPRCasFinder
NC_017271	4289686	4290716	23	22	0.53	0.35	25.00	0.38	71.00	0.00	-10.50	0.00	0.00	15.90	15.90	CRISPRCasFinder
NZ_CP011509	5747576	5747901	26	5	0.54	0.30	49.00	0.47	15.00	0.00	-6.30	0.00	0.00	12.20	0.00	CRISPRCasFinder
NZ_CP011509	2896636	2896858	25	4	0.68	0.30	41.00	0.34	13.00	0.61	-5.40	0.00	-2.00	0.00	0.00	CRISPRCasFinder
NC_016894	3904684	3905123	26	7	0.46	0.59	43.00	0.43	31.00	0.00	-2.50	0.00	0.00	14.00	14.00	CRISPRCasFinder
NC_018610	132852	133062	28	4	0.74	0.45	33.00	0.29	9.00	0.00	-7.00	0.00	0.00	14.00	14.00	CRISPRCasFinder
NC_000961	493810	494097	24	5	0.74	0.54	42.00	0.48	19.00	0.39	0.00	0.00	0.00	14.00	15.90	CRISPRCasFinder
NZ_CP012024	2767794	2768014	24	4	0.75	0.60	41.67	0.39	4.00	0.00	0.00	0.00	0.00	14.00	19.60	CRISPRCasFinder
NC_021149	1708724	1709000	30	5	0.71	0.25	31.50	0.36	17.00	0.00	-14.40	0.00	0.00	32.50	32.50	CRISPRCasFinder
NC_014734	3132650	3133200	26	9	0.80	0.57	39.63	0.41	8.00	0.00	-5.60	0.00	0.00	14.00	14.00	CRISPRCasFinder
NC_014734	2786079	2786274	26	5	0.70	0.69	16.50	0.25	8.00	0.00	-5.00	0.00	0.00	17.70	17.70	CRISPRCasFinder
NC_017584	1281858	1282021	23	4	0.68	0.21	24.00	0.34	13.00	0.36	-2.40	0.00	-1.50	14.00	14.00	CRISPRCasFinder
NC_014122	109860	110089	30	4	0.80	0.58	36.67	0.34	9.00	0.36	-6.20	0.00	0.00	15.90	21.40	CRISPRCasFinder
NZ_CP012159	9925468	9925931	29	6	0.67	0.32	58.00	0.46	20.00	0.00	-4.20	0.00	0.00	12.20	0.00	CRISPRCasFinder
NZ_CP012159	4793091	4793323	23	6	0.53	0.30	19.00	0.29	20.00	0.00	-2.50	0.00	0.00	12.20	0.00	CRISPRCasFinder
NZ_CP012159	7648697	7648889	24	5	0.79	0.29	18.25	0.39	5.00	0.00	-1.90	0.00	0.00	12.20	15.90	CRISPRCasFinder
NC_015634	2497017	2497370	25	6	0.77	0.73	40.80	0.34	6.00	0.00	-0.20	0.00	0.00	38.10	47.30	CRISPRCasFinder
NC_013790	592393	593115	27	10	0.66	0.74	50.33	0.46	29.00	0.19	0.00	0.00	-27.00	17.70	17.70	CRISPRCasFinder
NC_009776	1057227	1057458	29	4	0.70	0.53	38.67	0.33	11.00	0.00	-3.60	0.00	0.00	19.60	23.30	CRISPRCasFinder
NC_009776	952490	952925	26	7	0.68	0.54	42.33	0.32	13.00	0.00	-2.20	0.00	0.00	21.40	27.00	CRISPRCasFinder

NC_009776	557086	557649	26	9	0.71	0.58	41.25	0.31	13.00	0.21	-2.20	0.00	0.00	21.40	27.00	CRISPRCasFinder
NC_009776	413553	414303	26	12	0.67	0.57	39.91	0.32	23.00	0.26	-3.60	0.00	0.00	25.10	25.10	CRISPRCasFinder
NC_009776	915471	915892	23	7	0.72	0.57	43.50	0.31	9.00	0.00	-3.10	0.00	0.00	19.60	23.30	CRISPRCasFinder
NC_013131	4441942	4442333	26	7	0.64	0.26	35.00	0.36	12.00	0.00	-12.90	0.00	0.00	27.00	27.00	CRISPRCasFinder
NC_013131	9124185	9124391	24	4	0.46	0.24	37.00	0.53	13.00	0.36	-5.10	0.00	0.00	0.00	14.00	CRISPRCasFinder
NC_013131	4438676	4439799	26	19	0.77	0.19	35.00	0.35	22.00	0.00	-12.90	0.00	0.00	21.40	21.40	CRISPRCasFinder
NZ_CP014688	337638	338215	29	10	0.62	0.35	32.00	0.29	27.00	0.00	-12.90	0.00	0.00	27.00	27.00	CRISPRCasFinder
NZ_CP014688	349328	349587	28	5	0.45	0.37	30.00	0.28	27.00	0.31	-12.40	0.00	0.00	23.30	23.30	CRISPRCasFinder
NZ_CP014688	337181	337382	29	4	0.41	0.37	28.33	0.30	20.00	0.36	-12.40	0.00	0.00	25.10	25.10	CRISPRCasFinder
NC_015151	1482074	1482364	24	5	0.76	0.79	42.75	0.34	8.00	0.00	-2.50	0.00	0.00	17.70	34.40	CRISPRCasFinder
NC_007681	1095882	1096169	30	5	0.78	0.83	34.50	0.38	18.00	0.31	0.00	0.00	0.00	19.60	25.10	CRISPRCasFinder
NZ_CP014672	2130830	2131091	30	4	0.65	0.78	47.33	0.40	12.00	0.36	-0.60	0.00	0.00	15.90	19.60	CRISPRCasFinder
NC_014738	1695512	1696876	36	18	0.77	0.56	42.18	0.35	51.00	0.30	-2.60	93.52	0.00	15.90	15.90	CRISPRCasFinder
NC_014738	1831280	1831539	29	4	0.56	0.56	48.00	0.47	14.00	0.36	-9.00	0.00	0.00	14.00	15.90	CRISPRCasFinder
NC_017275	2266259	2266502	31	4	0.59	0.64	40.00	0.42	19.00	0.36	-1.60	0.00	0.00	15.90	15.90	CRISPRCasFinder
NZ_CP009516	3470339	3470565	23	4	0.55	0.75	45.00	0.41	13.00	0.00	0.00	0.00	0.00	14.00	15.90	CRISPRCasFinder
NC_013093	6295356	6295930	24	12	0.60	0.41	26.09	0.32	33.00	0.56	-13.70	0.00	0.00	14.00	14.00	CRISPRCasFinder
NC_013093	6440070	6440219	24	4	0.67	0.28	18.00	0.36	7.00	0.00	-4.70	0.00	0.00	14.00	14.00	CRISPRCasFinder
NC_023002	793547	794047	35	6	0.66	0.83	58.00	0.53	19.00	0.00	0.00	0.00	0.00	17.70	17.70	CRISPRCasFinder
NC_023002	576701	576953	40	4	0.71	0.84	31.00	0.39	21.00	0.00	-0.60	0.00	0.00	14.00	15.90	CRISPRCasFinder
NC_023002	792800	793402	35	7	0.70	0.80	59.50	0.48	23.00	0.00	0.00	82.90	0.00	15.90	15.90	CRISPRCasFinder
NZ_CP015820	1915380	1916141	36	12	0.74	0.55	29.91	0.30	33.00	0.00	-6.90	0.00	0.00	15.90	17.70	CRISPRCasFinder
NZ_CP018139	3485490	3485817	23	6	0.72	0.25	38.00	0.36	7.00	0.00	-8.50	0.00	0.00	32.50	32.50	CRISPRCasFinder
NZ_CP018139	1050394	1050612	24	4	0.45	0.23	41.00	0.39	17.00	0.61	-8.50	0.00	-2.50	14.00	14.00	CRISPRCasFinder
NC_019695	3355736	3356062	37	5	0.77	0.65	35.25	0.31	13.00	0.00	-6.00	0.00	0.00	23.30	34.40	CRISPRCasFinder
NC_013961	2891678	2892004	26	6	0.66	0.49	34.20	0.32	9.00	0.00	-9.10	0.00	0.00	49.10	49.10	CRISPRCasFinder
NC_014656	421553	421756	24	4	0.52	0.30	36.00	0.41	13.00	0.36	-6.20	0.00	0.00	14.00	14.00	CRISPRCasFinder
NC_017218	132537	132873	25	7	0.45	0.42	27.00	0.34	28.00	0.00	-5.20	0.00	0.00	17.70	17.70	CRISPRCasFinder
NC_015672	1709819	1710226	36	6	0.68	0.59	38.20	0.34	24.00	0.00	-1.10	0.00	0.00	14.00	15.90	CRISPRCasFinder
NC_012032	1637506	1637742	24	4	0.61	0.24	47.00	0.37	14.00	0.36	-5.50	0.00	0.00	14.00	14.00	CRISPRCasFinder
NC_020449	385220	385527	46	4	0.60	0.62	41.33	0.37	20.00	0.00	-8.50	0.00	0.00	15.90	15.90	CRISPRCasFinder
NC_010085	406483	407331	24	16	0.67	0.55	31.00	0.48	46.00	0.32	-1.50	0.00	0.00	14.00	14.00	CRISPRCasFinder
NC_021030	1994405	1994722	23	6	0.56	0.61	36.00	0.40	22.00	0.38	0.00	65.20	0.00	14.00	14.00	CRISPRCasFinder
NZ_CP007060	2257797	2258025	25	5	0.52	0.33	26.00	0.35	16.00	0.00	-7.10	0.00	0.00	12.20	0.00	CRISPRCasFinder
NZ_CP014214	1153895	1155875	29	33	0.73	0.40	32.00	0.30	70.00	0.00	-12.80	0.00	0.00	27.00	27.00	CRISPRCasFinder
NC_014205	1069948	1070366	25	7	0.59	0.69	40.67	0.38	20.00	0.00	0.00	0.00	0.00	17.70	21.40	CRISPRCasFinder
NC_014205	487286	487580	27	5	0.67	0.69	40.00	0.39	12.00	0.00	0.00	0.00	0.00	21.40	25.10	CRISPRCasFinder
NC_000918	156460	156768	31	5	0.76	0.57	38.50	0.36	18.00	0.39	-3.70	0.00	0.00	17.70	19.60	CRISPRCasFinder
NZ_CP012373	945611	945979	24	6	0.46	0.44	45.00	0.42	19.00	0.00	-4.20	0.00	0.00	12.20	0.00	CRISPRCasFinder
NC_021287	507941	508823	29	15	0.65	0.34	32.00	0.33	38.00	0.00	-11.40	0.00	0.00	12.20	14.00	CRISPRCasFinder
NC_021191	1943594	1943813	28	4	0.51	0.19	36.00	0.41	15.00	0.36	-9.30	0.00	0.00	14.00	14.00	CRISPRCasFinder
NZ_CP011350	704505	705191	32	11	0.71	0.57	33.50	0.32	19.00	0.00	-11.40	0.00	0.00	25.10	30.70	CRISPRCasFinder
NZ_CP011350	697165	697518	24	6	0.61	0.65	42.00	0.47	18.00	0.00	0.00	0.00	0.00	25.10	30.70	CRISPRCasFinder
NC_017093	8477847	8478140	23	6	0.52	0.29	31.00	0.38	25.00	0.00	-5.80	0.00	65.00	12.20	0.00	CRISPRCasFinder
NC_017093	2021893	2022107	25	4	0.55	0.37	38.33	0.37	16.00	0.00	-5.20	0.00	0.00	14.00	14.00	CRISPRCasFinder
NZ_CP007493	1309014	1309292	25	5	0.68	0.67	38.50	0.35	11.00	0.31	0.00	0.00	0.00	19.60	21.40	CRISPRCasFinder
NZ_CP012072	525993	526264	29	5	0.66	0.41	31.75	0.42	17.00	0.00	-5.70	0.00	121.70	14.00	14.00	CRISPRCasFinder

NZ_CP012072	1554612	1554802	23	4	0.45	0.32	33.00	0.42	19.00	0.00	-4.10	0.00	0.00	14.00	14.00	CRISPRCasFinder
NZ_CP014673	2149249	2149575	30	5	0.71	0.79	44.25	0.38	12.00	0.31	-0.60	0.00	0.00	15.90	19.60	CRISPRCasFinder
NC_014720	2788771	2789325	30	9	0.76	0.50	35.63	0.30	12.00	0.21	-5.60	0.00	0.00	19.60	28.80	CRISPRCasFinder
NC_003552	1779366	1779592	23	4	0.49	0.72	45.00	0.38	14.00	0.00	-0.10	0.00	0.00	14.00	15.90	CRISPRCasFinder
NZ_CP016784	2229656	2230019	30	6	0.73	0.53	36.80	0.35	10.00	0.38	-11.50	67.37	0.00	23.30	32.50	CRISPRCasFinder
NZ_CP016784	2237667	2237896	32	4	0.67	0.56	33.67	0.35	7.00	0.00	-11.50	0.00	0.00	25.10	34.40	CRISPRCasFinder
NZ_CP016784	2228729	2229023	30	5	0.67	0.54	36.00	0.33	11.00	0.31	-11.50	0.00	0.00	23.30	27.00	CRISPRCasFinder
NC_006582	3820899	3821324	32	7	0.77	0.53	33.67	0.34	11.00	0.00	-11.50	0.00	0.00	25.10	34.40	CRISPRCasFinder
NC_013315	3137064	3137275	26	4	0.65	0.51	36.00	0.43	15.00	0.61	-0.70	0.00	0.00	15.90	15.90	CRISPRCasFinder
NC_013315	2972867	2973192	26	6	0.49	0.63	34.00	0.37	24.00	0.00	0.00	0.00	105.90	14.00	14.00	CRISPRCasFinder
NC_013315	1712097	1712387	30	5	0.77	0.80	35.25	0.34	10.00	0.31	-4.00	0.00	0.00	36.20	36.20	CRISPRCasFinder
NC_013162	2108495	2108694	24	5	0.64	0.68	20.00	0.29	12.00	0.00	0.00	0.00	0.00	15.90	17.70	CRISPRCasFinder
NC_013162	1981483	1981661	29	4	0.59	0.63	21.00	0.50	25.00	0.00	0.00	0.00	0.00	12.20	0.00	CRISPRCasFinder
NC_019693	5531086	5531303	32	4	0.56	0.55	30.00	0.43	15.00	0.00	-3.70	0.00	0.00	14.00	14.00	CRISPRCasFinder
NC_019693	5530351	5530620	32	5	0.54	0.53	27.25	0.39	18.00	0.31	-2.90	0.00	0.00	14.00	14.00	CRISPRCasFinder
NC_019693	5519080	5519338	28	4	0.50	0.30	49.00	0.45	17.00	0.36	-3.30	0.00	0.00	14.00	15.90	CRISPRCasFinder
NC_009051	153124	153636	28	7	0.71	0.45	52.83	0.49	15.00	0.25	-9.30	0.00	0.00	12.20	14.00	CRISPRCasFinder
NC_007796	1566435	1566644	27	4	0.65	0.45	34.00	0.24	8.00	0.00	-8.60	0.00	0.00	19.60	19.60	CRISPRCasFinder
NC_015573	811112	814237	30	47	0.65	0.61	37.30	0.32	103.00	0.38	-4.80	83.64	0.00	27.00	34.40	CRISPRCasFinder
NC_015573	806820	807245	30	7	0.62	0.59	36.00	0.32	16.00	0.25	-4.80	0.00	0.00	27.00	34.40	CRISPRCasFinder
NC_015573	815813	817443	30	25	0.75	0.62	36.71	0.31	39.00	0.34	-4.80	0.00	0.00	27.00	34.40	CRISPRCasFinder
NC_011766	768270	768816	24	9	0.75	0.67	41.38	0.34	13.00	0.21	-0.40	0.00	0.00	21.40	23.30	CRISPRCasFinder
NC_011529	828033	828820	28	12	0.59	0.60	41.09	0.33	40.00	0.00	-1.10	0.00	0.00	15.90	15.90	CRISPRCasFinder
NC_021044	3438812	3439791	37	13	0.52	0.62	41.58	0.33	77.00	0.54	-5.60	89.03	0.00	14.00	0.00	CRISPRCasFinder
NC_021044	3437922	3438398	37	8	0.70	0.59	25.71	0.30	19.00	0.33	-4.90	98.66	0.00	14.00	0.00	CRISPRCasFinder
NC_011026	2084564	2084936	31	5	0.49	0.68	54.50	0.43	25.00	0.31	-4.10	0.00	0.00	14.00	15.90	CRISPRCasFinder
NC_007413	2727350	2727599	37	4	0.79	0.47	34.00	0.34	10.00	0.00	-7.40	0.00	0.00	15.90	15.90	CRISPRCasFinder
NZ_CP014232	625744	626575	37	12	0.77	0.49	35.27	0.34	18.00	0.17	-8.70	0.00	0.00	12.20	15.90	CRISPRCasFinder
NZ_CP014232	1225370	1225882	25	9	0.69	0.24	36.00	0.38	12.00	0.00	-13.00	0.00	0.00	19.60	19.60	CRISPRCasFinder
NZ_CP014232	639681	641023	37	19	0.78	0.50	35.56	0.32	28.00	0.30	-8.70	0.00	0.00	12.20	15.90	CRISPRCasFinder
NZ_CP014232	625014	625415	37	6	0.80	0.49	35.80	0.32	11.00	0.28	-8.70	0.00	0.00	12.20	15.90	CRISPRCasFinder
NZ_CP016463	264278	264733	29	8	0.74	0.28	32.00	0.35	17.00	0.00	-14.10	0.00	0.00	14.00	17.70	CRISPRCasFinder
NC_008578	1547941	1548204	24	6	0.62	0.25	24.00	0.40	15.00	0.28	-7.80	0.00	0.00	17.70	17.70	CRISPRCasFinder
NC_017551	1346214	1346656	24	7	0.54	0.59	45.83	0.46	22.00	0.00	-1.80	0.00	0.00	17.70	17.70	CRISPRCasFinder
NC_017551	3162190	3162505	28	5	0.73	0.54	43.75	0.44	10.00	0.00	-4.90	0.00	0.00	15.90	14.00	CRISPRCasFinder
NC_018018	2284595	2284796	25	4	0.51	0.56	34.00	0.33	15.00	0.36	0.00	0.00	0.00	12.20	15.90	CRISPRCasFinder
NC_018664	2342574	2342866	30	5	0.64	0.79	35.50	0.35	13.00	0.00	-2.10	0.00	0.00	19.60	19.60	CRISPRCasFinder
NC_010175	1636350	1636586	24	4	0.61	0.24	47.00	0.37	14.00	0.36	-5.50	0.00	0.00	14.00	14.00	CRISPRCasFinder
NC_011761	940769	941144	23	7	0.53	0.30	35.67	0.35	15.00	0.36	-4.60	0.00	0.00	12.20	15.90	CRISPRCasFinder
NC_011761	934674	934937	31	5	0.70	0.30	27.25	0.28	12.00	0.31	-10.40	0.00	0.00	14.00	15.90	CRISPRCasFinder
NC_013410	1905308	1905597	37	4	0.76	0.53	47.00	0.29	15.00	0.61	-7.20	0.00	0.00	15.90	15.90	CRISPRCasFinder
NC_021658	10090890	10091091	25	4	0.53	0.11	34.00	0.36	15.00	0.61	-9.10	0.00	0.00	14.00	14.00	CRISPRCasFinder
NC_021658	6751541	6751757	23	4	0.70	0.22	41.67	0.41	10.00	0.00	-7.40	0.00	0.00	0.00	14.00	CRISPRCasFinder
NC_021658	6104618	6105071	25	7	0.58	0.38	46.50	0.41	20.00	0.25	-2.70	0.00	0.00	12.20	0.00	CRISPRCasFinder
NC_021658	9128341	9128665	24	6	0.51	0.16	36.00	0.45	35.00	0.74	-11.00	0.00	0.00	12.20	0.00	CRISPRCasFinder
NC_021658	9128740	9128910	24	4	0.62	0.16	25.00	0.57	20.00	0.36	-12.30	0.00	0.00	14.00	14.00	CRISPRCasFinder
NC_011891	4616082	4616221	23	4	0.73	0.51	16.00	0.31	6.00	0.00	-2.60	0.00	0.00	15.90	15.90	CRISPRCasFinder

NC_010655	2507588	2507825	33	4	0.77	0.45	35.33	0.27	12.00	0.36	-14.80	0.00	0.00	27.00	32.50	CRISPRCasFinder
NC_018690	1567388	1567554	23	4	0.52	0.68	25.00	0.33	10.00	0.00	-0.10	0.00	0.00	17.70	17.70	CRISPRCasFinder
NC_018690	1566809	1567243	23	9	0.52	0.66	28.38	0.42	28.00	0.31	-0.80	0.00	0.00	14.00	14.00	CRISPRCasFinder
NC_013922	1647603	1649414	30	28	0.69	0.49	36.00	0.32	61.00	0.36	-4.70	0.00	0.00	30.70	30.70	CRISPRCasFinder
NC_015562	1262543	1262793	37	4	0.80	0.68	34.33	0.33	8.00	0.00	-5.60	0.00	0.00	32.50	32.50	CRISPRCasFinder
NC_013715	1379239	1380218	36	14	0.62	0.54	36.54	0.32	59.00	0.39	-9.00	0.00	0.00	17.70	17.70	CRISPRCasFinder
NC_013715	1380328	1382397	36	29	0.77	0.52	36.64	0.32	82.00	0.38	-9.00	0.00	0.00	17.70	17.70	CRISPRCasFinder
NC_018224	305689	305981	32	5	0.78	0.48	33.25	0.34	7.00	0.00	-10.20	0.00	0.00	39.90	39.90	CRISPRCasFinder
NC_009635	86949	87164	24	4	0.71	0.68	40.00	0.46	6.00	0.36	-1.00	0.00	0.00	17.70	19.60	CRISPRCasFinder
NC_015850	1236314	1236999	28	12	0.75	0.27	31.82	0.30	21.00	0.00	-13.10	73.67	0.00	19.60	19.60	CRISPRCasFinder
NZ_CP009518	1917044	1917443	25	7	0.54	0.67	37.50	0.34	21.00	0.25	-1.10	0.00	0.00	25.10	41.70	CRISPRCasFinder
NZ_CP009518	1932091	1932454	29	6	0.51	0.63	37.80	0.33	23.00	0.28	-4.60	0.00	0.00	21.40	30.70	CRISPRCasFinder
NZ_CP009518	1932542	1933098	29	9	0.62	0.65	37.00	0.32	27.00	0.31	-2.50	0.00	0.00	25.10	43.60	CRISPRCasFinder
NZ_CP006933	626297	626748	44	5	0.52	0.68	58.00	0.46	38.00	0.00	-4.30	0.00	0.00	17.70	17.70	CRISPRCasFinder
NC_007355	3090113	3090480	23	6	0.59	0.57	46.00	0.41	15.00	0.00	-1.90	0.00	0.00	14.00	14.00	CRISPRCasFinder
NC_021009	711854	712215	23	6	0.71	0.54	44.80	0.39	6.00	0.00	0.00	0.00	0.00	23.30	43.60	CRISPRCasFinder
NC_013887	926377	926684	31	5	0.78	0.62	38.25	0.32	8.00	0.39	-3.50	0.00	0.00	19.60	25.10	CRISPRCasFinder
NC_013887	60056	60377	32	5	0.73	0.60	40.50	0.31	16.00	0.39	-3.80	0.00	0.00	25.10	27.00	CRISPRCasFinder
NC_009033	602741	603023	25	5	0.80	0.66	39.50	0.33	4.00	0.00	0.00	0.00	0.00	21.40	25.10	CRISPRCasFinder
NC_013716	3224981	3225437	31	8	0.66	0.38	29.86	0.31	27.00	0.00	-15.10	0.00	0.00	47.30	47.30	CRISPRCasFinder
NC_009073	264904	265204	24	5	0.71	0.51	45.25	0.34	7.00	0.00	-7.90	0.00	0.00	14.00	15.90	CRISPRCasFinder
NZ_CP016077	4342575	4342785	28	4	0.78	0.23	33.00	0.35	4.00	0.00	-13.80	0.00	0.00	25.10	25.10	CRISPRCasFinder
NC_015388	1341298	1341672	55	4	0.79	0.65	51.67	0.31	12.00	0.36	-12.10	0.00	0.00	15.90	17.70	CRISPRCasFinder
NZ_CP012268	3202305	3202752	28	8	0.79	0.45	32.00	0.30	10.00	0.00	-9.10	0.00	0.00	41.70	41.70	CRISPRCasFinder
NZ_CP019701	2197783	2198013	32	4	0.76	0.38	34.33	0.31	6.00	0.00	-13.30	0.00	0.00	30.70	36.20	CRISPRCasFinder
NC_008611	5226864	5227325	25	9	0.52	0.20	29.50	0.43	41.00	0.58	-6.10	0.00	0.00	12.20	0.00	CRISPRCasFinder
NC_008611	4591961	4592300	25	6	0.46	0.34	38.00	0.42	30.00	0.53	-0.20	0.00	0.00	14.00	0.00	CRISPRCasFinder
NC_008611	5439250	5439807	24	12	0.51	0.17	24.55	0.33	56.00	0.65	-6.60	98.46	0.00	12.20	0.00	CRISPRCasFinder
NC_014330	2008853	2009476	24	9	0.57	0.73	51.00	0.46	28.00	0.00	0.00	0.00	0.00	14.00	15.90	CRISPRCasFinder
NC_016816	3919613	3920835	23	26	0.55	0.34	25.00	0.37	76.00	0.09	-5.40	0.00	0.00	17.70	17.70	CRISPRCasFinder
NZ_CP014774	1704936	1705128	23	4	0.65	0.35	33.67	0.40	8.00	0.00	-3.10	0.00	0.00	14.00	14.00	CRISPRCasFinder
NC_009925	2028067	2028372	30	5	0.44	0.49	39.00	0.37	23.00	0.00	-3.10	0.00	0.00	14.00	19.60	CRISPRCasFinder
NZ_CP012898	2754514	2754782	26	4	0.62	0.50	55.00	0.48	10.00	0.00	0.00	0.00	0.00	0.00	15.90	CRISPRCasFinder
NZ_CP008953	4801165	4801821	42	7	0.58	0.32	60.50	0.44	35.00	0.25	-17.20	0.00	0.00	17.70	15.90	CRISPRCasFinder
NZ_CP008953	7960213	7960448	23	4	0.61	0.21	48.00	0.47	14.00	0.00	-6.00	0.00	-14.40	0.00	0.00	CRISPRCasFinder
NZ_CP018786	980347	980569	25	4	0.57	0.57	41.00	0.36	16.00	0.00	-3.10	0.00	0.00	14.00	14.00	CRISPRCasFinder
NZ_CP018786	1803396	1803616	26	4	0.52	0.60	39.00	0.39	15.00	0.36	-0.20	0.00	0.00	12.20	14.00	CRISPRCasFinder
NZ_CP007056	386501	386753	37	4	0.75	0.48	35.00	0.33	13.00	0.00	-3.50	0.00	0.00	19.60	21.40	CRISPRCasFinder
NC_012225	891967	892590	24	9	0.57	0.73	51.00	0.46	28.00	0.00	0.00	0.00	0.00	14.00	15.90	CRISPRCasFinder
NC_012225	1412596	1412928	24	6	0.50	0.53	37.80	0.38	25.00	0.59	-5.80	75.47	3.50	14.00	14.00	CRISPRCasFinder
NC_008212	1383663	1383887	25	4	0.62	0.57	41.67	0.37	7.00	0.00	-6.00	0.00	0.00	34.40	34.40	CRISPRCasFinder
NC_015312	842912	843105	23	4	0.49	0.14	34.00	0.44	15.00	0.61	-7.60	0.00	0.00	12.20	0.00	CRISPRCasFinder
NC_013222	921305	921681	47	5	0.52	0.38	35.50	0.42	40.00	0.31	-12.10	0.00	0.00	14.00	0.00	CRISPRCasFinder
NC_019962	1830105	1830332	28	4	0.60	0.51	38.67	0.27	14.00	0.00	-1.00	0.00	0.00	25.10	25.10	CRISPRCasFinder
NZ_CP010905	1740903	1741458	29	9	1.00	0.83	36.88	0.37	0.00	0.37	-4.00	0.00	0.00	36.20	36.20	CRISPRCasFinder
NZ_CP010905	1920107	1921120	29	16	0.98	0.79	36.67	0.34	1.00	0.38	-2.10	62.46	0.00	47.30	47.30	CRISPRCasFinder
NZ_CP010905	2647541	2648492	29	15	0.96	0.79	36.93	0.33	4.00	0.38	0.00	0.00	0.00	30.70	30.70	CRISPRCasFinder

NZ_CP010905	1419380	1420263	29	14	0.98	0.76	36.77	0.36	1.00	0.37	-2.30	0.00	0.00	54.70	54.70	CRISPRCasFinder
NZ_CP010905	2892089	2892514	29	7	1.00	0.83	37.17	0.33	0.00	0.36	0.00	0.00	0.00	36.20	36.20	CRISPRCasFinder
NZ_CP010905	3440011	3441286	29	20	0.87	0.80	36.63	0.32	18.00	0.37	0.00	0.00	0.00	52.80	52.80	CRISPRCasFinder
NC_015680	693109	695095	30	30	0.99	0.67	37.48	0.30	1.00	0.31	-0.80	0.00	0.00	45.40	56.50	CRISPRCasFinder
NC_015680	556584	557635	30	16	1.00	0.67	38.13	0.31	0.00	0.32	-0.80	0.00	0.00	45.40	56.50	CRISPRCasFinder
NC_005090	1531926	1533495	37	22	1.00	0.57	36.00	0.32	0.00	0.33	-4.30	0.00	0.00	12.20	15.90	CRISPRCasFinder
NZ_CP009552	977342	977572	30	4	1.00	0.63	37.00	0.42	0.00	0.36	-2.70	0.00	0.00	25.10	32.50	CRISPRCasFinder
NZ_CP009552	1354028	1355001	30	15	0.98	0.63	37.43	0.32	1.00	0.33	-2.70	0.00	0.00	19.60	34.40	CRISPRCasFinder
NZ_CP009552	1644186	1644490	32	5	0.95	0.63	36.25	0.37	3.00	0.39	-2.70	0.00	0.00	25.10	32.50	CRISPRCasFinder
NZ_CP015577	552511	553395	30	14	0.95	0.77	35.77	0.35	6.00	0.30	-3.00	0.00	0.00	17.70	19.60	CRISPRCasFinder
NC_007426	165989	166216	30	4	1.00	0.47	36.00	0.32	0.00	0.36	-8.50	0.00	0.00	56.50	56.50	CRISPRCasFinder
NC_015703	5772288	5784494	30	185	1.00	0.67	36.18	0.31	4.00	0.35	-0.90	99.22	0.00	30.70	56.50	CRISPRCasFinder
NC_015703	4509667	4510731	37	15	1.00	0.59	36.43	0.33	0.00	0.38	-5.40	72.13	0.00	19.60	19.60	CRISPRCasFinder
NC_014960	3137389	3146252	37	121	0.94	0.46	36.56	0.32	55.00	0.41	-7.40	88.03	0.00	15.90	15.90	CRISPRCasFinder
NC_014960	757015	762020	37	69	0.98	0.48	36.07	0.31	12.00	0.33	-8.00	62.84	0.00	15.90	19.60	CRISPRCasFinder
NZ_CP014352	1700309	1701722	36	20	0.93	0.37	36.53	0.34	7.00	0.38	-17.30	97.59	0.00	15.90	15.90	CRISPRCasFinder
NZ_CP014352	1693660	1699113	36	76	0.99	0.36	36.23	0.34	3.00	0.36	-17.30	93.09	0.00	15.90	15.90	CRISPRCasFinder
NC_021355	134105	150352	30	246	0.99	0.77	36.20	0.33	5.00	0.36	-0.50	71.88	0.00	23.30	43.60	CRISPRCasFinder
NC_021169	1129025	1129258	31	4	0.87	0.68	36.67	0.33	4.00	0.36	-0.80	0.00	0.00	23.30	45.40	CRISPRCasFinder
NC_015975	1047155	1048214	36	15	0.96	0.59	37.14	0.35	7.00	0.38	-7.30	91.81	0.00	14.00	15.90	CRISPRCasFinder
NC_015975	1066225	1068633	30	37	0.99	0.73	36.08	0.32	7.00	0.36	0.00	0.00	0.00	28.80	28.80	CRISPRCasFinder
NC_014652	2722390	2725707	29	51	0.99	0.65	36.78	0.32	1.00	0.37	0.00	0.00	0.00	25.10	27.00	CRISPRCasFinder
NC_014652	2733415	2733967	29	9	1.00	0.52	36.50	0.32	0.00	0.37	-7.20	0.00	0.00	19.60	32.50	CRISPRCasFinder
NC_014652	2686105	2687448	30	21	0.94	0.54	35.70	0.33	7.00	0.31	-5.60	98.62	0.00	19.60	34.40	CRISPRCasFinder
NC_014652	2613496	2618702	29	80	1.00	0.69	36.54	0.32	1.00	0.39	0.00	0.00	0.00	19.60	21.40	CRISPRCasFinder
NC_014962	3141461	3167461	36	360	1.00	0.36	36.33	0.32	2.00	0.38	-14.40	98.99	0.00	19.60	19.60	CRISPRCasFinder
NC_015931	492539	492783	25	4	0.92	0.67	48.33	0.27	3.00	0.36	-0.50	0.00	0.00	21.40	25.10	CRISPRCasFinder
NC_016640	5189874	5191453	35	23	1.00	0.57	35.23	0.31	0.00	0.33	-4.90	0.00	0.00	14.00	19.60	CRISPRCasFinder
NC_016640	2903090	2904079	35	14	0.90	0.66	38.46	0.32	12.00	0.30	-1.90	0.00	0.00	14.00	15.90	CRISPRCasFinder
NC_014408	224060	226411	30	36	1.00	0.70	36.34	0.30	0.00	0.38	-3.00	90.23	0.00	25.10	49.10	CRISPRCasFinder
NC_000909	507264	508047	30	12	0.95	0.61	38.55	0.35	4.00	0.37	-3.70	0.00	0.00	52.80	52.80	CRISPRCasFinder
NC_000909	1575519	1576383	30	13	0.90	0.70	39.58	0.35	9.00	0.31	-2.70	0.00	0.00	51.00	51.00	CRISPRCasFinder
NC_000909	1049373	1050236	30	13	0.81	0.66	39.50	0.34	25.00	0.40	-3.70	0.00	0.00	49.10	49.10	CRISPRCasFinder
NC_000909	501491	501989	30	8	0.90	0.61	37.00	0.25	6.00	0.33	-4.10	0.00	0.00	25.10	27.00	CRISPRCasFinder
NC_000909	1034820	1035620	31	12	0.97	0.61	39.00	0.35	2.00	0.33	-4.10	0.00	0.00	27.00	28.80	CRISPRCasFinder
NC_000909	351694	352468	30	12	0.94	0.57	37.73	0.32	3.00	0.37	-4.20	50.00	0.00	25.10	25.10	CRISPRCasFinder
NC_021353	930280	937725	31	111	1.00	0.74	36.41	0.32	0.00	0.37	-1.90	0.00	0.00	17.70	17.70	CRISPRCasFinder
NZ_CP009503	3192212	3196902	30	71	1.00	0.63	36.59	0.32	0.00	0.39	-2.70	0.00	0.00	25.10	32.50	CRISPRCasFinder
NZ_CP009503	3179668	3183450	37	52	0.99	0.62	36.45	0.32	10.00	0.41	-2.60	97.34	0.00	38.10	38.10	CRISPRCasFinder
NC_019792	3413161	3421255	30	124	0.99	0.43	35.57	0.32	9.00	0.33	-8.50	66.85	0.00	51.00	51.00	CRISPRCasFinder
NC_020055	118127	118736	36	9	0.93	0.59	35.75	0.32	7.00	0.31	-5.10	0.00	0.00	19.60	19.60	CRISPRCasFinder
NC_019751	2038085	2039287	37	17	1.00	0.65	35.88	0.32	0.00	0.31	-7.80	0.00	0.00	23.30	28.80	CRISPRCasFinder
NC_019751	328043	328873	37	12	0.93	0.67	35.18	0.33	6.00	0.33	-7.10	94.13	0.00	17.70	17.70	CRISPRCasFinder
NC_019751	1621155	1621703	37	8	0.88	0.49	36.14	0.32	6.00	0.33	-18.40	0.00	0.00	17.70	17.70	CRISPRCasFinder
NC_019751	324319	325428	35	16	1.00	0.66	36.67	0.32	0.00	0.32	-5.20	0.00	0.00	15.90	15.90	CRISPRCasFinder
NC_019751	316560	317384	35	12	1.00	0.69	36.82	0.35	0.00	0.37	-5.40	0.00	0.00	17.70	17.70	CRISPRCasFinder
NC_010803	1009935	1011874	35	27	0.98	0.69	38.27	0.32	6.00	0.30	-0.70	63.47	0.00	21.40	25.10	CRISPRCasFinder

NC_010803	499776	508012	30	124	1.00	0.70	36.72	0.31	2.00	0.39	-1.90	99.70	0.00	21.40	27.00	CRISPRCasFinder
NC_013158	1415738	1419119	30	52	0.99	0.40	35.73	0.34	2.00	0.30	-5.00	89.23	0.00	45.40	45.40	CRISPRCasFinder
NC_017034	1139126	1145088	37	82	1.00	0.62	36.16	0.30	1.00	0.36	-7.70	97.86	0.00	28.80	28.80	CRISPRCasFinder
NC_000961	1117805	1118970	30	18	0.98	0.73	36.82	0.32	4.00	0.34	-2.00	0.00	0.00	56.50	56.50	CRISPRCasFinder
NZ_CP009505	3675722	3677662	37	27	0.98	0.59	36.19	0.31	3.00	0.37	-3.40	0.00	0.00	38.10	38.10	CRISPRCasFinder
NZ_CP009505	2794713	2800216	37	76	0.98	0.60	35.89	0.31	10.00	0.31	-3.30	0.00	0.00	38.10	38.10	CRISPRCasFinder
NZ_CP009505	2803925	2804183	37	4	0.80	0.58	36.67	0.29	9.00	0.36	-3.30	0.00	0.00	38.10	38.10	CRISPRCasFinder
NZ_CP009505	3725626	3726178	37	8	1.00	0.62	36.71	0.29	0.00	0.33	-2.60	0.00	0.00	38.10	38.10	CRISPRCasFinder
NZ_CP009505	3717699	3718685	37	14	0.99	0.59	36.08	0.32	1.00	0.34	-3.20	69.13	0.00	41.70	41.70	CRISPRCasFinder
NZ_CP009505	3680371	3688683	37	114	1.00	0.59	36.24	0.30	0.00	0.37	-3.40	95.54	0.00	38.10	38.10	CRISPRCasFinder
NZ_CP009505	3702192	3705176	37	41	0.98	0.60	36.70	0.32	12.00	0.38	-3.20	95.25	0.00	41.70	41.70	CRISPRCasFinder
NZ_CP009505	3677799	3678130	37	5	1.00	0.59	36.75	0.34	0.00	0.39	-3.40	0.00	0.00	38.10	38.10	CRISPRCasFinder
NZ_CP009505	2277759	2281756	37	55	0.94	0.64	36.35	0.30	39.00	0.36	-2.80	0.00	0.00	38.10	38.10	CRISPRCasFinder
NZ_CP012024	2797028	2798305	29	20	1.00	0.66	36.74	0.31	0.00	0.39	0.00	0.00	0.00	21.40	21.40	CRISPRCasFinder
NZ_CP012024	1860018	1860511	30	8	1.00	0.77	36.29	0.31	0.00	0.38	0.00	0.00	0.00	39.90	45.40	CRISPRCasFinder
NZ_CP012024	1914384	1914813	30	7	0.97	0.74	36.67	0.32	3.00	0.39	0.00	0.00	0.00	45.40	51.00	CRISPRCasFinder
NZ_CP012024	2794821	2796952	29	33	1.00	0.66	36.69	0.32	0.00	0.39	0.00	66.52	0.00	21.40	21.40	CRISPRCasFinder
NZ_CP012024	1857304	1857532	30	4	0.86	0.75	36.33	0.37	6.00	0.36	0.00	0.00	0.00	39.90	45.40	CRISPRCasFinder
NC_018870	2443641	2445938	30	35	0.94	0.58	36.71	0.31	9.00	0.37	-2.60	0.00	0.00	41.70	56.50	CRISPRCasFinder
NC_018870	2453564	2455337	30	27	1.00	0.60	37.04	0.30	0.00	0.39	-2.50	0.00	0.00	45.40	56.50	CRISPRCasFinder
NC_018870	1915104	1923411	37	113	1.00	0.54	36.85	0.32	1.00	0.38	-6.50	95.71	0.00	30.70	30.70	CRISPRCasFinder
NC_018870	2463808	2464503	30	11	1.00	0.60	36.60	0.28	0.00	0.38	-2.50	0.00	0.00	45.40	56.50	CRISPRCasFinder
NC_018870	2455744	2458157	30	37	0.98	0.60	36.22	0.30	9.00	0.37	-2.50	0.00	0.00	45.40	56.50	CRISPRCasFinder
NC_018870	2449772	2451060	30	20	1.00	0.60	36.26	0.30	0.00	0.37	-2.50	0.00	0.00	45.40	56.50	CRISPRCasFinder
NC_018870	1072199	1072754	37	8	0.96	0.54	37.14	0.31	4.00	0.38	-8.40	0.00	0.00	30.70	30.70	CRISPRCasFinder
NC_018870	2204611	2211549	30	105	1.00	0.57	36.43	0.31	0.00	0.38	-3.90	0.00	0.00	38.10	51.00	CRISPRCasFinder
NC_018870	2439391	2440146	30	12	0.92	0.58	36.00	0.30	10.00	0.37	-2.60	0.00	0.00	41.70	56.50	CRISPRCasFinder
NC_018870	2460106	2463402	30	50	1.00	0.60	36.65	0.31	0.00	0.39	-2.50	97.95	0.00	45.40	56.50	CRISPRCasFinder
NC_019776	4113500	4114042	37	8	1.00	0.70	35.29	0.35	0.00	0.33	-0.60	0.00	0.00	21.40	34.40	CRISPRCasFinder
NC_019776	4073710	4074460	37	10	1.00	0.49	42.33	0.32	0.00	0.38	-8.10	0.00	0.00	28.80	28.80	CRISPRCasFinder
NC_019776	3183114	3183517	37	6	0.96	0.73	36.40	0.30	1.00	0.38	-3.50	0.00	0.00	27.00	38.10	CRISPRCasFinder
NC_019776	1064438	1065555	37	15	1.00	0.51	40.21	0.32	0.00	0.37	-9.80	83.52	0.00	28.80	28.80	CRISPRCasFinder
NC_019776	3180046	3182768	37	38	0.99	0.73	35.57	0.33	8.00	0.31	-3.50	0.00	0.00	27.00	38.10	CRISPRCasFinder
NC_017584	2715965	2716300	37	5	0.90	0.44	37.75	0.31	2.00	0.39	-15.30	0.00	0.00	17.70	17.70	CRISPRCasFinder
NC_017584	213598	214173	37	8	0.97	0.44	40.00	0.33	1.00	0.44	-15.60	0.00	0.00	17.70	17.70	CRISPRCasFinder
NC_017461	61768	62836	24	18	0.99	0.71	37.47	0.32	1.00	0.30	-1.30	0.00	0.00	25.10	45.40	CRISPRCasFinder
NC_017461	13013	14144	24	19	1.00	0.67	37.56	0.33	0.00	0.33	-4.60	0.00	0.00	25.10	34.40	CRISPRCasFinder
NC_017461	55133	56194	24	18	0.96	0.71	37.06	0.31	3.00	0.36	0.00	0.00	0.00	25.10	45.40	CRISPRCasFinder
NC_015636	1629402	1633833	30	66	0.99	0.67	37.71	0.35	4.00	0.33	-4.40	92.09	0.00	27.00	34.40	CRISPRCasFinder
NC_015636	1529273	1531189	30	29	1.00	0.67	37.39	0.37	1.00	0.38	-4.40	97.66	0.00	27.00	34.40	CRISPRCasFinder
NC_015636	225510	230898	30	81	0.99	0.67	36.99	0.34	2.00	0.37	-4.40	89.09	0.00	27.00	34.40	CRISPRCasFinder
NC_015636	224644	225433	30	12	1.00	0.67	39.00	0.35	0.00	0.52	-4.40	81.03	9.70	27.00	34.40	CRISPRCasFinder
NC_015636	103466	105101	30	25	1.00	0.67	36.92	0.33	0.00	0.38	-2.00	91.77	0.00	27.00	34.40	CRISPRCasFinder
NC_015636	206578	207146	30	9	0.98	0.67	37.38	0.34	2.00	0.31	-4.40	0.00	0.00	25.10	32.50	CRISPRCasFinder
NC_015636	1302586	1304621	30	31	1.00	0.67	36.87	0.35	0.00	0.38	-2.00	95.98	0.00	27.00	34.40	CRISPRCasFinder
NC_018227	2627396	2637968	36	145	1.00	0.56	37.17	0.31	4.00	0.36	-12.30	66.98	0.00	15.90	15.90	CRISPRCasFinder
NC_009135	742879	744906	37	28	1.00	0.73	36.74	0.32	0.00	0.37	-1.80	0.00	0.00	69.40	69.40	CRISPRCasFinder

NC_014122	245939	247616	37	23	0.99	0.59	37.59	0.35	1.00	0.38	-5.60	0.00	0.00	32.50	32.50	CRISPRCasFinder
NC_014122	767940	768493	37	8	0.97	0.63	36.86	0.33	3.00	0.38	-5.60	0.00	0.00	32.50	32.50	CRISPRCasFinder
NC_014122	531383	532158	37	11	1.00	0.59	36.90	0.32	0.00	0.39	-5.10	0.00	0.00	32.50	32.50	CRISPRCasFinder
NC_014122	1111056	1111998	37	13	1.00	0.59	38.50	0.33	0.00	0.46	-5.60	0.00	0.00	32.50	32.50	CRISPRCasFinder
NC_014122	1295745	1295973	30	4	1.00	0.53	36.33	0.27	0.00	0.36	-6.20	0.00	0.00	15.90	21.40	CRISPRCasFinder
NC_014122	604543	605764	37	17	1.00	0.62	37.06	0.33	0.00	0.37	-5.60	0.00	0.00	32.50	32.50	CRISPRCasFinder
NC_014122	235755	236383	30	10	0.92	0.54	36.56	0.36	5.00	0.38	-5.00	0.00	0.00	15.90	21.40	CRISPRCasFinder
NC_014122	1082729	1083283	37	8	0.95	0.60	37.00	0.32	7.00	0.38	-5.60	0.00	0.00	32.50	32.50	CRISPRCasFinder
NC_014122	826017	826802	30	12	0.92	0.53	38.73	0.33	4.00	0.37	-5.00	0.00	0.00	15.90	21.40	CRISPRCasFinder
NC_003551	1362504	1363454	37	11	0.90	0.62	54.40	0.38	13.00	0.38	-5.40	0.00	0.00	14.00	15.90	CRISPRCasFinder
NC_003551	1297128	1297617	36	6	0.83	0.61	54.80	0.34	11.00	0.38	-10.60	0.00	0.00	14.00	0.00	CRISPRCasFinder
NC_000916	983373	991584	30	124	1.00	0.70	36.52	0.29	0.00	0.38	-2.50	76.69	0.00	25.10	49.10	CRISPRCasFinder
NC_000916	1472442	1475513	30	47	1.00	0.70	36.13	0.29	0.00	0.39	-3.00	96.80	0.00	25.10	49.10	CRISPRCasFinder
NZ_CP012159	7095638	7099260	36	51	0.96	0.39	35.74	0.33	13.00	0.36	-6.30	0.00	0.00	15.90	0.00	CRISPRCasFinder
NZ_CP012159	7109204	7114398	36	73	0.96	0.39	35.65	0.34	39.00	0.38	-7.90	99.05	0.00	15.90	0.00	CRISPRCasFinder
NZ_CP012159	8803502	8803833	37	5	1.00	0.35	36.75	0.35	0.00	0.31	-12.20	0.00	0.00	19.60	25.10	CRISPRCasFinder
NZ_CP012159	4524009	4524861	37	12	0.98	0.35	37.18	0.33	1.00	0.37	-12.20	0.00	0.00	19.60	25.10	CRISPRCasFinder
NC_018024	1096374	1098139	30	27	1.00	0.63	36.77	0.30	0.00	0.39	-2.50	0.00	0.00	23.30	39.90	CRISPRCasFinder
NC_018024	1107551	1113295	30	87	1.00	0.60	36.45	0.30	2.00	0.37	-2.50	97.29	0.00	28.80	39.90	CRISPRCasFinder
NC_018024	1604244	1609325	30	77	1.00	0.60	36.47	0.31	0.00	0.38	-2.30	98.31	0.00	23.30	34.40	CRISPRCasFinder
NC_013790	1036397	1037151	30	12	1.00	0.70	35.91	0.34	0.00	0.37	-2.50	0.00	0.00	32.50	56.50	CRISPRCasFinder
NZ_CP007140	457101	457669	30	9	0.97	0.63	37.38	0.30	2.00	0.31	-0.80	0.00	0.00	28.80	56.50	CRISPRCasFinder
NZ_CP007140	604299	606074	30	27	0.96	0.63	37.15	0.29	4.00	0.39	-0.80	96.75	0.00	28.80	56.50	CRISPRCasFinder
NZ_CP007140	1798198	1800241	30	31	0.98	0.63	37.13	0.30	4.00	0.32	-0.50	0.00	0.00	34.40	51.00	CRISPRCasFinder
NZ_CP016804	1201311	1203838	30	39	1.00	0.50	35.74	0.33	1.00	0.31	-5.00	0.00	0.00	39.90	39.90	CRISPRCasFinder
NC_014098	2770187	2778650	30	126	1.00	0.50	37.47	0.32	0.00	0.30	-1.60	85.70	0.00	30.70	36.20	CRISPRCasFinder
NC_014098	2703853	2705077	37	17	1.00	0.27	37.25	0.34	0.00	0.37	-17.30	0.00	0.00	14.00	17.70	CRISPRCasFinder
NC_014098	2714103	2715039	38	13	0.80	0.30	36.92	0.33	24.00	0.38	-19.20	0.00	0.00	15.90	17.70	CRISPRCasFinder
NC_014098	2750070	2768077	30	266	1.00	0.50	37.84	0.32	3.00	0.31	-1.60	93.40	0.00	30.70	36.20	CRISPRCasFinder
NC_015954	190284	194678	37	61	0.99	0.46	35.63	0.33	3.00	0.30	-3.50	0.00	0.00	19.60	21.40	CRISPRCasFinder
NZ_CP011267	273569	274405	30	13	0.92	0.44	37.25	0.31	9.00	0.36	0.00	0.00	0.00	17.70	17.70	CRISPRCasFinder
NZ_CP011267	795283	796727	30	22	1.00	0.73	37.38	0.33	0.00	0.33	-1.00	0.00	0.00	36.20	39.90	CRISPRCasFinder
NC_006624	468981	470566	30	24	0.96	0.68	37.65	0.31	8.00	0.32	-0.90	0.00	0.00	32.50	54.70	CRISPRCasFinder
NC_007681	1091284	1095037	30	57	1.00	0.83	36.48	0.35	9.00	0.39	0.00	87.08	0.00	19.60	25.10	CRISPRCasFinder
NC_007681	490169	494226	29	62	0.99	0.79	37.05	0.36	7.00	0.36	-0.70	97.83	0.00	17.70	17.70	CRISPRCasFinder
NC_012804	1221278	1222718	30	22	0.97	0.63	37.19	0.30	4.00	0.31	-0.20	0.00	0.00	28.80	56.50	CRISPRCasFinder
NC_012804	208373	208937	30	9	0.85	0.63	36.88	0.29	21.00	0.39	-0.80	0.00	0.00	28.80	56.50	CRISPRCasFinder
NZ_CP014672	1533576	1535710	30	33	1.00	0.77	35.78	0.31	0.00	0.33	-0.30	0.00	0.00	17.70	25.10	CRISPRCasFinder
NC_014152	2550336	2554327	30	60	0.97	0.63	37.15	0.31	10.00	0.37	-1.00	0.00	0.00	34.40	56.50	CRISPRCasFinder
NC_014152	2567569	2571266	30	56	0.97	0.63	36.67	0.31	6.00	0.39	-1.00	0.00	0.00	34.40	56.50	CRISPRCasFinder
NC_014152	2575925	2582292	30	95	1.00	0.63	37.43	0.31	4.00	0.34	-1.00	52.31	0.00	34.40	56.50	CRISPRCasFinder
NC_014152	2078344	2080300	36	27	1.00	0.53	37.88	0.33	1.00	0.30	-13.70	70.92	0.00	30.70	30.70	CRISPRCasFinder
NC_014152	2572406	2572636	30	4	0.88	0.63	37.00	0.27	5.00	0.36	-1.00	0.00	0.00	34.40	56.50	CRISPRCasFinder
NC_017275	753266	754107	24	15	0.94	0.71	34.43	0.34	9.00	0.46	0.00	0.00	0.00	15.90	15.90	CRISPRCasFinder
NZ_CP012098	3066058	3066991	30	14	0.84	0.81	39.54	0.31	22.00	0.56	0.00	0.00	0.00	19.60	27.00	CRISPRCasFinder
NZ_CP011266	1304078	1304554	37	7	0.91	0.74	36.17	0.33	4.00	0.36	-1.50	0.00	0.00	27.00	27.00	CRISPRCasFinder
NZ_CP009516	2392664	2394112	37	20	0.96	0.62	37.32	0.35	4.00	0.38	-2.80	0.00	0.00	36.20	39.90	CRISPRCasFinder

NZ_CP009516	2400559	2403809	37	45	1.00	0.62	36.05	0.32	0.00	0.36	-2.60	93.39	0.00	38.10	38.10	CRISPRCasFinder
NC_011295	1263870	1264966	30	17	0.98	0.60	36.69	0.31	1.00	0.38	-2.00	97.78	0.00	23.30	45.40	CRISPRCasFinder
NC_012034	164244	165518	29	20	0.92	0.66	36.58	0.34	17.00	0.39	-2.70	0.00	0.00	25.10	27.00	CRISPRCasFinder
NC_012034	2856808	2857631	30	13	0.99	0.50	36.17	0.32	1.00	0.31	-7.20	0.00	0.00	19.60	34.40	CRISPRCasFinder
NC_012034	151654	160400	29	134	1.00	0.66	36.55	0.32	1.00	0.39	-2.70	69.61	0.00	25.10	27.00	CRISPRCasFinder
NC_012034	2803920	2807402	30	53	0.95	0.53	36.40	0.33	14.00	0.37	-5.60	0.00	0.00	19.60	34.40	CRISPRCasFinder
NC_012034	2828426	2831032	30	40	0.97	0.53	36.08	0.32	11.00	0.32	-7.20	0.00	0.00	19.60	32.50	CRISPRCasFinder
NC_010546	359604	359851	35	4	0.85	0.61	36.00	0.31	6.00	0.36	-3.70	0.00	0.00	21.40	21.40	CRISPRCasFinder
NC_019695	3356802	3360948	37	57	1.00	0.68	36.39	0.31	2.00	0.35	-3.30	55.15	0.00	23.30	34.40	CRISPRCasFinder
NC_014721	2731763	2732193	30	7	0.86	0.53	36.83	0.30	5.00	0.39	-4.20	0.00	0.00	19.60	32.50	CRISPRCasFinder
NC_014721	2708850	2712824	29	61	0.99	0.62	36.77	0.33	3.00	0.38	0.00	94.77	0.00	19.60	25.10	CRISPRCasFinder
NC_014721	2740676	2740907	31	4	0.97	0.49	36.00	0.29	1.00	0.36	-9.00	0.00	0.00	17.70	28.80	CRISPRCasFinder
NC_014721	2666352	2668217	30	28	0.91	0.50	38.00	0.31	19.00	0.45	-6.10	91.41	0.00	19.60	28.80	CRISPRCasFinder
NC_014721	2496982	2506688	29	148	0.97	0.66	36.84	0.33	65.00	0.37	0.00	56.48	0.00	27.00	27.00	CRISPRCasFinder
NC_014721	2669623	2673685	30	62	0.91	0.49	36.11	0.33	31.00	0.35	-6.10	0.00	0.00	19.60	28.80	CRISPRCasFinder
NC_018012	4879408	4879738	37	5	0.88	0.55	36.25	0.35	4.00	0.31	-7.80	0.00	0.00	32.50	32.50	CRISPRCasFinder
NC_018012	4881079	4881922	37	12	1.00	0.54	36.36	0.33	0.00	0.37	-7.80	0.00	0.00	32.50	32.50	CRISPRCasFinder
NC_005877	15	309	30	5	1.00	0.77	36.25	0.32	0.00	0.31	-0.80	0.00	0.00	23.30	28.80	CRISPRCasFinder
NC_005877	1540318	1545873	30	83	1.00	0.77	37.39	0.33	2.00	0.34	-0.80	74.89	0.00	23.30	28.80	CRISPRCasFinder
NC_013201	31920	34849	30	45	0.98	0.47	35.91	0.33	3.00	0.33	-3.60	0.00	0.00	51.00	51.00	CRISPRCasFinder
NZ_CP008874	840840	844503	30	56	0.99	0.40	36.07	0.32	1.00	0.35	-6.60	0.00	0.00	39.90	39.90	CRISPRCasFinder
NZ_CP012946	1769474	1770632	37	16	1.00	0.43	37.80	0.34	0.00	0.32	-10.80	0.00	0.00	15.90	15.90	CRISPRCasFinder
NZ_CP007264	705938	708770	30	43	0.98	0.60	36.74	0.31	11.00	0.36	-0.30	0.00	0.00	28.80	56.50	CRISPRCasFinder
NZ_CP007264	182361	184798	30	37	0.98	0.63	36.89	0.31	6.00	0.33	-0.20	0.00	0.00	28.80	56.50	CRISPRCasFinder
NZ_CP007264	1461759	1462999	30	19	0.99	0.60	37.28	0.31	1.00	0.33	-0.90	0.00	0.00	27.00	54.70	CRISPRCasFinder
NZ_CP007264	451542	455179	30	55	1.00	0.63	36.81	0.31	0.00	0.34	-0.30	88.84	0.00	34.40	51.00	CRISPRCasFinder
NC_012032	4543861	4550058	37	84	0.95	0.46	37.23	0.33	45.00	0.34	-9.60	99.91	0.00	17.70	19.60	CRISPRCasFinder
NC_012032	3958399	3986006	37	375	1.00	0.54	36.72	0.31	8.00	0.39	-6.40	98.82	0.00	15.90	15.90	CRISPRCasFinder
NZ_CP015520	1383163	1384693	30	23	0.95	0.64	38.23	0.32	9.00	0.42	-0.90	0.00	0.00	27.00	56.50	CRISPRCasFinder
NZ_CP014859	364941	365495	37	8	1.00	0.27	37.00	0.44	0.00	0.33	-19.60	0.00	0.00	19.60	23.30	CRISPRCasFinder
NZ_CP014859	3928451	3929219	37	11	0.93	0.32	36.20	0.34	4.00	0.34	-19.70	70.91	0.00	17.70	19.60	CRISPRCasFinder
NC_002578	846049	849106	29	47	0.99	0.72	36.85	0.32	3.00	0.38	-1.80	0.00	0.00	21.40	23.30	CRISPRCasFinder
NC_007503	1926257	1930135	30	59	0.92	0.63	36.36	0.31	49.00	0.36	-2.80	0.00	0.00	27.00	51.00	CRISPRCasFinder
NC_007503	1944006	1949572	30	84	0.99	0.63	36.71	0.31	8.00	0.38	-2.80	0.00	0.00	23.30	56.50	CRISPRCasFinder
NC_014222	339765	342408	37	36	0.99	0.65	37.49	0.34	5.00	0.38	-3.80	0.00	0.00	38.10	38.10	CRISPRCasFinder
NC_014222	539822	541857	37	28	1.00	0.68	37.04	0.34	1.00	0.38	-3.80	68.65	0.00	38.10	38.10	CRISPRCasFinder
NC_014222	582647	594279	31	172	1.00	0.71	36.85	0.33	5.00	0.39	0.00	80.39	0.00	28.80	30.70	CRISPRCasFinder
NC_020449	1486615	1498952	38	167	1.00	0.68	36.10	0.31	2.00	0.34	-2.30	75.73	0.00	32.50	32.50	CRISPRCasFinder
NC_013205	38911	40569	37	23	0.92	0.37	36.73	0.30	16.00	0.37	-13.30	54.47	0.00	15.90	15.90	CRISPRCasFinder
NC_013205	65468	66899	37	20	0.96	0.37	36.42	0.31	10.00	0.38	-13.00	0.00	0.00	15.90	15.90	CRISPRCasFinder
NC_013205	2945305	2946296	37	14	0.96	0.36	36.46	0.32	8.00	0.39	-13.10	0.00	0.00	14.00	0.00	CRISPRCasFinder
NZ_CP009524	282078	284324	37	31	0.99	0.62	36.67	0.33	1.00	0.38	-3.10	0.00	0.00	38.10	38.10	CRISPRCasFinder
NZ_CP009524	716265	727114	37	149	1.00	0.62	36.06	0.31	3.00	0.36	-2.60	97.67	0.00	38.10	38.10	CRISPRCasFinder
NC_000918	1379598	1379894	30	5	0.84	0.57	36.75	0.30	9.00	0.39	-1.90	0.00	0.00	17.70	19.60	CRISPRCasFinder
NC_000918	279264	279555	29	5	1.00	0.62	36.75	0.38	0.00	0.39	-1.20	0.00	0.00	14.00	15.90	CRISPRCasFinder
NC_000918	244561	244791	29	4	1.00	0.55	38.33	0.31	0.00	0.36	-1.30	0.00	0.00	17.70	19.60	CRISPRCasFinder
NZ_CP008887	1220049	1224832	30	72	0.99	0.63	36.96	0.30	5.00	0.34	-0.20	0.00	0.00	28.80	56.50	CRISPRCasFinder

NZ_CP008887	1720540	1721978	30	22	0.99	0.63	37.10	0.29	1.00	0.31	-0.50	0.00	0.00	34.40	51.00	CRISPRCasFinder
NZ_CP008887	446433	449955	30	53	0.98	0.63	37.17	0.31	6.00	0.35	-0.20	54.52	0.00	28.80	56.50	CRISPRCasFinder
NC_016025	984883	988557	36	51	0.99	0.56	36.78	0.34	12.00	0.36	-9.30	99.50	0.00	15.90	15.90	CRISPRCasFinder
NC_016025	420934	428142	36	101	0.99	0.56	35.73	0.32	11.00	0.33	-8.80	99.67	0.00	14.00	0.00	CRISPRCasFinder
NC_008553	1696036	1700457	37	60	1.00	0.51	37.32	0.32	1.00	0.33	-6.80	98.23	0.00	19.60	25.10	CRISPRCasFinder
NC_008553	677280	677982	37	10	1.00	0.54	37.00	0.30	0.00	0.38	-1.70	0.00	0.00	39.90	69.40	CRISPRCasFinder
NC_008553	670839	677182	37	87	1.00	0.54	36.33	0.32	0.00	0.37	-1.70	87.99	0.00	39.90	69.40	CRISPRCasFinder
NC_013939	514731	516696	30	30	0.96	0.64	36.76	0.34	6.00	0.37	-0.60	0.00	0.00	34.40	56.50	CRISPRCasFinder
NC_013939	526129	527564	30	22	0.93	0.64	36.95	0.34	10.00	0.37	0.00	0.00	0.00	39.90	56.50	CRISPRCasFinder
NZ_CP007493	1408046	1408328	25	5	0.83	0.71	39.50	0.28	4.00	0.31	0.00	0.00	0.00	19.60	19.60	CRISPRCasFinder
NZ_CP012072	1116397	1119038	37	36	0.93	0.49	37.43	0.32	16.00	0.33	-9.90	59.92	0.00	12.20	15.90	CRISPRCasFinder
NC_015711	2377930	2379923	36	27	0.95	0.31	39.31	0.37	9.00	0.53	-12.40	92.79	0.00	21.40	21.40	CRISPRCasFinder
NZ_CP006019	1003934	1005710	30	27	0.93	0.73	37.19	0.32	12.00	0.36	-0.70	0.00	0.00	27.00	43.60	CRISPRCasFinder
NZ_CP006019	1426423	1428518	30	32	0.97	0.70	36.65	0.32	7.00	0.38	-2.00	0.00	0.00	34.40	51.00	CRISPRCasFinder
NZ_CP006019	735977	739414	30	52	1.00	0.70	36.82	0.32	0.00	0.39	-0.70	0.00	0.00	28.80	45.40	CRISPRCasFinder
NC_002939	74806	77552	37	38	1.00	0.38	36.24	0.31	0.00	0.38	-14.90	82.13	0.00	15.90	15.90	CRISPRCasFinder
NC_014720	2725100	2725725	30	10	0.94	0.50	36.22	0.30	5.00	0.38	-7.20	0.00	0.00	19.60	34.40	CRISPRCasFinder
NC_014720	2663461	2675104	29	177	0.99	0.66	36.99	0.33	8.00	0.36	0.00	0.00	0.00	25.10	27.00	CRISPRCasFinder
NC_014720	2735113	2736658	30	24	0.95	0.51	35.91	0.31	7.00	0.32	-7.20	0.00	0.00	19.60	34.40	CRISPRCasFinder
NC_014720	2745478	2746034	30	9	1.00	0.50	35.88	0.29	0.00	0.31	-7.20	0.00	0.00	19.60	34.40	CRISPRCasFinder
NC_003552	4523522	4525551	30	31	0.99	0.63	36.67	0.32	1.00	0.37	-2.70	0.00	0.00	25.10	32.50	CRISPRCasFinder
NC_003552	2379328	2381265	37	27	0.96	0.59	36.12	0.33	13.00	0.36	-3.10	0.00	0.00	38.10	38.10	CRISPRCasFinder
NC_015865	1907425	1908192	30	12	0.98	0.60	37.09	0.33	2.00	0.37	-0.20	0.00	0.00	23.30	54.70	CRISPRCasFinder
NC_015865	137547	140105	29	39	0.98	0.55	37.58	0.32	4.00	0.34	-2.40	61.82	0.00	14.00	15.90	CRISPRCasFinder
NC_015865	345768	346532	30	12	0.99	0.63	36.82	0.31	1.00	0.37	-0.20	0.00	0.00	28.80	56.50	CRISPRCasFinder
NC_015865	131168	133209	29	31	0.99	0.55	38.10	0.32	4.00	0.32	-3.40	0.00	0.00	14.00	14.00	CRISPRCasFinder
NC_013315	1709876	1710232	29	6	0.84	0.79	36.60	0.37	5.00	0.38	-0.10	0.00	0.00	30.70	30.70	CRISPRCasFinder
NC_013315	3244496	3246042	29	24	0.89	0.80	37.00	0.35	18.00	0.34	0.00	0.00	0.00	52.80	52.80	CRISPRCasFinder
NC_013315	1504069	1504821	29	12	0.89	0.79	36.82	0.35	11.00	0.37	-2.10	0.00	0.00	52.80	52.80	CRISPRCasFinder
NC_013315	1615439	1615732	29	5	0.82	0.82	37.25	0.38	3.00	0.31	-4.00	0.00	0.00	36.20	36.20	CRISPRCasFinder
NC_013315	3491430	3492052	29	10	0.90	0.83	37.00	0.33	4.00	0.38	-0.80	0.00	0.00	19.60	19.60	CRISPRCasFinder
NZ_CP009149	160575	161019	30	7	1.00	0.60	39.17	0.36	0.00	0.36	-4.20	0.00	0.00	47.30	47.30	CRISPRCasFinder
NZ_CP009149	320136	321364	30	18	0.90	0.63	40.53	0.37	16.00	0.47	-4.20	0.00	0.00	49.10	49.10	CRISPRCasFinder
NZ_CP009149	784070	784580	30	8	0.87	0.61	38.71	0.37	10.00	0.33	-4.20	0.00	0.00	41.70	41.70	CRISPRCasFinder
NZ_CP009149	432544	433077	30	8	0.97	0.60	42.00	0.34	1.00	0.53	-4.20	0.00	0.00	47.30	47.30	CRISPRCasFinder
NZ_CP009149	1464714	1465476	30	12	0.96	0.61	36.55	0.33	5.00	0.33	-4.20	0.00	0.00	47.30	47.30	CRISPRCasFinder
NZ_CP009149	1466619	1466858	30	4	0.90	0.60	40.00	0.35	2.00	0.36	-3.00	0.00	0.00	41.70	41.70	CRISPRCasFinder
NZ_CP009149	152748	153244	30	8	1.00	0.60	36.71	0.34	0.00	0.38	-3.00	0.00	0.00	41.70	41.70	CRISPRCasFinder
NZ_CP009149	769284	770276	30	15	0.91	0.60	38.79	0.34	7.00	0.38	-4.20	0.00	0.00	47.30	47.30	CRISPRCasFinder
NZ_CP009149	287107	288529	30	21	0.89	0.60	39.65	0.34	14.00	0.31	-4.20	0.00	0.00	41.70	41.70	CRISPRCasFinder
NZ_CP009149	974312	974826	30	8	0.96	0.60	39.29	0.31	1.00	0.33	-3.00	0.00	0.00	41.70	41.70	CRISPRCasFinder
NZ_CP014140	3202203	3202892	29	11	0.99	0.72	37.10	0.31	1.00	0.38	-0.30	0.00	0.00	25.10	25.10	CRISPRCasFinder
NZ_CP014140	1750499	1751850	30	21	0.97	0.66	36.10	0.31	2.00	0.31	-2.60	0.00	0.00	34.40	45.40	CRISPRCasFinder
NZ_CP014140	3215204	3218530	29	51	0.99	0.66	36.96	0.31	3.00	0.36	0.00	80.12	0.00	21.40	21.40	CRISPRCasFinder
NC_019693	7038938	7043138	37	58	0.96	0.54	36.05	0.31	20.00	0.35	-9.20	84.42	0.00	19.60	23.30	CRISPRCasFinder
NC_019693	6234891	6235861	37	13	0.85	0.55	40.83	0.31	10.00	0.51	-8.90	0.00	0.00	27.00	27.00	CRISPRCasFinder
NC_019693	6251489	6253282	37	25	0.97	0.57	36.21	0.31	5.00	0.38	-11.00	92.54	0.00	27.00	27.00	CRISPRCasFinder

NC_019693	7504261	7509286	37	70	0.98	0.54	35.30	0.32	13.00	0.31	-9.20	90.73	0.00	19.60	23.30	CRISPRCasFinder
NZ_CP009508	4282906	4287833	37	68	0.99	0.65	36.00	0.31	2.00	0.35	-2.60	0.00	0.00	38.10	38.10	CRISPRCasFinder
NZ_CP009508	2768489	2769988	37	21	0.99	0.62	36.15	0.32	2.00	0.38	-2.30	98.36	0.00	36.20	36.20	CRISPRCasFinder
NZ_CP009508	2819194	2822674	37	48	0.99	0.62	36.28	0.31	5.00	0.39	-2.60	0.00	0.00	38.10	38.10	CRISPRCasFinder
NC_015573	2972297	2976244	37	54	0.99	0.43	36.79	0.31	10.00	0.39	-5.10	86.56	0.00	27.00	27.00	CRISPRCasFinder
NC_015573	484645	486681	30	31	1.00	0.53	36.90	0.32	0.00	0.39	-5.60	96.53	0.00	32.50	56.50	CRISPRCasFinder
NC_015573	488724	488957	30	4	0.96	0.53	38.00	0.35	1.00	0.36	-5.60	0.00	0.00	32.50	56.50	CRISPRCasFinder
NC_015573	2990697	2994950	37	58	0.99	0.43	36.98	0.31	3.00	0.39	-4.10	0.00	0.00	27.00	27.00	CRISPRCasFinder
NC_015573	642189	644638	29	38	0.93	0.49	36.43	0.31	9.00	0.38	-1.60	98.05	0.00	15.90	17.70	CRISPRCasFinder
NC_011529	818816	819904	29	17	0.95	0.55	37.25	0.31	5.00	0.31	-4.00	0.00	0.00	15.90	15.90	CRISPRCasFinder
NC_011529	995057	997976	30	44	0.99	0.67	37.21	0.32	2.00	0.34	-0.90	0.00	0.00	32.50	54.70	CRISPRCasFinder
NC_011529	728608	730178	30	24	1.00	0.70	37.00	0.31	1.00	0.32	-3.20	0.00	0.00	34.40	56.50	CRISPRCasFinder
NC_011529	994457	994969	30	8	0.89	0.68	38.86	0.32	7.00	0.33	-0.90	0.00	0.00	32.50	54.70	CRISPRCasFinder
NC_021044	1279053	1279340	29	5	0.96	0.70	35.75	0.34	2.00	0.31	-1.50	0.00	0.00	27.00	36.20	CRISPRCasFinder
NC_021044	1272490	1272791	29	5	0.96	0.69	39.25	0.35	1.00	0.31	-1.50	0.00	0.00	27.00	36.20	CRISPRCasFinder
NC_011026	2683900	2694522	30	161	1.00	0.63	36.21	0.31	3.00	0.36	-2.80	98.14	0.00	25.10	27.00	CRISPRCasFinder
NC_011026	2813147	2819595	30	98	0.98	0.63	36.18	0.31	8.00	0.36	-2.80	96.57	0.00	25.10	27.00	CRISPRCasFinder
NC_012883	1328782	1330412	30	25	0.98	0.73	36.71	0.30	7.00	0.39	0.00	0.00	0.00	39.90	56.50	CRISPRCasFinder
NZ_CP012152	1992597	1995072	29	38	0.99	0.62	37.14	0.33	1.00	0.37	0.00	0.00	0.00	27.00	27.00	CRISPRCasFinder
NC_007413	4821250	4823752	37	35	0.99	0.60	35.53	0.31	6.00	0.35	-8.00	80.25	0.00	23.30	34.40	CRISPRCasFinder
NC_007413	5227213	5229282	37	29	0.99	0.65	35.61	0.30	2.00	0.33	-8.10	62.23	0.00	21.40	28.80	CRISPRCasFinder
NC_009634	127424	127680	37	4	0.97	0.61	36.33	0.37	1.00	0.36	-4.40	0.00	0.00	27.00	27.00	CRISPRCasFinder
NC_009634	262748	270115	37	101	1.00	0.68	36.31	0.33	6.00	0.37	-3.40	95.05	0.00	28.80	28.80	CRISPRCasFinder
NC_019892	474123	487675	36	189	0.99	0.36	35.90	0.33	11.00	0.33	-8.10	99.49	0.00	12.20	15.90	CRISPRCasFinder
NC_019892	5278274	5282042	37	52	0.96	0.30	36.18	0.33	10.00	0.36	-16.70	98.84	0.00	15.90	15.90	CRISPRCasFinder
NC_019892	5318064	5320663	37	36	0.99	0.35	36.20	0.33	6.00	0.35	-14.70	99.67	0.00	15.90	15.90	CRISPRCasFinder
NC_019892	5320758	5322402	37	23	1.00	0.35	36.09	0.33	0.00	0.38	-14.70	71.86	0.00	15.90	15.90	CRISPRCasFinder
NC_019892	5291568	5297044	37	75	0.99	0.35	36.51	0.33	6.00	0.38	-14.70	99.77	0.00	15.90	15.90	CRISPRCasFinder
NZ_CP019327	2757828	2758114	29	5	1.00	0.55	35.50	0.43	0.00	0.31	-2.30	0.00	0.00	43.60	43.60	CRISPRCasFinder
NZ_CP014232	643050	643669	37	9	0.85	0.48	35.88	0.33	8.00	0.37	-8.70	0.00	0.00	15.90	15.90	CRISPRCasFinder
NC_009337	1461368	1461602	32	4	1.00	0.34	35.33	0.33	0.00	0.36	-14.60	0.00	0.00	47.30	52.80	CRISPRCasFinder
NC_019757	3829839	3832320	37	35	1.00	0.65	34.91	0.32	0.00	0.30	-7.80	97.70	0.00	23.30	34.40	CRISPRCasFinder
NC_019757	6781582	6782133	37	8	1.00	0.65	36.57	0.31	0.00	0.38	-7.30	53.84	0.00	23.30	34.40	CRISPRCasFinder
NC_019757	1088841	1090259	37	20	1.00	0.65	35.74	0.32	0.00	0.32	-8.40	50.23	0.00	23.30	34.40	CRISPRCasFinder
NC_019757	6914375	6915668	37	18	0.96	0.48	36.94	0.33	3.00	0.38	-9.20	0.00	0.00	30.70	30.70	CRISPRCasFinder
NC_019757	4707333	4708567	36	17	0.87	0.65	38.94	0.34	17.00	0.38	-8.70	0.00	0.00	15.90	15.90	CRISPRCasFinder
NC_019757	5966534	5968600	37	29	1.00	0.65	35.50	0.33	0.00	0.33	-7.80	0.00	0.00	23.30	34.40	CRISPRCasFinder
NC_019757	4718802	4719490	36	10	0.95	0.64	36.56	0.30	2.00	0.38	-8.70	0.00	0.00	15.90	15.90	CRISPRCasFinder
NC_019757	1101502	1103134	37	23	0.99	0.59	35.55	0.31	1.00	0.33	-7.80	0.00	0.00	23.30	28.80	CRISPRCasFinder
NC_019771	1761752	1763483	37	24	1.00	0.68	36.70	0.32	2.00	0.39	-7.40	0.00	0.00	23.30	34.40	CRISPRCasFinder
NC_019771	5001036	5003639	37	36	0.99	0.70	36.34	0.32	2.00	0.37	-9.00	0.00	0.00	23.30	34.40	CRISPRCasFinder
NC_019771	3250545	3251389	37	12	1.00	0.38	36.45	0.33	0.00	0.37	-14.40	0.00	0.00	19.60	19.60	CRISPRCasFinder
NC_019771	5832140	5832391	36	4	1.00	0.47	36.00	0.37	0.00	0.36	-12.10	0.00	0.00	19.60	23.30	CRISPRCasFinder
NC_019771	5738603	5740678	37	29	1.00	0.68	35.82	0.32	0.00	0.35	-6.70	0.00	0.00	23.30	34.40	CRISPRCasFinder
NC_019771	4752371	4758593	37	84	0.99	0.57	37.53	0.32	4.00	0.36	-11.20	0.00	0.00	28.80	28.80	CRISPRCasFinder
NC_019771	57665	59539	37	26	0.99	0.70	36.52	0.31	2.00	0.38	-9.80	0.00	0.00	23.30	34.40	CRISPRCasFinder
NC_013854	3035919	3038413	37	34	1.00	0.46	37.48	0.33	0.00	0.31	-9.80	0.00	0.00	15.90	15.90	CRISPRCasFinder

NC_013854	1178059	1178606	37	8	0.84	0.42	36.00	0.30	11.00	0.33	-12.10	0.00	0.00	15.90	17.70	CRISPRCasFinder
NC_013854	3158606	3163849	37	71	0.99	0.35	37.39	0.33	4.00	0.35	-17.30	0.00	0.00	15.90	15.90	CRISPRCasFinder
NC_009715	878204	878430	30	4	0.93	0.75	35.67	0.33	4.00	0.36	-1.40	0.00	0.00	15.90	19.60	CRISPRCasFinder
NC_009715	870982	871542	30	9	0.97	0.70	36.38	0.30	1.00	0.37	-1.50	0.00	0.00	15.90	19.60	CRISPRCasFinder
NC_018018	1678085	1679372	37	18	0.97	0.68	36.59	0.33	6.00	0.38	-2.40	0.00	0.00	32.50	32.50	CRISPRCasFinder
NC_018018	11266	12758	37	21	1.00	0.68	35.80	0.34	1.00	0.36	-2.40	0.00	0.00	32.50	32.50	CRISPRCasFinder
NC_018018	205432	206414	37	14	0.98	0.68	35.77	0.33	1.00	0.37	-3.90	0.00	0.00	27.00	27.00	CRISPRCasFinder
NC_018664	704762	706990	30	34	0.95	0.78	36.64	0.34	14.00	0.39	-0.30	83.37	0.00	19.60	19.60	CRISPRCasFinder
NC_018664	2342944	2343634	30	11	0.93	0.78	36.10	0.32	5.00	0.39	0.00	0.00	0.00	17.70	17.70	CRISPRCasFinder
NC_008698	1281906	1282413	24	9	1.00	0.63	36.50	0.32	0.00	0.39	-3.40	0.00	0.00	17.70	17.70	CRISPRCasFinder
NC_008698	1253733	1254901	24	20	0.99	0.63	36.26	0.31	2.00	0.38	-3.40	0.00	0.00	17.70	17.70	CRISPRCasFinder
NC_008698	1260196	1260642	24	8	0.93	0.62	36.43	0.34	2.00	0.38	-3.40	0.00	0.00	17.70	17.70	CRISPRCasFinder
NC_008698	1227253	1228098	24	14	1.00	0.63	39.23	0.33	0.00	0.34	-3.40	0.00	0.00	17.70	17.70	CRISPRCasFinder
NC_008698	1269389	1270964	24	27	1.00	0.63	35.69	0.30	0.00	0.37	0.00	0.00	0.00	17.70	17.70	CRISPRCasFinder
NC_013407	1708464	1709448	30	15	0.92	0.60	38.21	0.32	6.00	0.36	-3.70	91.30	0.00	21.40	21.40	CRISPRCasFinder
NC_013407	886913	888224	30	20	0.97	0.61	37.47	0.35	5.00	0.39	-3.70	0.00	0.00	21.40	21.40	CRISPRCasFinder
NC_013407	293401	294444	30	16	0.90	0.61	37.60	0.34	7.00	0.38	-3.70	73.31	0.00	21.40	21.40	CRISPRCasFinder
NC_013407	763499	764628	30	17	0.96	0.60	38.75	0.36	4.00	0.31	-3.70	61.29	0.00	21.40	21.40	CRISPRCasFinder
NC_013407	443197	444230	30	16	0.90	0.60	36.93	0.37	11.00	0.36	-3.70	0.00	0.00	21.40	21.40	CRISPRCasFinder
NC_013407	226214	226449	31	4	0.90	0.62	37.33	0.32	4.00	0.36	-3.70	0.00	0.00	21.40	21.40	CRISPRCasFinder
NC_013407	1092182	1092483	30	5	1.00	0.63	38.00	0.39	0.00	0.39	-3.70	0.00	0.00	23.30	23.30	CRISPRCasFinder
NC_013407	590203	590633	30	7	0.95	0.61	36.83	0.27	3.00	0.39	-3.70	0.00	0.00	21.40	21.40	CRISPRCasFinder
NC_013407	1134676	1135852	30	18	1.00	0.60	37.47	0.37	0.00	0.38	-3.70	92.15	0.00	21.40	21.40	CRISPRCasFinder
NC_013407	3	568	30	9	0.92	0.59	37.00	0.39	3.00	0.37	-3.70	0.00	0.00	21.40	21.40	CRISPRCasFinder
NC_013407	450018	451283	30	19	0.93	0.58	38.67	0.34	12.00	0.48	-3.70	0.00	0.00	21.40	21.40	CRISPRCasFinder
NC_013407	743131	744904	30	27	0.89	0.61	37.08	0.35	31.00	0.39	-3.70	99.94	0.00	21.40	21.40	CRISPRCasFinder
NC_013407	1714105	1714761	30	10	0.91	0.62	39.67	0.34	8.00	0.36	-3.70	0.00	0.00	21.40	21.40	CRISPRCasFinder
NC_013407	299658	300282	30	10	0.87	0.64	36.11	0.34	9.00	0.54	-3.80	0.00	0.00	32.50	32.50	CRISPRCasFinder
NC_013407	934670	935163	31	8	0.86	0.65	35.14	0.36	11.00	0.38	-3.70	0.00	0.00	23.30	23.30	CRISPRCasFinder
NC_010175	3962639	3975922	37	181	1.00	0.54	36.59	0.31	0.00	0.39	-6.40	77.18	0.00	15.90	15.90	CRISPRCasFinder
NC_010175	4533814	4540010	37	84	0.96	0.46	37.22	0.33	34.00	0.34	-9.60	99.91	0.00	17.70	19.60	CRISPRCasFinder
NC_006391	20071	23194	30	48	0.88	0.42	35.83	0.32	27.00	0.34	-5.00	0.00	0.00	51.00	51.00	CRISPRCasFinder
NC_009633	2274931	2278995	30	62	0.99	0.73	36.15	0.33	4.00	0.38	-0.80	0.00	0.00	21.40	21.40	CRISPRCasFinder
NC_015943	135894	136874	30	15	0.84	0.43	37.93	0.32	18.00	0.37	-5.10	0.00	0.00	51.00	51.00	CRISPRCasFinder
NC_013966	204975	207584	30	40	1.00	0.50	36.15	0.33	1.00	0.36	-7.10	0.00	0.00	56.50	56.50	CRISPRCasFinder
NC_013410	1902105	1902768	37	9	0.84	0.53	41.25	0.31	22.00	0.50	-7.20	0.00	0.00	15.90	15.90	CRISPRCasFinder
NC_013410	1903817	1904406	37	8	0.84	0.53	41.86	0.36	19.00	0.53	-7.20	0.00	0.00	15.90	15.90	CRISPRCasFinder
NC_021658	9167757	9170662	36	40	0.98	0.31	37.59	0.35	6.00	0.44	-15.10	95.80	0.00	15.90	15.90	CRISPRCasFinder
NC_021658	610407	614540	36	57	0.99	0.31	37.18	0.37	4.00	0.38	-16.10	99.90	0.00	15.90	15.90	CRISPRCasFinder
NZ_CP015435	2193741	2195969	30	34	0.97	0.67	36.64	0.32	6.00	0.38	-1.50	0.00	0.00	45.40	51.00	CRISPRCasFinder
NZ_CP015435	257562	258193	30	10	1.00	0.70	36.89	0.31	0.00	0.36	-4.50	0.00	0.00	39.90	45.40	CRISPRCasFinder
NZ_CP015435	275281	275981	30	11	0.97	0.70	37.10	0.31	1.00	0.34	-4.50	0.00	0.00	39.90	45.40	CRISPRCasFinder
NZ_CP015435	1109847	1110145	30	5	1.00	0.63	37.25	0.26	0.00	0.31	-0.50	0.00	0.00	41.70	47.30	CRISPRCasFinder
NZ_CP015435	2325542	2325904	30	6	1.00	0.73	36.60	0.34	0.00	0.38	0.00	94.82	0.00	45.40	51.00	CRISPRCasFinder
NZ_CP015435	235572	236134	30	9	0.93	0.71	36.63	0.33	4.00	0.37	-4.50	0.00	16.40	39.90	45.40	CRISPRCasFinder
NZ_CP015435	296036	296599	30	9	0.85	0.67	36.75	0.33	9.00	0.37	-3.40	0.00	0.00	28.80	45.40	CRISPRCasFinder
NZ_CP015435	212163	213591	30	22	1.00	0.73	36.62	0.32	0.00	0.37	-1.90	0.00	0.00	45.40	51.00	CRISPRCasFinder

NZ_CP015435	308388	310205	30	28	0.97	0.74	36.22	0.33	6.00	0.36	-0.60	0.00	0.00	34.40	51.00	CRISPRCasFinder
NZ_CP015435	2182939	2184772	30	28	0.95	0.67	36.81	0.32	5.00	0.38	0.00	0.00	0.00	34.40	51.00	CRISPRCasFinder
NC_014804	1334169	1334873	30	11	0.97	0.66	37.50	0.30	1.00	0.34	-0.20	0.00	0.00	39.90	56.50	CRISPRCasFinder
NC_009674	2135668	2136221	29	9	0.93	0.66	36.63	0.30	7.00	0.39	0.00	0.00	0.00	21.40	21.40	CRISPRCasFinder
NC_009674	2126031	2126385	29	6	0.92	0.66	36.20	0.32	2.00	0.38	0.00	0.00	0.00	21.40	21.40	CRISPRCasFinder
NZ_CP017006	67	5416	24	90	1.00	0.67	35.84	0.31	0.00	0.30	0.00	0.00	0.00	15.90	15.90	CRISPRCasFinder
NZ_CP017006	2666936	2674015	24	119	1.00	0.67	35.80	0.31	2.00	0.34	0.00	0.00	0.00	15.90	15.90	CRISPRCasFinder
NZ_CP009515	2720506	2722227	37	24	0.98	0.60	36.22	0.32	3.00	0.37	-2.60	91.36	0.00	38.10	38.10	CRISPRCasFinder
NZ_CP009515	3273503	3275585	37	29	1.00	0.62	36.07	0.31	0.00	0.36	-3.30	89.96	0.00	25.10	28.80	CRISPRCasFinder
NZ_CP009515	2704979	2710429	37	74	0.98	0.62	37.16	0.33	11.00	0.38	-2.00	82.20	0.00	41.70	41.70	CRISPRCasFinder
NZ_CP009515	2521336	2523208	37	26	1.00	0.59	36.44	0.32	0.00	0.38	-2.60	84.55	0.00	38.10	38.10	CRISPRCasFinder
NZ_CP009515	2985348	2988430	37	42	0.81	0.61	37.29	0.32	182.00	0.42	-3.10	0.00	0.00	38.10	38.10	CRISPRCasFinder
NZ_CP009515	2722475	2723822	37	19	1.00	0.59	35.83	0.33	0.00	0.36	-2.60	0.00	0.00	38.10	38.10	CRISPRCasFinder
NZ_CP009515	2718618	2719925	37	18	0.96	0.62	37.71	0.34	12.00	0.39	-2.00	0.00	0.00	41.70	41.70	CRISPRCasFinder
NZ_CP009515	2980927	2983740	37	39	1.00	0.60	36.05	0.31	1.00	0.37	-3.10	0.00	0.00	38.10	38.10	CRISPRCasFinder
NZ_CP009515	2984293	2984767	37	7	0.98	0.59	35.83	0.28	2.00	0.36	-3.10	0.00	0.00	38.10	38.10	CRISPRCasFinder
NC_015562	7992	8691	37	10	0.89	0.66	36.67	0.36	12.00	0.38	-8.70	0.00	0.00	32.50	32.50	CRISPRCasFinder
NC_015562	952222	953000	37	11	0.88	0.67	37.20	0.34	11.00	0.39	-5.90	90.80	0.00	32.50	32.50	CRISPRCasFinder
NC_015562	1683471	1683952	37	7	0.80	0.66	37.17	0.30	12.00	0.36	-6.00	0.00	0.00	32.50	32.50	CRISPRCasFinder
NC_015562	295799	296134	37	5	0.86	0.67	37.75	0.32	4.00	0.31	-6.70	0.00	0.00	32.50	32.50	CRISPRCasFinder
NC_015562	717057	718059	37	14	0.95	0.66	37.31	0.30	4.00	0.39	-6.00	0.00	0.00	32.50	32.50	CRISPRCasFinder
NC_015562	1182094	1182647	37	8	0.84	0.67	36.86	0.31	22.00	0.38	-5.90	0.00	0.00	32.50	32.50	CRISPRCasFinder
NC_015562	1280412	1280888	37	7	0.96	0.67	36.33	0.32	2.00	0.39	-6.00	0.00	0.00	32.50	32.50	CRISPRCasFinder
NC_015562	1515497	1515804	32	5	0.93	0.59	37.00	0.33	2.00	0.39	-3.90	0.00	0.00	27.00	28.80	CRISPRCasFinder
NC_015562	1491593	1492727	31	17	0.96	0.55	38.00	0.35	3.00	0.37	-3.90	0.00	0.00	25.10	27.00	CRISPRCasFinder
NC_015562	592560	593764	31	17	0.94	0.58	42.38	0.36	8.00	0.58	-7.80	0.00	0.00	27.00	28.80	CRISPRCasFinder
NC_015562	1346391	1346824	31	7	0.96	0.59	36.17	0.38	5.00	0.36	-7.80	0.00	0.00	27.00	28.80	CRISPRCasFinder
NC_013156	1343405	1343703	30	5	1.00	0.60	37.25	0.30	0.00	0.31	-4.20	0.00	0.00	43.60	43.60	CRISPRCasFinder
NC_013156	760023	760581	30	9	0.96	0.66	36.13	0.34	4.00	0.37	-3.10	0.00	0.00	27.00	27.00	CRISPRCasFinder
NC_013156	531678	532047	31	6	0.95	0.63	36.80	0.31	4.00	0.38	-3.00	0.00	0.00	54.70	54.70	CRISPRCasFinder
NC_013156	717618	718580	30	15	0.91	0.61	36.64	0.35	11.00	0.39	-4.20	0.00	0.00	32.50	32.50	CRISPRCasFinder
NC_013156	281438	282884	30	22	1.00	0.57	37.48	0.32	0.00	0.33	-3.00	0.00	0.00	36.20	36.20	CRISPRCasFinder
NC_013156	347389	347890	30	8	0.89	0.58	37.43	0.34	7.00	0.38	-4.20	0.00	0.00	30.70	30.70	CRISPRCasFinder
NC_013156	675146	676254	30	17	0.98	0.60	37.44	0.32	1.00	0.35	-4.20	99.09	0.00	32.50	32.50	CRISPRCasFinder
NC_007514	2511697	2513118	30	22	1.00	0.70	36.29	0.32	0.00	0.36	-2.30	69.87	0.00	19.60	25.10	CRISPRCasFinder
NC_017079	1976690	2011841	37	482	1.00	0.54	36.00	0.32	2.00	0.33	-3.40	99.93	0.00	14.00	0.00	CRISPRCasFinder
NC_017079	2013095	2018981	37	81	0.99	0.54	36.13	0.32	5.00	0.35	-3.40	99.16	0.00	14.00	0.00	CRISPRCasFinder
NC_003901	679124	682529	37	47	0.94	0.62	36.24	0.31	20.00	0.36	-2.60	67.65	0.00	38.10	38.10	CRISPRCasFinder
NC_003901	4089310	4095187	37	81	0.98	0.62	36.01	0.31	9.00	0.33	-2.60	80.00	0.00	38.10	38.10	CRISPRCasFinder
NC_000917	398369	401590	30	48	0.99	0.63	37.91	0.32	1.00	0.32	-2.50	53.90	0.00	25.10	56.50	CRISPRCasFinder
NC_018178	929685	930781	30	17	0.98	0.73	36.69	0.30	4.00	0.38	-1.10	0.00	0.00	21.40	27.00	CRISPRCasFinder
NC_018178	2661872	2663291	30	22	1.00	0.73	36.19	0.31	0.00	0.36	-2.50	0.00	0.00	21.40	23.30	CRISPRCasFinder
NC_017941	1259098	1260440	30	21	1.00	0.50	35.65	0.33	0.00	0.31	-6.80	0.00	0.00	39.90	39.90	CRISPRCasFinder
NC_017941	2102185	2103823	30	25	1.00	0.50	37.04	0.32	0.00	0.43	-6.80	0.00	0.00	39.90	39.90	CRISPRCasFinder
NC_017941	1229454	1230536	30	17	1.00	0.50	35.81	0.31	0.00	0.35	-6.80	0.00	0.00	39.90	39.90	CRISPRCasFinder
NC_019429	39917	40243	37	5	0.86	0.62	35.50	0.28	9.00	0.31	-7.20	0.00	0.00	23.30	34.40	CRISPRCasFinder
NC_019429	14346	14672	37	5	0.86	0.62	35.50	0.28	9.00	0.31	-6.10	0.00	0.00	23.30	34.40	CRISPRCasFinder

NC_018224	842751	845139	30	37	1.00	0.40	35.53	0.32	1.00	0.31	-9.00	89.10	0.00	39.90	39.90	CRISPRCasFinder
NC_018224	2861125	2862408	30	20	0.96	0.43	36.00	0.34	4.00	0.32	-9.50	0.00	0.00	51.00	51.00	CRISPRCasFinder
NC_013385	925686	927040	30	21	1.00	0.57	36.20	0.31	0.00	0.31	-3.20	0.00	0.00	34.40	56.50	CRISPRCasFinder
NC_013385	1775088	1775565	37	7	1.00	0.46	36.50	0.31	0.00	0.39	-9.30	0.00	0.00	23.30	23.30	CRISPRCasFinder
NC_013385	991878	993835	30	30	1.00	0.57	36.48	0.33	0.00	0.38	-3.20	0.00	0.00	34.40	56.50	CRISPRCasFinder
NC_013385	694576	695935	30	21	0.95	0.56	36.50	0.32	8.00	0.38	-3.20	0.00	0.00	34.40	56.50	CRISPRCasFinder
NC_013385	928543	929635	30	17	1.00	0.57	36.44	0.32	0.00	0.38	-3.20	0.00	0.00	34.40	56.50	CRISPRCasFinder
NC_013385	1042896	1043922	30	16	0.99	0.54	36.47	0.34	1.00	0.39	-4.40	0.00	0.00	21.40	43.60	CRISPRCasFinder
NC_013385	927177	928404	30	19	1.00	0.57	36.50	0.32	0.00	0.38	-3.20	0.00	0.00	34.40	56.50	CRISPRCasFinder
NC_013385	818981	820731	30	27	0.98	0.53	36.19	0.32	2.00	0.30	-8.20	0.00	0.00	28.80	51.00	CRISPRCasFinder
NC_009635	1121706	1123032	37	19	0.99	0.65	34.67	0.37	1.00	0.33	-8.10	0.00	0.00	32.50	32.50	CRISPRCasFinder
NZ_CP015438	714007	715568	30	24	0.98	0.63	36.61	0.31	2.00	0.39	-0.60	77.75	0.00	51.00	56.50	CRISPRCasFinder
NZ_CP014265	463459	465605	30	33	0.98	0.77	36.13	0.34	2.00	0.36	-0.50	61.15	0.00	28.80	49.10	CRISPRCasFinder
NZ_CP014265	467425	477912	30	159	1.00	0.77	36.19	0.34	1.00	0.36	-0.50	89.96	0.00	28.80	49.10	CRISPRCasFinder
NZ_CP014265	465705	466726	30	16	1.00	0.77	36.07	0.33	0.00	0.32	-0.50	0.00	0.00	28.80	49.10	CRISPRCasFinder
NZ_CP014265	466896	467324	30	7	1.00	0.77	36.33	0.34	0.00	0.36	-0.50	0.00	0.00	28.80	49.10	CRISPRCasFinder
NC_015416	2749613	2750460	37	12	1.00	0.59	36.73	0.32	0.00	0.38	-2.70	79.20	0.00	34.40	51.00	CRISPRCasFinder
NC_015416	2747357	2747982	37	9	1.00	0.59	36.63	0.32	0.00	0.37	-2.70	0.00	0.00	34.40	51.00	CRISPRCasFinder
NC_015416	2856874	2860348	37	47	1.00	0.57	37.74	0.32	0.00	0.34	-5.20	81.84	0.00	21.40	23.30	CRISPRCasFinder
NC_007355	1660242	1661621	37	19	0.95	0.62	37.61	0.33	10.00	0.37	-3.10	0.00	0.00	38.10	38.10	CRISPRCasFinder
NC_007355	356467	359809	30	51	0.98	0.60	36.26	0.33	3.00	0.36	-4.20	0.00	0.00	19.60	27.00	CRISPRCasFinder
NC_007355	4007239	4009012	36	25	0.90	0.56	36.42	0.31	19.00	0.39	-7.00	57.86	0.00	17.70	19.60	CRISPRCasFinder
NC_019753	669542	671689	36	29	0.99	0.42	39.43	0.34	2.00	0.41	-11.30	0.00	0.00	27.00	27.00	CRISPRCasFinder
NC_013887	1021672	1022671	30	15	0.95	0.63	39.29	0.35	3.00	0.33	-3.10	0.00	0.00	36.20	36.20	CRISPRCasFinder
NC_013887	321361	322389	30	16	0.85	0.63	36.60	0.31	13.00	0.38	-3.10	0.00	0.00	36.20	36.20	CRISPRCasFinder
NC_013887	149917	150764	31	13	0.95	0.59	37.08	0.32	6.00	0.38	-3.80	0.00	0.00	25.10	27.00	CRISPRCasFinder
NC_013887	578759	579482	30	11	0.88	0.63	39.40	0.35	5.00	0.34	-2.50	0.00	0.00	36.20	36.20	CRISPRCasFinder
NC_013887	1693281	1694227	31	14	0.92	0.62	39.46	0.33	8.00	0.30	-3.50	0.00	0.00	19.60	25.10	CRISPRCasFinder
NC_013887	1150801	1152005	30	18	0.92	0.62	39.12	0.34	7.00	0.30	-2.50	56.45	0.00	36.20	36.20	CRISPRCasFinder
NC_013887	1507562	1508829	30	19	1.00	0.63	38.78	0.35	0.00	0.36	-3.10	0.00	0.00	36.20	36.20	CRISPRCasFinder
NC_013887	1004051	1004623	30	9	0.87	0.63	37.88	0.36	6.00	0.31	-3.10	0.00	0.00	36.20	36.20	CRISPRCasFinder
NC_007181	1650669	1658594	24	133	1.00	0.71	35.86	0.32	1.00	0.35	0.00	0.00	0.00	15.90	15.90	CRISPRCasFinder
NC_007181	1786307	1786584	25	5	0.81	0.62	38.25	0.34	6.00	0.31	-7.60	0.00	0.00	28.80	47.30	CRISPRCasFinder
NC_007181	1670962	1675586	24	78	0.98	0.71	35.75	0.32	5.00	0.32	0.00	0.00	0.00	15.90	15.90	CRISPRCasFinder
NC_003228	2998004	2998546	29	9	0.97	0.75	35.25	0.31	4.00	0.31	0.00	0.00	0.00	23.30	30.70	CRISPRCasFinder
NC_011831	688004	688483	37	7	0.93	0.53	36.67	0.36	7.00	0.39	-10.00	0.00	0.00	17.70	19.60	CRISPRCasFinder
NC_011831	689334	700642	37	154	1.00	0.54	36.67	0.31	4.00	0.38	-8.10	97.88	0.00	15.90	19.60	CRISPRCasFinder
NC_011567	804177	809818	29	86	1.00	0.62	37.04	0.31	0.00	0.38	-1.30	58.49	0.00	27.00	27.00	CRISPRCasFinder
NC_011567	812915	816909	29	61	0.99	0.62	37.10	0.32	5.00	0.38	-1.30	60.55	0.00	27.00	27.00	CRISPRCasFinder
NZ_CP009501	1021519	1026905	37	74	1.00	0.65	36.29	0.31	4.00	0.38	-3.10	96.62	0.00	38.10	38.10	CRISPRCasFinder
NZ_CP009501	2418131	2420381	37	31	1.00	0.67	36.80	0.32	1.00	0.38	-3.10	99.31	0.00	38.10	38.10	CRISPRCasFinder
NZ_CP009501	3029861	3033895	37	55	1.00	0.65	37.04	0.31	0.00	0.43	-3.10	0.00	0.00	38.10	38.10	CRISPRCasFinder
NC_017459	1403321	1404737	30	22	0.96	0.57	36.05	0.31	6.00	0.36	-6.80	0.00	0.00	38.10	38.10	CRISPRCasFinder
NC_017459	403466	406637	37	44	0.98	0.54	35.91	0.32	9.00	0.35	-4.30	0.00	0.00	19.60	27.00	CRISPRCasFinder
NZ_CP016077	4266176	4267245	37	15	0.86	0.26	36.79	0.37	19.00	0.36	-19.20	0.00	0.00	15.90	15.90	CRISPRCasFinder
NZ_CP016077	4231183	4231955	37	11	0.93	0.30	36.60	0.36	10.00	0.38	-17.20	58.20	0.00	14.00	15.90	CRISPRCasFinder
NZ_CP016077	3133043	3134042	37	14	0.89	0.27	37.08	0.35	9.00	0.34	-22.60	0.00	0.00	15.90	15.90	CRISPRCasFinder

NZ_CP016077	4236994	4237252	37	4	1.00	0.27	37.00	0.39	0.00	0.36	-19.20	0.00	0.00	15.90	15.90	CRISPRCasFinder
NZ_CP016077	3122733	3123582	37	12	0.98	0.27	36.91	0.35	1.00	0.37	-22.60	0.00	0.00	15.90	15.90	CRISPRCasFinder
NZ_CP009520	284092	288006	37	54	1.00	0.62	36.17	0.32	0.00	0.33	-3.10	0.00	0.00	38.10	38.10	CRISPRCasFinder
NZ_CP009520	3545472	3546205	37	10	1.00	0.49	40.44	0.33	0.00	0.38	-4.80	0.00	0.00	14.00	0.00	CRISPRCasFinder
NZ_CP009520	737419	742144	37	65	1.00	0.62	36.27	0.32	1.00	0.38	-2.60	0.00	0.00	38.10	38.10	CRISPRCasFinder
NC_015388	1934754	1935816	36	15	1.00	0.56	37.36	0.32	0.00	0.33	-2.50	0.00	0.00	14.00	17.70	CRISPRCasFinder
NC_015388	87673	94203	37	90	0.98	0.62	35.97	0.32	7.00	0.30	-3.00	51.40	0.00	39.90	39.90	CRISPRCasFinder
NC_019779	2090436	2094051	37	50	0.98	0.62	36.04	0.32	9.00	0.37	-9.50	0.00	0.00	19.60	19.60	CRISPRCasFinder
NC_019779	1442117	1459695	36	237	1.00	0.67	38.33	0.32	0.00	0.31	-5.40	62.59	0.00	30.70	30.70	CRISPRCasFinder
NC_019779	769221	777947	37	120	1.00	0.62	36.03	0.32	0.00	0.35	-10.20	0.00	0.00	19.60	25.10	CRISPRCasFinder
NC_019779	751854	754744	37	40	1.00	0.62	36.18	0.31	0.00	0.36	-9.80	50.65	0.00	19.60	25.10	CRISPRCasFinder
NC_019977	1149272	1149838	37	8	0.97	0.63	38.71	0.35	4.00	0.38	-3.10	0.00	0.00	32.50	32.50	CRISPRCasFinder
NC_019977	1144393	1145732	37	18	0.94	0.63	39.65	0.33	10.00	0.48	-3.40	51.33	0.00	38.10	38.10	CRISPRCasFinder
NC_019892	1423379	1423552	29	4	0.75	0.39	19.00	0.27	7.00	0.00	-6.30	0.00	0.00	14.00	14.00	CRISPRDetect
NC_014098	2714107	2714367	33	4	0.73	0.31	42.67	0.37	5.00	0.00	-17.00	0.00	0.00	15.90	17.70	CRISPRDetect
NC_010516	2308581	2308876	30	5	0.78	0.77	36.25	0.35	6.00	0.31	0.00	0.00	0.00	14.00	14.00	CRISPRDetect
NC_009776	413687	414037	26	6	0.74	0.58	38.80	0.30	7.00	0.28	-3.60	0.00	0.00	25.10	25.10	CRISPRDetect
NC_009776	290618	290905	26	5	0.79	0.56	39.25	0.32	4.00	0.00	-2.20	0.00	0.00	21.40	27.00	CRISPRDetect
NC_013715	1379456	1380072	35	9	0.68	0.55	37.63	0.32	22.00	0.31	-13.20	0.00	0.00	17.70	17.70	CRISPRDetect
NC_013715	1376010	1376696	35	10	0.78	0.44	37.33	0.34	15.00	0.19	-17.10	0.00	0.00	15.90	15.90	CRISPRDetect
NZ_CP009515	2208536	2208781	40	4	0.73	0.62	28.33	0.46	11.00	0.00	-5.80	0.00	0.00	17.70	19.60	CRISPRDetect
NZ_CP014265	948679	948941	28	4	0.80	0.74	50.00	0.54	6.00	0.36	0.00	0.00	0.00	15.90	17.70	CRISPRDetect
NC_015389	496713	496989	28	5	0.78	0.31	34.00	0.30	6.00	0.00	-14.30	0.00	0.00	27.00	27.00	CRISPRDetect
NC_018010	2530887	2531216	41	4	0.72	0.56	55.00	0.59	8.00	0.00	-2.00	0.00	0.00	14.00	0.00	CRISPRDetect
NC_021658	8837478	8837716	24	6	0.75	0.24	19.00	0.45	6.00	0.00	-6.60	0.00	0.00	12.20	0.00	CRISPRDetect
NC_010175	1265779	1266056	27	4	0.69	0.27	56.33	0.52	8.00	0.00	-4.40	0.00	0.00	0.00	15.90	CRISPRDetect
NC_010175	5185324	5185556	43	4	0.74	0.21	20.00	0.52	10.00	0.00	-19.60	0.00	0.00	15.90	15.90	CRISPRDetect
NC_019753	517036	517258	24	4	0.76	0.66	42.00	0.51	3.00	0.00	0.00	0.00	0.00	15.90	15.90	CRISPRDetect
NC_016109	7225411	7225751	45	4	0.72	0.32	53.33	0.68	18.00	0.36	-21.30	0.00	0.00	19.60	19.60	CRISPRDetect
NZ_CP012072	791143	791400	41	4	0.76	0.31	31.00	0.45	22.00	0.36	-8.60	0.00	0.00	14.00	17.70	CRISPRDetect
NC_014734	401568	401848	41	4	0.70	0.59	38.67	0.47	10.00	0.36	-3.50	0.00	0.00	14.00	17.70	CRISPRDetect
NC_013410	1900584	1900868	32	4	0.75	0.55	52.00	0.27	13.00	0.36	-6.80	0.00	0.00	14.00	15.90	CRISPRDetect
NC_015676	1562873	1563256	47	5	0.72	0.50	37.00	0.40	11.00	0.31	-9.50	0.00	0.00	14.00	0.00	CRISPRDetect
NC_017030	1310084	1310360	32	3	0.75	0.25	45.00	0.22	6.00	0.61	-6.90	0.00	0.00	12.20	0.00	CRISPRDetect
NC_017030	7624049	7624244	27	3	0.74	0.22	27.00	0.17	3.00	0.61	-5.10	0.00	0.00	14.00	14.00	CRISPRDetect
NC_017030	4130122	4130323	24	3	0.62	0.24	33.00	0.16	6.00	0.61	-6.30	0.00	0.00	14.00	14.00	CRISPRDetect
NC_017030	6459861	6460159	34	5	0.79	0.28	32.00	0.49	10.00	0.00	-14.10	0.00	0.00	12.20	0.00	CRISPRDetect
NC_017030	2080682	2080928	38	4	0.67	0.32	31.33	0.40	13.00	0.00	-12.90	0.00	0.00	14.00	0.00	CRISPRDetect
NC_021191	2262368	2262628	26	6	0.70	0.19	27.83	0.33	21.00	0.48	-11.20	0.00	14.40	14.00	15.90	CRISPRDetect
NZ_CP014859	31366	31594	24	3	0.80	0.33	40.33	0.33	2.00	0.36	-2.80	0.00	0.00	12.20	0.00	CRISPRDetect
NC_007963	253349	253622	29	5	0.75	0.28	32.00	0.33	6.00	0.00	-14.30	0.00	0.00	28.80	28.80	CRISPRDetect
NC_019693	2507860	2508300	38	7	0.79	0.61	29.00	0.48	10.00	0.00	-1.60	0.00	0.00	15.90	0.00	CRISPRDetect
NC_005966	3159445	3159764	34	5	0.67	0.49	37.25	0.43	19.00	0.77	-4.00	99.56	0.00	12.20	0.00	CRISPRDetect
NZ_CP011509	7157715	7158087	36	5	0.58	0.34	48.00	0.54	23.00	0.00	-12.00	0.00	11.80	14.00	0.00	CRISPRDetect
NZ_CP011266	386068	386626	33	8	0.70	0.66	42.00	0.56	19.00	0.23	-3.30	90.99	0.00	19.60	19.60	CRISPRDetect
NZ_CP018201	1402631	1402898	27	5	0.72	0.28	33.00	0.32	9.00	0.31	-12.50	0.00	0.00	12.20	0.00	CRISPRDetect
NC_015711	2109609	2109816	37	4	0.69	0.37	19.67	0.59	10.00	0.00	-12.20	0.00	0.00	14.00	0.00	CRISPRDetect

NC_015711	376379	376558	29	2	0.52	0.26	17.67	0.08	5.00	0.36	-12.50	0.00	0.00	15.90	15.90	CRISPRDetect
NC_015711	5324594	5324857	38	4	0.72	0.26	37.00	0.59	12.00	0.00	-12.10	0.00	0.00	12.20	0.00	CRISPRDetect
NC_021355	1061427	1061700	39	4	0.68	0.67	39.00	0.47	14.00	0.00	-2.00	0.00	0.00	19.60	19.60	CRISPRDetect
NC_023002	792984	793409	44	5	0.73	0.79	51.25	0.43	17.00	0.31	-1.70	66.32	0.00	17.70	17.70	CRISPRDetect
NZ_CP008953	4939568	4939747	23	4	0.75	0.22	29.00	0.40	4.00	0.36	-3.50	0.00	0.00	0.00	0.00	CRISPRDetect
NC_014472	1860530	1860780	28	4	0.59	0.61	46.00	0.43	11.00	0.00	-3.30	0.00	0.00	14.00	15.90	CRISPRDetect
NC_011026	322056	322383	49	4	0.87	0.57	43.67	0.67	10.00	0.36	-13.80	0.00	0.00	15.90	17.70	CRISPRDetect
NC_016640	1573710	1574056	53	4	0.88	0.47	44.67	0.66	11.00	0.36	-12.20	0.00	0.00	15.90	15.90	CRISPRDetect
NC_016640	1107107	1107339	40	4	1.00	0.40	54.67	0.68	0.00	0.00	-12.50	0.00	0.00	14.00	0.00	CRISPRDetect
NC_016894	3018674	3018876	34	4	0.86	0.46	34.00	0.65	4.00	0.36	-6.10	0.00	5.80	12.20	0.00	CRISPRDetect
NZ_CP014265	1029630	1029841	36	4	0.84	0.85	20.67	0.79	7.00	0.00	-0.20	0.00	0.00	14.00	15.90	CRISPRDetect
NZ_CP009524	2118738	2119023	38	5	0.96	0.65	40.25	0.66	1.00	0.58	0.00	0.00	0.00	17.70	21.40	CRISPRDetect
NC_014734	223917	224140	27	4	1.00	0.59	38.33	0.61	0.00	0.00	-3.40	0.00	0.00	19.60	19.60	CRISPRDetect
NZ_CP011350	391298	391500	26	4	0.88	0.59	30.00	0.63	2.00	0.36	-2.80	0.00	8.90	12.20	15.90	CRISPRDetect
NC_009953	4527637	4528481	26	19	1.00	0.38	34.44	0.74	0.00	0.38	-3.50	0.00	0.00	17.70	17.70	CRISPRDetect
NC_007355	2329061	2329332	55	4	0.83	0.59	54.33	0.70	6.00	0.00	-9.90	0.00	0.00	15.90	15.90	CRISPRDetect
NC_012032	5037792	5037988	34	4	0.91	0.35	37.00	0.73	2.00	0.36	-5.20	0.00	0.00	12.20	0.00	CRISPRDetect
NC_009792	3462671	3462885	28	4	0.96	0.40	34.00	0.64	1.00	0.36	-5.20	0.00	0.00	12.20	14.00	CRISPRDetect
NC_003551	1297217	1297618	36	5	1.00	0.58	55.25	0.32	0.00	0.39	-10.60	0.00	0.00	14.00	0.00	CRISPRDetect
NC_003551	1362696	1363455	36	9	1.00	0.61	54.38	0.37	0.00	0.37	-5.70	0.00	0.00	14.00	15.90	CRISPRDetect
NZ_CP016463	264278	264672	28	5	0.97	0.26	63.50	0.43	1.00	0.39	-14.00	0.00	0.00	14.00	17.70	CRISPRDetect
NC_011831	4279980	4280255	42	4	0.83	0.40	35.67	0.49	9.00	0.36	-7.10	0.00	0.00	14.00	15.90	CRISPRDetect
NZ_CP009149	152943	153181	31	4	1.00	0.61	38.00	0.34	0.00	0.36	-3.00	0.00	0.00	41.70	41.70	CRISPRDetect
NC_016640	5830307	5830622	24	6	0.82	0.53	31.83	0.37	8.00	0.74	-2.60	0.00	0.00	14.00	15.90	CRISPRDetect
NC_016640	2897603	2897928	35	5	0.94	0.71	37.50	0.35	1.00	0.31	-2.00	0.00	0.00	19.60	19.60	CRISPRDetect
NC_016640	6715986	6716198	29	4	0.87	0.48	39.00	0.42	4.00	0.36	-2.20	0.00	0.00	14.00	15.90	CRISPRDetect
NC_016640	5693729	5694044	33	3	0.85	0.47	53.00	0.25	2.00	0.36	-7.50	0.00	0.00	15.90	15.90	CRISPRDetect
NC_009776	89078	89370	26	5	1.00	0.54	40.50	0.30	0.00	0.31	-2.50	0.00	0.00	21.40	27.00	CRISPRDetect
NC_009776	414010	414304	27	5	0.92	0.59	39.75	0.31	2.00	0.31	-3.60	0.00	0.00	19.60	25.10	CRISPRDetect
NC_014222	590036	591511	31	22	1.00	0.71	37.76	0.33	0.00	0.36	0.00	0.00	0.00	28.80	30.70	CRISPRDetect
NZ_CP012159	2248191	2248445	41	3	0.87	0.32	32.67	0.31	3.00	0.36	-8.10	0.00	0.00	14.00	15.90	CRISPRDetect
NC_013407	68	366	31	5	1.00	0.61	35.75	0.36	0.00	0.31	-3.70	0.00	0.00	21.40	21.40	CRISPRDetect
NZ_CP017006	2666994	2668394	24	24	0.98	0.67	35.83	0.33	1.00	0.32	0.00	0.00	0.00	15.90	15.90	CRISPRDetect
NZ_CP017006	2668428	2669899	24	25	1.00	0.67	36.29	0.32	0.00	0.38	0.00	0.00	0.00	15.90	15.90	CRISPRDetect
NZ_CP017006	3105	4565	24	25	1.00	0.67	35.83	0.31	0.00	0.34	0.00	0.00	0.00	15.90	15.90	CRISPRDetect
NC_021658	11093169	11093450	26	5	0.84	0.27	23.80	0.31	5.00	0.53	-4.40	0.00	0.00	15.90	15.90	CRISPRDetect
NC_021658	9397554	9397800	30	3	0.83	0.17	40.00	0.17	5.00	0.61	-12.90	0.00	0.00	14.00	15.90	CRISPRDetect
NC_016001	1901505	1901911	29	6	0.94	0.76	46.40	0.38	1.00	0.53	0.00	0.00	0.00	14.00	15.90	CRISPRDetect
NC_000909	1034820	1035552	31	11	0.97	0.62	39.10	0.35	1.00	0.34	-4.10	0.00	0.00	27.00	28.80	CRISPRDetect
NC_003552	5529616	5529799	27	5	0.85	0.60	23.50	0.37	3.00	0.39	0.00	72.83	0.00	15.90	17.70	CRISPRDetect
NC_007181	1672454	1673914	24	25	0.99	0.71	35.83	0.32	1.00	0.34	0.00	0.00	0.00	15.90	15.90	CRISPRDetect
NC_007181	1656951	1658414	24	25	1.00	0.71	35.96	0.32	0.00	0.31	0.00	0.00	0.00	15.90	15.90	CRISPRDetect
NC_007181	1674481	1674867	25	5	1.00	0.72	65.25	0.39	0.00	0.58	0.00	0.00	0.00	15.90	15.90	CRISPRDetect
NC_003901	2645597	2645812	25	3	0.89	0.76	42.33	0.31	3.00	0.36	0.00	0.00	0.00	17.70	17.70	CRISPRDetect
NZ_CP014232	1225367	1225822	28	5	0.81	0.23	78.75	0.46	4.00	0.31	-13.00	0.00	0.00	19.60	19.60	CRISPRDetect
NC_008698	1271320	1272732	24	24	1.00	0.63	36.35	0.31	0.00	0.43	-3.40	62.85	0.00	17.70	17.70	CRISPRDetect
NC_008698	1227253	1228099	24	14	1.00	0.63	39.23	0.33	0.00	0.34	-3.40	0.00	0.00	17.70	17.70	CRISPRDetect

NC_008698	1253733	1254902	24	20	0.99	0.63	36.26	0.32	2.00	0.38	-3.40	0.00	0.00	17.70	17.70	CRISPRDetect
NC_008698	1272774	1273334	24	10	1.00	0.63	35.56	0.30	0.00	0.36	-3.40	0.00	0.00	17.70	17.70	CRISPRDetect
NC_008698	1269389	1270900	24	26	1.00	0.63	35.48	0.29	0.00	0.36	0.00	0.00	0.00	17.70	17.70	CRISPRDetect
NC_008698	1281906	1282414	24	9	1.00	0.63	36.50	0.32	0.00	0.39	-3.40	0.00	0.00	17.70	17.70	CRISPRDetect
NC_013887	1004119	1004558	31	7	0.95	0.65	37.00	0.39	2.00	0.36	-3.10	0.00	0.00	36.20	36.20	CRISPRDetect
NC_013887	149980	150765	31	12	1.00	0.58	37.55	0.32	0.00	0.37	-3.80	93.64	0.00	25.10	27.00	CRISPRDetect
NC_007963	266255	266528	29	4	0.82	0.27	52.33	0.36	4.00	0.36	-14.30	0.00	0.00	23.30	23.30	CRISPRDetect
NC_019693	6293280	6293511	25	4	1.00	0.56	43.67	0.59	0.00	0.36	0.00	0.00	0.00	12.20	14.00	CRISPRDetect
NC_019693	6666166	6666361	31	3	0.90	0.54	25.33	0.11	1.00	0.36	-1.00	0.00	0.00	14.00	19.60	CRISPRDetect
NC_009953	2940163	2940921	29	10	0.89	0.25	52.00	0.39	5.00	0.36	-13.90	0.00	0.00	21.40	21.40	CRISPRDetect
NC_005966	2339398	2339726	28	5	1.00	0.50	47.00	0.31	0.00	0.31	-10.20	0.00	0.00	39.90	39.90	CRISPRDetect
NZ_CP014688	506741	507075	29	5	1.00	0.31	47.25	0.32	0.00	0.31	-9.30	0.00	0.00	27.00	27.00	CRISPRDetect
NC_015562	1346593	1346823	29	4	0.92	0.56	38.33	0.42	1.00	0.36	-7.80	0.00	0.00	25.10	27.00	CRISPRDetect
NC_015562	592560	593661	31	16	1.00	0.58	40.33	0.36	0.00	0.48	-7.80	0.00	0.00	27.00	28.80	CRISPRDetect
NC_015562	1045115	1045422	31	5	1.00	0.58	38.00	0.30	0.00	0.31	-7.80	0.00	0.00	27.00	28.80	CRISPRDetect
NC_007355	359508	359742	31	4	1.00	0.61	36.67	0.33	0.00	0.36	-4.20	0.00	0.00	19.60	27.00	CRISPRDetect
NC_022116	6451338	6451610	28	4	0.80	0.23	53.33	0.43	3.00	0.36	-13.40	0.00	0.00	15.90	15.90	CRISPRDetect
NC_014408	546685	547056	29	5	0.93	0.57	56.50	0.49	2.00	0.39	-0.80	0.00	0.00	14.00	14.00	CRISPRDetect
NC_015850	1236374	1237000	28	9	0.81	0.26	36.56	0.25	10.00	0.38	-13.10	0.00	0.00	19.60	19.60	CRISPRDetect
NC_021044	1272488	1272792	31	5	0.92	0.70	37.25	0.33	3.00	0.31	-1.90	0.00	0.00	27.00	36.20	CRISPRDetect
NC_014374	323	1051	24	12	1.00	0.58	40.00	0.30	0.00	0.33	-3.20	66.11	0.00	17.70	17.70	CRISPRDetect
NZ_CP011267	1166413	1166868	24	8	1.00	0.54	37.57	0.32	0.00	0.33	-2.90	0.00	0.00	15.90	17.70	CRISPRDetect
NC_015636	1530820	1531123	31	5	0.98	0.67	37.00	0.38	1.00	0.31	-4.40	0.00	0.00	27.00	34.40	CRISPRDetect
NC_012032	5036161	5036460	41	3	0.85	0.37	41.67	0.28	7.00	0.61	-4.80	0.00	0.00	12.20	0.00	CRISPRDetect
NZ_CP007044	73619	73948	28	3	0.89	0.51	63.67	0.17	1.00	0.36	-9.10	0.00	0.00	52.80	52.80	CRISPRDetect
NC_018524	2254142	2254469	29	5	0.95	0.21	45.50	0.33	3.00	0.31	-14.50	0.00	0.00	25.10	25.10	CRISPRDetect
NC_015312	3053029	3053421	25	6	0.84	0.38	48.40	0.50	6.00	0.59	-2.90	0.00	0.00	12.20	0.00	CRISPRDetect
NC_013222	1525087	1525382	25	5	0.88	0.42	42.50	0.49	3.00	0.31	-0.20	0.00	0.00	14.00	14.00	CRISPRDetect
NC_013922	2854510	2854683	41	4	0.87	0.34	25.00	0.55	3.00	0.00	-4.30	0.00	0.00	17.70	17.70	CRISPRDetect
