# Supplementary material for: CRISPRidentify: identification of CRISPR arrays using machine learning approach
Source: Nucleic Acids Res. 2020 Dec 8;49(4):e20. doi: 10.1093/nar/gkaa1158 (PMC7913763; doi:10.1093/nar/gkaa1158)
Supplement: gkaa1158_Supplemental_Files [file gkaa1158_supplemental_files.zip › CRISPRidentify_Suppl-3.pdf]

CRISPRidentify: Identification of CRISPR arrays using machine learning approach – Supplementary Material 3

Alexander Mitrofanov, Omer S Alkhnbashi, Sergey Shmakov,
Kira M. Makarova, Eugene V. Koonin and Rolf Backofen

Here we demonstrate the cases from figure 5 which are unique for CRISPRidentify. Although our approach is able to identify such cases many of them were assigned to the "low score" category.

```
('JXUQ01000003', 132961, 133306)

133034   ................A...-......C  GACCGCCACGCGCGCGGATCGTCTGTGGTGCTGACGTCCTCGATGG
s:2 i:0 d:1

133107   C.....................G...A.  CGACCGCCACCGCCGCCGGAGGGCGACCGCCACCTCGCCCTGGAAGCCGCCTCCTGGTCGTCATACCTGC
s:3 i:0 d:0

133205   ............................  CGACCGCCACTCCGCGAGGTCATGAAGTGGCTCTCCGACCCCAAGG
s:0 i:0 d:0

133279   ......................G.....
s:1 i:0 d:0

______________________________________________________________________________________________

         AGTTGCGATCCTCGGCCGCCCCAGGGGG
s:6 i:0 d:1
```

```
('KE734710', 3575463, 3575672)

3575461   ..........................  NNNNNNNNNNNNNNNNNNNNNCCCCCCTTGCTAAGGGGG      s:0 i:0 d:0

3575525   ..........................  CATTACTAAGGGGGGGGCTAGGGGGGGTAACTTCC         s:0 i:0 d:0

3575586   CC.A......................  TCTTGGTAAGGGGGGGGCTAGGGGGGGTAACTTCC         s:3 i:0 d:0

3575647   CC........................                                              s:2 i:0 d:0

______________________________________________________________________________________________

          GGAGAAGAAAACCAAACATTTCCCCC                                              s:5 i:0 d:0
```

```
('KE734710', 3575463, 3575672)

3575570   .....................  AGAAAACCAAACATTTCCCCCTCTTGGTAAGGGGGGGGCT      s:0 i:0 d:0

3575631   ...................G.  AGAAAACCAAACATTTCCCCCCATTACTAAGGGGGGGTT       s:1 i:0 d:0

3575691   ...........T.........                                                s:1 i:0 d:0

______________________________________________________________________________________________

          AGGGGGGGTAACTTCCCCAAA                                                s:2 i:0 d:0
```

```
('BAZG01000054', 6916, 7094)

6965   ...............C-T...GT-C  CGCTCATGGCGAGCCTCCGGTGTCC               s:4 i:1 d:2

7013   ....A................ ...  TGAGCGGGCGGACGCCCGTGGATGAGCGAGCTCC      s:1 i:0 d:0

7071   ..................... ...                                          s:0 i:0 d:0

______________________________________________________________________________________________

       GCTCGTGGTGAGCCCGTCGAA CCA                                          s:5 i:1 d:2
```

('KQ950182', 250231, 250454)

250289   .A.......-C.C.......... GTCCCGACACTGGGGGAGAGCCCCTCACCTCCCCGGA   s:2 i:1 d:1

250350   ....G.. GTCC   s:1 i:0 d:0

250377   -C.....T...C ....-G.. ATCCGGACAGCCGACAGGGTGTCGGGACAGACCTGC   s:4 i:0 d:2

250434   ....   s:0 i:0 d:0

TGCTCCCCGCTG CTGCGGGGATG   s:7 i:1 d:3

('JYGU01000006', 307798, 308028)

307882   .... ATGAAAAAACTAAATGTTTTTCATATAGTTTTCATTTTTTGT   s:0 i:0 d:0

307956   --.TT.T-A...................... TTAAGGTGCCTT   s:4 i:0 d:3

307997   ....   s:0 i:0 d:0

AAGAAAAATATTAAGGTGCCTTAAGAAAAATA   s:4 i:0 d:3

('JYGU01000006', 307798, 308028)

307793   .... ATGAAAAAACTAAATGTTTTTCATATAGTTTTCATTTTTTGTG
s:0 i:0 d:0

307864   ....C.............. TTAAGGTGCCTTAAGAAAAATAATGAAAAAACTAAATGTTTTTCATATAGTTTTCATTTTTTGTG
s:1 i:0 d:0

307957   .... TTAAGGTGCCTTAAGAA
s:0 i:0 d:0

308002   AA..-......................
s:2 i:0 d:1

TTATAATTAAGGTGCCTTAAGAAAAATA
s:3 i:0 d:1

('CP006805', 366949, 367316)

367134   .... TGGGGCAGGCTGACCAAACCCTTGTTGCGGTTGTTGCCATTGTTGTTGACC
s:0 i:0 d:0

367230   ....G........ TGGAGC
s:1 i:0 d:0

367281   ............T..........C...........T........
s:3 i:0 d:0

AGGTTGACCAAACCCTTGTTGAGGTGCTTGTTGGAACCCTTGTCC
s:4 i:0 d:0

('CP006805', 366949, 367316)

367133 .... AGGTGCTTGTTGGAACCCTTGTCCTGGGG s:0 i:0 d:0

367184 ....C................. CGGTTGTTGCCATTGTTGTTGAC s:1 i:0 d:0

367229 .... AGGTGCTTGTTGGAAGCCTTGTCCTGGAG s:0 i:0 d:0

367280 ....T......... AGGCGCTTGTTGGAATCCTTGTCCTGGGG s:1 i:0 d:0

367331 ....C................. s:1 i:0 d:0

CAGGTTGACCAAACCCTTGTTG s:3 i:0 d:0

('JH791965', 57, 411)

57 .T....... .... ACGTTTACAATCATGCTGTCTTTATCATCTATATCATAGCATTACG s:1 i:0 d:0

126 -...G.... ..A......T.... ATAGTCTTTATCTTCTAATAAGTACCCTATATCATAGCATTGC s:3 i:0 d:1

191 .T....... ....T.... TGAATCTTTATGAGGGAAATCCTAATTAGTATCATAGCATTGC s:2 i:0 d:0

257 A........ .... ATGATGAAATTATATTACAACAAGTATATGAGCATAGCATAGT s:1 i:0 d:0

323 A........ ....T...... TATTCAGGAATCTAACGTTATCCTTTAATATTCATAGCATAGC s:2 i:0 d:0

389 ......-A.TC.T.......... s:3 i:1 d:1

GATGTTTCT TAGGGAACTATAGC s:12 i:1 d:2

('CP003418', 1389736, 1390437)

1390201 .... CTTTGCTGTTTTGCCTGCTTTATGAGCTTTGTCCTCGTTTTAATCGAACCACTT
s:0 i:0 d:0

1390297 .... CCCAACAAGAGCTGAGATTGTCCCCCATTAGTATTTGAGTTTTAATCGAACCACCT
s:0 i:0 d:0

1390395 ....
s:0 i:0 d:0

GTCCCTAACGAAACGAAGTGAAGTGGGATAGTGGAATTGAGA
s:0 i:0 d:0

('AL157959', 1555693, 1556244)

1556299 .... GGGATTTTAGGTTTCTGATTTTGGTT s:0 i:0 d:0

1556348 -...-T.T.....A.-....... GGGATGCAGGTTTTCTTAACCCTGCGTCCTAG s:3 i:0 d:3

1556400 .... s:0 i:0 d:0

ATTCCCGCTTTTGCGGGAATGAC s:3 i:0 d:3

('CP010333', 929319, 930667)

929322 .... CGGCGGCACGTCGGTTGCCACCGGGGGGCCGGGAACGGCGGTGCCGGCGGGGCC
s:0 i:0 d:0

929420 .... CGGCGGCACGTCGGTTGCCACCGGGGGGCCGGGAACGGCGGTGCCGGCGGCGCC
s:0 i:0 d:0

929518 ....C.....
s:1 i:0 d:0

____

GGCGGCGGGGCCGGGCTGATCGGCAACGGCGGCAACGGTGGCAG
s:1 i:0 d:0

('CP010333', 929319, 930667)

929322 .... C s:0
i:0 d:0

929367 ...... ACGTCGGTTGCCACCGGGGGGCCGGGAACGGCGGTGCCGGCGGGGCC s:0
i:0 d:38

929420 .... CGGCGGCACGTCGGTTGCCACCGGGGGGCCGGGAACGGCGGTGCC s:0
i:0 d:0

929509 .... CC s:0
i:0 d:37

____

GGCGGCGGGGCCGGGCTGATCGGCAACGGCGGCAACGGTGGCAG s:0
i:0 d:75

('KK357435', 924667, 925925)

924667 .... CGGCGGCACGTCGGTTGCCACCGCCAACGGCTCTACCGGCGGTGCCGGCGGG
s:0 i:0 d:0

924766 .... CGGCGGCACGTCGGTTGCCACCGGGGGGGCCGGGAACGGCGGTGCCGGCGGC
s:0 i:0 d:0

924865 ....C.....
s:1 i:0 d:0

____

GCCGGCGGCGGGGCCGGGCTGATCGGCAACGGCGGCAACGGTGGCAG
s:1 i:0 d:0

('KK357435', 924667, 925925)

924667 .... CGGCGGCACGTCGGTTGCCACCGCCAACGGCTCTACCGGCGGT s:0
i:0 d:0

924757 .... G s:0
i:0 d:39

924766 .... CGGCGGCACGTCGGTTGCCACCGGGGGGGCCGGGAACGGCGGT s:0
i:0 d:0

924856 .... s:0
i:0 d:38

____

GCCGGCGGCGGGGCCGGGCTGATCGGCAACGGCGGCAACGGTGGCAG s:0 i:0 d:77

('ALWD01000163', 11389, 11601)

11285 ............--C...TG. CTTA s:3 i:0 d:2

11309 .... ATGTATCTGGTACTTACGGGAACAAAGGTACAAAATAGGCTAAACTTACGGAGAGAACTC s:0 i:0 d:0

11391 ....G TTTTACAAGCCTTACCGCGTAAGTCATCAAAAAGTCAACCCGAAAAAAATG s:1 i:0 d:0

11464 .... ATG s:0 i:0 d:0

11489 .... CAAGGTGATGTGTGCTGGGCTTACGGAGAAAACTC s:0 i:0 d:0

11546 .... GACTCAAAATCA s:0 i:0 d:0

11580 ....G.TT s:3 i:0 d:0

TACGCCGGACTCACGGATAAAA s:7 i:0 d:2

('ALWD01000163', 11389, 11601)

11304 .C-T . ... AATGTATCTGGTACTTACGGGAACAAAGGTACAAAATAGGCTAAACTTACGGAG s:2 i:0 d:1

11384 .G..C. C.................... GTTTTACAAGCCTTACCGCGTAAGTCATCAAAAAGTCAACCCGAAA s:2 i:1 d:0

11458 .... . G.................... s:1 i:0 d:0

11485 .--. . G.................... ACAAGGTGATGTGTGCTGGGCTTACGGAG s:1 i:0 d:2

11539 ....C. C.................... AGACTC s:1 i:1 d:0

11573 .... .C..................G.T s:2 i:1 d:0

AAAA T ATACGCCGGACTCACGGATAAA s:9 i:3 d:3

('LDVW01000012', 28533, 28759)

28532 .... ACACAACTTCGATGTAAACATTTCCCGGCTGTAAC s:0 i:0 d:0

28592 ....C......... GCACGTGTGCTGTGCTTTCACGTCACGAAGCTCGG s:1 i:0 d:0

28652 ....G....... GCACAATCCCCGGTAGGCAACGTTTGATGGCTCTT s:1 i:0 d:0

28712 ................-T--G.T. s:3 i:0 d:3

28734 ---...................... s:0 i:0 d:3

TTTTCTAAGCTGCCTGTACGGCAGT s:5 i:0 d:6

('ALES01000047', 469892, 470103)

469892 .... AGGATTTCGCCGCGCTGTTGGCCTCCAGATTC s:0 i:0 d:0

469953 .... CCGTGCCTGTCCAGGACAAATTGCCGATTATT s:0 i:0 d:0

470014 .... CCGTGCCTGTCCAGGACAAATTGCCGATTATT s:0 i:0 d:0

470075 .... s:0 i:0 d:0

GTGTTCCCCGCGCCAGCGGGGATAAACTG s:0 i:0 d:0

('ALES01000047', 469892, 470103)

469892 ....AG. ATTTCGCCGCGCTGTTGGCCTCCAGATTC s:2 i:0 d:0

469953 .... TGCCTGTCCAGGACAAATTGCCGATTATT s:0 i:0 d:0

470014 .... TGCCTGTCCAGGACAAATTGCCGATTATT s:0 i:0 d:0

470075 .... s:0 i:0 d:0

GTGTTCCCCGCGCCAGCGGGGATAAACTGCCG s:2 i:0 d:0

('ALES01000047', 469892, 470103)

469956 .... GACAAATTGCCGATTATTGTG s:0 i:0 d:0

470017 .... GACAAATTGCCGATTATTGTG s:0 i:0 d:0

470078 .... s:0 i:0 d:0

TTCCCCGCGCCAGCGGGGATAAACTGCCGTGCCTGTCCAG s:0 i:0 d:0

('KK211044', 110, 631)

113 .... TGTTCTTATCCTTCTATCTACTTCCAAACCATTGTCTAGTT s:0 i:0 d:0

180 .... TAGGTTTGTAATAGCTTGTCTAGGGTTTGAATCTTATT s:0 i:0 d:0

244 ....TT.......G-A........TC.. s:4 i:2 d:1

GAACAATAACATA AGATGTATTT AAA s:4 i:2 d:1

('HE973250', 50730, 50967)

50730 ....G.......... CAGCCGATTGGAACGGCGAAATTATTCAAAAAGGTGATCACTGGGAGATA s:1 i:0 d:0

50803 .... CATTCGATTGCCACATTATAAGGCAAGCACCATATAAAGTCAAGTCTT s:0 i:0 d:0

50874 ....T.................. AAATCGATTGGAACCAGGGCTGAGTGGTTGGTCAATTGGCAATCTTTA s:1 i:0 d:0

50945 .............T.....-... s:1 i:0 d:1

GTTTCAATCCCTAATAGGGAATT s:3 i:0 d:1

```
('JAUE01000064', 28945, 29454)

29358   ......... ............  CTAAATCACTATATGATTTTAAACCAGATGTATTAACTGTAACGCCTGCTTTC     s:0 i:0 d:0

29433   .T....... ............  ACATTGTGTTTGTGATTGTGTTTTGTGTAGTT                          s:1 i:0 d:0

29487   .....T...T.-C.-....A..A                                                           s:4 i:1 d:2

        TGAGCATCT GCTAAAGCTTGCG                                                           s:5 i:1 d:2



('AM889136', 624935, 625457)

625287   -.......A...T.G-T.........  GCGATTAGAGTTTCAAAATTTATTCTAAATAGCTGAAATTCAATGAACC     s:3 i:1 d:2

625360   ..........C... ...........  GAAGTGGAAGTTACCCGAAACTTAAAACAAGCGAAACCGAACGAGCC       s:1 i:0 d:0

625432   .............. ...........                                                        s:0 i:0 d:0

         GGATTCCCGCTTGC GCGGGAATGAC                                                        s:4 i:1 d:2



('AM889136', 624935, 625457)

625287   -.......A...T.G-T........  CGCGATTAGAGTTTCAAAATTTATTCTAAATAGCTGAAATTCAATGAACC     s:3 i:1 d:2

625360   ..........C... ..........  CGAAGTGGAAGTTACCCGAAACTTAAAACAAGCGAAACCGAACGAGCC       s:1 i:0 d:0

625432   .............. ..........                                                         s:0 i:0 d:0

         GGATTCCCGCTTGC GCGGGAATGA                                                         s:4 i:1 d:2



('JH791966', 12, 298)

14     ..G.... ..A......T....  ATAGTCTTTATCTTCTAATAAGTACCCTATATCATAGCATTGCGT     s:3 i:0 d:0

80     ....... .........T....  TGAATCTTTATGAGGGAAATCCTAATTAGTATCATAGCATTGCAA     s:1 i:0 d:0

146    ....... ..............  ATGATGAAATTATATTACAACAAGTATATGAGCATAGCATAGTAA     s:0 i:0 d:0

212    ....... .......T......  TATTCAGGAATCTAACGTTATCCTTTAATATTCATAGCATAGCGA     s:1 i:0 d:0

278    ....-A.TC.T...........                                                     s:3 i:1 d:1

       TGTTTCT TAGGGAACTATAGC                                                     s:8 i:1 d:1



('JH815303', 97556, 97913)

97555   ..A............A...........  TTTCAGGCTGCCTGAATCACTGGGCAGCCTGTTGTCGTCC     s:1 i:1 d:0

97623   .. ........................  CCCAACCTTTGGCTTTTGCTACGTCCAACAGTTGTAGTT      s:0 i:0 d:0

97689   .. ........................  TCACTCACGCGCTCACGTATCTATCACTCAGTTGTAGTT      s:0 i:0 d:0
```

```
97755   .. ........................  TAACACACGCACTCACGTATCTATCACTCAGTTGTAGTT       s:0 i:0 d:0

97821   .. ........................  TATCACGTTCACGCATCTATCACGTTCACGGTTGTAGTT       s:0 i:0 d:0

97887   .. ........................                                                s:0 i:0 d:0
____________________________________________________________________________________________________

        CC CCCTCTCATCTCGTAGTGCTATAAT                                               s:1 i:1 d:0



('JH815303', 97556, 97913)

97546   -....A.C.-.C.A.. ..........A............  TTCAGGCTGCCTGAATCACTGGGCAGC       s:4 i:1 d:2

97611   .T.....C..C ....C......................-.  CCCAACCTTTGGCTTTTGCTACGTCCAA      s:3 i:1 d:1

97678   .......... .... .......................  CACTCACGCGCTCACGTATCTATCACT       s:0 i:0 d:0

97744   .......... .... .......................  AACACACGCACTCACGTATCTATCACT       s:0 i:0 d:0

97810   .......... .... .......................  ATCACGTTCACGCATCTATCACGTTCA       s:0 i:0 d:0

97876   .G........ .... ......................A                                    s:2 i:0 d:0
____________________________________________________________________________________________________

        CAGTTGTAGTT CCCC CTCTCATCTCGTAGTGCTATAATT                                  s:9 i:2 d:3



('CM000720', 1251676, 1251913)

1251667   ..G... ........A.. ......  G
s:1 i:1 d:0

1251691   .. ...G.......... A.....  GA
s:1 i:1 d:0

1251716   .. ... .......G... -A-C.G
AAAAAAGCTAATGAAGACAAATTAAAACAAGAAGAAGCTCAAAAGAAAGCTGCTGAAGAACAGCGCAAACAACAAGAACAGCAGCAAGAGCAACAACGTAAACAGCAAGAA
GAAGCTCAA      s:4 i:0 d:2

1251856   ..G... .......... .....G  AA
s:1 i:1 d:0

1251881   .. ... .......... ......  GA
s:0 i:0 d:0

1251905   .. ... .......... A.....  G
s:1 i:0 d:0

1251928   .. ...G........A..GC..A-.
s:3 i:2 d:1
____________________________________________________________________________________________________

          AA AAA GCTGAAGATGA GGCTAA
s:11 i:5 d:3



('LFKB01000012', 235507, 235774)

235507   ............................  CGACGGGGTGCGGTAAAACCTTTGCGAACGC      s:0 i:0 d:0

235567   ............................  CGACGGGGTGCGGTAAAACCTTTGCGAACGC      s:0 i:0 d:0

235627   ...........................G  CTGTATTCGGCAAATGAAGTGGATGTTGATG      s:1 i:0 d:0

235687   .................T..........  TCGTACTTAACGAACTGTTGCTCCTCTGCTG      s:1 i:0 d:0
```

```
235747   ........T..A........A-.-A....                                        s:4 i:0 d:2
______________________________________________________________________________________________
         GTTCACTGCCGTACAGGCAGCTTAGAAAT                                        s:6 i:0 d:2


('JMJE01000011', 365419, 365686)
365419   ...........................  TCGACGGGGTGCGGTAAAACCTTTGCGAACGC      s:0 i:0 d:0
365479   ...........................  TCGACGGGGTGCGGTAAAACCTTTGCGAACGC      s:0 i:0 d:0
365539   ...........................  GCTGTATTCGGCAAATGAAGTGGATGTTGATG      s:0 i:0 d:0
365599   .................T.........  TTCGTACTTAACGAACTGTTGCTCCTCTGCTG      s:1 i:0 d:0
365659   ........T..A........A-.-A...                                         s:4 i:0 d:2
______________________________________________________________________________________________
         GTTCACTGCCGTACAGGCAGCTTAGAAA                                         s:5 i:0 d:2


('JFJM01000006', 141524, 141791)
141524   ...T........................  CGACGGGGTGCGGTAAAACCTTTGCGAACGC      s:1 i:0 d:0
141584   ............................  CGACGGGGTGCGGTAAAACCTTTGCGAACGC      s:0 i:0 d:0
141644   ...........................G  CTGTATTCGGCAAATGAAGTGGATGTTGATG      s:1 i:0 d:0
141704   .................T..........  TCGTACTTAACGAACTGTTGCTCCTCTGCTG      s:1 i:0 d:0
141764   ........T..A........A-.-A....                                        s:4 i:0 d:2
______________________________________________________________________________________________
         GTTCACTGCCGTACAGGCAGCTTAGAAAT                                        s:7 i:0 d:2


('JFJL01000011', 141524, 141791)
141524   ...T........................  CGACGGGGTGCGGTAAAACCTTTGCGAACGC      s:1 i:0 d:0
141584   ............................  CGACGGGGTGCGGTAAAACCTTTGCGAACGC      s:0 i:0 d:0
141644   ...........................G  CTGTATTCGGCAAATGAAGTGGATGTTGATG      s:1 i:0 d:0
141704   .................T..........  TCGTACTTAACGAACTGTTGCTCCTCTGCTG      s:1 i:0 d:0
141764   ........T..A........A-.-A....                                        s:4 i:0 d:2
______________________________________________________________________________________________
         GTTCACTGCCGTACAGGCAGCTTAGAAAT                                        s:7 i:0 d:2


('AVGM01000051', 129773, 130214)
129776   .. ... .................  GTCGAATTTATTTTAAATAAACTTAATAAAAAAAGTAAAATGACCGTAT      s:0 i:0 d:0
129848   .. ... .................  AGATTTTTCAAAAAAACTAAATGACCGT                           s:0 i:0 d:0
129899   ..A...T.................                                                         s:0 i:2 d:0
```

AT TCT CCGTACTTAACAAAAAAT s:0 i:2 d:0

('AVGM01000051', 129773, 130214)

129773 .... ATGTCGAATTTATTTTAAATAAACTTAATAAAAAAAGTAAAATGACCG s:0 i:0 d:0

129845 .... ATAGATTTTTCAAAAAAACTAAATGACCG s:0 i:0 d:0

129898 .....CT................ s:2 i:0 d:0

TATATTCTCCGTACTTAACAAAAA s:2 i:0 d:0

('AVIX01000072', 72663, 73104)

72665 ... ... ATGTCGAATTTATTTTAAATAAACTTAATAAAAAAAGTAAAATGACCGTA s:0 i:0 d:0

72737 ... ... ATAGATTTTTCAAAAAAACTAAATGACCG s:0 i:0 d:0

72788 ...A...T................ s:0 i:2 d:0

TAT TCT CCGTACTTAACAAAAA s:0 i:2 d:0

('AVIX01000072', 72663, 73104)

72664 .... AATGTCGAATTTATTTTAAATAAACTTAATAAAAAAAGTAAAATGACCGT s:0 i:0 d:0

72736 .... AATAGATTTTTCAAAAAAACTAAATGACCGT s:0 i:0 d:0

72789 ....CT............... s:2 i:0 d:0

ATATTCTCCGTACTTAACAAAA s:2 i:0 d:0

('AVMA01000025', 71125, 71566)

71127 ... ... ATGTCGAATTTATTTTAAATAAACTTAATAAAAAAAGTAAAATGACCGTA s:0 i:0 d:0

71199 ... ... ATAGATTTTTCAAAAAAACTAAATGACCG s:0 i:0 d:0

71250 ...A...T................ s:0 i:2 d:0

TAT TCT CCGTACTTAACAAAAA s:0 i:2 d:0

('AVMA01000025', 71125, 71566)

71126 .... AATGTCGAATTTATTTTAAATAAACTTAATAAAAAAAGTAAAATGACCGT s:0 i:0 d:0

71198 .... AATAGATTTTTCAAAAAAACTAAATGACCGT s:0 i:0 d:0

```
71251   ....CT...............                                          s:2 i:0 d:0
________________________________________________________________________________
        ATATTCTCCGTACTTAACAAAA                                         s:2 i:0 d:0
```

```
('ABHD02000060', 8119, 8560)
8126   .................-T.T  CGAATTTATTTTAAATAAACTTAATAAAAAAAGTAAAATGACCGTATATTC      s:2 i:0 d:1
8198   .....................  TTTTTCAAAAAAACTAAATGACCGTATATCT                        s:0 i:0 d:0
8251   .....................                                                      s:0 i:0 d:0
________________________________________________________________________________
       TCCGTACTTAACAAAAAATAGA                                                     s:2 i:0 d:1
```

```
('ABHD02000060', 8119, 8560)
8120   ........................  GTCGAATTTATTTTAAATAAACTTAATAAAAAAAGTAAAATGACCGT      s:0 i:0 d:0
8192   ........................  AGATTTTTCAAAAAAACTAAATGACCGT                         s:0 i:0 d:0
8245   ....CT..................                                                       s:2 i:0 d:0
________________________________________________________________________________
       ATATTCTCCGTACTTAACAAAAAAT                                                      s:2 i:0 d:0
```

```
('AFLS02000008', 208303, 208549)
208378   ...................G...  GCGTTGAGCACAGTTGATAGAGCGGTACTTCTAAAACTTTTTCCATCC       s:1 i:0 d:0
208450   .......................  GCGTTGAGCACTGTGTATATCTTGATTTACTCAAAAGTGATAATTCTAC      s:0 i:0 d:0
208523   .......................                                                          s:0 i:0 d:0
________________________________________________________________________________
         TGAATATAACTTTGATGCCGTTAG                                                         s:1 i:0 d:0
```

```
('JAIZ01000806', 138145, 138688)
138536   ........G.... GC...-G..-A  TGTGGATCTACCTGCTGCTGGAGGTCTACACCCACCGGTTGTT      s:4 i:1 d:2
138601   ............. ..........  GCGCGCTTCCCATTCTCGTGGGAGGCGTTCATGAACTTGCT         s:0 i:0 d:0
138665   .............G ..........                                                    s:0 i:1 d:0
________________________________________________________________________________
         ACCCCTCCCAGGG  TGATGAGACC                                                    s:4 i:2 d:2
```

```
('JAIY01000005', 153079, 153622)
153470   ........G.... GC...-G..-A  TGTGGATCTACCTGCTGCTGGAGGTCTACACCCACCGGTTGTT      s:4 i:1 d:2
```

```
153535   ............. ..........  GCGCGCTTCCCATTCTCGTGGGAGGCGTTCATGAACTTGCT        s:0 i:0 d:0

153599   .............G ..........                                                  s:0 i:1 d:0
______________________________________________________________________________________________________

         ACCCCTCCCAGGG  TGATGAGACC                                                  s:4 i:2 d:2



('JQQK01000100', 16161, 16598)

16491   T-T..A..C......A......  CAAAGTTATATTCAGCCTCTAAAGGAGAGAGGCCAAATCCGCGCATGGACA      s:4 i:1 d:1

16563   ........ .............  CAAAGTTATATTCAGTCGGCACAAATTAAGTTGATTGCAACAATTCCATT       s:0 i:0 d:0

16634   ........ .............                                                           s:0 i:0 d:0
______________________________________________________________________________________________________

        GTGCTCAA CGCCTACCGGCAT                                                           s:4 i:1 d:1



('CP013328', 1564136, 1564927)

1564155   .....................A. G  ATCTTCCAAAATGGTTGATCAATTCTATTCAAGTTTCCGTCTCCTTTTG              s:2 i:0 d:0

1564228   ............T.......... .  AAGTTTTGGGCTAAGGTAAGAGCAGGAGAGATTGGAGTGTTTCCGTCTCCTCTCA        s:1 i:0 d:0

1564307   ............T......-T.. .  TGGAAAAACTAATCATCTTACATCTTTACCTAACACAGAAGTTTCCGTCTCCTTTTG      s:2 i:0 d:1

1564387   ....................TT.T.                                                              s:2 i:1 d:0
______________________________________________________________________________________________________

          GAGTTATATTCTCTCTTATTAGA C                                                              s:7 i:1 d:1



('LGUP01000071', 13419, 13626)

13419   .......T.G..........-A  ACAGGGCGGCGGCCGGTCTTCCAGAGCGCGCGCCTCGGGA      s:3 i:0 d:1

13480   ...................A..  CAGGAGCCGACGACCATGAGGTGTCGGATCACGTCGGGG       s:1 i:0 d:0

13541   .............C........  CAGGTCGCCAGGCCGATCGCGTTCATCCGGGTCCAGGGA       s:1 i:0 d:0

13602   ......................  CAGACTCGACACCTGCCAGGTTAGGAGGCAGGGCCGGGA       s:0 i:0 d:0

13663   .............-A..A-C-A                                                s:4 i:0 d:3
______________________________________________________________________________________________________

        CCATCCCCGCGGGTGCGGGGAG                                                s:9 i:0 d:4



('JNWK01000103', 13419, 13626)

13419   .......T.G..........-A  ACAGGGCGGCGGCCGGTCTTCCAGAGCGCGCGCCTCGGGA      s:3 i:0 d:1

13480   ...................A..  CAGGAGCCGACGACCATGAGGTGTCGGATCACGTCGGGG       s:1 i:0 d:0

13541   .............C........  CAGGTCGCCAGGCCGATCGCGTTCATCCGGGTCCAGGGA       s:1 i:0 d:0

13602   ......................  CAGACTCGACACCTGCCAGGTTAGGAGGCAGGGCCGGGA       s:0 i:0 d:0

13663   .............-A..A-C-A                                                s:4 i:0 d:3
```

____

CCATCCCCGCGGGTGCGGGGAG s:9 i:0 d:4

('JOHG01000026', 3864, 4587)

3871 .... TGGCCACGGGCACGGTGACGACGTTCGACG s:0 i:0 d:0

3932 .... CCCGGTAGACGGAGACGGTGTGACCGGTGG s:0 i:0 d:0

3993 ..........TT...............T.-C s:4 i:0 d:1

____

CCGAGTCAGCCCCGCTCGCGCGGGCTCAACG s:4 i:0 d:1

('JOHG01000026', 3864, 4587)

4444 ..C..GC............... CACGCGGGCTCAACGCCGTGAGGTCATCGGCGTACTGCC s:3 i:0 d:0

4505 .... CACGCGGGCTCAACGCCGTGAGGTCATCGGCGTACTGCT s:0 i:0 d:0

4566 .... s:0 i:0 d:0

____

GCGAGTACGAGTCAGCCCCGCT s:3 i:0 d:0

('CP007466', 1696716, 1697293)

1697261 .... TCCGCGCAAAGCCGCGGTGGAGGCGGCCATCGCCCGCGCCAAAGCCCGCAAA
s:0 i:0 d:0

1697354 .... CCCGCGCAAAGCCGCGGTGGAAGCGGCTATCGCCCGCGCCAAAGCCCGCAAG
s:0 i:0 d:0

1697447 ....G...................... TCCGCGTAAAGCCGCGGTGGAAGCGGCCATCGCCCGCGCCAAAGCCCGTAAG
s:1 i:0 d:0

1697540 ....C...
s:1 i:0 d:0

____

CAGGAGCAGCAGGCCGGAAGCGAACCTGCCGAACCGGTCGA
s:2 i:0 d:0

('AZXF01000033', 12409, 12724)

12543 .................C-A..A......G..... CACCCGTGCGCCCCCTAGTGCTGCGCGACCAGCAGCAGCCT s:3 i:1 d:1

12618 .....A................ .... CCGGGCGACGGTCCGCTACCTCGGCCCGGACCTCCACCT s:1 i:0 d:0

12691 .... s:0 i:0 d:0

____

TCAACGTGGACGGCTCCTATGG AGCCGTAGACAC s:4 i:1 d:1

('AZXF01000033', 12409, 12724)

12409 ..-C.....C.G........... CTGCATGACCCAGTCCGCGTGGGATCGGCCGAACCTCCTTCAACGGAGAT s:3 i:0 d:1

12482 .... GTGTACGCCGAGGCGGACTTCAAGTCCGCTTACGCCCTTCAACGTGGAC s:0 i:0 d:1

12554 .... CACCCGTGCGCCCCCTAGTGCTGCGCGACCAGCAGCAGCCTTCAACATGGAC s:0 i:0 d:1

12629 ......T....-......A..... s:2 i:0 d:1

GGCTCCCATGGAAGCCGTGGACAC s:5 i:0 d:4

('JOHO01000007', 227200, 227867)

227194 . ... GGATGGTGTGGAGCGACCGACCCGGCCATTGCGAGGGGCGCAGCTGCTCCG s:0 i:0 d:0

227266 . ....T.............. AGCTGGTGCGGAGCAACTAAGCGGCCAATGGCCTCGCGACCGACGACGCG s:1 i:0 d:0

227337 .T..................G s:1 i:1 d:0

C GCTGCGATGGTGCCGCACCA s:2 i:1 d:0

('KK213723', 496053, 496673)

496074 .... CGGT s:0 i:0 d:0

496101 .... TGGAAAAGATGGTGCCAAAGGTGACCGCGGTGAAGCTGGT s:0 i:0 d:0

496164 .... s:0 i:0 d:0

CCTGCTGGTCCACGCGGTGAAGC s:0 i:0 d:0

('KK213723', 496053, 496673)

496561 ....C GGT s:1 i:0 d:0

496588 .... GGAAAAGATGGTGCCAAAGGTGACCGCGGTGAAGCTGGT s:0 i:0 d:0

496651 .... s:0 i:0 d:0

CCTGCTGGTCCACGCGGTGAAGCT s:1 i:0 d:0

('GL872408', 564164, 564420)

564227 ........T........T........ TAATTACCGTAACAACCGCAACTACCGTTACAAC s:2 i:0 d:0

564287 ....T..... CAATTATAGCTATAACTACGGCTACAATGGCTAT s:1 i:0 d:0

564347 .....C.............T..... TTATCCAGGCTATAACAACTGG s:2 i:0 d:0

564395 .....C......A---G......... s:3 i:0 d:3

AATTATAACTATGGTTACAACAACTA s:8 i:0 d:3

('KB291184', 8482, 10011)

9336 .... CTCACGCCCTTGCAGGTTAAAGTGTACTTTGTTGTTATCAATCG
s:0 i:0 d:0

9412 .... GAATTAGCTCGACAACAAGCCAGTTTCAACGTTGTTATCAATCA
s:0 i:0 d:0

9488 .... GACTTATTAACGCTGGTTATGAATGGGCTAGTTGTCATCAATCG
s:0 i:0 d:0

9564 .... ACCAACAAGTACTCTAAGCACCAAGCAATAGTTGTTATCAATCG
s:0 i:0 d:0

9640 .... ATCGGTCTGCCGTGCCGAATTTTTGCAATTGTTGTTATCAATCG
s:0 i:0 d:0

9716 ....
TACAACTTTTTTGCGTATTTTTTTTATATTGTTGTTATCAATCGCAAAACTACAAAATTTGNNNNNNNNNNNNNNNNNNNN s:0 i:0 d:0

9829 .... CTTTTCCGTTGAGTTGCTTGTACTTCTTTAGTTGTTATCAATCA
s:0 i:0 d:0

9905 ........T....A.................. GCCTTACGGGGCTGGAGAAAAGGGATTGTGTTGTTATCAATTG
s:2 i:0 d:0

9980 .....G.............A.........A
s:3 i:0 d:0

CAAAACTACAAAATTTGAAAGCAATTCACAAC
s:5 i:0 d:0

('ALXU01000028', 76074, 76587)

76075 ....CG.C TTGTTCGGTTTCGGTTTTTTTTGAGGTTTCGGGCAACTTCTAAAC s:3 i:0 d:0

76149 .... CCTTAGAATAACAGCAATATTCAAAGATTATAAAAGACC s:0 i:0 d:0

76217 ....T.............. s:1 i:0 d:0

CGTCATTCCCGCGCAGGCGGGAATCTAGA s:4 i:0 d:0

('KK211379', 64070, 65100)

65004 .... TAACTATTCTATCCTACAATCCCCAATTTCAAAGTGTCTCCA s:0 i:0 d:0

65072 ....-.... s:0 i:0 d:1

65097 --....-.........C......A.A s:3 i:0 d:3

CCTAAAAATAAAATATATAAAAATAT s:3 i:0 d:4

('AZMK01000015', 9716, 9979)

9794   ...C.A.T.................-.AG. ATTGGCTATCAATTCAATATCTTGTTAAATGCAGATGTAGTATTTCCGTC   s:3 i:2 d:1

9874   ....A............. .. AGAGAGAATGTCTCGCACTAATTTAAATACTATGGCTGAATATCCGCC   s:1 i:0 d:0

9951   ....   s:0 i:0 d:0

____

CCCATCA GGGGATTATTTAATTTAACC AA   s:4 i:2 d:1

('AZMK01000015', 9716, 9979)

9796   .C.A.T.................-.AG. ATTGGCTATCAATTCAATATCTTGTTAAATGCAGATGTAGTATTTCCGTCCC   s:3 i:2 d:1

9876   ....A............. .. AGAGAGAATGTCTCGCACTAATTTAAATACTATGGCTGAATATCCGCCCC   s:1 i:0 d:0

9953   ....   s:0 i:0 d:0

____

CATCA GGGGATTATTTAATTTAACC AA   s:4 i:2 d:1

('KK340196', 937632, 939001)

937632   .... TGGCGGAATGGGCGATGGCGGCGGGAACGGCGGTGCCGGCGGG
s:0 i:0 d:0

937722   .... CGGCGGCACGTCGGTTGCCACCGGGGGGGCCGGGAACGGCGGTGCCGGCGGC
s:0 i:0 d:0

937821   ....C.....
s:1 i:0 d:0

____

GCCGGCGGCGGGGCCGGGCTGATCGGCAACGGCGGCAACGGTGGCAG
s:1 i:0 d:0

('KK340196', 937632, 939001)

937632   .... TGGCGGAATGGGCGATGGCGGCGGGAACGGCGGT   s:0
i:0 d:0

937713   ....   G   s:0
i:0 d:39

937722   .... CGGCGGCACGTCGGTTGCCACCGGGGGGGCCGGGAACGGCGGT   s:0
i:0 d:0

937812   ....   s:0
i:0 d:38

____

GCCGGCGGCGGGGCCGGGCTGATCGGCAACGGCGGCAACGGTGGCAG   s:0
i:0 d:77

('LAGJ01000229', 57354, 57577)

57358   .... CAACTTATAGGATAACTTTAGTTCCCTAGTGTAGCATTTATTT   s:0 i:0 d:0

57423   .....T............... CAATACAAAAATAAGCGATATCATAGTGCCAGAGGTATTATTT   s:1 i:0 d:0

57488 ..........T......C.... s:2 i:0 d:0

AAATACATCTCATGTTATTGTT s:3 i:0 d:0

('LAGJ01000229', 57354, 57577)

57421 .......T....C......T...... ATACAAAAATAAGCGATATCATAGTGCCAGAGGTATTAT s:3 i:0 d:0

57486 .... ACTAATTCAATCTGAACAATTAAAAGTTAGATATAAAT s:0 i:0 d:0

57550 .... s:0 i:0 d:0

TTAAATACATCTTATGTTACTGTTCA s:3 i:0 d:0

('LAGI01000658', 14536, 14759)

14540 .... CAACTTATAGGATAACTTTAGTTCCCTAGTGTAGCATTTATTT s:0 i:0 d:0

14605 .....T................ CAATACAAAAATAAGCGATATCATAGTGCCAGAGGTATTATTT s:1 i:0 d:0

14670 ..........T......C.... s:2 i:0 d:0

AAATACATCTCATGTTATTGTT s:3 i:0 d:0

('LAGI01000658', 14536, 14759)

14603 .......T....C......T...... ATACAAAAATAAGCGATATCATAGTGCCAGAGGTATTAT s:3 i:0 d:0

14668 .... ACTAATTCAATCTGAACAATTAAAAGTTAGATATAAAT s:0 i:0 d:0

14732 .... s:0 i:0 d:0

TTAAATACATCTTATGTTACTGTTCA s:3 i:0 d:0

('JOJM01000027', 89004, 89180)

89063 -G.C.T....C............. CCCGCCTGCGAGGCCGGGCTGCCCATCTGAGAGACCGA s:4 i:0 d:1

89125 ....A.... CCCGTCTGCGGAAACGG s:1 i:0 d:0

89167 ...............G...A..... s:2 i:0 d:0

GCTGCCCGTCTGCGGAAACGGGCTG s:7 i:0 d:1

('JH791809', 1035351, 1035652)

1035582 .... TTCTTGTTTTAGTTTATCTTCATTGGTCTTTTTCAGGGCTTCT s:0 i:0 d:0

1035648   .... T   s:0 i:0 d:0

1035672   .............---C..A..   s:2 i:0 d:3

____

TCGTCAGCTTTTTTCTTAGCTTC   s:2 i:0 d:3

('JH791809', 1035351, 1035652)

1035351   ......T.TC .... CA
s:3 i:0 d:0

1035375   .... CG
s:0 i:0 d:0

1035399   .... CG
s:0 i:0 d:0

1035423   ....C....... CAGCG
s:1 i:0 d:0

1035450   ....-...T.TG........-C.
TGCTGTTTACGTTGTTGTTCTTGTTGCTGCTGTTGTTCTTGTTGTTTACGTTGTTGTTCTTGTTGCTGTTGCTGTTGTTGTTGTTGCTGTTCTTGCTGTTTACGCTGATCT
TCGTCA   s:3 i:1 d:2

1035588   ......T... ....T.
s:2 i:0 d:0

____

GCTTTTCTCT TAGCTTCTTCGT
s:9 i:1 d:2

('JH791809', 1035351, 1035652)

1035580   .... TTCTTGTTTTAGTTTATCTTCATTGGTCTTTTTCAGGGCTT   s:0 i:0 d:0

1035646   ....   s:0 i:0 d:0

1035671   -...............GC.T.--T   s:4 i:0 d:3

____

CTTCGTCAGCTTTTTTCTTAGCTTC   s:4 i:0 d:3

('AEXR01000024', 76098, 76310)

75977   ....
AGTTCCTTTCAATCGAGACTTTCGATGATGGCTCCCCCGTCTCCCTTCGTGTTTCGAAAGCGAATCGCCTTAAACCTGATTCTCCTTTGTCCTTACGCTA   s:0
i:0 d:0

76100   .... AAAGGGTCAAGAGCTCGGTTTATTGTCGTG
s:0 i:0 d:0

76153   .G...............A... . CAATGGTCTAACCTCAGTCTGTTTCGCGTCATG
s:2 i:0 d:0

76209   .................C.CTT. CCTGAGGTGCGTTTCGCTCA
s:3 i:1 d:0

76253   ....C..............T. .
s:2 i:0 d:0

____

AATGTTTCACGTGAAACATTAG A
s:7 i:1 d:0

('AEXR01000024', 76098, 76310)

76152 ..G................A. AGACAATGGTCTAACCTCAGTCTGTTTCGCGTCAT s:2 i:0 d:0

76208 ....C. CTTACCTGAGGTGCGTTTCGCTC s:1 i:0 d:0

76252 A....C............... TGAAACGTCGTGATTT s:2 i:0 d:0

76289 .... s:0 i:0 d:0

GAATGTTTCACGTGAAACATT s:5 i:0 d:0

('JFYZ01000001', 618466, 618692)

618519 . ... ATTGTTGTCTCCGCGGC s:0 i:0 d:0

618557 .A..A..A..G..... ...... ATTGTTGTCTCCGCGACG s:3 i:1 d:0

618597 . ... A..... GTTGTCGTCTCGGCGGTTGTTGTCCGCGCGCCATGCATTGTTGCGGTCGCGGTT s:1 i:0 d:0

618672 . ....C......G.GGT-.... s:4 i:1 d:1

G TCGTTGCCTCGCCA GCCGTT s:8 i:2 d:1

('AVKQ01000204', 8177, 8389)

8056 ....
GAAGTTCCTTTCAATCGAGACTTTCGATGATGGCTCCCCCGTCTCCCTTCGTGTTTCGAAAGCGAATCGCCTTAAACCTGATTCTCCTTTGTCCTTACGCTA s:0
i:0 d:0

8179 .... GAAAAGGGTCAAGAGCTCGGTTTATTGTCGTG
s:0 i:0 d:0

8232 .G................A.. GACAATGGTCTAACCTCAGTCTGTTTCGCGTCATG
s:2 i:0 d:0

8288 ....C.C TTACCTGAGGTGCGTTTCGCTCA
s:2 i:0 d:0

8332 ....C...............T
s:2 i:0 d:0

AATGTTTCACGTGAAACATTA
s:6 i:0 d:0

('AVKQ01000204', 8177, 8389)

8231 ..G................A.A GACAATGGTCTAACCTCAGTCTGTTTCGCGTCAT s:3 i:0 d:0

8287 ....C.. TTACCTGAGGTGCGTTTCGCTC s:1 i:0 d:0

8331 A....C..............-T TGAAACGTCGTGATTT s:3 i:0 d:1

8368 .... s:0 i:0 d:0

GAATGTTTCACGTGAAACATTC s:7 i:0 d:1

('CP010089', 1742810, 1743099)

1742962 .... CTTAGCTTCGTCTTGTTTTACTTTATCTTCATTAGCCTTTTTCTGGGCTTCCTCATCAGCTTT s:0 i:0 d:0

1743052 .........T.....A..G........ s:3 i:0 d:0

1743079 ---.................T..... s:1 i:0 d:3

TTTCTTAGCCTCTTCTTCAGCCTTTTT s:4 i:0 d:3

('CP009422', 1251145, 1251654)

1251487 ....A............ GGTCTTTTATAATCTTTGAATATTGCTGTTGTTCTAAGGTCTA s:1 i:0 d:0

1251555 .... GTTTAGAAGTTGCCCGAAACCTCAAAAAAAACCGAAACCGAACAAGCCG s:0 i:0 d:0

1251629 .... s:0 i:0 d:0

GATTCCCGCCTGCGCGGGAATGACG s:1 i:0 d:0

('LABV01000049', 11477, 11766)

11550 .-..-.......C.......-.... CTTTTGAGCTTCTTCTTGCTGTTTACGTTGTTGTTCTTGCTGTTGCTGTTCTTGTTG s:1 i:0 d:3

11629 .........C.....T......... TTCTTAGCTTCGTCTTGTTTTACTTTATCTTCATTAGCCTTTTTCTGGGCTTCCTCATCAGCTTT s:2 i:0 d:0

11719 ....G...... s:1 i:0 d:0

11744 .-.......C.....T....-.... s:2 i:0 d:2

TTTCTTAGCTTCTTCATCAGCCTTT s:6 i:0 d:5

('LABP01000119', 11477, 11766)

11550 .-..-.......C.......-.... CTTTTGAGCTTCTTCTTGCTGTTTACGTTGTTGTTCTTGCTGTTGCTGTTCTTGTTG s:1 i:0 d:3

11629 .........C.....T......... TTCTTAGCTTCGTCTTGTTTTACTTTATCTTCATTAGCCTTTTTCTGGGCTTCCTCATCAGCTTT s:2 i:0 d:0

11719 ....G...... s:1
i:0 d:0

11744 .-.......C.....T....-.... s:2
i:0 d:2

TTTCTTAGCTTCTTCATCAGCCTTT s:6
i:0 d:5

('HG810017', 167902, 168191)

168055 .... TTAGCTTCGTCTTGTTTTACTTTATCTTCATTAGCCTTTTTCTGGGCTTCCTCATCAGCTTTT s:0
i:0 d:0

168145 ........T.....A..G......... s:3
i:0 d:0

168172 .---...............T...... s:1
i:0 d:3

TTCTTAGCCTCTTCTTCAGCCTTTTTC s:4
i:0 d:3

('APUC01000016', 13096, 13637)

13096 ...........A.............-C. GTTATTTAAAACTGCAGAAATTTATCCGAAGCAACAACAATCTTTCCAT s:2 i:0 d:1

13172 ....T............. CAACGAAAAGTAACAGGAATTTATCGGGAAAACAGAAACCTCTATGC s:1 i:0 d:0

13247 .... s:0 i:0 d:0

CGTCATTCCCGCGCAGGCGGGAATCTAG s:3 i:0 d:1

('APUC01000016', 13096, 13637)

13098 ....A.............-C GGTTATTTAAAACTGCAGAAATTTATCCGAAGCAACAACAATCTTTCCATCG s:2 i:0 d:1

13174 ....T............ GCAACGAAAAGTAACAGGAATTTATCGGGAAAACAGAAACCTCTATGCCG s:1 i:0 d:0

13249 .... s:0 i:0 d:0

TCATTCCCGCGCAGGCGGGAATCTA s:3 i:0 d:1

('JREU01000001', 662472, 662981)

662815 ....A............. GTCTTTTATAATCTTTGAATATTGCTGTTGTTCTAAGGTCTAG s:1 i:0 d:0

662883 .... TTTAGAAGTTGCCCGAAACCTCAAAAAAAAACCGAAACCGAACAAGCCGG s:0 i:0 d:0

662957 .... s:0 i:0 d:0

ATTCCCGCCTGCGCGGGAATGACGG s:1 i:0 d:0

```
('JRET01000004', 380345, 380854)

380688   ..........A.............  GTCTTTTATAATCTTTGAATATTGCTGTTGTTCTAAGGTCTAG           s:1 i:0 d:0

380756   ........................  TTTAGAAGTTGCCCGAAACCTCAAAAAAAACCGAAACCGAACAAGCCGG     s:0 i:0 d:0

380830   ........................                                                        s:0 i:0 d:0
_____________________________________________________________________________________________________

         ATTCCCGCCTGCGCGGGAATGACGG                                                       s:1 i:0 d:1


('CP008933', 10628, 11144)

10633   .. ...................  ACTCATTACGGGTGTCCATTCGGATCTGGTAGTGCCGA      s:0 i:0 d:1

10693   ......................  ACTTTAATCCACTGCCGGAAACAGGAGTCCGGAACCGA      s:0 i:0 d:0

10754   ..........CA.....-....                                              s:2 i:0 d:1
_____________________________________________________________________________________________________

        TAACCCCCGCATGCGGGGGGAAT                                             s:2 i:0 d:2


('CP008933', 10628, 11144)

10750   .....................-......C  GGCTGTTTCAGCTGATTTTTTTTAAAAGTCA      s:1 i:0 d:1

10810   ..............GG............  TTAATCCACTGCCGGAAACAGGAGTCCGGAA      s:2 i:0 d:0

10871   .............T.............A  GCAGCAGCTGACCAACTGACGGAAATCCAGC      s:2 i:0 d:0

10932   ..............TG............  ACTAACAATATTTAACGAACGCCAACACACA      s:2 i:0 d:0

10993   ..............TG............  TCCAGCCAGCGGGTAACCGTGGACAACACCA      s:2 i:0 d:0

11054   ............................  ATTCAGCCAGTTCTCCTGGGCACCGGGTCGG      s:0 i:0 d:0

11115   ............................                                       s:0 i:0 d:0
_____________________________________________________________________________________________________

        CCGATAACCCCCGCACACGGGGGGAATACT                                     s:9 i:0 d:1


('CDQG01000075', 419, 935)

423   ... ......................  CATTACGGGTGTCCATTCGGATCTGGTAGTGCCG      s:0 i:0 d:1

483   ..........................  TTAATCCACTGCCGGAAACAGGAGTCCGGAACCG      s:0 i:0 d:0

544   ...........CA.....-......C                                          s:3 i:0 d:1
_____________________________________________________________________________________________________

      ATAACCCCCGCATGCGGGGGGAATACT                                         s:3 i:0 d:2


('CDQG01000075', 419, 935)
```

```
541    .....................-......C  GGCTGTTTCAGCTGATTTTTTTTAAAAGTCA      s:1 i:0 d:1
601    ...............GG............  TTAATCCACTGCCGGAAACAGGAGTCCGGAA      s:2 i:0 d:0
662    ..............T.............A  GCAGCAGCTGACCAACTGACGGAAATCCAGC      s:2 i:0 d:0
723    ...............TG............  ACTAACAATATTTAACGAACGCCAACACACA      s:2 i:0 d:0
784    ...............TG............  TCCAGCCAGCGGGTAACCGTGGACAACACCA      s:2 i:0 d:0
845    .............................  ATTCAGCCAGTTCTCCTGGGCACCGGGTCGG      s:0 i:0 d:0
906    .............................                                       s:0 i:0 d:0
_____________________________________________________________________________________________
       CCGATAACCCCCGCACACGGGGGGAATACT                                      s:9 i:0 d:1


('AZRS01000121', 15947, 16181)
16014   ............CT..............-T.--T.  TGATATAAGTTATTTCTATGACAATACAAGAAAA      s:4 i:0 d:3
16080   ...................................  ATGTGGTAACTTATAAATATCTATAAAATAA         s:0 i:0 d:0
16146   ..............................C....                                          s:1 i:0 d:0
_____________________________________________________________________________________________
        TTTAAATACATCTCATGTTAATGTTCAATGAAAAT                                          s:5 i:0 d:3


('AZRS01000121', 15947, 16181)
16011   .T.............CT..............-T.-T  TTGATATAAGTTATTTCTATGACAATACAAGAA      s:4 i:1 d:2
16078   . ..................................  ATATGTGGTAACTTATAAATATCTATAAAAT        s:0 i:0 d:0
16144   . ..............................C..                                          s:1 i:0 d:0
_____________________________________________________________________________________________
        A ATTTAAATACATCTCATGTTAATGTTCAATGAAA                                         s:5 i:1 d:2


('AEQZ01000044', 114, 362)
115    ................ .......  TCTGGTTCGTTCGGTTTCGCTTGTTTTAAGTTTCGGGTAACTTCCACT         s:0 i:0 d:0
187    ................ .......  TCCAGTGCGTTGAGTTTCAGCTATTTAGAATAAATTTTGAAACTCTAATCC      s:0 i:0 d:0
262    -..........A..A.AG-T.....                                                          s:4 i:1 d:2
_____________________________________________________________________________________________
       TCGTCATTCCCGCGCAG GCGGGAA                                                          s:4 i:1 d:2


('APTF01000126', 16539, 16786)
16614   ........................CG.  CTTGTTCGGTTTCGGTTTTTTTTGAGGTTTCGGGCAACTTCTAAAC      s:2 i:0 d:0
16688   ...........................  ACCTTAGAACAACAGCAATATTCAAAGATTATAAAAGACC            s:0 i:0 d:0
16756   ............T.............                                                        s:1 i:0 d:0
```

___

CGTCATTCCCGCGCAGGCGGGAATCTAG s:3 i:0 d:0

('JQQI01000181', 392, 826)

384 -T..A..TT.....A....... AAAGTTATATTCAGAAAACGATACAAAGACTAGATAGTTAAACTTTCCAG s:4 i:1 d:1

455 .... AAAGTTATATTCAGATACATCAATCCTTCTGGGAATCAAATTCCGTCATTG s:0 i:0 d:0

527 ....A.... s:1 i:0 d:0

___

TGCTCAA CGCCTACCGGCATC s:5 i:1 d:1

('JQPF01000311', 336, 773)

618 ......T...... .... AGTTAATCAAAAAGATTCTGAGTATTTTAGAAGAGCTGAATATAACTTTG s:1 i:0 d:0

689 .... CGAAAAAATTTTCAATTTCTAACACTTCGATTCAAGCTGAATATAACTTTG s:0 i:0 d:0

761 .............A..T.-G.. s:2 i:1 d:1

___

ATGCCGGTAGGCG TTGAGCAC s:3 i:1 d:1

('KQ236283', 146, 394)

147 .... ATCTGGTTCGTTCGGTTTCGCTTGTTTTAAGTTTCGGGTAACTTCCACT s:0 i:0 d:0

219 .... ATCCAGTGCGTTGAGTTTCAGCTATTTAGAATAAATTTTGAAACTCTAATCC s:0 i:0 d:0

294 -..........A..A.AG-T.... s:4 i:1 d:2

___

TCGTCATTCCCGCGCAG GCGGGA s:4 i:1 d:2

('KQ236157', 146, 394)

147 .... ATCTGGTTCGTTCGGTTTCGCTTGTTTTAAGTTTCGGGTAACTTCCACT s:0 i:0 d:0

219 .... ATCCAGTGCGTTGAGTTTCAGCTATTTAGAATAAATTTTGAAACTCTAATCC s:0 i:0 d:0

294 -..........A..A.AG-T.... s:4 i:1 d:2

___

TCGTCATTCCCGCGCAG GCGGGA s:4 i:1 d:2

('JWCO01000046', 24005, 25007)

24009 .... TGGTTATCGAATAAATATGGATCAAAATTCTATGATGCTCGTTTCTTCCCGTT s:0 i:0 d:0

24083 ....T..... CAGTTATCGATCGTATTTCCTATTCAAATCACTACAAAAACTACTCCGTT s:1 i:0 d:0

24154   .........C.C..T.A.....   s:3 i:1 d:0

CTCGTCCCC TGTTCTCCGGGG   s:4 i:1 d:0

('AKGC01000007', 3801, 4849)

3802   .... TGGTTATCGAATAAATATGGATCAAAATTCTATGATGCTCGTTTCTTCCC   s:0 i:0 d:0

3876   ....T..... CAGTTATCGATCGTATTTCCTATTCAAATCACTACAAAAACTACTCC   s:1 i:0 d:0

3947   ...........C.C..T.A.....   s:3 i:1 d:0

GTTCTCGTCCCC TGTTCTCCGGGG   s:4 i:1 d:0

('KL590673', 28153, 28370)

28155   ....G..................... GGCGTTGTTCGCGGCACTCGGGATGGCCTACACCT   s:1 i:0 d:0

28216   .... GGTGGCCGACGTTGCCGGGGCCTTTGACCAGGACT   s:0 i:0 d:0

28277   ....T...............   s:1 i:0 d:0

CCTCCCCGCACCCGCGGGGGTCAGCC   s:2 i:0 d:0

('KL590673', 28153, 28370)

28158   .G.................... CGGCGTTGTTCGCGGCACTCGGGATGGCCTACACCTCCTC   s:0 i:1 d:0

28220   . ... CGGTGGCCGACGTTGCCGGGGCCTTTGACCAGGACTCCTC   s:0 i:0 d:0

28281   . .....T.............   s:1 i:0 d:0

C CCGCACCCGCGGGGGTCAGC   s:1 i:1 d:0

('JOAB01000027', 39023, 39228)

39023   .... TCCCCGAGGACGCCACCTCGGGTCCGGACACCGGAT   s:0 i:0 d:0

39084   .... TCCCTGAGCGGCAGCGTGTACGCCCACGCCACCGAC   s:0 i:0 d:0

39145   ....T..........   s:1 i:0 d:0

GTGCTCCCCGCGCCCGCGGGGATGG   s:1 i:0 d:0

('JOAB01000027', 39023, 39228)

39023   .... GTCCCCGAGGACGCCACCTCGGGTCCGGACACCGGAT   s:0 i:0 d:0

39084 .... GTCCCTGAGCGGCAGCGTGTACGCCCACGCCACCGAC s:0 i:0 d:0

39145 ....T......... s:1 i:0 d:0

GTGCTCCCCGCGCCCGCGGGGATG s:1 i:0 d:0

('JOAB01000027', 39023, 39228)

39026 .... CGAGGACGCCACCTCGGGTCCGGACACCGGATGTG s:0 i:0 d:0

39087 .... TGAGCGGCAGCGTGTACGCCCACGCCACCGACGTG s:0 i:0 d:0

39148 ..........T.........-G..G s:3 i:0 d:1

CTCCCCGCGCCCGCGGGGATGGTCCC s:3 i:0 d:1

('CP001561', 43608, 44117)

43610 .... GGGATAACAGCAATATTCAAAGGTTATAAAAGACCT s:0 i:0 d:0

43678 ....C....... GGGATAACAGCAATATTCAAAGGTTATAAAAGACCC s:1 i:0 d:0

43746 .... s:0 i:0 d:0

GTCATTCCCGCGCAGGCGGGAATCTAGACCTT s:1 i:0 d:0

('CP001561', 43608, 44117)

43611 .... TGGGATAACAGCAATATTCAAAGGTTATAAAAGACCTG s:0 i:0 d:0

43679 ....C...... TGGGATAACAGCAATATTCAAAGGTTATAAAAGACCCG s:1 i:0 d:0

43747 .... s:0 i:0 d:0

TCATTCCCGCGCAGGCGGGAATCTAGACCT s:1 i:0 d:0

('APTY01000001', 318, 827)

660 ....A.......... CGGGTCTTTTATAATCTTTGAATATTGCTGTTGTTCTAAGGTCTA s:1 i:0 d:0

728 .... CGGTTTAGAAGTTGCCCGAAACCTCAAAAAAAAACCGAAACCGAACAAGCCG s:0 i:0 d:0

802 .... s:0 i:0 d:0

GATTCCCGCCTGCGCGGGAATGA s:1 i:0 d:0

('ALXN01000004', 97, 539)

98 .... CTGGTTCGTTCGGTTTCGCTTGTTTTAAGTTTCGGGTAACTTCCACT s:0 i:0 d:0

170 .... CCAGTGCGTTGAGCTTCAGCTATTTAGAATAAATTTTGAAACTCTAATCG s:0 i:0 d:0

245 -..........A..A.AG-T...... s:4 i:1 d:2

TCGTCATTCCCGCGCAG GCGGGAAT s:4 i:1 d:2

('ANRO01000006', 87879, 88388)

87881 .... TCCGGCTTGTTCGGTTTCGGTTTTTTTTGAGGTTTCGGGCAACTTCTAAACC s:0 i:0 d:0

87955 .... TCTAGACCTTAGAACAACAGCAATATTCAAAGATTATAAAAGACCC s:0 i:0 d:0

88023 ....T......... s:1 i:0 d:0

GTCATTCCCGCGCAGGCGGGAA s:1 i:0 d:0

('AVNW01000004', 234, 974)

233 .... TCCAGAACGTAAAATCTAAAGAAACCGTGTTGTAACGGCAGACCGAT s:0 i:0 d:0

305 ....T......... TCTAGACCTTGGGATAACGGCAATATTCAAAGTTTATAAAAGA s:1 i:0 d:0

373 C....T.................. s:2 i:0 d:0

GCCGTCATTCCCGCGCAGGCGGGAA s:3 i:0 d:0

('AVNW01000004', 234, 974)

241 ....C...A CGTAAAATCTAAAGAAACCGTGTTGTAACGGCAGACCGATGCCGTCAT s:2 i:0 d:0

313 ....T............... CTTGGGATAACGGCAATATTCAAAGTTTATAAAAGACCCGTTAT s:1 i:0 d:0

381 .... s:0 i:0 d:0

TCCCGCGCAGGCGGGAATCTAGAC s:3 i:0 d:0

('AVGW01000014', 4567, 4919)

4570 .. .. GTCGAATTTATTTTAAATAAACTTAATAAAAAAAGTAAAATGACCGTAT s:0 i:0 d:0

4642 .. .. AGATTTTTCAAAAAAACTAAATGACCGT s:0 i:0 d:0

4693 ..A...T................. s:0 i:2 d:0

AT TCT CCGTACTTAACAAAAAAT s:0 i:2 d:0

('AVGW01000014', 4567, 4919)

4567 .... ATGTCGAATTTATTTAAATAAACTTAATAAAAAAAGTAAAATGACCG s:0 i:0 d:0

4639 .... ATAGATTTTTCAAAAAAACTAAATGACCG s:0 i:0 d:0

4692 .....CT................ s:2 i:0 d:0

TATATTCTCCGTACTTAACAAAAA s:2 i:0 d:0

('AGBP01000056', 21, 535)

367 ....G-G. TTGTTCGGTTTCGGTTTTGGTTTTTTTTGAGGTTTCGGGCAACTTCTAAACCGTC s:2 i:0 d:1

447 ....T.... CTTAGAACAACAGCAATATTCAAAGATTATAAAAGACCTGTC s:1 i:0 d:0

515 .... s:0 i:0 d:0

ATTCCCGCGCAGGCGGGAATCCAGAC s:3 i:0 d:1

('AGBS01000038', 135, 531)

355 .... GGGTCTTTTATAATCTTTGAATATTACTGTTGTTCTAAGGTCTA s:0 i:0 d:0

423 .... GGTTTAGAAGTTGCCCGAAACCTCAAAAAAAAAAAAAAAAACCGAAACCGAACAAGCCG s:0 i:0 d:0

506 .... s:0 i:0 d:0

GATTCCCGCCTGCGCGGGAATGAC s:0 i:0 d:0

('APSP01000108', 15806, 16464)

16367 .... TTGGGATAACGGCAATATTCAAAGGTTATAAAAGACCC
s:0 i:0 d:0

16435 ....T..... TTTCGTGGGAATGACGGGATGTAGGTTCGTAGGAATGACGTGGTGCAGGTTTCCGTATGGATGGATTC
s:1 i:0 d:0

16533 ....
s:0 i:0 d:0

GTCATTCCCGCGCAGGCGGGAATCCAGACC
s:1 i:0 d:0

('AVHP01000900', 1518, 1959)

1521 .. .. GTCGAATTTATTTAAATAAACTTAATAAAAAAAGTAAAATGACCGTAT s:0 i:0 d:0

1593 .. .. AGATTTTTCAAAAAAACTAAATGACCGT s:0 i:0 d:0

1644 ..A...T.................. s:0 i:2 d:0

```
      AT TCT CCGTACTTAACAAAAAAT                                                      s:0 i:2 d:0


('AVHP01000900', 1518, 1959)

1518  ........................  ATGTCGAATTTATTTTAAATAAACTTAATAAAAAAAGTAAAATGACCG      s:0 i:0 d:0

1590  ........................  ATAGATTTTTCAAAAAAACTAAATGACCG                         s:0 i:0 d:0

1643  .....CT.................                                                        s:2 i:0 d:0
_____________________________________________________________________________________________
      TATATTCTCCGTACTTAACAAAAA                                                        s:2 i:0 d:0


('AVHQ01000017', 5514, 5955)

5517  .. ... ..................  GTCGAATTTATTTTAAATAAACTTAATAAAAAAAGTAAAATGACCGTAT     s:0 i:0 d:0

5589  .. ... ..................  AGATTTTTCAAAAAAACTAAATGACCGT                         s:0 i:0 d:0

5640  ..A...T..................                                                       s:0 i:2 d:0
_____________________________________________________________________________________________
      AT TCT CCGTACTTAACAAAAAAT                                                       s:0 i:2 d:0


('AVHQ01000017', 5514, 5955)

5514  ........................  ATGTCGAATTTATTTTAAATAAACTTAATAAAAAAAGTAAAATGACCG      s:0 i:0 d:0

5586  ........................  ATAGATTTTTCAAAAAAACTAAATGACCG                         s:0 i:0 d:0

5639  .....CT.................                                                        s:2 i:0 d:0
_____________________________________________________________________________________________
      TATATTCTCCGTACTTAACAAAAA                                                        s:2 i:0 d:0


('AVHS01000013', 4683, 5124)

4685  ... ... ................  ATGTCGAATTTATTTTAAATAAACTTAATAAAAAAAGTAAAATGACCGTA    s:0 i:0 d:0

4757  ... ... ................  ATAGATTTTTCAAAAAAACTAAATGACCG                         s:0 i:0 d:0

4808  ...A...T................                                                        s:0 i:2 d:0
_____________________________________________________________________________________________
      TAT TCT CCGTACTTAACAAAAA                                                        s:0 i:2 d:0


('AVHS01000013', 4683, 5124)

4684  ......................  AATGTCGAATTTATTTTAAATAAACTTAATAAAAAAAGTAAAATGACCGT      s:0 i:0 d:0

4756  ......................  AATAGATTTTTCAAAAAAACTAAATGACCGT                         s:0 i:0 d:0

4809  ....CT................                                                          s:2 i:0 d:0
```

___

ATATTCTCCGTACTTAACAAAA s:2 i:0 d:0

('AVHU01000021', 5514, 5955)

5516 ... ... ATGTCGAATTTATTTTAAATAAACTTAATAAAAAAAGTAAAATGACCGTA s:0 i:0 d:0

5588 ... ... ATAGATTTTTCAAAAAAACTAAATGACCG s:0 i:0 d:0

5639 ...A...T............... s:0 i:2 d:0

___

TAT TCT CCGTACTTAACAAAAA s:0 i:2 d:0

('AVHU01000021', 5514, 5955)

5515 .... AATGTCGAATTTATTTTAAATAAACTTAATAAAAAAAGTAAAATGACCGT s:0 i:0 d:0

5587 .... AATAGATTTTTCAAAAAAACTAAATGACCGT s:0 i:0 d:0

5640 ....CT............... s:2 i:0 d:0

___

ATATTCTCCGTACTTAACAAAA s:2 i:0 d:0

('AVHY01000016', 5232, 5673)

5234 ... ... ATGTCGAATTTATTTTAAATAAACTTAATAAAAAAAGTAAAATGACCGTA s:0 i:0 d:0

5306 ... ... ATAGATTTTTCAAAAAAACTAAATGACCG s:0 i:0 d:0

5357 ...A...T............... s:0 i:2 d:0

___

TAT TCT CCGTACTTAACAAAAA s:0 i:2 d:0

('AVHY01000016', 5232, 5673)

5233 .... AATGTCGAATTTATTTTAAATAAACTTAATAAAAAAAGTAAAATGACCGT s:0 i:0 d:0

5305 .... AATAGATTTTTCAAAAAAACTAAATGACCGT s:0 i:0 d:0

5358 ....CT............... s:2 i:0 d:0

___

ATATTCTCCGTACTTAACAAAA s:2 i:0 d:0

('AVID01000008', 62827, 63268)

62829 ... ... ATGTCGAATTTATTTTAAATAAACTTAATAAAAAAAGTAAAATGACCGTA s:0 i:0 d:0

62901 ... ... ATAGATTTTTCAAAAAAACTAAATGACCG s:0 i:0 d:0

62952 ...A...T............... s:0 i:2 d:0

TAT TCT CCGTACTTAACAAAAA s:0 i:2 d:0

('AVID01000008', 62827, 63268)

62828 .... AATGTCGAATTTATTTAAATAAACTTAATAAAAAAAGTAAAATGACCGT s:0 i:0 d:0

62900 .... AATAGATTTTTCAAAAAAACTAAATGACCGT s:0 i:0 d:0

62953 ....CT................ s:2 i:0 d:0

ATATTCTCCGTACTTAACAAAA s:2 i:0 d:0

('AVGY01000038', 5248, 5689)

5251 .. .. GTCGAATTTATTTAAATAAACTTAATAAAAAAAGTAAAATGACCGTAT s:0 i:0 d:0

5323 .. .. AGATTTTTCAAAAAAACTAAATGACCGT s:0 i:0 d:0

5374 ..A...T.................. s:0 i:2 d:0

AT TCT CCGTACTTAACAAAAAAT s:0 i:2 d:0

('AVGY01000038', 5248, 5689)

5248 .... ATGTCGAATTTATTTAAATAAACTTAATAAAAAAAGTAAAATGACCG s:0 i:0 d:0

5320 .... ATAGATTTTTCAAAAAAACTAAATGACCG s:0 i:0 d:0

5373 .....CT................ s:2 i:0 d:0

TATATTCTCCGTACTTAACAAAAA s:2 i:0 d:0

('AVHG01000011', 5132, 5573)

5135 .. .. GTCGAATTTATTTAAATAAACTTAATAAAAAAAGTAAAATGACCGTAT s:0 i:0 d:0

5207 .. .. AGATTTTTCAAAAAAACTAAATGACCGT s:0 i:0 d:0

5258 ..A...T.................. s:0 i:2 d:0

AT TCT CCGTACTTAACAAAAAAT s:0 i:2 d:0

('AVHG01000011', 5132, 5573)

5132 .... ATGTCGAATTTATTTAAATAAACTTAATAAAAAAAGTAAAATGACCG s:0 i:0 d:0

5204 .... ATAGATTTTTCAAAAAAACTAAATGACCG s:0 i:0 d:0

5257 .....CT................ s:2 i:0 d:0

TATATTCTCCGTACTTAACAAAAA s:2 i:0 d:0

('AVHH01000024', 5509, 5950)

5512 .. .. GTCGAATTTATTTTAAATAAACTTAATAAAAAAAGTAAAATGACCGTAT s:0 i:0 d:0

5584 .. .. AGATTTTTCAAAAAAACTAAATGACCGT s:0 i:0 d:0

5635 ..A...T.................. s:0 i:2 d:0

AT TCT CCGTACTTAACAAAAAAT s:0 i:2 d:0

('AVHH01000024', 5509, 5950)

5509 .... ATGTCGAATTTATTTTAAATAAACTTAATAAAAAAAGTAAAATGACCG s:0 i:0 d:0

5581 .... ATAGATTTTTCAAAAAAACTAAATGACCG s:0 i:0 d:0

5634 .....CT................ s:2 i:0 d:0

TATATTCTCCGTACTTAACAAAAA s:2 i:0 d:0

('AVKM01000015', 5782, 6223)

5784 ... ... ATGTCGAATTTATTTTAAATAAACTTAATAAAAAAAGTAAAATGACCGTA s:0 i:0 d:0

5856 ... ... ATAGATTTTTCAAAAAAACTAAATGACCG s:0 i:0 d:0

5907 ...A...T................ s:0 i:2 d:0

TAT TCT CCGTACTTAACAAAAA s:0 i:2 d:0

('AVKM01000015', 5782, 6223)

5783 .... AATGTCGAATTTATTTTAAATAAACTTAATAAAAAAAGTAAAATGACCGT s:0 i:0 d:0

5855 .... AATAGATTTTTCAAAAAAACTAAATGACCGT s:0 i:0 d:0

5908 ....CT............... s:2 i:0 d:0

ATATTCTCCGTACTTAACAAAA s:2 i:0 d:0

('AVKN01000094', 4553, 4994)

```
4555   ... ... ...............  ATGTCGAATTTATTTTAAATAAACTTAATAAAAAAAGTAAAATGACCGTA      s:0 i:0 d:0

4627   ... ... ...............  ATAGATTTTTCAAAAAAACTAAATGACCG                          s:0 i:0 d:0

4678   ...A...T...............                                                         s:0 i:2 d:0
_____________________________________________________________________________________________________

       TAT TCT CCGTACTTAACAAAAA                                                        s:0 i:2 d:0
```

('AVKN01000094', 4553, 4994)

```
4554   .....................  AATGTCGAATTTATTTTAAATAAACTTAATAAAAAAAGTAAAATGACCGT      s:0 i:0 d:0

4626   .....................  AATAGATTTTTCAAAAAAACTAAATGACCGT                        s:0 i:0 d:0

4679   ....CT...............                                                         s:2 i:0 d:0
_____________________________________________________________________________________________________

       ATATTCTCCGTACTTAACAAAA                                                        s:2 i:0 d:0
```

('AVLM01000069', 5244, 5685)

```
5246   ... ... ...............  ATGTCGAATTTATTTTAAATAAACTTAATAAAAAAAGTAAAATGACCGTA      s:0 i:0 d:0

5318   ... ... ...............  ATAGATTTTTCAAAAAAACTAAATGACCG                          s:0 i:0 d:0

5369   ...A...T...............                                                         s:0 i:2 d:0
_____________________________________________________________________________________________________

       TAT TCT CCGTACTTAACAAAAA                                                        s:0 i:2 d:0
```

('AVLM01000069', 5244, 5685)

```
5245   .....................  AATGTCGAATTTATTTTAAATAAACTTAATAAAAAAAGTAAAATGACCGT      s:0 i:0 d:0

5317   .....................  AATAGATTTTTCAAAAAAACTAAATGACCGT                        s:0 i:0 d:0

5370   ....CT...............                                                         s:2 i:0 d:0
_____________________________________________________________________________________________________

       ATATTCTCCGTACTTAACAAAA                                                        s:2 i:0 d:0
```

('AROD01001420', 23, 369)

```
19     .................A.........  TAACGAACTGAATAAAATGTCAGAAAGTGACG                     s:1 i:0 d:0

80     ...C........................  GCAGCTTAGCGACGAAATTAAAACCGAACTCAC                  s:1 i:0 d:0

142    ............................  TCTTTATCGTCAATGCGAAATTTTCCGCGACG                   s:0 i:0 d:0

203    ............................  TCTTTATCGTCAATGCGAAATTTTCCGCGACG                   s:0 i:0 d:0

264    ............................  TCTTTATCGTCAATGCGATCATGTCAAACGCCATCAGCGTTCCGGCAT   s:0 i:0 d:0

341    ..............A............  AATCGAGCCCCGTCCAGAAATGA                              s:1 i:0 d:0

393    .................A.........                                                       s:1 i:0 d:0
```

____

CGGTTTATCCCCGCTGGCGCGGGGAACAC s:4 i:0 d:0

('AROD01001420', 23, 369)

19 ....A........... AACGAACTGAATAAAATGTCAGAAAGTGACG s:1 i:0 d:0

80 ...C.........................G CAGCTTAGCGACGAAATTAAAACCGAACTCAC s:2 i:0 d:0

142 .... CTTTATCGTCAATGCGAAATTTTCCGCGACG s:0 i:0 d:0

203 .... CTTTATCGTCAATGCGAAATTTTCCGCGACG s:0 i:0 d:0

264 .... CTTTATCGTCAATGCGATCATGTCAAACGCCATCAGCGTTCCGGCAT s:0 i:0 d:0

341 ....A.............A ATCGAGCCCCGTCCAGAAATGA s:2 i:0 d:0

393 ....A........... s:1 i:0 d:0

____

CGGTTTATCCCCGCTGGCGCGGGGAACACT s:6 i:0 d:0

('AROD01001420', 23, 369)

147 .... TGCGAAATTTTCCGCGACGCGGTT s:0 i:0 d:0

208 .... TGCGAAATTTTCCGCGACGCGGTT s:0 i:0 d:0

269 .... s:0 i:0 d:0

____

TATCCCCGCTGGCGCGGGGAACACTCTTTATCGTCAA s:0 i:0 d:0

('LAGH01000043', 29995, 30852)

30000 .... TAGTAACGCTCTTTTTGCAGCAGAAATGAGTTTTAAATTTAAA s:0 i:0 d:0

30066 ....T............... AAAGAATATAAATATTGAACATTATGAAATCTATGCATTTGAA s:1 i:0 d:0

30132 ....G........T s:2 i:0 d:0

____

TACATCTCATGTTATTGTTCAAC s:3 i:0 d:0

('APUE01000001', 33513, 34505)

34339 .... TCCAGAACGTAAAATCTAAAGAAACCGTGTTGTAACGGCAGACCGAT s:0 i:0 d:0

34411 ....T......... TCTAGACCTTGGGATAACGGCAATATTCAAAGTTTATAAAAGA s:1 i:0 d:0

34479 C....T................... s:2 i:0 d:0

____

GCCGTCATTCCCGCGCAGGCGGGAA s:3 i:0 d:0

('APUE01000001', 33513, 34505)

34348 ....C...A CGTAAAATCTAAAGAAACCGTGTTGTAACGGCAGACCGATGCCGTCATT s:2 i:0 d:0

34420 ......T............... CTTGGGATAACGGCAATATTCAAAGTTTATAAAAGACCCGTTATT s:1 i:0 d:0

34488 .... s:0 i:0 d:0

CCCGCGCAGGCGGGAATCTAGAC s:3 i:0 d:0

('APTW01000016', 4487, 5179)

4487 T...........C-CGC........ CCGACTTGTTTGGTTTCGGTTATTTTTCGTTTCGTAACTTTTGAG s:4 i:1 d:1

4557 ....T... CCGGCTTGTTCGGTTTCGGTTCTTTTTCTCGTTTCGGGTGATTTCTAAA s:1 i:0 d:0

4631 .... s:0 i:0 d:0

CCGTCATTCCCGCGCA GGCGGGAAT s:5 i:1 d:1

('APUT01000031', 4655, 5348)

4656 -...T........C-CGC........ CCGACTTGTCTGGTTTCGGTTATTTTTCGTTTCGTAACTTTTGAG s:4 i:1 d:2

4725 ....T... CCGGCTTGTTCGGTTTCGGTTCTTTTTCTCGTTTCGGGTGATTTCTAAA s:1 i:0 d:0

4799 .... s:0 i:0 d:0

CCGTCATTCCCGCGC AGGCGGGAAT s:5 i:1 d:2

('ALXU01000021', 79838, 80433)

80262 .... CTGGTTCGTTCGGTTTCGCTTGTTTTAAGTTTCGGGTAACTTCCACT s:0 i:0 d:0

80334 .... CCAGTGCGTTGAGCTTCAGCTATTTAGAATAAATTTTGAAACTCTAATCG s:0 i:0 d:0

80409 -..........A..A.AG-T...... s:4 i:1 d:2

TCGTCATTCCCGCGCAG GCGGGAAT s:4 i:1 d:2

('AEPI01000051', 2481, 3148)

2977 ....A............. TTCATAAGTTTCCCGAAACACCCGAAAAAACCGAAACCGAACGGACTA s:1 i:0 d:0

3051 .... TTCATAAGTTTCCCGAAAAAAACCGAAAAACCGAACCCGAACGGACCG s:0 i:0 d:0

3124 ....T........... s:1 i:0 d:0

GATTCCCGCCTGCGCGGGAATGACGA s:2 i:0 d:0

('APSU01000003', 21951, 23234)

23068   .---G..TT........................... ACCTTGGGATAACAGCAATATTCAAAGTTTA   s:3 i:0 d:3

23133   .... ACCTTGGGATAACAGCAATATTCAAAGGTTA   s:0 i:0 d:0

23201   ....C..   s:1 i:0 d:0

TAAAAGACCCGTCATTCCCGCGCAGGCGGGAATCTAG   s:4 i:0 d:3

('AVNM01000096', 22457, 23103)

22931   .........T.T..T.........--G CGATTAGAGTTTCAAAATTTATTCTAAATAGCTGAAACTCAACGCACTG   s:4 i:0 d:2

23005   .... GTGGAAGTTACCCGAAACTTAAAACAAGCGAAACCGAACGAACCA   s:0 i:0 d:0

23077   ....   s:0 i:0 d:0

GATTCCCGCCTGCGCGGGAATGACGAA   s:4 i:0 d:2

('AVNM01000096', 22457, 23103)

22934   ......T.T..T.........-G CGATTAGAGTTTCAAAATTTATTCTAAATAGCTGAAACTCAACGCACTGGAT   s:4 i:0 d:1

23008   .... AGTGGAAGTTACCCGAAACTTAAAACAAGCGAAACCGAACGAACCAGAT   s:0 i:0 d:0

23080   ....   s:0 i:0 d:0

TCCCGCCTGCGCGGGAATGACGA   s:4 i:0 d:1

('JMCO01000014', 49434, 50376)

50207   .... TTTAGAAATCACCCGAAACGAGAAAAAGAACCGAAACCGAACAAGCCG   s:0 i:0 d:0

50281   ....A................. CTCAAAAGTTACGAAACGAAAAATAACCGAAACCAGACAAGTCG   s:1 i:0 d:0

50351   .........GC.G...........-.   s:3 i:0 d:1

GATTCCCGCCTGCGCGGGAATGACGG   s:4 i:0 d:1

('ANRI01000017', 124980, 125858)

125594   .T..-A...G.............
AAGTGGGAATCCAGTTTTTTGAGTTTCAGTCATTCCCGATAAATTGCCTTAGCATTGAATGTCTAGATTCTCGCCTGCGCGGGAATGACGAATCCATCCATACGGAAA
s:2 i:1 d:1

125725   . ... A
s:0 i:0 d:0

125749   . ...
s:0 i:0 d:0

C CTGCATCCCGTCATTCCCACGA
s:2 i:1 d:1

('ANRC01000013', 79978, 80856)

80592   .T..-A...G.............
AAGTGGGAATCCAGTTTTTTGAGTTTCAGTCATTCCCGATAAATTGCCTTAGCATTGAATGTCTAGATTCTCGCCTGCGCGGGAATGACGAATCCATCCATACGGAAA
s:2 i:1 d:1

80723   . ... A
s:0 i:0 d:0

80747   . ...
s:0 i:0 d:0

C CTGCATCCCGTCATTCCCACGA
s:2 i:1 d:1

('APUI01000045', 16120, 17603)

16122   .... ATCCGGCTTGTTCGGTTTCGGTTTTTTTTGAGGTTTCGGGCAACTTCTAAACC   s:0 i:0 d:0

16196   .... ATCTAGACCTTAGAACAACAGCAATATTCAAAGATTATAAAAGACCC   s:0 i:0 d:0

16264   ....T........   s:1 i:0 d:0

GTCATTCCCGCGCAGGCGGGA   s:1 i:0 d:0

('ALXM01000022', 174578, 176209)

174576   .... CAGAACGTAAAATCTAAAGAAACCGTGTTGTAACGGCAGACCGAT   s:0 i:0 d:0

174648   ....T.......... TAGACCTTGGGATAACGGCAATATTCAAAGTTTATAAAAGA   s:1 i:0 d:0

174716   C....T....................   s:2 i:0 d:0

GCCGTCATTCCCGCGCAGGCGGGAATC   s:3 i:0 d:0

('ALXM01000022', 174578, 176209)

174579   .... TCCAGAACGTAAAATCTAAAGAAACCGTGTTGTAACGGCAGACCGATGCC   s:0 i:0 d:0

174651   ....T......... TCTAGACCTTGGGATAACGGCAATATTCAAAGTTTATAAAAGACCC   s:1 i:0 d:0

174719   ..T..................   s:1 i:0 d:0

GTCATTCCCGCGCAGGCGGGAA   s:2 i:0 d:0

('JMDE01000014', 63164, 64108)

64075   .-C..TC.G..............
AAGTGGGAATCCAGCTTTTTGAGTTTCAGTCATTTCCGATAAATTGCCTTAGCATTGAATGTCTAGATTCTCGCCTGCGCGGGAATGACGAATCCATCCATACGGAAA
s:3 i:1 d:1

64206   .... A
s:0 i:0 d:0

64230   ....T ....
s:1 i:0 d:0

CCTGC ATCCCGTCATTCCCACGA
s:4 i:1 d:1

('ALXQ01000026', 180262, 182677)

182507   .... CCGGCTTGTTCGGTTTCGGTTTTGGTTTTTTTTGAGGTTTCGGGCAACTTCTAAACCG   s:0 i:0 d:0

182587   .... CTAGACCTTAGAACAACAGCAATATTCAAAGATTATAAAAGACCTG   s:0 i:0 d:0

182655   ....   s:0 i:0 d:0

TCATTCCCGCGCAGGCGGGAAT   s:0 i:0 d:0

('ALXQ01000026', 180262, 182677)

180264   ....A.... CAGACCTTGGGATAACGGCAATATTCAAAGTTTATAAAAGACCC   s:1 i:0 d:0

180332   ....T........... CAGACCTTGGGATAACAGCAATATTCAAAGTTTATAAAAGACCT   s:1 i:0 d:0

180400   ....   s:0 i:0 d:0

GTCATTCCCGCGCAGGCGGGAATC   s:2 i:0 d:0
