# Supplementary material for: CRISPRidentify: identification of CRISPR arrays using machine learning approach
Source: Nucleic Acids Res. 2020 Dec 8;49(4):e20. doi: 10.1093/nar/gkaa1158 (PMC7913763; doi:10.1093/nar/gkaa1158)
Supplement: gkaa1158_Supplemental_Files [file gkaa1158_supplemental_files.zip › CRISPRidentify_Suppl-4.pdf]

CRISPRidentify: Identification of CRISPR arrays using machine learning approach – Supplementary Material 4

Alexander Mitrofanov, Omer S Alkhnbashi, Sergey Shmakov,
Kira M. Makarova, Eugene V. Koonin and Rolf Backofen

Cases with identical spacers

Two identical spacers were found in:

AJVG01000014-77908-79192
CM001166-78729-79830
FR900184-1950-3289
FR900184-3194-4661
KK322870-2781998-2783565
AUDD01000002-205924-207294
CATS01000007-126624-127167
CART01000001-272075-272652
KK328754-3127346-3128121
JRXC01000092-0-395
KK328428-3121054-3121470
AOYR01000111-34008-36051
FR889647-5-4365
BBCB01000168-35-1259
AZEM01000031-1055-1814
CP000967-4539665-4544623
KI912609-2853473-2856670
KI912609-459415-461924
JH109152-847713-850548
KK354274-1476165-1477593
CP001390-3765797-3767699
CP002164-2460382-2461806
CP002164-2449153-2450643
LATT01000253-11039-14957
CP012590-2277581-2278195
CP012590-667519-670661
CP003355-1647331-1647898
KK328987-3145479-3146825
CP002293-1450221-1453456
KB890619-138490-140468
LHKN01000004-0-1080
CP006694-727418-727996
AKWJ02000017-259432-260132
ALMP01000046-94578-97649
JYIJ01000009-108-2140
CP002340-639511-641921
CP002340-1363056-1364415
FO203503-1120958-1125463
KK333680-58-757
KK356627-3113477-3113893
CP009575-2761784-2762873
JYUQ01000079-0-1093
KE386491-1313607-1315990
JH378833-425-3639
JAAC01000184-6089-9398
CP013828-979302-984692
AETC01000013-247848-249342
KK355761-3156444-3157861

LJFA01001316-9-709
LK053000-2169316-2173290
KK322146-3138698-3140355
KB894380-47671-53062
JFJL01000073-1-366
HG796200-122756-124969
JYSZ01000016-0-1163
JQHE01000012-0-2223
KB906648-9962-10296
FR902801-0-656
AQFC01000045-68084-68660
LOEW01000044-747-7244
CCIL01000001-223893-227763
AUZI01000011-4923-6352
CAIQ01000001-33-1632
CP003557-2661871-2663291
JQRV01000033-20179-21061
CYUO01000005-726357-726989
LDLY01000106-0-532
CP001743-3049067-3053623
AUBP01000007-172428-173377
LAJC01000004-89339-90891
CM001486-1149489-1152925
ACZO01000007-751646-752928
CP009526-4403632-4404900
LJDX01001333-9-404
JYVF01000092-0-302
JPOJ01000001-3230638-3242075
JPOJ01000001-3185360-3190488
GL882862-0-728
LJEP01001097-8-694
AROB01003195-2-306
APAI01000798-0-880
ALQU01000054-45025-45727
CP007756-1329033-1329728
KE159726-262958-263495
KE701413-1153947-1154830
CP002913-1030341-1034394
JQHL01000079-4-545
KK036928-192864-193625
JOMK01000001-2409124-2412331
CCHH01000003-109351-113280
AUFU01000028-4770-5497
JRNH01000014-102346-104755
AOXD01000072-16408-17109
JZWV01001387-28-342
JMFG01000047-53-1013
KE386828-133720-135094
GG698602-544398-545267
CARZ01000046-110489-110923
KI911915-2611672-2614448
KK354345-3121486-3122619
CP005998-1360007-1361176
CP000804-3447454-3453071

JQRP01000017-47839-48901
CP003510-863552-866560
JVRZ01000045-40683-42737
HE997184-328401-331863
CP000099-1660241-1661509
AASE01000005-50456-57031
JQMN01000001-32506-35135
KE124774-2608662-2612526
BCAE01000004-12695-19184
KK327974-2792350-2793855
JOAW01001059-1-1042
BBZR01000081-0-725
KE702063-1690931-1691857
AZEM01000104-18901-20944
KK211068-81853-83801
LDNU01000324-14657-16218
KE699566-1990316-1991016
LODR01000031-48019-49789
CZJZ01000007-643219-651590
CP001189-460232-461968
ANCX01000004-43960-45250
LGIP01000023-117746-123411
GG667604-126678-128274
CP000539-499829-503883
KK356536-59-810
LLOZ01000012-279909-281585
ABFF01000007-121216-123686
CP001358-1083778-1085579
ABFH02000001-2106929-2110315
LHHR01000026-0-542
BBBM01000005-15-10929
AOMF01000015-63871-68671
CP013068-4415672-4418258
CP001229-1493361-1494643
AJXS01000272-0-417
KK306903-33626-34909
LN515531-79730-90367
LJDS01000026-142576-143276
KE384103-49734-50844
KE384103-51927-53156
AJXS01000386-18947-21620
CP002690-1769727-1776837
JUJN01000002-181504-182251
AZLV01000242-0-2330
GG667520-54476-59079
LMVE01000001-910-3747
BAOQ01000039-27-1176
CP003179-3258231-3259962
AWVZ01000001-120765-121593
ANAE01000127-82033-82977
KK327704-3107016-3108152
KB899916-52926-60031
JYFD01000220-18796-19584
CP002049-2504069-2507589

KE952480-20595-22453
KK338849-930984-931314
JQDZ01000022-168-3616
AQFF01000020-169915-170919
AFLY02000062-6789-7974
LOBL01000113-223440-224708
CP003418-1381766-1387646
CAKP01000114-2438-7058
AE008692-113783-114170
CP014024-1541212-1542142
AUKN01000029-12993-13813
KK328509-1113947-1114502
KK328509-1117525-1119541
APAL01000098-28909-29611
GL872339-1034075-1036094
AAEW02000018-9057-15613
KK328084-328050-329544
KK328084-323131-324190
AMSY01000047-9-859
JPOG01000001-2844761-2848455
KK213790-921266-922168
KB892328-9291-10428
KE993487-0-716
KB905817-329170-329932
LGDH01000186-40389-41396
CP002792-1629400-1633832
LJZS01000005-146890-149198
KK338875-476343-477781
AOYR01000044-1-426
KB894353-59746-69498
KE701449-159267-159479
JH932293-2435-4078
APAO01000037-27977-28983
JZWY01001118-3375-4136
JYRS01000066-1-426
ALEE01000725-1494-3503
CP012506-3144696-3144891
JONO01000007-81999-82806
JONO01000007-71550-72298
JQRD01000440-359-965
KB896085-123052-123933
AESI01000002-248111-249605
ASAI01000009-70555-74247
KB373044-21903-22133
AYKD01000008-2268-2600
JENW01000154-453-2203
ALJL01000027-43787-48087
AMSH01000057-21176-21871
ANQP01000018-8-704
LN734822-888736-897241
AUCQ01000060-6-1394
CP009261-511711-515040
CP003553-10996-13699
AQOF01000125-32-976

JJPD01000191-5155-9065
KK340221-315327-316540
KK340221-314163-315224
CCUK01000001-1658729-1659389
LMXM01000001-656052-660161
JQZV01000009-108895-119011
CP001100-2813212-2819595
JAGE01000001-3119941-3123405
KK328471-779758-781170
KI912586-23575-25411
JALQ01000133-5-375
LSZQ01000039-0-356
AZXG01000025-103414-104337
AZAX01000015-47900-49470
KK321548-3145746-3148211
CP011386-1644373-1647001
KI913032-787892-788324
LFSL01000148-6253-14562
AP014507-1130216-1131908
AP014507-194015-199098
CCEU01000003-104476-107803
CM002370-1308214-1309966
CZJP01000175-8115-10087
GL882852-46689-49505
JYQF01000104-5-537
CAFD01000053-0-381
CP000771-1019472-1031163
CP001808-119723-121570
HF997015-64527-66614
LECC01000001-470380-470909
AFHI01000014-50586-60456
CP000702-1107559-1110144
AGHE01000001-9571-11795
JYIJ01000019-1199312-1201049
JYIJ01000019-79875-82402
KK311834-135466-136602
KK336757-12-1533
CTBD01000049-46420-48069
JJNX02000069-8836-11650
JYWN01000127-0-475
LDUO01000074-0-296
KB661998-139403-141442
DF236949-2985225-2985929
KB235958-3974658-3977066
KI914527-2805246-2806715
CP002422-1924164-1925322
CCUB01000001-160138-160764
JH719942-3813414-3834634
JH719942-82224-102179
ACJN02000002-1140479-1143096
CP003508-910568-912980
AXLL01000002-65785-69244
LIOE01000139-0-606
AESM01000017-107737-109231

JHVW01000030-11897-13019
KK327770-0-775
CATA01000001-10973-11518
KK342394-3120060-3120759
JYZD01000079-0-302
APHG01000020-9-320
CP002341-819368-823300
JFHZ01000026-8274-10300
AQVB01000002-149765-154656
CP001251-615152-617249
CP001251-470869-474362
JVME01000026-55995-57162
KK339288-3097471-3099042
JAPY01000022-13320-14488
KK356400-3121272-3122613
JDTZ01000007-201-2548
KK331696-0-1663
CP006977-1878951-1883236
JJPZ01000041-8251-13345
AFNV02000025-66925-67298
KI542675-166613-167670
KK317395-59-751
JIDQ01000190-7-843
AIEA01000110-0-1013
JHUV01000008-48896-50126
LBNA01000044-75622-76970
JJUG01000034-38946-39999
KB896473-110001-112594
LGZA01000004-55591-58267
AXLL01000003-41-5829
CARY01000006-108280-108981
LDAI01000018-409563-410523
LCSP01000238-0-657
AEPN01000047-142-666
AFYY01000012-270368-270764
HE576794-2190079-2193775
HE576794-1758456-1761039
KK322135-1414-3357
GG698599-158645-164749
JATP01000030-8-1627
JYVY01000092-10-570
AEZE01000002-150032-150520
CM001225-3080795-3082587
AHOJ02000018-54017-54903
JVIC01000082-81836-85099
ANAZ01000027-84235-85851
GG657556-438023-438583
AWUR01000038-96042-98563
JJPJ01000080-17939-24431
KK328023-588173-588714
CP002544-1524049-1528710
FO704551-787641-788829
AOJP01000010-90508-91937
CZVL01000033-6-3363

FR884308-3599-3960
LJDL01000017-25635-26335
BBYA01000015-22303-28620
CXWS01000037-40641-41707
CP003686-1419392-1420640
KK339170-254992-255825
KK339347-315535-316154
KQ961744-2-1646
KK353392-2805922-2806542
JZIA01000003-3294437-3296655
JOAU01000118-0-301
AE006470-1052107-1055064
JNMA01000062-1360-2608
ALPP01000014-108253-108832
KB733140-1741636-1741970
GL878494-7284-11347
AZBN01000083-8-315
KI912183-3050216-3052256
JFKK01000001-29866-31619
JVBN01000025-0-1126
CM001488-3126730-3133590
CM001488-2748848-2750111
AM889285-63000-64899
AOZC01000054-107060-108373
KK339361-486669-488458
AETO01000018-248111-249605
CP003685-865557-868059
CP010306-1550135-1550436
LFDX01000003-16729-17393
ALWO02000027-77753-79815
KK353358-3136156-3137007
KK353358-3129256-3130405
KK318706-3122149-3122773
KK328957-3125575-3127823
AQUY01000002-498383-504468
CP003154-480144-482860
CP003154-1161624-1162811
JJPW01000078-32634-39130
CCTT01000001-2240539-2241118
AESZ01000014-107741-109236
ALUB01000017-267885-268911
KK317440-48094-48713
AFSL01000029-52-457
CP002043-318378-319855
KK327823-1352355-1353643
KK338960-153009-154794
KK338960-140774-141691
ATMD01000004-415154-416116
CP001140-752203-757947
LFGQ01000002-92534-93171
CP004120-745577-750495
AXWU01000012-132272-132984
KB904821-2762455-2764470
KB904821-907480-908615

JQQY01000067-27538-27934
BBBM01000086-27-1803
BAER01000105-3-907
LILC01000006-87947-89035
LM995703-571791-572487
JYVO01000108-0-607
LJFB01001237-4-1696
AERG01000050-31-1423
JZIB01000003-3323261-3325546
LHER01000063-0-305
AFQS01000011-4759-6778
AP009510-331297-338700
KQ040465-59489-69616
KB899041-3-1204
BAHT02000295-5-1129
KB892158-388941-391959
CP001146-46313-50337
LCXT01000004-267896-268790
AYYI01000101-0-529
AGBG01000102-2-497
KK355407-7197-8192
KK355407-22-1167
AYCZ01000087-9464-11565
AOXL01000001-22706-25053
KK319879-0-476
LDNT01000147-217-4574
AVGZ01000019-118227-119312
CP003415-454455-455277
CP003415-451273-452586
LGFZ01000033-0-8377
JQNR01000004-238199-239203
JJPY01000040-6101-15089
KI519153-3162142-3162828
JYYK01000160-0-665
FO082060-1516464-1519583
APAP01000092-0-946
KK356651-2779757-2781323
CCUZ01000001-752065-752644
KB947319-167304-168000
LLVU01000062-138508-142380
KK317310-59-892
CDRZ01000167-11-4176
ALUC01000029-276994-278020
CP007029-2977325-2982937
AMSH01000007-23207-23713
KK327821-1767897-1769239
JYIA01000132-52-540
LOBK01000182-223724-224992
BAUO01000011-33-3735
ANDW01000022-239072-240955
CP002425-725208-732590
AFCE01000231-61-1438
LEID01000069-0-1073
CP003191-2508337-2513170

AUBL01000065-15485-16989
CARV01000006-126457-126730
BAFM01000051-1-453
HF997013-28292-28591
HF997013-55443-55882
HF997013-56263-57769
CP003732-2204610-2211549
CP007574-8371391-8373189
JQLK01000005-116071-116981
ATOL01000049-4-426
CCVV01000001-2004655-2005234
LN874944-308563-318858
AHMT02000065-6833-7777
JRNG01000036-32-1767
JYIK01000827-3085-5117
KK328979-2786318-2787520
DS996351-28273-29888
CP006245-1419482-1420914
CP001037-7769880-7771303
JXLL01000004-62-1509
JYUQ01000073-0-462
JYWJ01000068-0-668
KB898999-19767-21444
JUJF01000011-136761-138652
JOMH01000001-3169753-3177285
GL636934-1327771-1333288
AQFD01000036-175016-175592
CP002416-976886-982276
KB906895-96-918
KB732249-206974-207543
CP003046-989381-991847
LEIQ01000086-0-298
JJPV01000090-38479-44975
JXIJ01000153-163538-164715
JJPH01000059-5162-12770
JYVI01000050-0-830
LEFQ01000287-0-597
KK357663-3130141-3130624
AIIJ01001025-0-668
KK357282-482111-482509
AYZD01000025-2293-3911
CP001696-717617-718511
LJEP01000972-6-1114
KK335946-135520-136149
JGZP01000006-18848-22083
FP565176-3423445-3425663
BAZZ01000017-43431-44907
CASR01000001-108140-109329
JFZJ01000209-33-2319
AHHV01000031-107876-109370
JTDM01000014-128143-130363
KK321435-3138061-3139274
AOLF01000001-222209-223586
GG697147-3630-5377

CP006931-3042756-3043982
CAJD01000011-100651-102878
KK211229-229108-231056
ATOI01000124-4-307
JVBN01000026-0-1258
JJPQ01000045-14208-19302
JGYD01000004-0-432
KK355887-1122891-1124750
KK328879-2788998-2790572
KK338907-803868-804765
JSWT01000001-2646621-2648115
AAJT02000034-22421-23590
CASV01000001-0-1002
BBKT01000023-18026-23440
AAJQ01000007-41957-43114
JRRF01000004-160856-164785
KK355900-3060406-3061249
CP009961-551076-559846
KK338572-59-1409
AVAF01000165-4980-5723
JPRQ01000032-157217-161288
CZLC01000001-643219-651590
AUCQ01000059-7-1398
AUIT01000013-34315-35522
AOJE01000010-93049-98267
JUDP01000149-0-362
JNCX01000137-280619-281071
JJMM01000010-488888-495857
GG658170-611801-614648
CP009512-901786-906213
KB822674-1266276-1270148
CR931997-773365-777054
LGEO01000056-7128-9073
LBKU01000008-288776-289472
APKF01000451-3-332
CP001931-246541-250335
CP001931-1496454-1500602
LIXQ01002180-2-434
LLLF01000016-47450-48499
LJDU01000003-261-1001
KK322458-3142106-3143822
JDUL01000113-0-3126
CAAD010000050-80851-81776
JFCF01000026-0-2957
AOLG01000031-43977-48656
KQ257675-0-363
KI515715-532914-534712
CP011307-3074030-3077986
JVTL01000031-8682-9930
JYZD01000038-0-424
CARM01000159-0-302
AHON02000067-386-1572
JYBP01000003-465956-470082
KB903721-13996-14426

JMFG01000018-2118-3366
KB733030-699961-700894
KI913112-758075-763796
CCUL01000002-254454-254849
JEZN01000005-203773-210888
CP004079-106133-109594
JYQK01000056-0-424
JQGI01000558-13-495
CP010525-709155-711834
KK354672-48094-49310
AOIJ01000030-135463-143097
LEIY01000082-0-296
AP013045-2414488-2416791
BBCX01000049-0-1134
LEGC01000109-0-412
KB850089-1906763-1907571
CCLJ01000001-160144-160723
AQOJ01000366-0-523
KK339242-819955-820720
KE702425-2337565-2338060
ANBA01000014-315189-317414
AUCQ01000062-0-1321
HF990891-54-1346
KI911557-348741-354936
CM002803-1667722-1669397
AWWC01000006-66396-68255
KK327941-3103975-3105125
BCSU01000028-164-9465
AJTZ01000005-812219-812984
KK338856-1552-1878
KK339319-3122979-3124246
CP007174-2110970-2118191
KK316937-2-839
LSVK01000004-792938-794859
AZWD01000017-6-1556
KK337216-1725-2579
AE008384-4089308-4095187
CCXU01000007-26822-30949
AFWT01000006-66406-72671
KE383937-216934-219302
KK357893-245787-246004
JYQK01000038-22922-25208
KI867150-2377354-2378184
JVTL01000081-35-4884
KI911412-86169-86808
AJGT01000100-22663-28053
CP013118-4170252-4176361
LFCU01000167-13916-14932
KK350916-315512-316356
AHUU01000128-2-424
KB904822-4196-5262
KB904822-15855-16935
LJFB01001243-5-1783
KB733179-1401747-1402691

AOZW01000017-1-519
JLPO01000057-16-986
CP002351-1176110-1178006
KK353342-3126321-3127585
LCXX01000015-267627-268983
CP001349-6088471-6089163
CM001226-3099580-3101372
CP008802-2130997-2134138
AJLK01000074-26119-26654
KI973153-2684871-2686277
JALQ01000131-5-557
JXYA01000161-0-392
KK322805-482539-483237
LFGN01000003-16890-17639
FR902814-0-916
KB946719-1769400-1769833
KK328434-823427-824785
JQID01000006-6930-8595
KE386489-1957112-1958232
KE386489-1962654-1965938
JNWU01000019-87860-90328
LHFA01000028-0-1152
JTCM01000018-2305-2708
CDHJ01000147-8306-11062
KQ961776-3-1042
KL571237-27488-28246
LN909045-212176-212879
LPVQ01000002-88690-90247
KI519499-567306-568381
LJFB01001149-73-773
KI911492-123746-125177
CP001654-836653-840342
CP003969-9167756-9170662
LGCK01000011-21268-27585
JHAB01000007-4351-8737
ATHI01000015-51-3897
JAFB01000058-13939-14637
KK357649-1590827-1591044
CP002772-1531116-1536379
AKWQ02000032-62807-64704
CM001471-2931502-2933051
KK318583-48094-48867
HE974285-34-721
LDXS01000013-4-1901
KK327758-816666-818090
AZVZ01000039-3-886
AUNH01000173-2734-3499
AVAF01000171-36-1739
LM997407-128696-129396
KB907867-13407-14863
CZJP01000497-42-1770
KK322562-481887-483032
AZFL01000014-10901-13308
CP001715-2565675-2575157

LFJO01000002-423098-424059
ALMU01000025-33309-33872
JUTP01000136-0-729
KK354825-1491401-1492166
CP000471-4407394-4416588
AQUY01000008-4-1163
BBRB01000003-180188-182333
LHMY01000060-0-298
CCLZ01000001-753728-754185
AJVN01000017-5010-7544
CP000568-716797-720141
CP000568-2729745-2741081
CP002131-74004-78146
JMZY02000213-11429-15743
JZTM01000011-105503-106997
AEAT01000014-0-1459
APCS01000112-72-1431
LHMY01000062-0-300
AZGJ01000058-9020-9935
FO704550-570114-575028
AEJL02000100-156-4209
JUGU01000001-147526-150989
CCTW01000001-1690143-1690600
JVTN01000053-11275-17344
JQFA01000004-1117811-1118836
JHAC01000002-89948-104979
KB913013-4726406-4728810
CP000575-332545-333483
ALIV01000033-295-1883
AJLF01000002-391438-395269
CP003260-1576110-1580704
KI518814-792073-792942
KK339304-487853-489636
LMVI01000001-764888-765123
LMVI01000001-764558-764793
CARK01000001-289660-290421
JJOY01000033-78939-83792
CP006819-1558840-1559668
APAH01000134-29552-30804
LEKT01000005-92398-95924
CP003198-1604243-1609325
AP014508-340778-345855
AP014508-263332-264346
AP014508-278737-279551
CEEL01000002-135639-142868
AUGH01000011-119232-120750
APYY01000445-75376-76137
KK338399-1744-2893
LKEV01000001-242664-244034
CZPX01000005-542025-543762
AENV01000008-111793-118502
LEFD01000074-0-477
KK328378-1759582-1761125
CP010827-2029846-2033839

BAHR02000110-3403-4499
KK322466-308887-310170
BBND01000027-3662-7954
KK328477-1488825-1490350
JREO01000009-139265-141304
LECD01000001-470295-470866
KK356185-1-1353
JJPT01000126-9373-15881
KK338833-3098022-3099607
KK338833-3099978-3101544
JH594505-19448-20590
ACJX03000001-406172-411451
KB908393-41835-43059
KK350898-3055011-3056282
JEXJ01000423-0-666
KK339316-807575-807977
CP001792-165661-167962
KB851021-51612-52673
CM001475-1790552-1793023
CP006872-2309201-2310570
BBND01000001-59459-69211
KK333245-14-349
KK333245-1818-2548
CCLR01000001-159923-160502
CP009479-1247135-1250876
JJPJ01000024-15924-22913
ASLU01000029-0-3331
BAZR01000011-33-3735
KK097721-118423-119550
JQRE01000288-10474-13373
KL575750-34149-35397
AOHB02000050-16483-17061
CP001804-6642689-6685248
ACXM01000006-414682-415644
CP000141-1926326-1930135
JFBE01000075-20727-21605
KK340196-3139410-3140899
CP005290-1137911-1142191
HE978568-128141-130303
KK355303-1482406-1483615
LIKR01000020-611585-613249
LJDQ01000003-142367-143067
BBFN01000014-25529-32545
KI519264-613552-614557
ATZH01000031-320-2546
LHMY01000039-0-1078
CP003182-1561434-1562501
ADJT01005537-1-1145
AKXB02000110-50741-52771
JZIF01000003-3312159-3314444
AZWH01000024-1840-3766
AMSB01000021-0-363
HF545617-253726-262984
CP002028-1952689-1959253

JYUO01000122-57-1209
LHSK01000001-620921-623331
CP003059-218990-221898
KK328732-2778677-2779889
AUHH01000033-2852-5810
AJTS01000045-27437-27712
CP000891-3981874-3984902
AHHW01000057-120035-121530
BBAQ01000023-27640-28397
ABVG02000001-1913923-1923730
AOLJ01000011-58-3869
FO818640-3904507-3907600
AJWR01000020-38765-40138
JXVO01000025-37-798
AMFN01000002-542145-542538
CP009792-2470667-2473459
JJPV01000052-6122-12822
AVJY01000049-1342-2623
AVHT01000034-3980-5198
JIBI01000056-117-2428
AP012029-1369173-1384073
AP012029-757970-762020
AYYW01000049-0-533
KE136744-1055809-1056613
ALYN01000062-31-658
LHIX01000104-0-1460
KE386857-160288-160911
CP003167-2098626-2103812
AMJB01000210-24293-29123
AYYL01000001-30885-32857
CP010451-375419-380208
HE978586-257084-258210
BANU01000022-54-3871
HE578923-474400-474970
KQ961766-6-745
CP000716-754561-758169
CCTO01000001-2186737-2187316
KK322844-3118508-3119782
JH941057-161389-161776
JDTK01000010-11228-16324
JAAG01000320-33-1184
KB891296-912573-915164
JQDQ01000121-8529-11064
JPEC01000017-24391-27205
ATZI01000028-342-5089
BBXX01000024-12005-20391
CP009528-3468844-3477647
KK328358-1487187-1487583
LFGZ01000004-27691-28056
AP009389-1989144-1996925
AYQB01000018-115386-115946
JQIS01000009-142486-149775
JYUQ01000076-0-484
CP001731-796685-804197

CP000283-1176802-1180005
AUTU01000017-38725-40886
CP002630-274043-277499
ALIY01000071-11208-18010
ALIY01000071-18043-18620
KE136820-146037-146553
CAFD01000120-16439-17200
CARP01000005-126297-126814
BADL01000257-1028-4721
JRPB01000001-984980-985610
KE384577-14170-15458
ALPT02000161-0-1290
APUO01000093-32136-33687
KK325005-3049865-3050708
AFLY02000082-15444-16704
LACO01000001-277963-278591
LACO01000001-488072-490219
CP000477-670838-677982
KI518951-1388799-1389487
KK339269-3104044-3106875
KI911515-8988-10300
JWPU01000102-0-911
KB889965-79155-87181
JXLR01000026-10152-12166
LAGG01000102-32542-49859
JQLF01000006-33127-34559
KK328500-2803716-2804478
CP001825-584090-585218
BBFX01001086-1-1130
GL501401-324857-332730
CP000922-812914-816907
KB895346-43091-44465
KK328603-152-1683
LAXD01000001-389801-392388
LAXD01000001-4555247-4556923
KK356549-216-1380
KB732996-1576223-1577045
KB732996-1549029-1550522
AHOS02000006-225654-226288
JMLW01000004-6-1621
LN869922-2043645-2043945
L77117-377-1923
CBLF010002125-8-375
CU928145-3120000-3120944
GG695972-5676-9643
JJPI01000128-15931-22920
JJPU01000048-6239-12068
JQNI01000004-285908-288779
JQNI01000004-413608-415956
APUZ01000114-16964-18190
FBBJ01000018-0-385
BCQG01000028-28236-29903
JDTP01000003-1-304
KE384251-308673-309249

KL405699-481706-482919
KK332193-0-624
CP010999-1368585-1369413
LHEP01000111-1-730
CCUN01000001-3575050-3575507
JUJC01000001-158314-161033
LDNS01000172-229-4586
LLVV01000005-92646-96252
KQ950180-737826-740781
APUC01000062-737-2423
CVTY01000006-433633-437450
HG916826-842636-846204
CP002638-2510750-2513279
AFLV02000023-66712-67227
BCTC01000028-60227-65006
CAOM01000515-12-3496
LHHW01000040-0-1522
CP000108-2511696-2513118
KK213125-0-449
CP002829-1221571-1225129
KB899641-44570-45236
KK355929-3128216-3129647
KK329541-1-1226
KK321818-2786192-2787337
ATOH01000052-4-485
JYTS01000100-0-647
CAUK01000004-677883-679701
LMFV01000001-247475-249567
JYHN01000025-36175-37479
CCSO01000001-3629176-3629509
CCSO01000001-3612180-3612637
JYZE01000056-0-788
KK327822-3055758-3057325
CP011217-1383837-1384867
LHKT01000137-6-314
KB904783-1531-2653
KQ961735-46967-49617
LK391695-844855-848423
ADDR02000003-36699-38201
ALUW01000002-68108-69926
CARD01000001-272231-272930
LHIW01000011-0-1091
LDYH01000001-470448-471131
CP007264-705939-708770
AHIK01000006-435500-436994
CP001738-420038-421226
KI519507-79325-80080
KI911498-69928-73376
KI911498-68134-69807
JZTT01000003-107930-109363
CP000493-702373-705556
HF997012-39950-41145
KK354720-315997-316765
BBGT01000003-10263-10903

AVMK01000039-113745-115495
KL544017-11487-16075
LFJS01000001-107781-109521
JQMP01000003-935492-945668
FR873482-713942-715692
CCVK01000001-2189682-2190261
LECC01000003-25-599
LOER01000004-145736-148255
KK332729-1725-2593
CP003364-5291567-5297044
CP007289-2961365-2961822
CCLU01000001-159340-159919
JHYD01000010-8299-8664
KK328190-1479885-1480879
AGIB01000093-0-427
DS990445-105131-106685
CCUP01000001-2177225-2177804
LDRH01000191-3-1284
AELS01000027-59176-62114
HE610999-40101-40934
CP001698-461100-462491
KL662191-1217459-1223242
AP009380-1410009-1410839
KB900537-226022-227577
JMQF01000016-7829-9667
BCNT01000001-622958-625810
LN890522-3086165-3088269
LN890518-3290596-3293127
KK316642-3116440-3118155
LFCG01000001-470406-471271
BCQP01000056-36901-38641
KI912105-2873676-2876280
LFCI01000004-4-1040
ANCE01000043-1804-4774
KB892181-62609-63127
KK333696-0-1581
FR896016-19283-20053
LSBP01000014-58873-63722
KK339141-793910-794809
KK353372-2302333-2303032
KK322277-1496016-1497947
ANCQ01000024-243772-244269
JYUJ01000042-0-544
CP003412-2861124-2862408
LLNZ01000023-346733-348165
JMCH01000092-28-1346
CZIN01000716-2839-4162
KL406378-486552-488347
LEFP01000238-0-477
JH590863-468421-470706
BAII02000009-265228-266734
LMJZ01000023-165363-170548
AZLW01000289-5345-5776
AHMH02000142-13151-14186

AVGP01000132-64159-65836
CP001638-358936-361366
CP001638-2059340-2060801
AHHS01000064-122668-124162
KK327798-3052341-3053550
KN549132-1587-2336
JFZT01000065-16236-22497
CCJA01000016-14156-18085
AESA02000083-6125-6746
KB733011-3556721-3557238
KK356564-3109606-3110296
CP003065-2496244-2502078
ANDH01000029-109846-111267
KK316833-3131039-3132245
KK350906-3136510-3138078
KK357157-808495-810142
BAID02000285-35-2381
AE015924-1243691-1244189
LGHJ01000027-15610-23996
AQDQ01000022-543-1471
KK339400-811965-814776
AE013598-888610-892516
AJIP01000043-73429-76897
AP012330-1624513-1626558
LBLA01000023-79515-81096
AOUB01000126-7855-8395
AP006840-721897-723646
LFSA01000044-1-4602
AXSW01000009-115739-117842
KB741839-757725-758091
CP000359-1488141-1489823
KK338837-817372-817847
KE159493-1018960-1019523
LCZR01000035-222607-224028
CP006933-1477405-1484584
KB896608-531024-531603
JJPU01000010-8600-15096
ALTR01000009-52-550
KK339070-3136701-3137913
AYJW01000170-1-364
BAHD01000045-33-2135
AKWM02000060-685-1508
CZJZ01000134-82464-86046
KL661960-26891-29621
AJFI01000064-13268-19582
LACM01000001-142261-145473
LACM01000001-125510-128480
KB892667-6770-9775
KK339075-3103321-3104606
LDZG01000004-101944-102889
AROD01001423-0-432
KK340825-2179701-2180545
JQJB01000013-57512-59128
CM003135-686288-687843

JJPL01000073-20746-25912
KK356933-3118418-3119180
KL544003-51991-53908
JYPM01000067-0-1134
ABXP02000121-11-1422
LSFT01000153-37-1314
JDUS01000003-29-1058
LGYU01000009-329690-335281
APAG01000074-12633-13516
LOBJ01000111-444819-446086
AP010946-3158605-3163849
KL543987-35755-36865
ARRZ01000040-94104-97423
CU468135-934258-935308
KE699459-948204-948654
JYPT01000052-2-1294
CP002735-4895218-4897497
KB905693-433543-434426
AE008691-2326769-2331141
CP001185-326882-329288
CP001185-368609-371819
GL834309-221640-225506
JRMQ01000032-56-728
JVAC01000041-214630-216650
JVEI01000025-0-1034
CP003494-2786007-2788174
LHEV01000058-0-972
KK327774-3053261-3054828
JJPG01000029-5162-12846
AETP01000017-248105-249599
BBYR01000088-6-412
CUWI01000001-3118698-3119463
CP006965-954067-956266
CASD01000054-79652-80476
LBNQ01000013-25395-34245
LCTR01000037-42783-44468
CP009788-2152377-2156250
CP002171-309413-312133
GL732470-17206-19027
JFZT01000041-429-5855
GL945017-2369818-2378638
KK315860-58-385
KB913024-2875754-2876271
ASAI01000042-27587-31279
LMTQ01000479-389-2065
APAP01000112-57-942
CM001857-824845-828533
JJPY01000002-2880-7974
LHOM01000084-0-726
LEGM01000417-0-417
AP012338-768723-772772
FR873693-81115-83218
KK339262-3125190-3125952
JJPN01000139-8923-14017

KE137068-105633-105967
KK334158-16-1635
LIKP01000035-1-487
KQ089472-184091-186255
AP010968-623529-625875
AHMJ02000002-107568-107962
CP001807-1886944-1889769
ANEP01000040-64772-65786
KK339466-808800-809724
LFGR01000003-6-317
KK321540-3120540-3122494
KK351005-3106787-3107777
AGFO01000018-67027-71816
AESL01000016-317449-318943
CP014234-1096544-1101975
BCRO01000012-300076-300532
ANAS01000036-98259-99756
KK320104-36073-37199
JFCO01000075-19900-22308
AAWO01000063-30-1280
KI530681-161019-161902
FR891835-16580-17018
FR891835-127-564
LGSP01000127-11103-11619
LIST01000008-120571-126312
KE386863-25294-26360
CP012952-1163019-1167670
LNDM01000004-110613-112045
KK350942-3053022-3053348
KK327881-484890-486386
CP000950-1821346-1824138
KK321911-3119105-3121118
JXTT01000045-157647-160483
KB822986-4226-6775
AAWO01000062-0-9619
CP002476-144273-145340
CP000612-1768138-1768715
CP009530-2108493-2110352
ALOX01000172-43-2112
KK339493-63-1121
KE387205-12810-14364
KQ033885-1595967-1600816
ABDV01000023-42677-47303
CP002403-1313205-1317474
CM002272-132103-133866
LAYP01000012-33881-34520
KI911510-84878-85763
KE700344-2903523-2904406
JYXG01000055-12-757
CAJE01000012-732086-739595
JUBR01000139-0-849
CAFD01000102-63723-66559
ALSJ01000014-55283-56110
JJPI01000071-38457-44948

JJPK01000046-92791-99428
KK327809-809382-810812
JVFV01000043-76828-78447
JJOV01000033-58295-65951
KK339267-0-1069
FBGG01000023-59126-60152
BBCP01000004-42-3846
CP003066-2643364-2668991
CP011254-4410604-4413153
JWIO01000014-81095-83019
KK355972-1000341-1002124
CP003255-3418361-3420970
CATT01000008-54499-54843
KB849468-798805-799436
ALWJ01000036-32495-33316
CALZ01000079-35296-38253
CM001838-1533984-1535937
ATTH01000001-3022278-3023527
LHPH01000008-72906-77379
GG770540-124622-126755
KK342251-3111473-3112537
CP000686-3781970-3786321
CP000686-2427546-2436730
AEJK02000067-122941-127172
HG003686-733786-735219
AVAH01000196-3912-5740
KK327957-3051677-3052887
CARB01000003-109071-109585
BX470251-2395198-2395826
KE340297-1173203-1175615
AZXC01000036-12940-15164
KB895358-1976-4204
AJFF01000627-12-453
ALQG01000092-35674-36553
JQJI01000021-11394-11752
KK350997-2364826-2366036
CP010309-1585880-1587105
CP002426-365877-373263
CP002830-2680593-2682515
CP002830-2377929-2379047
HE972855-72-1113
KK328007-12-1066
AUFJ01000003-1658-2175
AUFJ01000003-560-1260
KB945052-896298-896598
LN913013-474296-476505
CP008903-814308-815281
CP008903-1638836-1639530
CP001839-1395573-1396938
KK322452-3137286-3139242
KK332752-139741-140421
LQMP01000016-5143-6773
FR900073-56-696
JYQI01000031-19-843

JZJI01000139-573-2148
ACEQ02000077-47-1269
JYYB01000020-0-1194
JYYT01000023-106155-107084
JMLW01000012-23432-26042
AHJO01000001-858005-861905
JMCG01000001-1238173-1240042
KK328950-480305-481795
KE137187-796965-797421
CP000312-1054740-1058680
JYZO01000071-0-850
CATN01000001-108204-109073
KK321513-1783330-1784771
CP000946-1018444-1019386
LIDX01000018-52250-54073
KK321730-3135794-3136865
KK321730-3129385-3131111
ANMT01000025-320850-323069
BAYV01000107-12432-14167
CAFD01000135-524-857
CP003220-3491652-3498191
KK342180-0-411
GL455129-64347-64951
AZTB01000064-3559-4820
AXWW01000017-76675-77312
KE695773-123638-124921
AQOV01000002-150623-151874
JOIP01000080-0-609
KK327838-3056340-3057908
JYUY01000116-0-1867
CP010430-4492627-4501501
JALQ01000115-0-301
CP003178-5987684-5989880
CP002048-1597644-1605566
CDDH01000008-5447-11060
LRDG01000006-153776-156427
AQOS01000003-10972-12467
KK319834-3121620-3123256
AFQU01000001-1890221-1892829
CP003653-536593-537916
JRMY01000018-32-488
CP007443-1592210-1595415
JOKH01000006-211061-213105
LN885086-993769-1002140
CASU01000001-107813-108332
JQQA01000115-1818-3002
JSLV01000048-589710-590959
BCAN01000026-194019-197826
CCTM01000001-159670-160188
JWPU01000196-0-484
AANQ01000001-163125-163755
KL406311-1488419-1489188
CCTU01000001-2173455-2173912
AENV01000002-174767-175426

JALQ01000106-4-436
KI518955-4251830-4253201
KB021579-1760674-1761618
KB898192-66018-67123
JNXJ01000149-0-301
KQ034040-1805825-1809968
CCJC01000020-204898-205841
JJPK01000084-15930-22919
JWHQ01000012-7087-9436
BASD01000005-137246-138076
KK327832-1758589-1759426
JUUD01000016-179623-180517
LROC01000081-17210-18002
KI258974-39951-40317
KK323284-136243-137238
HG934468-2381450-2386414
GG698724-8-899
KK328726-2800959-2803423
KK336431-1638-3683
AMIC01000010-0-418
CP009312-1010204-1011452
KB896022-90669-91310
KE386604-23800-24560
KE386604-34922-35814
JYYQ01000153-2-297
AGIY02000001-795217-797136
LDUO01000040-0-950
LFGM01000002-14-319
LM995447-2129473-2135837
CP002637-2182862-2188966
KI632512-2544119-2549035
KI632512-866486-869199
CP003659-4752370-4758593
KK357348-486165-486933
JDTQ01000002-31-621
LMKS01000021-165322-170536
AYYW01000057-20-2104
CARN01000045-0-362
AQOI01000071-1-921
KK356973-3114082-3114778
CP003273-832965-834392
JH114379-47923-50719
LJDM01000059-142627-143327
LN831256-88351-93782
LKBG01000131-0-1463
KK356567-1137941-1140680
JPDT01002993-423-7298
BCBV01000109-61319-62343
CP003267-66099-70220
CP007684-2642301-2644452
AXAQ01000192-14154-14698
LAZX01000009-126-634
LSZQ01000018-0-417
JNAD01000109-37-554

KK334101-136240-136938
CP001312-1434349-1434909
AEMC01000031-31328-35647
JALQ01000142-3-311
KI783332-47883-49386
KB851182-2974389-2975665
HG313790-65578-67009
CP003056-1096064-1097943
CP000450-2341825-2342637
AJKQ01000017-121081-123162
AHMF02000018-196348-196954
KK357138-58-1479
LHKK01000104-0-591
CP009515-3273502-3275585
CP009515-2705052-2710429
CP009515-2718697-2719924
AKXB02000116-6487-6945
KE701968-3003105-3003622
APXB01000181-78104-78974
CP007530-2646621-2648115
JPGB01000004-575115-578118
LDNZ01000146-21214-23492
LDNZ01000146-9254-13134
KK335142-280-1232
KB732762-3379415-3379992
JYRS01000009-10517-11766
AQOJ01000254-2-531
AHOE02000055-29019-30024
JVRY01000039-215842-217896
BBBH01000054-3039-15041
KK357092-3151896-3152530
LRNF01000003-503252-503891
AETN01000019-338335-339829
CP000249-32160-34505
KK322726-2761853-2763061
LDMP01000082-0-897
JXYA01000089-0-328
KK328030-3160698-3161248
AP011615-4296782-4298530
CP001824-1142246-1144538
CP002330-2663461-2675104
CP002330-2735112-2736593
KK353983-135727-136867
CP001896-756776-762359
KQ969241-762408-763696
JZRE01000065-0-356
LMWI01000002-145740-148269
KK321739-1428974-1430689
AOTQ01000537-54-4646
AE017261-1540317-1545939
KB851022-97896-98978
LOPZ01000198-32-774
JRPD01000003-466290-467182
JH414740-201299-204945

CP000230-989381-991847
HE973582-1098036-1104409
FR775234-106349-109005
FR775234-125512-127616
CP009709-2584185-2585866
KB733426-377767-378815
CXXH01000003-387011-387526
HF996449-307000-308530
AUCQ01000061-7-1339
JPFV01000012-147711-149593
LFRU01000021-409-1484
KK328327-813971-815627
BBZZ01000005-92798-95868
GG694006-215062-218574
CDRZ01000155-9-3044
JJPW01000020-6253-12953
CP012069-1961130-1963790
AGBX01000001-940380-943769
CDON01000007-106519-110933
JSQK01000112-32505-32717
KK106988-493212-494645
KK315117-1415-3134
ALTE01000014-44040-45792
AQOS01000050-18969-20170
KK329669-1930-4242
LHKC01000094-0-433
LOBI01000111-163689-164957
KK322751-3113823-3114887
CP003651-100514-103079
LPXI01000040-49902-54362
KI911335-105471-107034
JJQE01000092-5162-12846
AANJ01000002-164381-164745
CP001097-499775-508012
GL698449-677078-678694
CP003592-2206596-2212373
JVTQ01000373-0-421
KK328372-817624-818968
KB905472-1517959-1518927
DS483542-56156-61019
AFVX01000004-147395-148159
CP000679-60111-62054
KI519076-1987580-1988809
KK339929-13-342
KK342454-1487103-1487873
AHJJ01000199-36-679
JALQ01000135-2-365
AZVL01000051-6-1729
JQSB01000214-14160-15776
GL636070-1240369-1240933
CP009514-3314775-3320263
KI530683-8717-9390
BCRK01000024-39463-40039
ALPT02000173-0-849

BAVR01000029-45420-46186
KK356487-3152919-3153901
LIUQ01000045-47-7235
AKZN01000027-16155-19623
KB899416-45847-46745
KB733194-1702839-1703588
CAUJ01000008-479375-481726
JXBL01000001-1849268-1854541
LHDZ01000071-6-684
BAUJ01000030-46087-47458
LN890520-3238918-3240778
LN890520-3220368-3222411
CP005385-1501132-1505688
JAAH01000112-9736-15806
LN913005-129621-132747
LFSS01000021-409-1484
CP009622-1909887-1911990
CP011642-2104473-2106740
CM002369-639771-641324
LHYW01000051-18109-18791
CP002210-2348662-2362246

Three identical spacers were found in:

FR900184-58-1525
JJLN01000224-185552-187202
LHKC01000075-0-494
CP002792-1529272-1531189
KE699185-371799-372907
KK211352-404554-405057
CT573213-513102-518802
AHJJ01000200-34-548
ATWX01000005-108851-109795
JYZW01000078-13-687
CASR01000001-125673-126462
LCXN01000043-43751-44513
AVKA01000010-429723-430941
CP001901-1021671-1022671
KK353342-3127736-3129007
CP000575-592269-593339
KB893283-2770-4061
CP003022-1595959-1603670
KE705264-394974-396040
CP001798-1192983-1194875
KK338497-1492720-1493801
AUFJ01000006-1-1220
KI517354-255077-256665
KE384526-493045-495087
CUYA01000041-109-2237

Two sequential identical spacers were found in:

JQJE01000020-93403-94138
AZDU01000052-9828-10588
KK338951-3112983-3115068
KK354768-319952-320502
KK354768-317825-319619
LFCC01000125-160732-162392
LMBK01000235-109105-109740
ANDJ01000170-40859-42149
CM001166-85486-86247
KB932483-647820-648384
AUXW01000208-1007-2236
AQCU01000182-74104-74865
JYRL01000011-15198-16415
JGZT01000006-344344-350270
JYTE01000023-94581-95464
CP001941-983166-984132
ABTR02000001-1118553-1120680
CP004353-2250209-2262139
GL945270-13870-14833
CART01000001-290071-290276
AQBR01000085-43675-44314
AZCS01000008-19728-21586
KK328754-3131265-3132687
JJJW01000001-799506-799740
LIML01000002-28018-28230
LIML01000002-27531-28108
JZUU01000038-270643-271832
KK350941-3062708-3064248
KK350941-3064849-3066429
AVSW01000117-66953-68674
KK322832-834733-835504
KK328870-3701-5341
FQ859183-2549335-2550091
KK338900-830120-831397
KK338900-829038-830018
JWQG01000002-449266-450990
CP007775-233888-234844
KK320672-6-1241
CCRD01000106-127690-128021
KK356251-3120260-3121828
KI912609-2864046-2865967
KI912609-2860581-2862577
KK340401-298717-299407
KK340401-302461-302938
AIHB01000062-153311-153706
LHOQ01000007-266874-269751
KK355896-5078-7309
KK355896-2504-3563
KK335314-0-1467
BBGT01000176-36-3733
KB944537-1408757-1409057
AXVO01000015-3-277

CP002609-1001644-1002648
JPFP01000063-188276-189997
CP002164-2464719-2466679
JALQ01000022-18675-22852
ALIA01000673-3043-3681
CP012590-693111-704380
CP012590-865452-866285
CP002031-1485510-1500188
KK213883-787734-787970
CDPW01000001-1661797-1665604
LIOG01000006-88301-89856
AL157959-608412-609504
LCZX01000019-43702-44927
CP002293-1954445-1956204
JYPQ01000006-15796-16374
KK328748-2785504-2786264
BBZD01000056-138162-139979
FR746099-403465-406565
KK333859-0-1333
LFCV01000100-18374-19016
JYUG01000016-165864-167173
KB896874-43164-44838
AOIB01000028-9653-10869
AZXF01000015-41233-42728
CP002045-1912138-1916500
KK339452-3139360-3140072
KK339452-3142406-3144122
LROC01000013-83597-84236
KK321905-3138234-3140026
LHMG01000103-8756-9395
KK336940-130172-132192
CP009575-535150-540395
KE386491-2118005-2138611
AP011529-526195-527564
JYSU01000003-469481-470912
KK316975-2782741-2784379
JFKL01000035-30121-32467
KK353354-2172955-2174166
CWLH01000068-5583-6082
KK317404-286-1123
KK354750-1491156-1492214
KK328013-854643-855783
AUVD01000035-131683-133537
CP003243-1142856-1143548
LK053000-2182090-2185869
KK322146-3135344-3136765
KK322146-3130461-3131297
KK322146-3140507-3141573
KK322146-3131472-3132598
KK316685-766158-767080
KK337499-297-1280
AERV01000005-45086-47189
AQCW01000119-105155-105916
CP006262-3425897-3426415

CP007250-2970250-2970462
CP007250-2969763-2970340
KK341227-3119381-3120436
KK337143-0-1523
CP007543-56271-57341
CAPA01000009-100700-102924
KK331144-1653-3491
KK328525-3133208-3134781
KK339441-3099901-3102064
KK340419-775366-777007
JQHL01000008-5525-6333
APHB01000001-31-425
GG668518-597866-598383
JDTG01000017-83641-87460
KI914461-2799256-2799884
LIQD01000003-813652-814291
LQTW01000001-277108-277566
ADWU01000002-63962-64724
KI535657-2994239-2995342
CP010341-413340-416346
KK322500-2215905-2216977
KK350895-1348441-1349723
CP011396-953773-954047
CP011396-952919-953863
KK322653-836406-837033
CUWO01000001-3156880-3158165
JYUX01000045-5924-9106
JYQP01000030-95642-96708
KK336144-1-2019
APGQ01000097-44613-45830
JPYA01000060-42930-44880
LGZP01000017-96593-96988
LHJQ01000052-0-373
BBBW01000007-46061-47429
CP006717-2949546-2950673
GL870795-117651-117917
CP003282-490482-493390
JPZN01000153-57-3651
CP002629-87672-94203
CP012144-943069-944745
BAFN01000001-351146-359887
CP007262-2971020-2971597
CP007262-2971507-2971719
ALGA01000034-29854-30431
ALGA01000034-30341-30553
AQES01000011-156464-157103
CP007156-450165-454652
JFCB01000051-5418-6423
BX248333-3078417-3080138
APYR01000152-486-1796
JXTK01000038-114660-116381
KK329384-68-973
GG698602-546562-548329
CARZ01000046-108279-110367

CARZ01000046-127268-127846
BBII01000008-74605-75428
BBII01000008-74406-74704
CP001472-895485-895723
CP001472-894164-895591
KK350922-3079169-3080785
KI912411-181089-181853
JWHG01000019-458540-459963
KN046795-1711215-1716133
LN881716-355478-358496
KI542980-4086-4358
JQMQ01000005-5941651-5943873
ANRC01000017-14898-16188
CP001790-439446-439842
JYZX01000005-322584-323589
CP000804-376921-377195
KK335306-59-1637
LIMZ01000003-123138-124692
CUWJ01000001-3129422-3130706
APHC01000067-57103-58321
KK328487-1497810-1499308
KK328487-1487712-1488763
CP003107-4012487-4013653
JOLU01000001-227664-228608
LHGR01000016-51088-52093
CATB01000006-108049-108972
CATB01000006-109196-109780
CWMG01000002-5644-7067
JNMZ01000061-2586-3042
KK327974-2798031-2799534
AKWY02000003-55924-56687
JQEP01000051-149188-150909
JZTO01000005-126937-129936
KK328103-1755748-1756148
AZEM01000104-8773-9109
KK328856-2794146-2795354
CWMD01000039-4130-5553
ALYC02000001-91658-93006
JHKX01000008-120785-121420
CASY01000001-125636-126260
KK340061-3126507-3127983
CM001151-3137472-3137744
LAKD01000050-37156-37507
JYUQ01000028-32181-33064
JYSL01000103-58-1124
JWQY01000008-45499-48498
CP003211-39025-39783
KK328001-1518-2573
GL892076-1995153-1997045
GL892076-1997328-2002155
ASYZ01000178-9081-15120
JZTV01000014-45238-48237
AOXC01000014-16681-18235
CP007248-2969353-2969991

BAZE01000014-3331-4704
ABAO01000021-71741-73356
CCNM01000043-128998-131486
KK322763-2240250-2241597
KK322763-2234705-2235330
KK322763-2238313-2239955
KK332615-129338-130256
JPQQ01000089-10-1565
CP001960-1497556-1497857
KB907624-23535-25307
CP001229-1082775-1083474
JSTF01000019-33935-35763
KK354763-492368-493423
FAYJ01000002-400903-401203
JRZX01000004-106058-107534
AQAD01000047-107898-108660
BCMK01000067-59063-59333
KK354881-3106630-3107912
CAOZ01000010-100352-102578
LOOJ01000007-8854-9493
CP006770-1309635-1310786
KK338319-0-404
LDDS01000017-277305-277639
KK327868-810800-812241
CASE01000001-290347-291048
AQBI01000050-43908-44547
KK736261-2028135-2028705
KK327800-3119725-3120855
KB372963-34705-37642
JYFD01000220-20029-20301
CXPG01000018-2791-6053
KK356557-0-617
CP002049-2489904-2491457
KK333860-58-1262
KQ950181-364261-364654
KK338849-931204-932113
KK338849-934370-935572
CP000909-709625-731164
AIGZ01000021-143922-144439
CXXD01000002-92287-93049
GL397173-1457209-1457588
JJKR01000001-780064-780364
KK356571-1037498-1039242
KK356571-1039398-1039946
KK357943-483362-485227
KK357943-481494-481893
KK338747-2788349-2789118
KK338747-2790587-2790990
KK338747-2791786-2792538
AE016825-1293341-1294986
JYTS01000003-20059-20881
JNSA01000087-1920-2376
KB896840-384910-386953
JH815216-13178-14733

LINZ01000027-127264-127841
LINZ01000027-127751-127963
KK354986-2764806-2765798
ALXU01000028-45581-47993
ALXU01000028-47891-48191
KK328509-1114703-1116056
CP000155-2917500-2921171
JOHY01000050-987-2168
JQAO01000001-92205-93314
BBCL01000036-10-5379
JZDZ01000048-263894-264412
CBYL010000009-55497-60429
CCLY01000002-165250-165951
LATD01000178-1437-1894
ACYG01000030-196239-197782
KK339248-3145793-3147584
KK339248-3140817-3142247
CP009170-2268103-2273799
CP009170-2344709-2347139
CP007039-1230531-1230920
KK340311-3115010-3115701
KK340311-3233144-3233406
LAPF01000001-3900965-3902580
CP003125-2064939-2065907
LHDT01000001-234724-236155
KK328341-3120115-3121398
LM996653-1171488-1172066
HE972745-28136-34199
CP001826-23513-24703
CP002792-224643-230898
CP002792-1302585-1304621
JYYC01000003-6-1621
AY596293-35012-36630
CP007251-2966435-2967073
KK317050-3115503-3116715
KK316602-314348-315490
CP007258-2966766-2966978
CP007258-2966279-2966856
APUR01000105-23289-24314
CP012914-2425506-2426609
KK328417-808735-809866
AFYX01000011-270138-270648
LN851177-600757-601317
LN851177-484141-486903
BASG01000069-9060-10211
KK337594-1655-2879
GL622359-1488541-1495573
GL622359-1486878-1488641
CZVU01000003-15452-18767
JHQP01000012-9-5905
BAWA01000021-38871-40769
JQQA01000117-17774-18231
AQBB01000136-93918-95167
AMSH01000057-10341-10837

JQEW01000063-67089-68810
KK338947-3097897-3099265
JYZF01000045-19937-20942
AFZA01000010-269879-270397
CP003360-911734-919030
KK339365-3113700-3114475
KK328408-771380-773019
KK328408-773771-775257
LM997016-1012355-1012873
KK328014-1856-2911
JYYS01000049-105262-106267
CP007253-2971790-2972002
CP007253-2971303-2971880
KL406386-3128325-3129312
JYSE01000021-50-994
LDZK01000013-19616-21170
LOOG01000034-39-434
JAIY01000010-1234660-1236154
ALEM01000049-469950-470588
APYS01000159-508-1818
ALGU01000053-122834-123472
KK322701-838769-839769
ALET01000022-12-405
LMXM01000001-124544-129879
CP001100-2683899-2689160
CP001100-2689063-2694522
ANRG01000007-251735-253355
KK355285-1132479-1133014
EQ973643-603758-604056
EQ973643-603034-603858
JPKL01000016-85814-86268
CP009922-4973325-4974208
AM408590-3072632-3073989
LINQ01000011-15688-15900
LINQ01000011-15201-15778
KK322280-3141108-3142628
KK322280-3137500-3138849
KB946319-327723-328164
JYUM01000023-15196-16627
KK327929-1774287-1775432
LGYY01000138-54679-63888
JOER01000065-46440-48545
JOER01000065-38195-39503
KK333089-3269-3808
JGYL01000001-244601-255079
LOFI01000013-22296-25025
BALG01000005-2385-3811
BAJL01000012-14221-18757
KK321548-3142170-3143511
KK321548-3137752-3139193
JUDM01000067-46-1418
BANO01000140-1224-5474
LATL02000179-0-1949
KK322865-3123835-3124397

CP001940-1780098-1782570
CCXH01000075-6568-7084
AP014507-199002-202809
AP014507-1131811-1135249
AP014507-1146034-1147056
JAEY01000066-5229-7087
ALUL01000038-222596-224217
CP003633-54488-55197
KK355296-2187931-2189500
LLMR01000014-894-2183
CU466930-1486614-1498952
JDTH01000008-49621-54793
CP001808-65828-66347
CP001808-32200-33116
JKPJ01000067-1723-2169
KK213388-957467-957900
CM001274-2978956-2980634
KK355895-3152186-3153335
CCVB01000001-158922-159501
CP005287-2525396-2526157
KK322353-1384-2441
JEND02000002-86224-87107
CP001874-1730562-1735406
AP004311-16309-19901
ALEQ01000029-123093-123731
JQER01000060-66856-68577
KK322451-59-1129
CASQ01000036-108904-109855
AUBO01000073-388-4256
CZLC01000016-211327-214856
JGZD01000008-32990-42032
CDQL01000002-14145-14898
KK331499-0-626
AP012203-296281-300394
ALHD01000139-61722-62360
AQEX01000024-134067-134828
JRZH01000005-116080-117555
AP009256-1459648-1460282
AP009256-1460182-1461836
JFZT01000067-14055-17369
KK341005-136061-136980
KK327770-2244-3299
KK357435-3114649-3115416
KK339487-3129797-3131535
CEDR01000021-145191-145880
AHON02000042-48650-49898
KK328241-3119977-3121333
KK353575-1903690-1904680
CATA01000001-10179-11063
CWLV01000056-6377-7800
LHLB01000124-7442-8447
KK350912-3056159-3056782
KB908041-8715-13425
BBZA01000275-15903-16386

LHIC01000016-0-1065
AE009950-623118-625176
LRNW01000113-10185-11311
LHEO01000003-15183-16310
CP000680-4149217-4150162
AE006641-1254481-1255077
GL622296-522620-524723
KK339296-491653-493066
KK317294-63-1260
ALGI01000013-123092-123669
ALGI01000013-123579-123791
AKYQ01000021-13117-14402
ALIC01000031-203716-204357
CP002737-1002272-1003356
CP002737-592559-593660
CP002737-717056-718059
CP002737-1352424-1352940
CP003590-1226681-1229689
LGDQ01000002-24769-25042
KK312300-42-308
JOLZ01000023-86326-87027
CCNY01000001-824822-825094
JNOL01000009-496834-497402
JQAD01000001-97502-99809
JUFI01000099-184-2197
KK338925-2261865-2264189
KK339323-3261182-3262393
KK356400-3119817-3120660
JDTZ01000007-3008-6026
JQAE01000001-97502-98070
CP003479-1563835-1566442
CDLD01000015-35553-37350
AKWG02000010-35128-36027
BATA01000151-54-873
LOOF01000009-92112-92507
ALTQ01000019-276827-277058
ALTQ01000019-276959-278181
CP006977-1845967-1856887
BCQG01000002-81217-82371
JH165054-909845-911460
CM001403-7603672-7619499
JYPP01000055-88300-89977
JOHL01000066-20458-22255
JOHL01000066-32756-38190
JYWI01000067-0-1672
CP009090-2970456-2971033
CP009090-2970943-2971155
JQRV01000075-531-1839
BBXF01000002-314115-314813
HG916765-1034961-1038293
HE858529-1130920-1132211
KK335453-1874-3709
KL406150-14-1007
JQNI01000002-413505-416077

LABZ01000004-2777-5309
AEQD01000080-37902-39588
CP000876-134010-135605
JWHH01000066-458540-459963
LINU01000033-52472-53477
KB944688-883914-884215
KB944688-658769-659399
HG764817-5842529-5845164
JOLX01000009-61446-62390
CP009083-2971358-2971935
CP009083-2971845-2972057
CXYJ01000003-317981-318245
JQEO01000041-67087-68808
CP002400-674782-693586
LIMY01000002-258364-258941
LIMY01000002-258851-259063
CM001772-1749479-1756891
CP010340-3140328-3142191
KB894093-521645-522003
JQRJ01000140-12-1443
CXWW01000003-260249-260513
KK316614-3120410-3121618
JH470338-1276642-1279288
JH470338-1275484-1276305
JQNS01000004-29443-34932
KL406441-57-464
CP007260-2966572-2967210
AIHD01000060-158518-159036
AIHD01000060-184750-185145
CP001337-689333-700642
CP001337-4555003-4555086
KK341218-3049654-3051000
KK341218-3051417-3052036
JFJU01000010-153928-155427
CP007524-611044-612597
BAEN01000014-35-726
KK321117-3051079-3052261
KL407259-36624-37694
AEZE01000002-147637-148564
AEZE01000002-150895-153150
KK331527-15-1234
ALHW01000337-2932-3570
KK356050-3114929-3115329
KK356050-3118594-3120157
AQEL01000009-153756-154395
KK353403-3108556-3109255
KK319541-135682-136612
CP014358-3128631-3130185
AOMF01000033-33039-36146
CP002868-2131096-2133630
CP003369-21238-23875
CP003098-795087-798822
CP003098-837533-839153
LHDX01000047-6-1011

KK327888-3121635-3122181
KK321418-2801353-2803965
KK321418-2806705-2807842
KK321418-2805503-2806489
CZLC01000019-57341-64872
FO704551-546400-546978
KK328000-58-1414
CZVL01000033-3266-4495
CP006019-1004000-1005710
KK327775-1303962-1305601
KN360927-30577-31803
LN907858-1593569-1595058
KK354900-1117693-1118163
AUXR01000016-470900-472454
CP007528-1291292-1291930
KK316978-315759-318561
KK316978-314841-315602
KK339347-317623-319414
AOYN01000068-65499-67052
AROD01001420-18-421
KK357335-3150524-3151448
JLGS01000052-0-470
BBNB01000008-216158-217591
KK322269-3127886-3128203
KK321971-59-610
LCWO01000049-5-401
KK328055-1354082-1355729
AOYS01000103-104315-104833
CDRU01000209-11400-12648
JYQE01000015-44817-46127
KK353886-143062-143693
LIQA01000002-260607-261431
AKYT01000407-69-1426
JOQO01000031-56-512
ADVG01000001-2625448-2629072
CP001392-61494-62454
JENE03000001-288429-289312
GL834361-107993-120456
KK313417-13-484
KK321118-3050239-3050857
CP001787-743933-744769
KK322261-3131741-3132523
CCFJ01000043-128998-131486
CP010331-3051434-3052195
KK357743-482365-484675
KK318706-3116826-3118245
BAEO01000038-39741-41569
LGDF01000004-4534-5234
AMLS01000243-44-1537
AL954747-139663-140263
JUDR01000006-286521-291928
ANBA01000007-284832-286935
JUCC01000005-90641-91280
AHUK01000012-302626-302898

CXWZ01000032-8857-10532
JQES01000048-149058-150779
KK328743-3127392-3130370
KK328743-3124402-3126425
JQOG01000003-313745-314496
AZWY01000025-30090-32925
KK339334-3121931-3122476
KK339334-3123538-3124668
KK338859-3090806-3091926
KK338859-3092445-3093128
CP001363-3114455-3116070
JYXG01000028-10381-13380
CP009449-3031426-3032565
JXTG01000002-188924-190944
CP011055-2134654-2135250
FN434113-2914480-2916704
AYVJ01000044-88365-90285
KK327823-1350313-1350920
KK338960-146171-147242
LGUR01000230-318080-321278
CP008872-2818097-2819745
LMZE01000001-149834-150534
CP003939-1597611-1598169
KK355277-3514-4504
KQ950182-298182-300590
JPHI01000001-97502-99809
KK336730-0-694
LHMC01000003-258378-258955
LHMC01000003-258865-259077
KK328609-3128786-3130365
KK328609-3144043-3145174
KK328609-3136639-3137774
KK328609-3132756-3133980
KK328609-3137964-3140278
BAJM01000012-24446-27044
APYI01000052-43768-44407
AGIA01000039-105472-106905
JNIM01000001-3261585-3261928
JNIM01000001-1230674-1231521
CP002085-1844780-1865928
AKIO01000034-32-641
KK328494-3154385-3156329
KK328494-3149461-3150956
CP003040-2759397-2764809
KK354551-1129518-1130499
KK338949-3061065-3063087
CP003295-1273106-1273801
KK322634-315347-316410
JRZM01000006-116088-117502
ADUP01000034-119550-119763
CCTX01000002-775370-775838
KK355839-2782470-2783457
JAQC01000071-7583-8601
CXYP01000008-58845-59911

KK328279-1915-2811
LIMJ01000016-15377-16015
DS985177-298035-300490
KE702177-968096-968360
KK330736-1384-2527
KB907312-857477-862448
GL622425-6877-9883
LHKR01000036-50084-51089
CP001719-1033122-1037151
ANRF01000021-48153-48783
ANRF01000021-47493-48255
CP001737-3336814-3338504
CP001737-3348442-3355073
KK340389-3118264-3118884
LRNP01000002-114258-115628
KE387025-220422-222097
CCNU01000001-3545351-3545991
JQEQ01000046-149076-150797
KK328923-2776533-2778110
KK328923-2783426-2784847
JQOH01000005-183863-184851
CP008744-3052331-3053472
CP001778-3798896-3799945
AHMU02000009-29265-30814
ALFD01000109-19483-20121
AESR01000035-25-422
GL698429-772938-774164
ABZS01000002-10132-11615
LINB01000006-58-1124
CP000875-2842558-2851605
LLSP01000002-106233-106994
LHIT01000003-88308-89739
LHEA01000018-181337-182830
KB896018-84224-88889
KK322672-1046546-1047614
ALNP01000045-10520-10854
ATHI01000024-210-1655
KB903808-72599-78150
KK339197-483492-484183
JJTJ01000035-36421-37270
AQBM01000055-43882-44521
KK322546-3113475-3114251
KL405696-3142974-3143957
CP011057-2134654-2135250
KK320223-19-1059
LONM01000033-40-435
JPFR01000065-66970-68691
BAXP01000029-14854-16340
CP002427-1307835-1309024
AMSH01000007-22017-22512
AL513382-2926180-2926563
LBHA01000124-24092-26198
LBHA01000124-33135-34443
KK316996-16340-17316

KK332861-2159-3827
CUWF01000001-3119180-3120464
CCNK01000042-1511-3936
LHHX01000018-432580-433524
AHBF01000056-16974-17244
JWRD01000014-288812-291872
KK320381-58-1041
LHOL01000012-204007-205012
LBIB01000002-211902-213701
CP007261-2975462-2976100
LHIU01000050-48-382
JOMG01000002-2067483-2069493
AOHP01000095-14866-15811
JJVP01000063-438-1930
KK338897-3107053-3108565
CARV01000006-126856-127220
CARV01000006-126640-127007
CP003732-2443640-2445333
AKXB02000080-268-1089
ANBA01000009-275820-279695
CM001484-2340904-2341236
JNOK01000009-496932-497500
CUWN01000001-3131345-3132702
ALOD01000110-9782-10082
ALEV01000023-123108-123746
LHTA01000001-762228-763131
KK321999-1491060-1493000
KK328979-2791680-2793106
ANBB01000002-24574-25031
KK331943-73-1301
LGCI01000010-11879-16485
LGCI01000010-21204-25587
LGCI01000010-769927-775044
AKWV02000063-79881-80824
LINW01000001-257960-258537
LINW01000001-258447-258659
CP001404-1893531-1897307
KK328894-4298-5438
CCBX01000123-15089-16337
JXUQ01000001-3868242-3870772
ATHI01000019-51-1929
JRYU01000002-160577-162542
CP001130-1037775-1039663
AQAE01000088-108224-108986
KK321399-0-1941
KK321399-3410-4808
LADR01000053-38008-47735
KK322053-59-1057
AHVA01000007-275444-276982
LHFZ01000012-94582-95587
KK308677-1415-2111
CCUR01000002-275604-276244
KK327808-3061411-3062978
ALHX01000052-122960-123598

JWQF01000006-449260-450984
AKFW01000004-123086-123663
AKFW01000004-123573-123785
KK322520-3145760-3146615
CP007249-2972642-2973280
FQ312003-3116351-3117723
KK322160-1512893-1514306
CM001974-1587714-1589260
KK350952-2020227-2021441
LAPG01000086-361763-363378
ANCX01000001-273250-273682
KK316649-999982-1001125
KK335301-130-1415
KK357663-3122135-3123416
KK324016-58-755
AP012322-1483172-1494077
CP012889-2205468-2211639
CP002216-91067-95394
KK357357-136061-136837
ASSE01000008-28-328
KK334363-1-545
KK357282-482400-483162
CP001696-760022-760580
CP001696-281437-282884
CP010333-3281393-3281618
CCDT01000005-32395-34997
CP008746-2376807-2378788
CP000423-356873-358122
KK356838-135571-136418
CP001634-439675-442253
KK355922-3125110-3125957
AZWU01000030-72254-72588
KK328928-2812216-2813560
KK328928-2813725-2814719
KK316624-3124385-3125961
KK356449-1613-2160
JWHJ01000048-458540-459963
CP002583-3643809-3646958
CP007453-17327-21913
AZAX01000004-29265-31909
KK327763-1767126-1767733
KK355887-1124933-1125701
KK328879-2791380-2792811
AHUR01000022-96506-97144
AKWG02000077-27296-28059
CP002017-2750069-2755046
CP002017-2755016-2768077
CP002017-2770186-2778650
JYSY01000022-0-1247
LHOA01000003-177553-178984
LGUW01000145-3313-6643
JMLA01000001-4025896-4028916
LHHN01000004-234724-236155
JH590866-511345-513762

DS264586-538364-545839
JXWA01000012-107690-108391
JQHP01000005-183863-184851
DS990394-14974-17062
JMCG01000002-12032-13860
AZAC01000078-41364-43041
JZUT01000004-246680-248903
KK339117-821056-823306
KK339117-818805-820742
KK355900-3064061-3065274
AOHP01000019-0-758
JYTU01000028-219134-220749
KK334218-1656-3502
JWQZ01000019-18271-21270
AHMF02000100-12993-14526
KB849164-898955-899823
LLJK01000001-254833-255367
CP009085-2971483-2972121
JUBL01000002-180184-182839
CCTV01000001-2387959-2388641
AQBN01000051-43839-44478
CCQN01000129-208670-209249
AHZD02000018-85321-86083
KK355226-1125706-1127062
AADL01002507-85456-89882
JPON01000001-2974571-2978624
LHEI01000048-33021-33801
CP014051-2579182-2580719
ALGZ01000064-251938-252576
AOTM01000161-4739-8538
LGDS01000196-3443-4576
ANMY01000055-76483-77730
CP001931-177440-180135
LINC01000004-502133-502771
LLUO01000042-22222-23871
AQBL01000050-43784-44423
AKWK02000026-66564-67241
CP002444-1166825-1168349
KK356246-485181-486468
KK327802-803203-804338
AMJQ01000130-25866-26261
KK354175-136240-137012
KB947334-1527899-1528199
LN810019-3995460-3999627
ABXB03000002-138643-139219
KK322458-3133901-3134972
CCLL01000004-1789844-1790483
KE701870-61-1005
CAAD010000050-98244-98721
KK328290-1534-2557
JGYU01000010-27135-28322
KE356582-87995-88616
CAPB01000035-90278-92441
BBGT01000081-2233-4274

JRGA01000002-363128-364173
LHEM01000001-226905-227910
ACVN02000303-16037-21117
AP009552-2826227-2829166
JYRX01000041-6-1621
CCSU01000001-958262-958791
JYTI01000034-5587-7201
AKWG02000008-25891-28373
LAYT01000238-3300-6272
LGYH01000002-160947-163756
LGYH01000002-160749-161049
JYTG01000013-0-1127
AWOI01000099-64882-65520
JQEM01000050-67083-68804
JYRT01000086-5344-6410
AHQG01000301-530-924
AZCO01000048-3034-4283
AP012325-1508551-1510594
KK353410-952621-953470
KI913112-4178230-4179833
KI913112-753466-755272
KE951406-18946-27338
CP007259-2967538-2968176
KL407340-2798293-2798836
KK355846-1399146-1400939
KI911562-2350162-2353208
JH376576-20835-24100
KI911784-3102944-3111673
AP009044-792954-799754
KI911520-103111-104054
KK338758-3096738-3097735
CP011666-68592-68927
CP011666-52116-52694
ALGJ01000026-123093-123730
CBYN010000031-5430-13886
KK329134-0-1063
ANAC01000036-26527-27288
AZWM01000002-2755-4799
AP012492-1362504-1364592
AAQF01000292-828-3593
CP001901-1004050-1004622
KK334993-221-1367
JZAJ01000043-15278-15797
CCLJ01000001-177274-177546
JYJO01000011-5812-7235
CP013097-2968493-2969131
KK339242-815598-817033
LGKH01002224-299727-300470
BCNX01000006-72757-78072
BX248353-2306021-2307638
JRYN01000001-266349-267375
CCUI01000001-3601773-3602045
JQAH01000001-97503-98551
AKGA01000007-258712-259289

AKGA01000007-259199-259411
AQEP01000012-776913-777675
KK312481-42-308
KK328041-3109941-3110924
KK328041-3112393-3113372
KK328041-3106962-3107730
LHLH01000007-56-1305
FR733645-2697466-2698758
FR733645-2697202-2697568
KK350957-3131789-3133513
KI911560-1235053-1236765
JWIA01000142-34-2129
LHMJ01000049-56-1244
CTDX01000015-369231-372757
HF680312-2343574-2351279
KI867150-2370437-2375115
JYMG01000019-24943-25811
KK355577-3104257-3105168
CP007631-972926-974942
JQRQ01000001-21020-21660
JRZS01000013-106061-107476
CP007581-3128679-3130233
JYPW01000009-433172-434177
LHTI01000001-476386-477391
KK327963-1760178-1761391
KK327963-1757403-1758684
JOQI01000059-2249-2705
BCNA01000001-526361-528930
AQCP01000058-95828-96589
ANAM01000008-563675-567588
AP009493-4152729-4153732
GL383090-187567-187840
KK353342-3135808-3137605
KK353342-3137711-3139200
AFIN01000194-16186-16949
CP004050-134104-150352
CP012095-3082962-3084683
ANAZ01000006-44-1844
KK339150-484187-484806
LHJH01000047-27242-28490
CP012381-596135-598034
CP012589-1486602-1489992
JNPV01000010-18457-18850
KK328434-820376-821201
CP003776-420053-420937
AOHI01000054-67825-68768
KK350899-3051782-3053057
CP001687-1415737-1419119
CP002835-3459884-3464595
LHKJ01000018-77487-78492
LGZO01000016-65466-66471
AROF01001187-725-1213
AROF01001187-548-820
AWNH01000018-568-2258

ADNB01000032-341-2138
AKWE02000112-29026-29481
JYUL01000012-15164-15741
JYUL01000012-15651-15863
APYM01000176-518-1828
APHE01000064-44614-45831
KK337375-0-1239
CCFL01000042-1511-3936
CP007523-3778345-3780448
JJPQ01000106-6131-15192
KK357423-2228438-2229794
GL573157-523515-524475
CZVI01000044-10940-13174
LKJD01000079-8664-10506
CCTD01000001-236349-236928
JYTL01000028-5334-6949
KK342213-430742-431008
CP010411-1537289-1541350
KK327833-2640969-2642609
HE978511-1095565-1097799
CP011542-2748619-2754129
CP003969-8415641-8416646
JDTY01000002-245041-247387
JWZI01000003-149968-150730
AKWF02000085-26886-27464
AQBQ01000058-43817-44456
KK334741-58-900
LOBY01000137-806-1551
LHDO01000065-33022-33802
KE375783-83458-86664
DS570395-38484-38819
DS570395-54717-55295
CP011449-2202771-2204856
ADJS01020203-19332-20293
JFCH01000046-14549-16649
LHOO01000017-94582-95587
KK339309-486659-487859
KK339309-482887-484314
KK328184-805953-807364
KK328184-810588-811284
KK328184-808065-809120
CASH01000036-108911-109120
CASH01000036-109772-109975
AIHQ01000023-132706-134017
CP000033-1541099-1543018
JWYX01000037-86264-87270
BARH01000009-167939-169635
JZTU01000007-289124-292123
HG313817-66049-67419
CASK01000006-109234-109509
CASK01000006-108002-108769
CASK01000006-108884-109327
KK338483-3109255-3111118
KK327758-819559-819893

ALGQ01000035-122972-123610
CP000113-8884280-8888066
CP000113-8579389-8583254
DS999574-1059673-1065559
JOFE01000013-190012-190957
LJEO01000041-11839-12830
LDZH01000005-174955-179071
LDZH01000005-189803-191663
KE386770-93722-96224
CP003380-1061662-1065770
LLNO01000015-37388-38316
KB732975-378766-379694
BAEM01000041-22703-24829
KK336513-2-1540
KK340207-321752-322740
KK338617-1-1072
CP010529-2853937-2856505
CP002865-1311052-1312057
AZVX01000021-76796-77862
AP012212-2161500-2165127
CP011056-2134654-2135250
LHLR01000005-346443-347710
JYWC01000043-16273-17704
KK323490-63-1429
LHGQ01000030-54-1425
KK354825-1493635-1494036
KK339568-289624-290540
CZJZ01000009-833451-836980
CP009093-2962954-2963592
CP001393-151653-160400
CP001393-2866257-2866746
CP001393-2866651-2868124
CP001393-2805781-2807335
FCOY01000004-51958-60267
AFVX01000016-1675-2070
JOFV01000013-66375-71534
KK316824-481410-481883
KK316824-481775-483121
CP000568-1712608-1719089
KK355415-1116854-1118577
CASS01000001-109485-110321
KK353375-583278-584841
LJOJ01000070-2480-3121
AE017282-679950-684452
CP002738-1526145-1530434
KK336994-61-1731
FO704550-2323034-2326769
KK328720-2791613-2793326
LGGZ01000108-3309-5323
CWKW01000045-5492-6915
CXXU01000011-66465-67653
KK357677-58-1120
KK317060-315649-316550
KK339134-486190-487824

KK213378-637196-637431
KK321735-3128663-3130754
BCQT01000032-1228-2058
CP000575-395458-395995
KK353768-483814-484365
JYSA01000014-317308-318801
KK320940-1-1415
LHJA01000043-5474-6479
CP002831-145369-153339
CP002831-3742955-3753431
LMVI01000001-764229-764529
KK307882-211-1120
LDNS01000095-8921-9413
CP000859-3590107-3592515
LIND01000016-15650-15862
LIND01000016-15163-15740
CP007051-545524-549853
JOFJ01000001-62670-67041
JOFJ01000001-76532-80774
KK320885-58-751
JUDP01000007-57-635
CP003198-1107550-1113295
CP001087-629856-635494
AP014508-439363-441307
ANQQ01000012-91146-91776
AYQA01000010-38372-43236
KK355856-1709-2177
JQEX01000051-149191-150912
CM001022-298106-302945
CM001022-607349-610496
AP014568-1275538-1279909
KK328606-2310395-2312030
KK328606-2305137-2306128
KK328606-2299183-2300903
CAPF01000063-35899-40482
BAJJ01000019-44273-49300
BAJJ01000019-52438-54988
KK356498-2776489-2777847
BBIH01000109-2379-3278
KK328378-1761404-1763043
LJGO01000094-32316-33964
KK338939-3096168-3098252
KK338939-3092752-3094699
KK342194-599268-599534
AP014924-245295-248298
CP011522-3813332-3814214
KK327785-607276-608114
BAKQ01000001-127235-128177
JOLP01000024-68198-69142
KK322466-311600-312954
KK317027-3058800-3059482
JOBV01000049-170-3472
LGKN01000010-524-2290
JASU01000001-2946221-2946919

AGJL01000056-10287-10765
LHGX01000078-27281-28286
KK355397-979-1586
KK328477-1495677-1497543
AOCG01000002-264628-265060
GL831112-1226948-1227576
APGX01000065-420355-421572
LHHA01000032-5531-6536
KK318774-59-1231
LHGD01000025-124362-126466
CWMA01000070-5574-6997
KK339104-612844-613965
LGRF01000003-166742-167370
JH976535-1089516-1091216
JH976535-1091108-1091869
FP929045-2155457-2155964
AQXD01000005-91031-92137
AHOK02000012-136540-137239
AQCO01000060-135429-136129
JYUF01000010-469487-470918
KK353865-595344-595606
KK307981-136242-137524
CP009517-3729888-3737068
CM001475-3123008-3133609
JQQA01000136-14514-15155
CP001683-2195668-2196549
CP001683-1762886-1764626
KQ235753-192552-198059
LFCW01000016-35614-38389
JH376832-136474-138247
CCLR01000001-177053-177325
KK330982-1815-3596
KK327986-3915903-3917550
CM001858-3657764-3658258
CP002859-5772287-5784494
KK356328-3125370-3126716
JTED01000005-6346-7718
BBSJ01000047-12702-17782
APGY01000014-44612-45829
CP009084-2971482-2972120
KK334206-6-1587
KK328467-827367-828348
AHUJ01000034-226133-226405
KK317045-3065259-3066392
KK355876-3132842-3133383
KK355876-3125230-3125928
KB900536-1338908-1345409
AZIS01000003-123885-126965
LHGN01000015-286485-287752
CP001720-2200136-2202288
CP001720-940047-943825
BCRR01000025-98-675
BCRR01000025-18173-18827
CP010067-2091484-2092957

CP002196-4426-7627
CP000478-3489021-3493873
CP009503-3179667-3183450
ATXO01000008-65196-66406
KK319891-1414-2918
KK353680-437155-437397
CP006905-2331403-2332023
ATJV01000001-212280-220056
ALUV01000234-6683-8367
JNMV01000109-45-440
JH815593-1534551-1534978
JH815593-1534876-1535176
KK339957-1500355-1501279
FAUR01000062-54677-55009
KK307568-5-404
AIHS01000033-39823-41134
KK333161-2157-2920
HE578925-94673-94912
ALGW01000059-123356-123994
GL884456-142086-142787
KK336504-1638-3288
CP009089-2972017-2972655
HG326225-345118-347381
LBKG01000024-281206-282034
JOBO01000019-112776-114880
JLGR01000070-985-1749
KK214998-1210323-1214134
KK356917-3135219-3136130
KK356917-3139081-3140066
JHUS01000043-1400-2951
CATM01000001-108179-109530
ANDA01000105-168362-169982
KK328732-2785558-2786966
KQ236589-43793-44423
KK322792-135657-136649
AJTS01000045-19238-26165
LOEL01000001-92893-93259
KK354080-3119773-3120983
KK341544-3017-3923
KB892099-78713-80757
KB892099-82005-83610
AHOI02000607-11145-12146
JJOX01000086-4545-6718
LOPB01000003-104365-104629
JQEU01000060-67027-68748
LHLW01000003-29077-30082
CP009088-2970858-2971070
CP009088-2970371-2970948
KK341741-606340-606602
ABVG02000001-182014-187860
KK341955-3052897-3053879
KK341955-3055298-3056868
AE006468-3076616-3078154
AOGI01000007-151173-152239

AOGI01000007-150154-151263
KK320664-0-690
KK328594-57-1477
CCSK01000001-276923-277195
KQ087694-124431-125194
KB898398-76-410
AOXB01000062-69984-70927
KB901875-33975-36335
KK310941-0-1065
JH378029-1403862-1404745
CP003900-3042280-3043637
KB733395-697177-698225
KK353240-770679-771744
LING01000021-16273-17704
KK339272-490195-491619
KK339272-484148-485360
KK339272-498023-499007
LHJM01000001-60001-61269
KK321722-2796968-2798562
KK321722-2791140-2793007
KK321722-2783866-2785147
KK321722-2789174-2790914
JFCQ01000009-2025-3394
CP010451-369533-372496
KK317073-3119737-3120278
CBLT010000011-65018-66754
KK355624-1414-3210
CP000240-866109-867185
CP000481-2208609-2210116
KK309579-42-308
LFUS01000031-52243-54411
JYSQ01000009-0-1491
AY596292-20532-23194
KK322844-3116469-3118106
JNVT01000096-18733-22241
JXLL01000012-48120-52449
CCLN01000001-259534-260296
LIQT01000004-9226-10658
CP001800-2076432-2077028
LGCI01000009-11370-15954
CP000768-1654297-1654663
KK318256-135571-136345
KK327850-3111867-3112712
LN868937-753704-756192
KK341828-1590632-1590898
CP009528-248100-255527
KB733122-2883105-2883500
JGZO01000006-187162-188900
CCUE01000002-242123-242763
ALFJ01000031-123034-123611
ALFJ01000031-123521-123733
BBBN01000234-228-2146
BA000048-1130820-1134377
KK317480-63-1194

KK327502-178351-179540
JTAU01000001-4305460-4305912
AP009389-1998602-2014655
LDYC01000023-16912-18137
AAWS01000010-193962-196271
KK322769-1125448-1127459
CP000504-31-1216
ANYY01000061-22061-22944
KE701201-148594-149172
KK322595-853388-854145
KK356735-1676-2671
BCON01001204-873-1435
CP002630-1465974-1467823
AOLR01000061-16828-20535
KL406066-2000-3376
KK328221-780350-781556
KK319689-57-316
CP007247-2965304-2965942
CP007633-983575-986933
AP010655-743990-748582
AQBO01000054-43721-44360
GG663535-1500013-1510735
KK328105-3124303-3125351
LNAA01000003-494742-500077
DS990539-7931-8897
CDRZ01000237-6866-8274
KI912266-1960428-1963932
KI912266-1963843-1966433
JLJQ01000012-135675-136594
LACO01000001-495965-498589
KB892068-118263-118903
KB892068-107435-108376
AOXE01000072-3-1556
JWRB01000006-98659-101353
KI783301-2407387-2409461
KI783301-388872-390782
CP001091-243045-244812
JGZH01000004-57322-61804
JYQT01000002-107795-108740
KK339395-12176-12650
HG964454-118858-121032
KK321804-1869-3689
JYWB01000014-77489-78494
JWZC01000049-0-456
KK339269-3101563-3103885
KK339269-3107361-3110633
KK339269-3100484-3101400
LN868537-519136-519976
KE699590-1670433-1670950
AP013035-1054584-1057199
KB889965-3508120-3509166
KB889965-2788037-2791312
FN666575-854677-856780
KK353266-1770419-1771481

CAOS01000004-71021-75642
KK328500-2802118-2803185
KK328500-2798677-2799223
JYVI01000016-156341-157224
KB912485-47805-50031
CP009220-350834-355749
KK338878-3102033-3104341
GL501401-278974-301556
CM000604-3289127-3292053
JRZO01000045-106674-108590
CVPE01000004-335961-342161
CP002656-985208-997384
KI535580-196208-197581
JRUZ01000019-3733-6506
JAFT01000007-31313-35797
CM001580-366391-367919
CCBW01000093-68-646
LROL01000061-89-545
ALGO01000052-469793-470431
JYCF01000054-52540-55497
JOQQ01000062-2325-2781
LHJN01000009-49-1054
AQFI01000022-171538-172299
LCWP01000036-25-420
KK315813-42-308
AQBP01000060-19597-20236
AOHH01000189-30210-31032
CP002457-731076-732724
JNXC01000049-31247-32740
CP001014-1036119-1038486
CP001014-1017597-1019142
LEKI01000151-4751-8616
LHHC01000013-77487-78492
CM001487-1908354-1913378
KK354779-138697-140266
LHIO01000009-233490-233702
LHIO01000009-233003-233580
AP012340-3228272-3228539
BBCG01000008-106649-106876
LIMF01000003-422705-423343
JFFO01000087-66393-66601
JFCP01000008-51-1968
CWMJ01000108-5840-7263
CP006714-1635872-1636582
BBDF01000008-3005-4375
KB905815-526336-526854
KK316087-58-975
LRNV01000073-120-1185
LRNV01000073-2-207
KE384039-212382-213344
ALUQ01000040-0-1155
LHNK01000007-267186-270063
LHMH01000013-51088-52093
APHX01000152-2892-4813

CCUN01000001-3592058-3592330
KK357933-2242608-2244550
LGCZ01000010-3443-4576
KK340081-2772007-2772400
KK340081-2768006-2768997
KI913020-1522811-1523046
JPEN01000103-56647-58134
LDZN01000001-626263-627791
CP013099-2656664-2660295
CP013099-2656124-2656752
KQ950180-1180547-1181737
KQ950180-897776-899751
KK356210-482103-482649
LHLI01000019-27055-28486
LACN01000012-68185-69544
ALXW01000020-46008-46902
HG916826-4455182-4457247
HG916826-4439046-4441654
KK354051-782000-783440
LHLB01000030-60232-61500
CP002638-2529911-2532438
CP003991-40552-41008
CP003991-121-1003
KK350929-1027-2082
CXWU01000004-107584-108285
KK334131-59-834
LHNT01000003-226590-227595
KN127219-400876-401242
KK339127-482256-483613
KK339127-483828-485188
KK355929-3127247-3128083
KK321818-2784328-2785899
KK321818-2789920-2791144
JDFY01000001-2196110-2200459
KK310725-42-308
AOXW01000042-17047-19986
LHJV01000009-60001-61269
JNZS01000015-74920-77142
AP012344-1346373-1347400
CM000776-1556546-1556780
LGDP01000204-3828-5078
ANRY01000020-18874-19504
ANRY01000020-18214-18976
KK327822-3052572-3053185
JNLL01000001-909312-909890
KK338855-3123862-3124772
LINA01000068-1770-2775
KK327855-780963-781570
KK328275-57-389
JOLY01000003-195008-195708
LK391695-4449689-4452297
LK391695-4465825-4467890
AL732656-951907-952801
CP000956-54527-61224

LHII01000015-15211-16642
KL362962-280910-285389
JQAG01000001-97502-98070
KK321413-1726-2341
KK321413-5068-6129
CCLW01000001-3577562-3578080
AHUL01000091-31172-31810
CWLK01000003-5572-8184
CP006593-790609-792097
CXYL01000002-100355-101117
KK328515-146132-147770
KK328515-140633-141690
KK322817-3123849-3124827
LIOF01000030-43-1537
LHEF01000035-16244-16881
CP000267-4365456-4371084
CARW01000044-110222-110435
JZAF01000102-1-1024
JQRG01000069-70382-71325
KB949589-295529-296225
CP010432-1541378-1543297
LGVW01000012-225208-225656
KK328295-3131805-3132935
AERU01000003-64375-65441
JNLO01000001-260072-263144
AOYU01000089-156368-156885
AHOR02000015-22918-23819
CP014230-2227049-2228900
BBPN01000011-49268-51001
LIVK01000012-94402-94980
FP929056-2091100-2094344
JWRC01000021-80896-83669
JQMP01000003-80118-86300
JRZN01000049-114564-116418
LJZQ01000011-15693-20751
KK321895-296-1684
CAPC01000011-100699-102923
CP012382-6984781-6985602
CP008860-1543748-1544556
CATU01000002-0-394
CP012514-2943249-2944315
KK357076-2795237-2796080
KK340423-1657705-1658771
JATG01000004-458512-460723
CP003607-7673106-7673726
CXYR01000014-18056-18390
CXXB01000008-435067-436256
KK321411-3056077-3057493
LDDV01000052-101748-102144
JPRU01000002-145951-148054
AZVD01000011-124459-125159
ATHI01000018-50-1870
AMSN01000068-7713-8840
KE150262-869789-873051

KK329826-0-699
ALEX01000037-123048-123625
ALEX01000037-123535-123747
AP009380-2181483-2187654
JYVT01000012-128629-129999
AARR02000019-44886-47498
JUDG01000016-107505-108144
CP007456-1714302-1717226
AWTD01000008-233727-234295
KK350950-1786894-1788309
KK316642-3122320-3123735
CXYK01000011-94299-95243
KI912105-2879697-2890207
LHDZ01000007-213785-214669
KB944824-484489-484723
JQAC01000001-97502-99809
JATD01000008-70163-73039
JPOV01000001-1183790-1184486
CP002040-1645358-1645629
CP002040-2013510-2015306
CP014222-171776-173575
CP007030-1357951-1362486
LDNT01000005-8909-9401
ACUK01000006-9191-9787
KK353372-2296345-2297550
KK322277-1494120-1495836
KK353220-3131630-3133343
CP003216-31840-32965
KK339663-0-545
KK327846-1468003-1469653
LINH01000010-127788-128000
LINH01000010-127301-127878
KK314818-1-695
CATW01000001-289170-290593
AXDC01000030-690-3604
CP003496-274751-283929
JNMI01000110-45-440
KK321916-1428958-1430621
KK321916-1422838-1424552
CARM01000005-609-992
CARM01000005-0-699
ANSI01000019-19436-20660
BAII02000009-275579-276932
ALER01000028-123062-123700
LDNZ01000113-8582-12788
JH660698-374987-376545
AZWT01000027-75399-77315
AQBK01000054-43883-44522
KK333319-0-2409
CP013741-3065705-3066990
APZX01000443-43754-45674
KK328439-2800877-2802085
KK321484-1414-3359
CCUS01000001-160144-160662

KK327738-3118988-3120204
KK342440-3121664-3122640
LN866274-2614450-2615332
KK320438-2716-3322
BBPO01000028-68506-72925
LBMZ01000120-43859-46228
JOVW01000011-0-1157
CP012147-38195-40115
KK316833-3132380-3134390
KK357157-810786-812060
CP009092-2971516-2972154
ADJS01013055-4964-5664
LOEL01000010-10058-10753
KK317177-63-1444
LIRE01000736-2-736
KK322647-1060439-1061581
APYP01000051-501-1811
JQAB01000001-97502-99809
CWLD01000044-5482-6905
ANMY01000033-2092-2913
LN999997-2324614-2326517
LHHF01000009-94582-95587
FR880553-21192-21963
LBCJ01000068-44-1070
CP002280-2368504-2369637
KK338837-819611-820538
KK316908-64139-65428
AENP01000006-71961-76429
KK353539-2784594-2785728
BCAG01000003-1041541-1043555
ABYK01000079-3193-6404
CCMO01000257-73019-74121
JYWE01000034-20136-20591
CP012196-71179-74031
CP009243-3065410-3066767
LM996576-407988-408383
AQCT01000107-105668-106338
ADSA02000075-13996-14446
JQRM01000024-70380-71323
JNPO01000015-2117-2510
JYPX01000051-16513-17533
LIOI01000010-258860-259072
LIOI01000010-258373-258950
JAQA01000017-226120-227003
KK339070-3138070-3139125
JYXW01000051-16146-17761
LSBP01000011-18286-26796
LIDY01000001-106207-108174
CP006594-335971-338583
FR884407-59-1036
CP003214-42132-44725
KK316487-42-308
CWLY01000016-160133-161556
FM999788-1921684-1923370

LACM01000001-169110-172940
LOOU01000004-167868-168132
ADJR01000026-86440-92734
CP003836-3074260-3075737
JRZE01000006-544994-545816
KK353954-173122-174041
KK356933-3119071-3120420
KK356933-3117399-3118316
LCZU01000035-153578-155462
AQEH01000055-158431-158695
JTFF01000004-190333-190972
JOIG01000015-60464-61225
KK338497-1493953-1495835
KK338497-1498776-1499771
KK338497-1497304-1498584
KK314046-42-308
JYSB01000002-88300-90220
KK340570-3097895-3098810
CCUV01000001-160144-160662
CASN01000004-108003-109048
LHMQ01000014-46429-47697
AAMJ02000003-25865-26260
AHUQ01000056-223547-224185
BCNW01000001-2292528-2300490
BCNW01000001-1249887-1253638
CP014275-2307636-2310127
LN868944-235472-238471
AQCV01000056-104619-105319
AP010918-3065405-3066762
LHIS01000001-0-1187
LN848277-24-865
APYL01000053-487-1797
JYUA01000002-0-1005
CAPI01000126-28281-32865
KK339811-3116011-3116930
JNAF01000004-135324-136609
LINT01000038-29686-30876
ALFB01000016-123123-123761
CP001185-1959887-1963518
KB900148-126518-127224
KB900148-127133-128511
JRZV01000007-123720-124969
CP009583-27291-28725
CP002095-3051997-3053354
JYRR01000009-10329-11335
JNMV01000117-1667-2062
CP003494-2783470-2784538
CP007175-2967608-2967820
CP007175-2967121-2967698
JOQJ01000106-2474-2869
JYON01000039-18-3974
JOLK01000021-118690-119391
AJLK01000058-13772-18917
ALEW01000013-122963-123540

CP007267-2971906-2972118
CP007267-2971419-2971996
AP012331-2307004-2308803
KL407303-3118186-3119331
KB891921-4586-7670
AKVS01000066-149073-150182
AGFD01000009-73504-79455
JYRW01000017-88301-90343
AMRI01000011-273-789
KK339124-3247209-3247471
LJBR01000161-54267-56914
CASD01000054-97429-98071
JQIP01000024-15112-20548
KK337706-58-754
GG749281-110037-110942
CP003264-225723-227879
LHDR01000040-55-877
KK333857-14-1819
KB896388-79788-81227
JYVN01000015-209507-210816
CP009788-2418755-2420931
JQQB01000062-20693-22182
KK328615-3138353-3140142
ALRU01000104-885-1845
KK327777-3582572-3583563
AEOS01000293-7958-10368
CP002589-2808032-2811108
AM412059-3065127-3066484
KI912258-253821-255375
KK339662-9807-10427
CP008959-22036-22231
JKHL01000056-58-681
GL982489-155560-158197
JKHL01000011-136239-137523
CP007028-859097-860004
CP007028-95669-96909
AP014510-238238-244124
BBOX01000149-19232-22190
KE137288-1406977-1407494
ALGR01000022-302549-302881
KK330096-56-1035
LINL01000017-88301-89855
ATXC01000001-645290-647530
CP000386-262282-263239
GL397254-1907727-1909413
JQRL01000032-461-1403
KB733078-1689601-1690545
LADO01000050-0-4822
AHOE02000043-81677-83230
AOZQ01000040-1-824
KE387027-7586-8591
KK350910-3122805-3124089
CP001807-539685-544166
GG669566-1141001-1142311

JYZY01000006-226590-227595
CCTY01000001-159370-159888
CP006597-1643360-1645973
AKWB02000004-11919-12618
GG753640-247392-252056
CP003001-2564412-2566153
JUET01000062-1728-6136
KK328337-802073-803208
KK329115-0-548
KK328101-3137592-3138647
KK328101-3129362-3130207
CP003155-3520455-3523595
AHOP02000060-61173-61873
KK357293-3113568-3113972
AFWV01000012-93498-95142
JOQO01000183-561-773
CCVA01000001-160144-160784
AZWM01000041-1566-3179
CM000855-1456508-1458715
AKWH02000016-87992-88670
LPUG01000009-376156-380675
LN898221-698473-702168
CATJ01000005-109632-109845
CEKE01000067-154108-156981
ALEN01000037-123094-123732
CP002104-836406-838204
LAOU01000011-422637-423275
CCQX01000013-39431-40742
CP003032-1066225-1068633
CP002326-2497047-2506688
LAVM01000042-7661-9521
LAVM01000042-19070-23064
CP002364-1701540-1707620
KK333448-0-1640
LINO01000026-60001-61269
KK328705-2793144-2794853
CYST01000082-45756-47113
JOLN01000023-18652-19352
CP009507-2312020-2316133
CP009507-2243218-2245294
JOEP01000001-323146-326000
JH600068-3179972-3187081
JH600068-1177874-1179442
KK337602-170-1744
KK350942-3052151-3053129
KK327881-482507-483421
KK322225-59-1199
CP001933-149124-149932
AQBS01000051-43771-44410
KI519072-1066586-1067355
JYVC01000007-501926-502564
LKCS01000008-212736-213168
CM001977-413013-414384
ALXR01000005-157640-159932

JLPO01000053-354-1787
BCPK01000154-110427-114060
JYYD01000004-15420-16425
ANQO01000012-145254-145685
AFCE01000229-66-1370
LBBN01000063-18-1327
JUTD01000067-14104-14801
JUTD01000067-15425-16979
KK327780-3108201-3109197
KK357957-822117-822963
CP000612-1079903-1083390
LIMS01000062-5551-6189
ATVC01000025-33107-34172
CARQ01000001-289548-290377
JYWE01000011-232947-234967
JYZQ01000017-27258-28323
BCPF01000079-5321-5883
CZJZ01000020-8608-16139
KK356209-2-1643
CP007246-2967338-2967550
CP007246-2966851-2967428
CAIQ01000030-1750-4799
KB900614-1764078-1765144
KK341220-3052183-3053671
AP012332-697300-699097
AKYV01000111-2760-4117
KK353314-3108250-3109025
LNIE01000214-9665-10304
CP002292-1352287-1357227
CP009091-2964898-2965536
CWKX01000041-6352-7775
LRNL01000001-31-915
KE136913-906574-907029
FR733646-2690252-2692864
AUBM01000006-284561-285688
KK353400-3137957-3138797
HG005289-170719-173386
KK335976-4-477
CP002487-3116351-3117723
AHJJ01000197-0-772
CXWR01000003-99037-99738
CCNX01000001-3691686-3691958
KK321552-1496524-1497517
CP006602-4349997-4351612
KI515688-889117-889389
CATT01000008-52618-53989
KK357268-3116970-3118614
CM001838-3665100-3665567
BAYW01000010-191398-193562
LCVU01000014-60-954
AIHP01000021-763178-764489
ALEL01000035-31-244
KK332079-70865-72153
CP007559-986598-987846

KK357085-3143989-3145050
KK357085-3149425-3151318
KK357085-3138072-3139212
KK357085-3142934-3143842
CAMA01000153-9886-11257
JAGA01000003-949272-950251
KK327957-3054543-3055887
AJMP01000045-81765-84046
CARB01000003-108140-108820
CARB01000003-125930-126203
CARB01000003-126113-126659
AHUZ01000007-275417-276955
ANCE01000165-6532-7426
CP008855-2708939-2709371
FR890234-16560-19549
AYYK01000008-199677-205512
BAJN01000005-128274-128903
AONC01000039-10722-15450
CP007254-2967570-2968147
CP007254-2968057-2968269
KK328862-3131324-3133418
KK328862-3138203-3138904
KK311209-8-559
CASP01000040-108132-109321
KK350903-3049864-3051501
CP002124-308956-309838
AP011952-522411-525420
JRUT01000016-65665-67523
JZWK01000003-468561-470665
LGDO01000063-14791-15125
AJVD01000004-123258-123835
AJVD01000004-123745-123957
LHFY01000019-15202-16633
CAGT01000321-17533-22614
CP000743-1121705-1123032
KK354994-1503711-1504547
JYRK01000014-95811-97365
BCOY01000089-477323-480645
JHHC01000005-131549-132799
JYQY01000007-258096-258673
JYQY01000007-258583-258795
LINS01000012-5417-6055
FM162591-3153287-3154156
BAEH01000082-11-1009
KK322452-3143305-3144002
KK356123-3119940-3121577
KK333372-59-683
ALID01000058-90946-91584
LHIH01000017-177553-178496
CP002009-245938-247616
CP009505-2277976-2281756
CP009505-2794712-2800142
CP009505-3702263-3705176
ALES01000042-123089-123668

BBSD01000028-9090-15129
JYXD01000025-6-1621
JMCG01000001-3238101-3239749
KK353357-3121485-3122631
KK353357-3115918-3117127
KK353357-3119930-3121269
KK328164-3111769-3112747
JNXC01000022-223209-224397
CP007245-2968744-2969382
KE137225-1456108-1456558
CP009524-2353260-2355301
AHTL01000051-123019-123356
CP002360-2336691-2342216
JOUV01000035-61123-62016
KK353369-2304161-2305502
KB913025-3724915-3730431
KB913025-23244-29374
AQCS01000094-135384-136084
JZTJ01000021-51130-51769
KK321513-1788379-1790176
LDCX01000018-131218-132651
ALEY01000022-258452-259090
KK317063-3116643-3118209
KK354087-2789968-2791309
KL406373-808128-809047
GL878028-4187278-4187856
LHEN01000014-122844-123746
JZTP01000033-84874-87873
AFHN01000018-42742-44966
JYRD01000029-15133-17053
CP007263-2970632-2971209
CP007263-2971119-2971331
JYWQ01000072-51-463
JHOB01000014-107717-108360
LGCY01000097-64694-65759
CUWK01000001-3124781-3126138
KQ236439-50248-50944
KK334473-0-1428
ALGT01000018-219442-220080
JOVH01000002-38605-38905
AIHK01000023-6865-8480
KB891114-2284-3528
KE386858-17486-17759
KE386858-17669-17881
KK337314-58-921
KK318143-58-895
CP003178-5986431-5987780
FR900384-3213-4581
AULT01000023-19252-21310
KK213902-141950-143166
LHHH01000030-27168-28173
KB932593-907213-907778
ACNP01000057-23-966
AVHN01000098-4625-5182

ALUA01000044-138399-139359
CP002215-1235688-1237374
GG770381-214584-216080
AHUV01000050-53-814
JQEN01000061-66991-68712
CP001281-2518070-2519746
CP001281-2519656-2520906
AOXM01000067-56-695
JOCE01000032-15975-19483
CP012513-2949889-2951016
CP007329-2971536-2972113
CP007329-2972023-2972235
KQ236553-890-1256
KQ236553-1154-1850
KQ236553-761-989
CP000453-295777-296049
KK327819-3049999-3051566
CP009439-25220-27627
CP009439-30088-32078
JWRA01000016-274559-277558
BBIJ01000011-74404-74702
BBIJ01000011-74603-75426
APXP01000161-94038-94800
LN885086-2456752-2460281
JOLW01000017-12606-13307
ANAS01000038-111706-112397
KN173625-3856469-3857353
JOES01000053-61-3326
BCAN01000026-182604-183788
CWMM01000269-50-879
LDNU01000080-4577-5121
AJPQ01000134-3507-5793
AQAB01000040-108136-108898
KK336488-58-541
AZAC01000002-35465-36334
KK331930-1651-3329
KK331930-0-1499
CP002780-1435555-1439454
CP002780-1421834-1423723
CP009149-1464713-1465474
KL406096-70864-71346
JUDC01000014-98438-99200
KK339602-3148126-3149035
JOMW01000076-202-410
JUQY01000051-0-484
JUQY01000051-384-996
AENV01000002-152860-155944
KK338394-0-693
AUVY01000013-277026-277986
KK353152-2646175-2647660
CP003249-878386-883773
KK338368-58-1400
LN865153-683220-683515
AKWD02000054-6928-7254

KK322938-13-710
CWMF01000021-52087-53510
KK357081-3121632-3122471
KK327832-1760861-1761772
KK327832-1763002-1764280
KK327832-1755302-1756070
CP011971-31992-34132
ALGY01000031-302546-303184
AJTU01000004-160710-162125
CP002683-238339-242116
AAOH01000006-214147-219636
CTDZ01000009-1798179-1800608
JOLO01000006-227664-228608
CXYH01000005-127821-128583
LGUZ01000031-66972-67853
CP002353-3141460-3167461
CASC01000006-108001-109829
KK338567-1416-1818
CP013202-965066-965556
LDZY01000011-45848-49253
KQ483404-53-1482
JOLL01000008-227664-228608
LJJK01000002-92369-94334
AQBJ01000054-43780-44419
AQDU01000111-96860-97255
BCOD01000095-261530-262152
LHJS01000007-160593-161478
CP000254-1568545-1573395
JQNC01000001-16876-18280
ALXV01000023-18545-19703
CP007252-2967818-2968030
CP007252-2967331-2967908
CDRY01000236-19913-20491
LBMP01000001-355323-364065
KK324248-135581-136363
CM001984-411673-412130
JYSI01000014-228436-230173
LGUQ01000296-318038-321236
ALES01000047-469891-470103
CP001275-31266-35139
JQKG01000090-5640-7255
AJMN01000020-419919-421136
CCUT01000001-160144-160723
GG666994-227485-233491
KK331230-1384-2379
KK319000-57-1330
CUWH01000001-3123052-3124336
KK356582-3109051-3110767
CP009501-1021519-1026905
CCLM01000001-2237315-2237955
JH993791-100431-103811
BAHU02000015-55954-56659
JODS01000065-3683-4343
JWIC01000007-584830-587319

CP001336-2361840-2365779
CP001336-2346027-2349091
KK356964-3110214-3111849
KK328383-3049604-3051177
LJXY01000006-109115-109753
KK341857-484382-485675
LBCK01000002-533825-544222
FR875178-712331-715137
AVMQ01000049-39070-41342
ALFM01000025-258842-259054
ALFM01000025-258354-258932
JQRD01000286-10507-11391
APGW01000013-420354-421571
CP013990-3585420-3587372
BCQZ01000006-193464-195506
AGFD01000058-1546-7947
JQLR01000001-4060457-4064555
KL406019-128545-129320
KK356938-1497011-1497859
KK356567-1141457-1142946
JQAF01000001-97502-98070
CCUD01000001-2421852-2422430
CWLE01000008-5805-7228
CP003267-16307-17502
KK317034-3051242-3052955
CP011546-1839143-1842015
LAZX01000009-546-1290
CP007269-2967570-2968147
CP007269-2968057-2968269
JWRE01000018-288978-291244
JYSO01000014-160597-161768
KK356896-3128724-3129134
KK356896-3129025-3129714
KK350919-2783110-2784240
LIUL01000001-37191-40124
CP001364-4543860-4549687
CP001364-709767-731306
JWHK01000065-458540-459963
JYSX01000001-361703-362769
CP002219-2613495-2618702
CP002219-2686104-2687446
KK328479-2798368-2799867
KE698887-630277-630916
ANAX01000072-10821-11399
KK339422-2782023-2783455
JAFS01000001-339102-340655
CP012073-1162281-1166079
JPMZ01000005-41606-42060
ALWB01000058-189-5265
JYPM01000002-97307-98737
JUDI01000073-0-411
GG665866-138460-140031
CP009515-2720504-2722226
LAOZ01000017-346394-347296

FR720325-2660795-2663407
JH114305-36994-37826
KE699235-163538-164726
KK357092-3143595-3145172
KK357092-3138970-3140103
KK357092-3137675-3138752
KK357092-3152420-3153401
CP009087-2970632-2971209
CP009087-2971119-2971331
AQCQ01000053-97950-98711
AE000516-3116726-3117781
CP004387-992446-995100
JNMU01000149-56-451
KI912588-43239-44790
CASF01000054-38670-39248
AAWS01000095-1599-3903
AQDK01000114-97956-98656
KK339726-1414-2039
CP002330-2745477-2746034
CP002330-2769631-2770838
HF937208-3128685-3130239
AGSL01000112-42-621
CP005926-1540674-1542593
AEZG01000002-63747-64741
APYN01000056-490-1800
LHHK01000049-90957-92572
LMWI01000002-164901-167428
KK321739-1431115-1433865
KK321739-1425793-1426716
KK339476-2795157-2795930
JJUV01000012-135192-136036
CBLP010000013-65022-66941
CZVK01000070-27072-28357
KK321519-3126963-3128767
KK321519-3128876-3130308
CUWE01000001-3061073-3062358
CATK01000001-126265-126660
LHDY01000024-5430-6861
JJPE01000147-7582-12789
KK340380-3114120-3115256
KK340380-3113915-3114224
KK340380-3108687-3109958
AAWS01000008-236656-237877
CP003639-1538046-1540340
KK318998-58-1480
CP009709-1536668-1541901
JYOM01000001-6033-7257
HG425166-392575-392979
HG425166-392870-393342
JQKJ01000013-70504-72652
AYGY02000192-9185-11676
KK330851-92240-92652
CP000142-1134548-1141347
KK339254-1207134-1210254

KL407037-42-308
KK328327-810478-811389
KK321352-58-821
JHRY01000220-243-455
AENO01000039-4033-5945
AP012053-1484495-1485257
AP012053-1485155-1486444
AFSL01000113-748-1233
AFSL01000113-1714-2262
AFSL01000113-5351-6097
AFSL01000113-3844-4334
JJPB01000021-5156-12774
ALAS01000231-4621-9854
LHIJ01000022-225354-226359
LHHI01000010-125758-126885
KQ948222-79746-82824
LHOV01000004-226721-227726
KK106988-1705565-1706325
CP003184-875548-876718
KK328381-1761955-1763596
KK328887-3133801-3135324
KK328887-3138387-3139230
KK328887-3122952-3124971
KK332738-295-1246
CP007804-3007746-3009361
KK339571-1505-2634
KK329669-5540-6254
KK329669-4432-5652
CWKM01000032-6310-7733
JQEV01000071-1579-3300
KK338778-490890-492100
KK338778-484127-485181
KK339163-23-852
KK339163-3963-5019
JYXX01000003-88301-89916
KK322751-3110220-3111281
CWMQ01000110-6447-7870
BASG01000021-35400-36368
KK328917-308657-310817
KK336916-1414-2987
CP003532-2471272-2476498
KK327779-3046743-3047953
KK327779-3048139-3048685
AUQV01000001-313960-315332
KK342425-147693-148836
KK317053-3120441-3121279
KK317053-3118414-3120200
CP001144-3137471-3137743
AKFX01000004-23474-24051
AKFX01000004-23961-24173
KK338869-56-1263
DS990370-316929-317163
ABXY01000010-32-366
ALXN01000038-74673-76623

CP003592-2213432-2214421
KK328372-820437-821506
KK328372-821674-822522
CP003386-3115516-3116827
KK333223-58-828
AQAA01000307-92216-92978
JYQA01000038-88301-89916
CCUO01000002-1788356-1788750
CP003537-898722-903504
AIGG01000057-114332-114725
LHKB01000042-6-1437
CP009086-2971302-2971514
CP009086-2970815-2971392
CEGO01000019-74069-78525
CP000679-356503-357986
KI519076-1997744-1998671
JATC01000006-656154-661665
KK338554-0-550
JSVJ02000135-495-1029
AIHR01000024-132727-134038
JANJ01000004-247393-248485
JSRK01000099-231356-231997
APYK01000156-480-1790
CP009716-27294-28117
GG729933-172952-178891
HG313760-64685-66421
AOXY01000018-16789-17673
JQAI01000001-97502-99809
JQET01000071-2238-3959
LHJP01000005-52471-53476
LHES01000008-51088-52093
KK073872-3009130-3009430
CP004350-953569-959210
LIMM01000049-50-1196
KE700470-533432-533888
CP002659-1302006-1306491
KK350956-0-987
AQXD01000001-940589-941035
LPZJ01000216-94299-94755
CP002521-4426773-4430214
AOHC02000012-25758-26804
KI912107-5306352-5307449
AYUQ01000065-98128-100353
CP002057-582646-594279
KK353321-3123462-3124365
CASA01000002-109760-110276
JZTN01000037-126612-129611
AZXB01000038-41814-44180
ANRI01000006-24722-26276
JHDG01000149-96332-96788
CCQR01000046-9834-10168
KK328714-1619283-1620856
CM002757-2569526-2571386
JPFQ01000053-67141-68862

JYNR01000001-2260666-2265789
KK353329-3102889-3103954
KB733436-160895-161778
KB733069-125194-125833
LIQU01000091-28401-31603
JZUS01000002-513957-516180
LOOT01000013-167867-168131
JRZA01000003-458427-459651
KL537463-2124-4605
CP007133-3425901-3426419
JNBN01000066-173169-173808
CP001367-67423-76153
KK357243-1134458-1137353

Three sequential consecutive spacers were found in:

KK353227-3148813-3150540
APXL01000411-90861-91683
AROB01001369-995-1817
KB896854-2040-4793
LINP01000003-258362-259002
LLYY01000010-22680-27963
LRNI01000001-16829-18138
CASW01000046-125983-126720
KK328103-1761177-1762665
FR901410-12458-17320
KK328084-318723-319926
KK334413-136240-137522
LIOH01000003-422379-423082
LHDU01000002-470270-470973
ALFU01000030-302574-303335
ALFK01000035-469926-470687
CP007505-4494384-4495815
CARY01000006-125888-126831
AEXT01000007-252142-253102
CP006019-735976-739414
KK327775-1308419-1309702
CP000252-2747385-2752983
ALFP01000034-423606-424367
CP001787-1101871-1102591
CP001787-886912-888224
JTBM01000001-3511-4214
KK332861-0-690
LIMX01000001-94632-95335
ALGS01000023-5509-6270
KK356866-2660-4441
KK356866-63-1191
KK354298-1125544-1126380
CP000816-955648-957104
KK327802-806996-808128
CAAD010000050-80118-80719
CP001901-1207511-1208848
ALXQ01000023-72484-74578
KK339319-3124644-3125466
ALEO01000012-469922-470683
ALGV01001139-3274-4035
ACDY02000005-83370-84527
KK322665-809017-810153
KK322667-3104240-3106699
CP003969-610406-614540
AROG01000346-2444-2900
CASH01000036-108084-108794
KK328107-1782506-1783790
KB897534-9319-11563
CASS01000001-92433-93134
LMVI01000001-765020-765850
ALGX01000035-302556-303317
AOXQ01000101-17224-18654

KK355876-3126116-3127469
CP003944-2987978-2988229
BA000023-32701-39895
LINV01000001-470057-470760
KL476895-98-1161
FR891471-0-1587
KK328500-2924653-2925053
ALXP01000028-85285-86917
KK354757-3069126-3070464
AUSV01000051-9674-10604
CP006714-1641127-1643189
CP002791-108233-109011
LFOF01000120-55-1469
LACN01000012-71008-72560
KK317046-3112091-3113360
AOGJ01000015-112951-114627
CARS01000006-108123-109452
KK328515-137891-139164
CARW01000044-109549-110088
KK337223-3-1405
ALFQ01000018-470041-470802
CATW01000001-272097-272824
KK327738-3118005-3118774
LHLJ01000002-470057-470760
KK316833-3134505-3135121
KK319679-0-480
KB905235-14581-17916
CZVN01000096-51-2748
KK338497-1615624-1616213
AZQN01000005-151202-153939
KK340417-3121301-3122582
CP006965-855388-857656
KK327881-481423-482341
KK354233-3115002-3116283
KK311638-64250-65380
KK327809-812327-813689
KB733036-829713-830047
CATT01000008-35130-36332
KK357085-3139333-3140546
CP002830-2379260-2379849
KK353357-3117369-3118495
KK339156-483492-484697
CASI01000005-108299-109431
LHDN01000002-470057-470760
KK332200-351-1254
KE150483-761819-762488
AUPX01000124-377-1517
KK339459-664813-666096
ALXT01000023-72244-74470
JYPN01000001-5416-6119
KK353689-456444-456978
ALGF01000028-470037-470798
DS264459-428611-436130
AEZG01000002-66210-68361

AKFY01000027-5042-5803
AKYU01000130-33-645
ALGE01000024-470040-470801
LIMO01000013-51084-51787
CASA01000002-126616-127366
KK329014-70865-71417
